# Supplementary figures and images for: Single-cell transcriptomic profiling of C. elegans Q neuroblast lineage during migration and differentiation
Source: PLoS One. 2026 Mar 3;21(3):e0343734. doi: 10.1371/journal.pone.0343734 (PMC12956130; doi:10.1371/journal.pone.0343734)

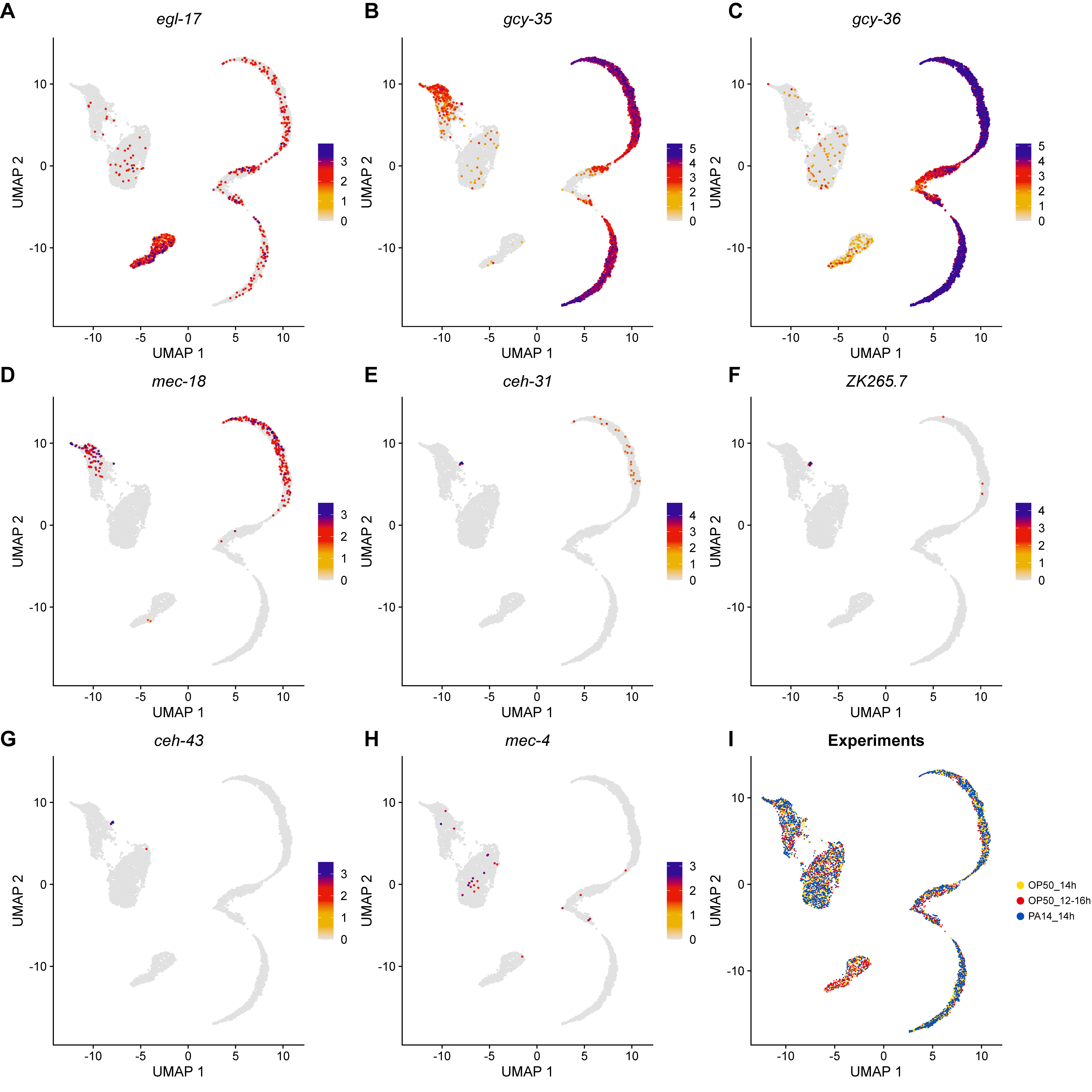

Supplement: S1 Fig — (TIF) [file pone.0343734.s002.tif]

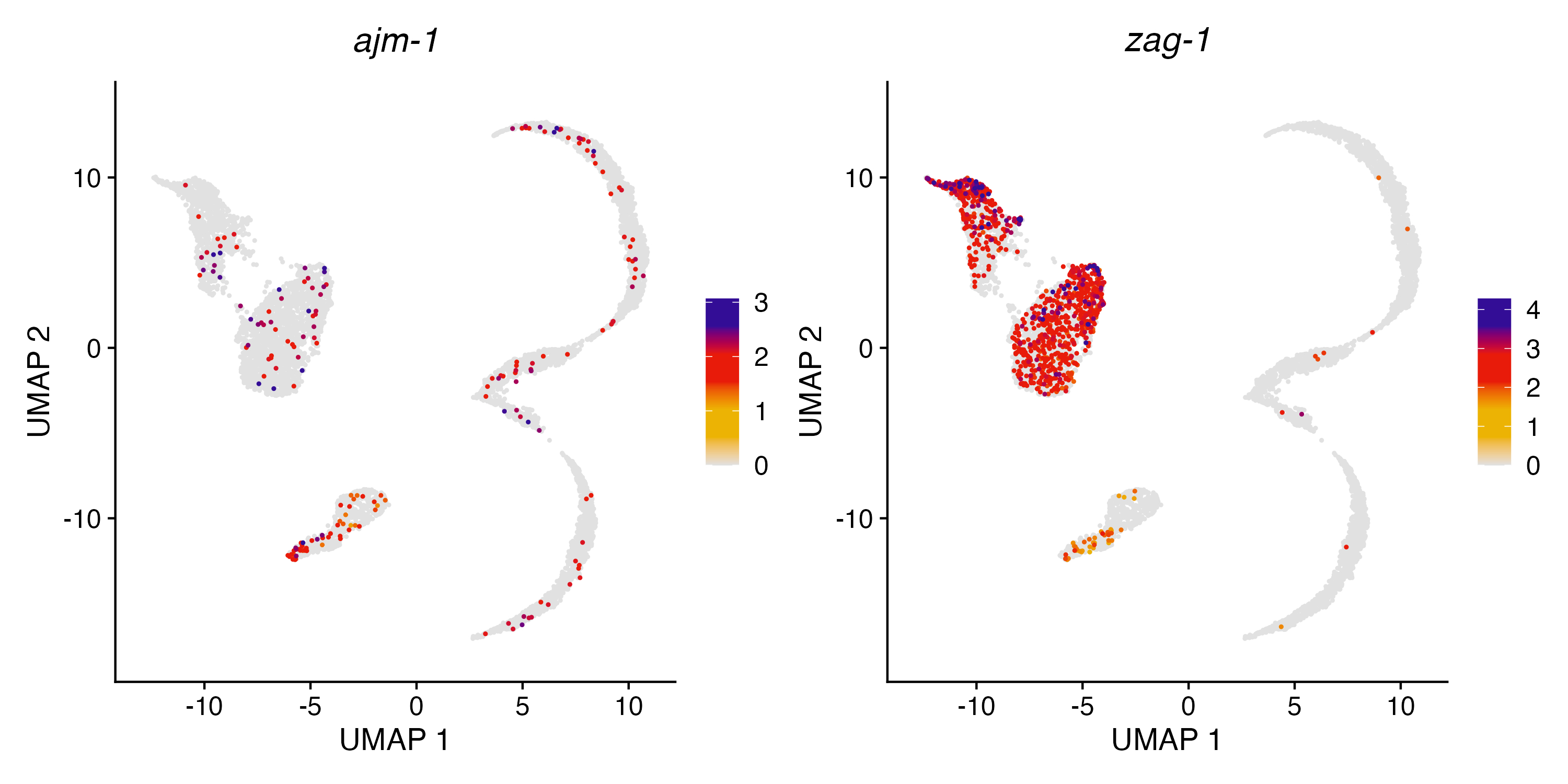

Supplement: S2 Fig — (TIFF) [file pone.0343734.s003.tiff]

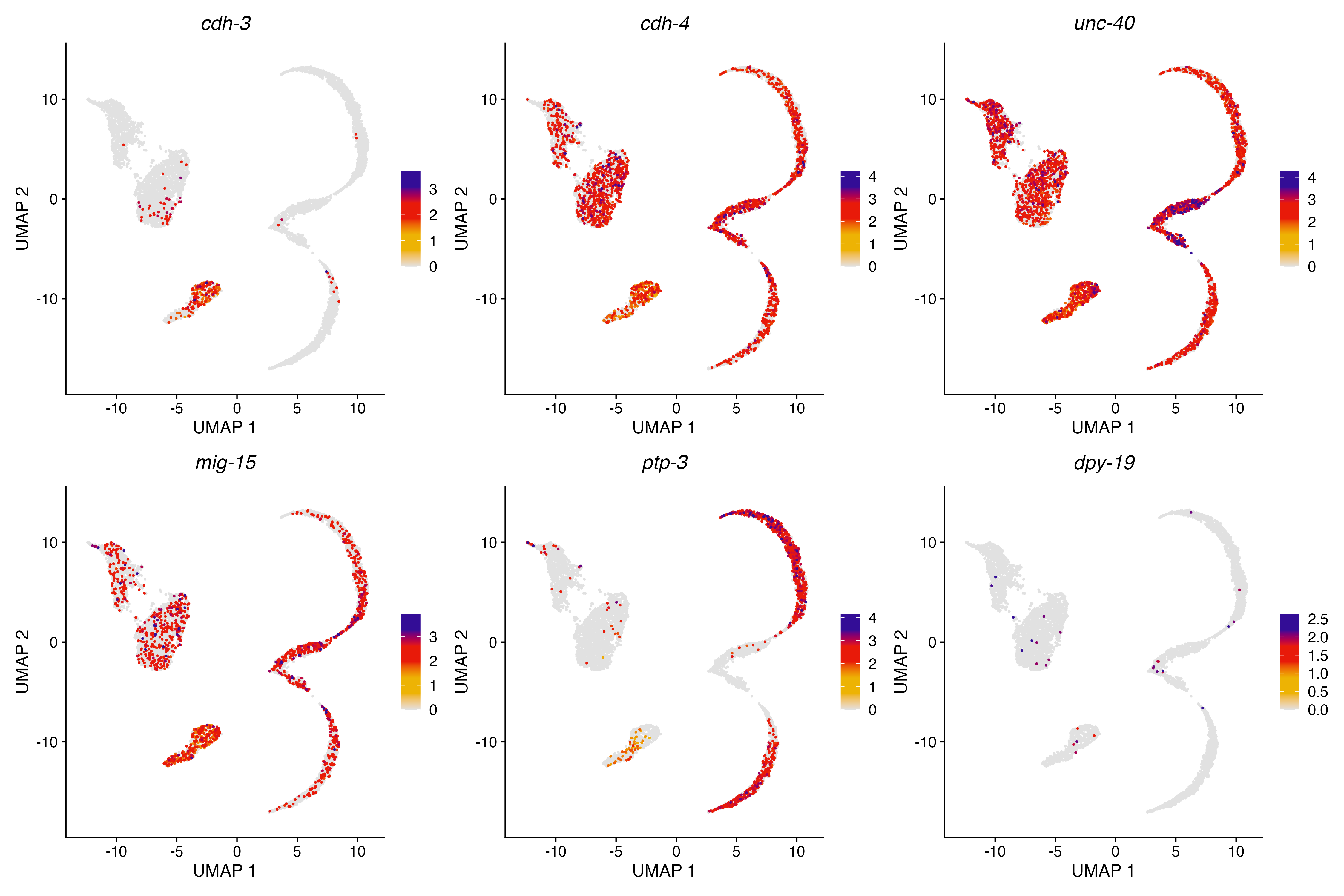

Supplement: S3 Fig — (TIFF) [file pone.0343734.s004.tiff]

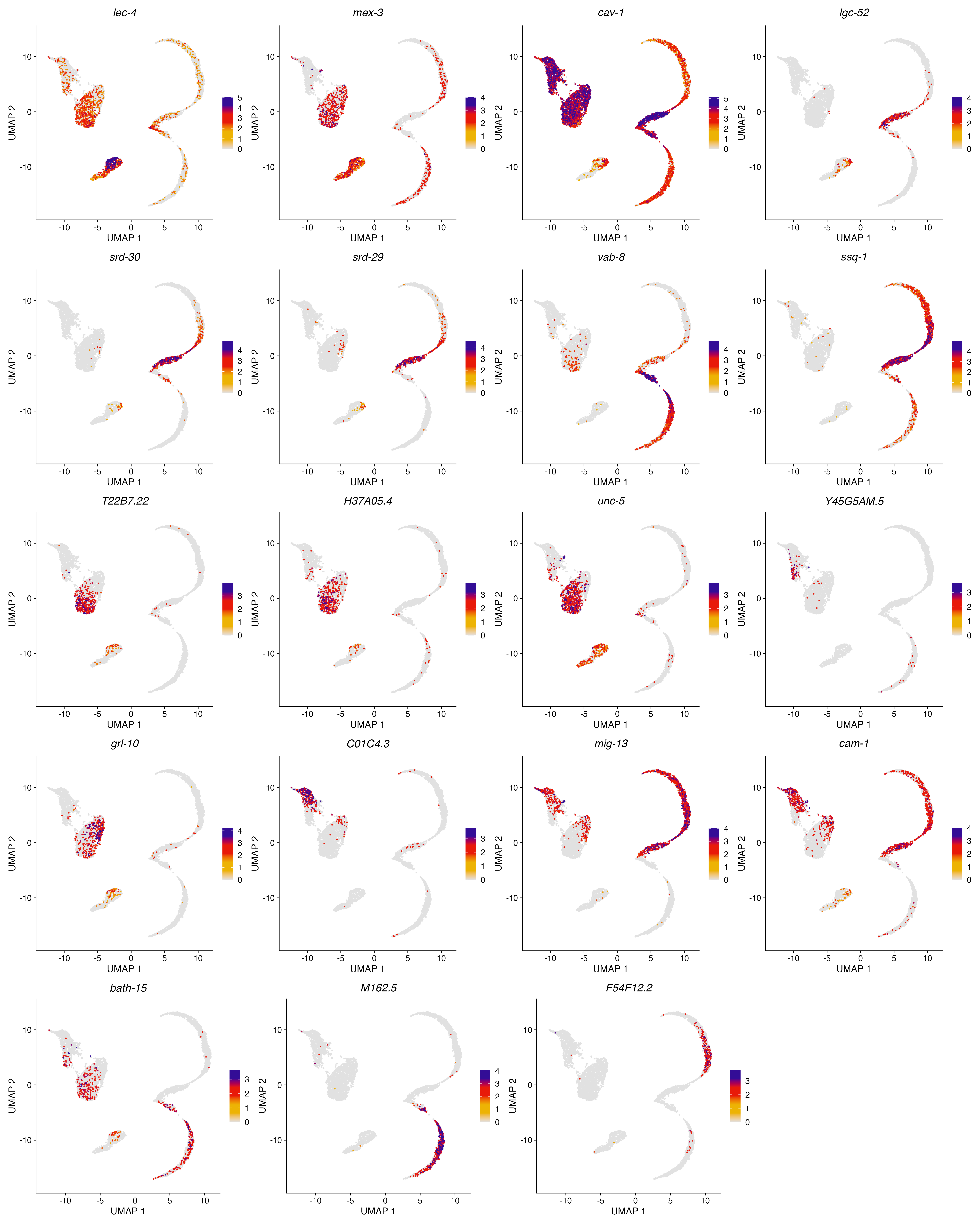

Supplement: S4 Fig — (TIFF) [file pone.0343734.s005.tiff]

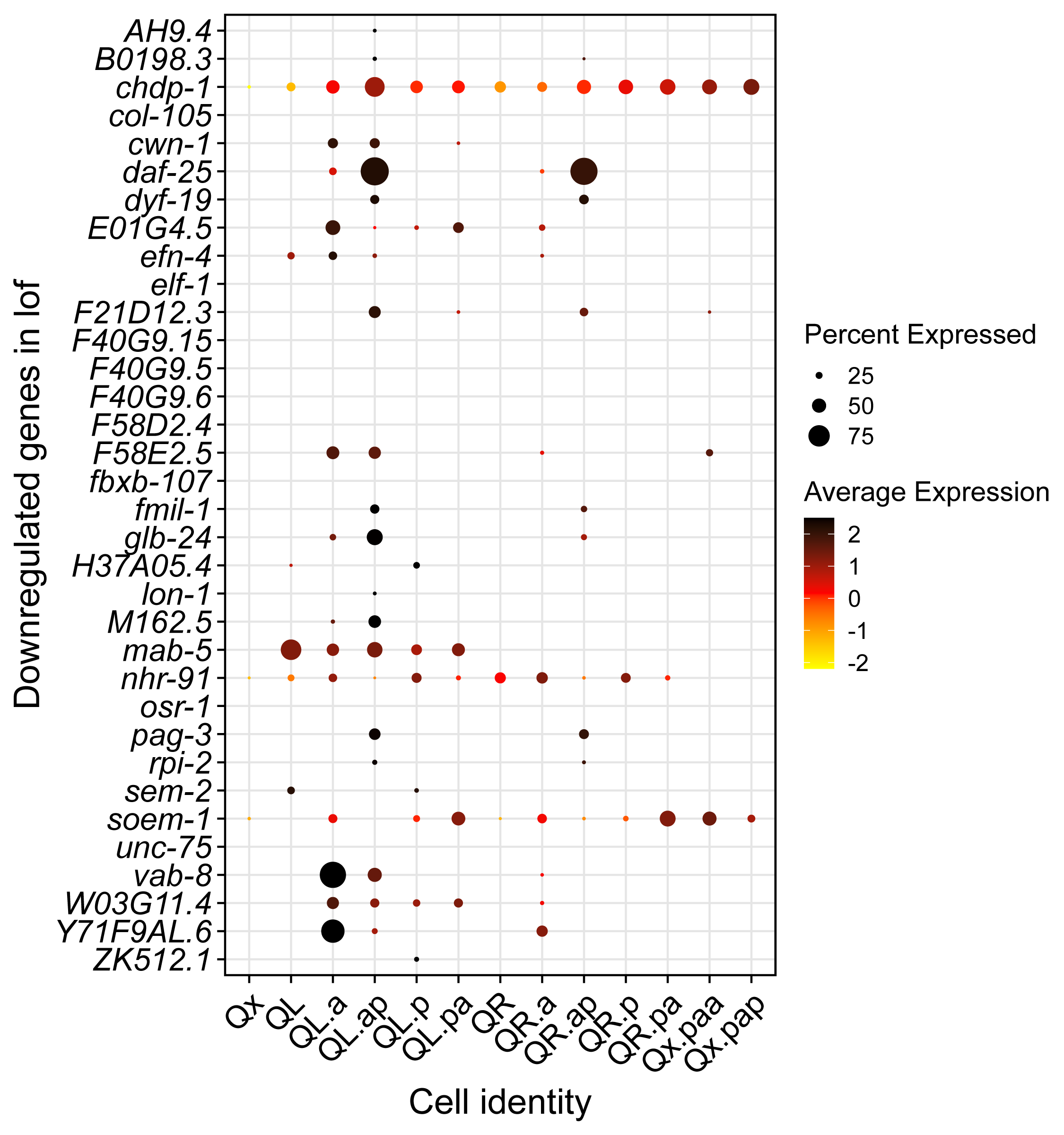

Supplement: S5 Fig — (TIF) [file pone.0343734.s006.tif]

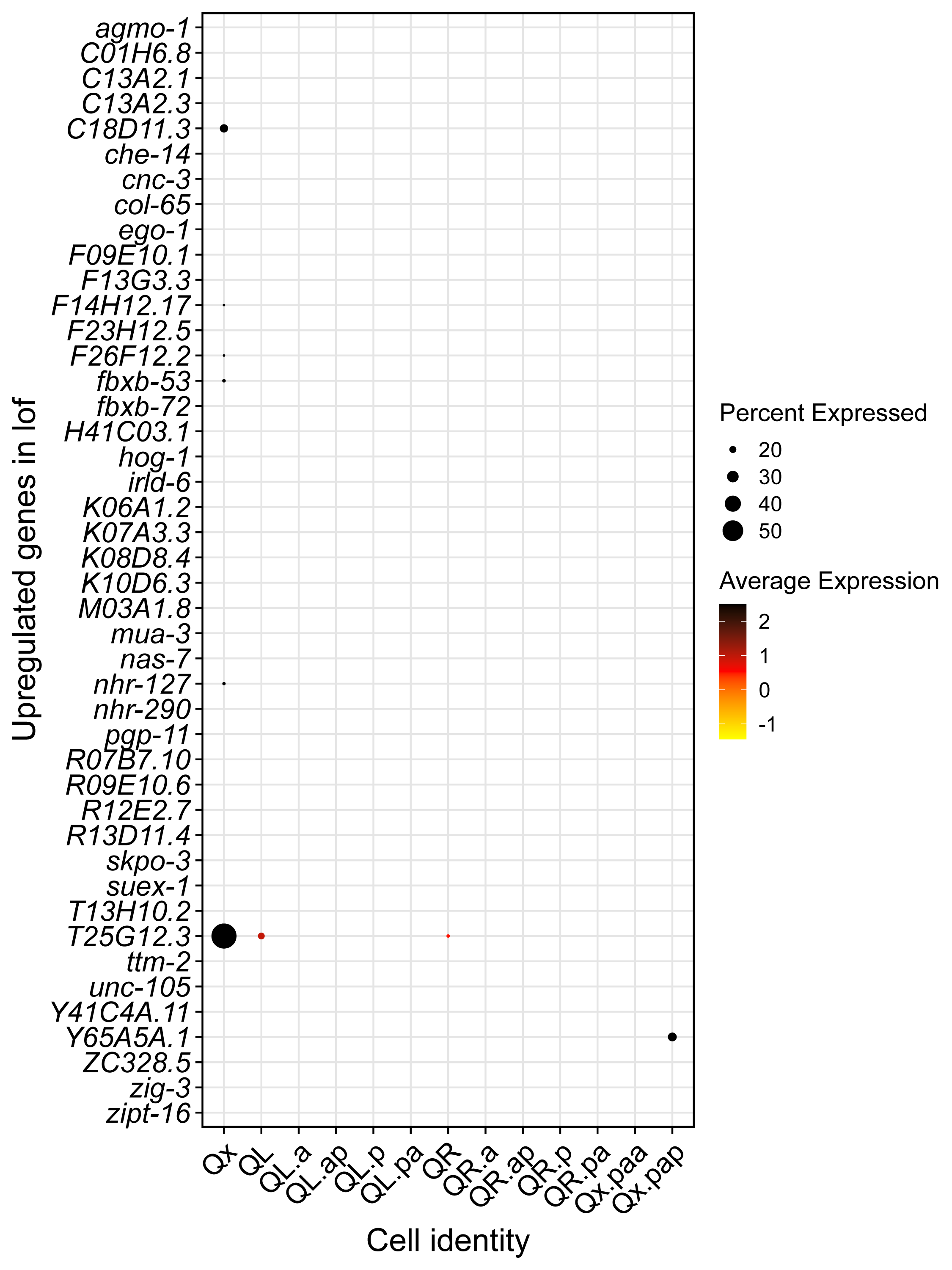

Supplement: S6 Fig — (TIF) [file pone.0343734.s007.tif]

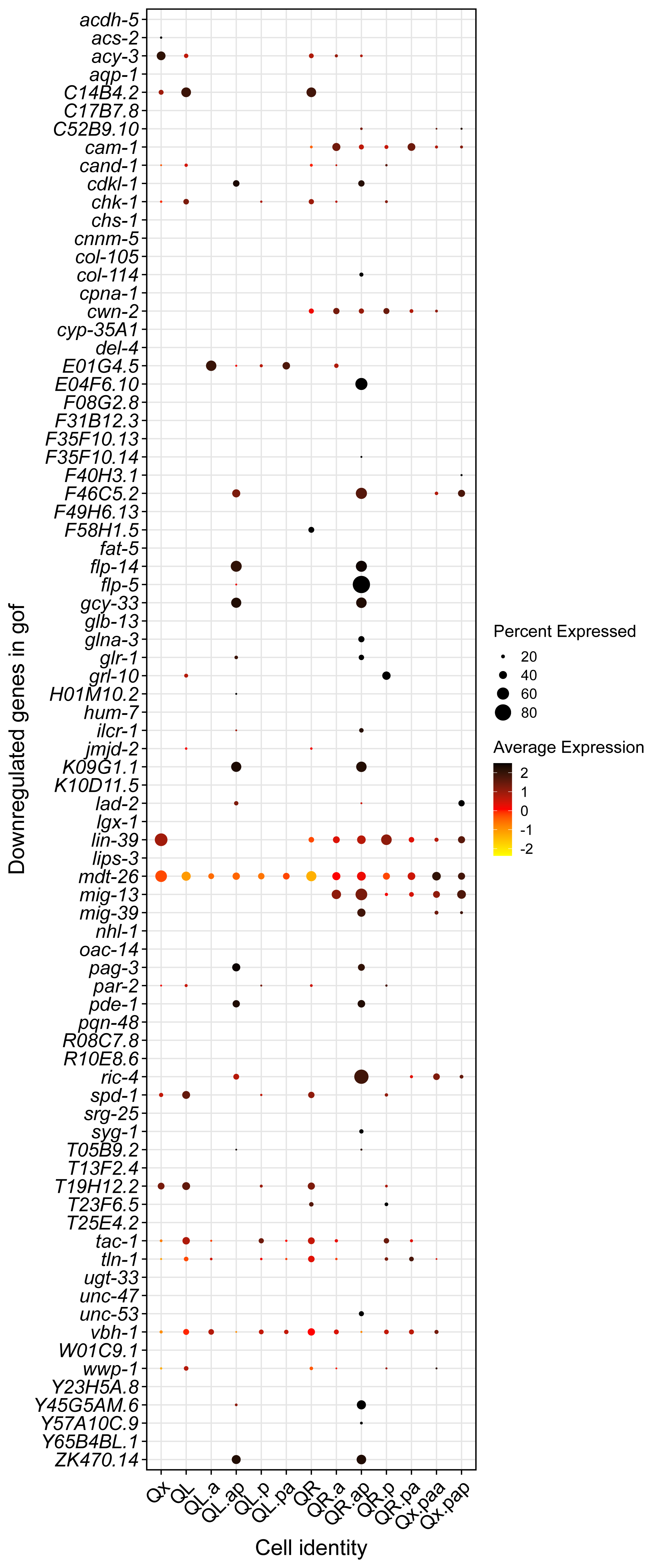

Supplement: S7 Fig — (TIF) [file pone.0343734.s008.tif]

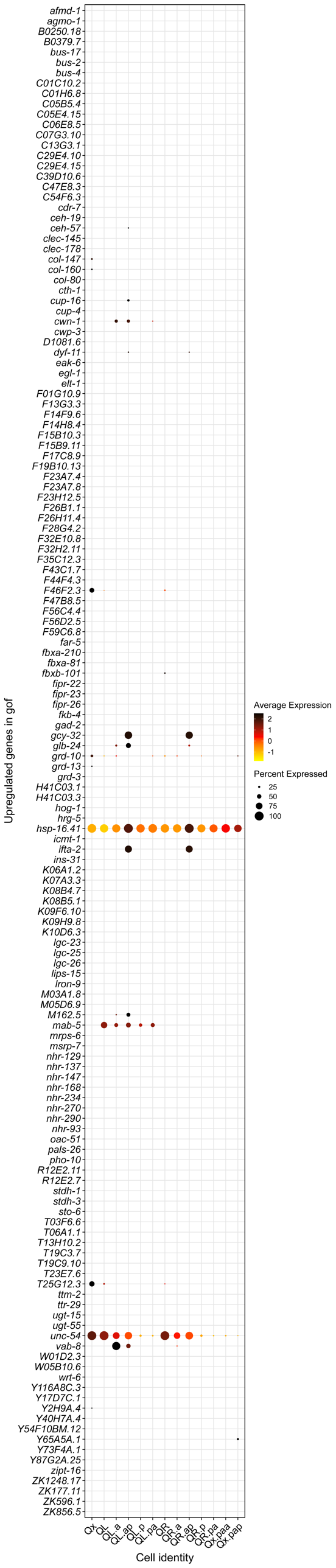

Supplement: S8 Fig — (TIF) [file pone.0343734.s009.tif]

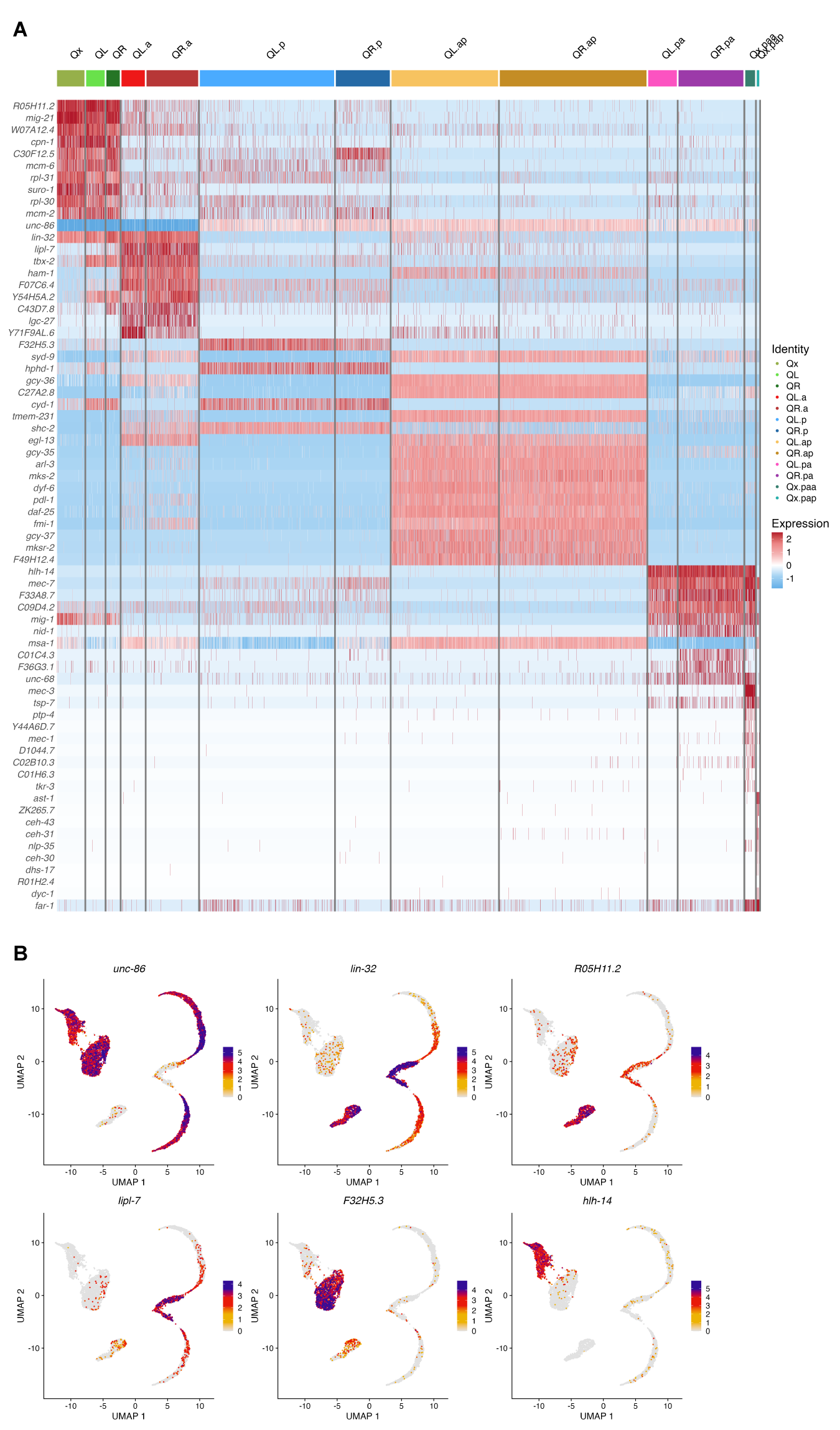

Supplement: S9 Fig — (A) Heatmap showing the top 10 markers for the clusters displayed in Fig 5B, presented here with left-right segregation. (B) UMAP plots showing the expression patterns of selected marker genes across the Q lineage. (TIF) [file pone.0343734.s010.tif]

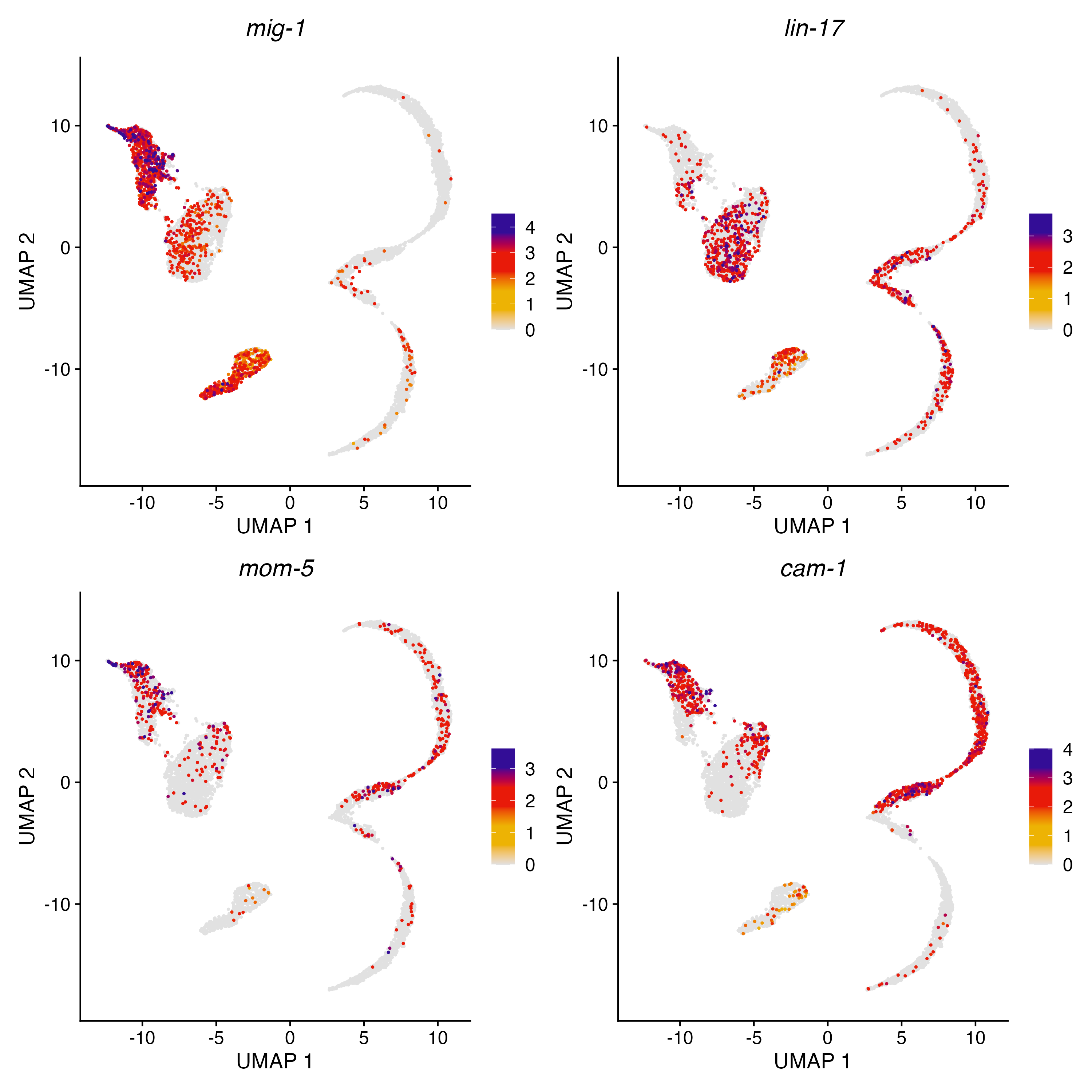

Supplement: S10 Fig — (TIFF) [file pone.0343734.s011.tiff]
