# Supplementary material for: Single-cell transcriptomic profiling of C. elegans Q neuroblast lineage during migration and differentiation
Source: PLoS One. 2026 Mar 3;21(3):e0343734. doi: 10.1371/journal.pone.0343734 (PMC12956130; doi:10.1371/journal.pone.0343734)
Supplement: S7 File — (DOCX) [file pone.0343734.s018.docx]

S7 File

**Note**: this notebook depends on the output from CellRanger in the [scrnaseq v2.6.0 pipeline from nf-core](https://nf-co.re/scrnaseq/2.6.0/), organized by the sequencing run and sample. You may need to adjust the file paths for various functions reading inputs and writing outputs, depending on how you’ve organized your project directory.

# -

# OP50 Data (WT_Q_cells_1_OP50_14h)

OP50.data <- Read10X(data.dir = "cellranger_output/2024_07_22_data/count/OP50/outs/filtered_feature_bc_matrix")

OP50 <- CreateSeuratObject(counts = OP50.data, project = "OP50")

Warning: Feature names cannot have underscores ('_'), replacing with dashes
('-')

OP50

An object of class Seurat
22113 features across 2528 samples within 1 assay
Active assay: RNA (22113 features, 0 variable features)
 1 layer present: counts

OP50 <- NormalizeData(OP50)

Normalizing layer: counts

OP50 <- FindVariableFeatures(OP50)

Finding variable features for layer counts

op50_features <- rownames(OP50)
OP50 <- ScaleData(OP50, features = op50_features)

Centering and scaling data matrix

OP50 <- RunPCA(OP50)

PC_ 1
Positive: F58B4.2, T04C12.9, T01D3.1, WBGene00014307, guk-1, flp-14, gcy-33, dyf-18, hsp-70, egl-21
 pde-1, ZK470.14, flp-5, Y102A11A.9, nlp-43, lfi-1, F58H1.7, T27C4.1, pgal-1, E04F6.10
 ZK742.7, ins-18, C33A12.4, abts-1, F59B10.2, fkh-5, B0205.14, glb-1, gasr-8, F18A11.5
Negative: rpl-7A, rps-1, col-3, col-103, sams-1, rps-4, rpl-12, rps-14, rpl-32, rpl-31
 nlp-24, eef-1A.1, F46F2.3, col-117, rpl-3, col-98, rpl-11.2, eef-2, rpl-7, rps-23
 rpl-20, rpl-14, nlp-29, rps-21, rpl-1, rps-0, rpl-6, rpl-30, rpl-21, rpl-10
PC_ 2
Positive: cpn-3, unc-15, let-2, T21B6.3, Y71H2B.4, ttn-1, cpl-1, EGAP4.1, mlc-1, pqn-48
 zyx-1, csq-1, ttr-16, unc-22, pck-1, D1086.5, T04A6.1, unc-87, R13H4.2, tsp-8
 K02F3.9, ccg-1, T23E7.2, ZK809.8, Y37D8A.2, CC8.2, dig-1, upb-1, cpna-2, pqn-24
Negative: rbm-3.2, rpl-12, his-32, F32H5.3, Y54H5A.2, rpl-32, rpl-7A, ztf-11, tost-1, ctf-8
 rpl-3, rpl-18, smo-1, C30F12.5, mec-7, eef-2, rpl-41.2, rpl-19, rpl-24.1, rod-1
 eef-1A.1, C37A2.7, rpl-43, tbx-2, eef-1G, tyms-1, rps-11, R05H11.2, tbca-1, rack-1
PC_ 3
Positive: rpl-12, F32H5.3, his-32, rbm-3.2, F46H5.3, mec-7, ztf-11, ctf-8, tbx-2, tost-1
 Y54H5A.2, F33A8.7, tbca-1, eef-1A.1, rpl-41.2, rpl-7A, smp-1, eef-2, C43D7.8, rpl-32
 ant-1.1, smo-1, emb-9, memb-2, nid-1, grl-10, bub-3, Y71H2B.4, T21B6.3, sem-2
Negative: F58B4.2, gcy-33, T04C12.9, T01D3.1, flp-14, WBGene00014307, pde-1, guk-1, egl-21, sams-1
 col-117, dyf-18, col-98, ZK470.14, col-3, nlp-29, nlp-43, col-94, glb-1, ins-18
 col-10, nlp-24, flp-5, pgal-1, upp-1, col-103, pmt-2, F26G1.2, ttr-15, F58H1.7
PC_ 4
Positive: egl-46, mec-7, F33A8.7, T01D3.3, sams-1, col-117, nid-1, col-98, ram-2, K09F6.13
 nlp-29, K07E3.4, F58E2.5, grd-3, col-94, pmt-2, col-10, WBGene00023302, F26G1.2, mec-3
 tsp-7, nlp-33, far-1, grd-13, col-3, gln-3, col-103, ttr-15, R09E12.9, T14A8.2
Negative: cpn-1, F46H5.3, R05H11.2, pde-1, nlp-43, gcy-33, ins-18, T05A8.3, F58H1.7, flp-14
 egl-17, WBGene00022730, unc-54, pgal-1, glb-1, ctf-8, smo-1, tyms-1, dgk-5, B0205.14
 plc-1, nphp-2, F32H5.3, glna-3, egl-21, rps-20, T05B9.2, emb-9, rpl-21, F58F9.1
PC_ 5
Positive: Y39B6A.5, inos-1, clec-178, cpi-1, cup-4, Y73F4A.1, lgc-26, T19C3.5, lgc-25, Y116A8C.3
 mig-6, unc-122, dig-1, ccg-1, F56C4.4, Y54F10BM.12, K02E7.11, F32E10.8, lgc-23, cof-2
 far-1, ZK593.3, lbp-2, cubn-1, C29E4.10, F35C12.7, C18A11.1, K07E3.4, W05B10.6, M01H9.3
Negative: T01D3.3, egl-46, WBGene00023302, F58E2.5, K09F6.13, gasr-8, unc-54, ssq-1, Y71F9AL.6, vab-8
 M162.5, smp-1, lpr-6, F35B3.7, srd-30, F18A11.5, lat-1, lon-1, C33A12.4, tbx-2
 R57.2, F59B10.2, Y54H5A.2, dyf-19, scp-1, hsp-70, sng-1, sca-1, slc-25A46, fkh-5

ElbowPlot(OP50, ndims = 50)


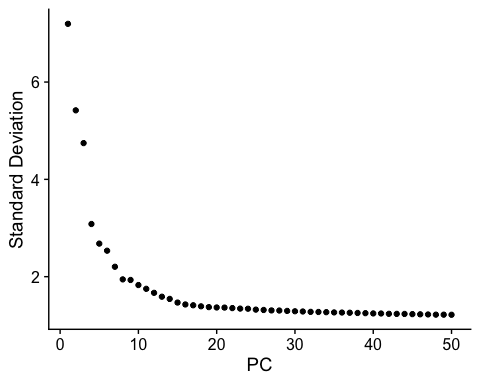


OP50 <- FindNeighbors(OP50, dims = 1:15, reduction = "pca")

Computing nearest neighbor graph

Computing SNN

OP50 <- FindClusters(OP50, resolution = 1.0, cluster.name = "OP50.unintegrated_clusters")

Modularity Optimizer version 1.3.0 by Ludo Waltman and Nees Jan van Eck

Number of nodes: 2528
Number of edges: 88835

Running Louvain algorithm...
Maximum modularity in 10 random starts: 0.8323
Number of communities: 16
Elapsed time: 0 seconds

OP50 <- RunUMAP(OP50, dims = 1:15, reduction = "pca", reduction.name = "umap.OP50.unintegrated")

Warning: The default method for RunUMAP has changed from calling Python UMAP via reticulate to the R-native UWOT using the cosine metric
To use Python UMAP via reticulate, set umap.method to 'umap-learn' and metric to 'correlation'
This message will be shown once per session

11:04:32 UMAP embedding parameters a = 0.9922 b = 1.112

11:04:32 Read 2528 rows and found 15 numeric columns

11:04:32 Using Annoy for neighbor search, n_neighbors = 30

11:04:32 Building Annoy index with metric = cosine, n_trees = 50

0% 10 20 30 40 50 60 70 80 90 100%

[----|----|----|----|----|----|----|----|----|----|

**************************************************|
11:04:32 Writing NN index file to temp file /var/folders/2_/0b7d0hy11bd2g2nl32tfghfh981cxm/T//RtmpZVhw0e/file18f3102f87f5
11:04:32 Searching Annoy index using 1 thread, search_k = 3000
11:04:33 Annoy recall = 100%
11:04:33 Commencing smooth kNN distance calibration using 1 thread with target n_neighbors = 30
11:04:34 Initializing from normalized Laplacian + noise (using RSpectra)
11:04:34 Commencing optimization for 500 epochs, with 100638 positive edges
11:04:34 Using rng type: pcg
11:04:37 Optimization finished

op50_clusters <- as.character(OP50$seurat_clusters)
names(op50_clusters) <- as.character(names(OP50$seurat_clusters))

## DropletUtils

Note: Mostly just following the [vignette](https://bioconductor.org/packages/release/bioc/vignettes/DropletUtils/inst/doc/DropletUtils.html)

OP50.data <- Read10X(data.dir = "cellranger_output/2024_07_22_data/count/OP50/outs/filtered_feature_bc_matrix")

op50_mol_info <- read10xMolInfo("cellranger_output/2024_07_22_data/count/OP50/outs/molecule_info.h5")

### Downsampling

set.seed(100)
op50_withsample <- downsampleReads("cellranger_output/2024_07_22_data/count/OP50/outs/molecule_info.h5", prop = 0.5)
sum(op50_withsample)

[1] 3936559

op_out <- barcodeRanks(op50_withsample)
plot(op_out$rank, op_out$total, log="xy", xlab="Rank", ylab="Total")

Warning in xy.coords(x, y, xlabel, ylabel, log): 13215 y values <= 0 omitted
from logarithmic plot

o <- order(op_out$rank)
lines(op_out$rank[o], op_out$fitted[o], col="red")

abline(h=metadata(op_out)$knee, col="dodgerblue", lty=2)
abline(h=metadata(op_out)$inflection, col="forestgreen", lty=2)
legend("bottomleft", lty=2, col=c("dodgerblue", "forestgreen"),
 legend=c("knee", "inflection"))


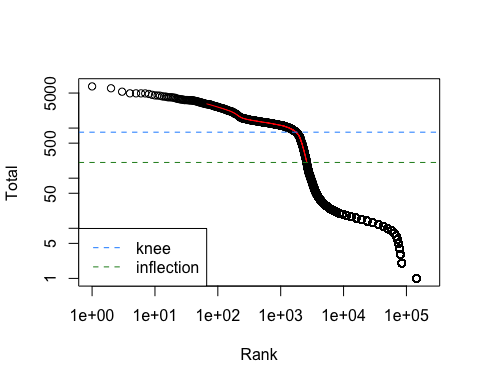


op50_empty <- emptyDrops(op50_withsample, lower = 50)

op50_isCell <- op50_empty$FDR <= 0.01

table(Limited=op50_empty$Limited, Significant = op50_isCell)

Significant
Limited FALSE TRUE
 FALSE 765 62
 TRUE 0 2840

plot(op50_empty$Total, -op50_empty$LogProb, col=ifelse(op50_isCell, "red", "black"),
 xlab="Total UMI count", ylab="-Log Probability")


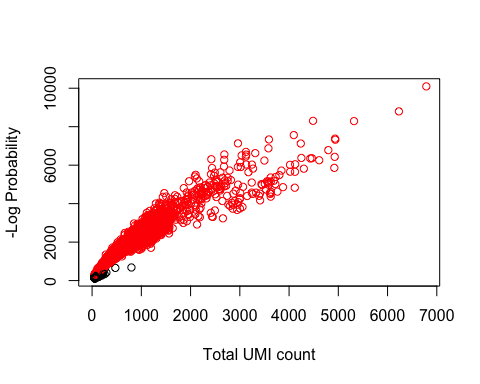


op50_empty %>%
 as.data.frame() %>%
 rownames_to_column(., var = "barcode") %>%
 filter(., FDR <= 0.01) %>%
 dplyr::select(., barcode) %>%
 as_vector() %>%
 unname() -> op50_nonempty

## SoupX

Mostly following the [vignette](https://cran.r-project.org/web/packages/SoupX/vignettes/pbmcTutorial.html)

op50_toc <- Seurat::Read10X("cellranger_output/2024_07_22_data/count/OP50/outs/filtered_feature_bc_matrix")
op50_tod <- Seurat::Read10X("cellranger_output/2024_07_22_data/count/OP50/outs/raw_feature_bc_matrix/")
op50_sc <- SoupChannel(op50_tod, op50_toc, calcSoupProfile = FALSE)
op50_sc = estimateSoup(op50_sc, soupRange = c(0, 25))

op50_sc <- setClusters(op50_sc, op50_clusters)

op50_sc <- autoEstCont(op50_sc)

1206 genes passed tf-idf cut-off and 501 soup quantile filter. Taking the top 100.

Using 1329 independent estimates of rho.

Estimated global rho of 0.01


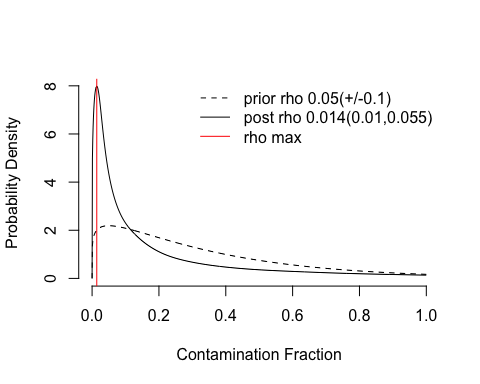


op50_out <- adjustCounts(op50_sc)

Warning in sparseMatrix(i = out@i[w] + 1, j = out@j[w] + 1, x = out@x[w], :
'giveCsparse' is deprecated; setting repr="T" for you

Expanding counts from 16 clusters to 2528 cells.

cntSoggy = rowSums(op50_sc$toc > 0)
cntStrained = rowSums(op50_out > 0)
mostZeroed = tail(sort((cntSoggy - cntStrained)/cntSoggy), n = 10)
mostZeroed

col-94 col-117 grd-10 nlp-29 col-98 phat-8 dod-6
0.07200000 0.07500000 0.08522727 0.09271523 0.12000000 0.14285714 0.17142857
 phat-7 col-107 ram-2
0.26315789 0.34773663 0.54931715

tail(sort(rowSums(op50_sc$toc > op50_out)/rowSums(op50_sc$toc > 0)), n = 20)

Y71H2AM.29 F54H12.10 E02H9.13 H04D03.7 W04B5.8
 1 1 1 1 1
 Y39A3B.8 Y54F10BM.22 WBGene00306069 ctb-1 nduo-6
 1 1 1 1 1
WBGene00010958 WBGene00010959 atp-6 nduo-2 ctc-3
 1 1 1 1 1
 nduo-4 ctc-1 ctc-2 nduo-3 nduo-5
 1 1 1 1 1

DropletUtils::write10xCounts("output/all_fed_dataset/postprocess/OP50", op50_out)

## Creating Seurat object and initial QC

OP50.data <- Read10X(data.dir = "output/all_fed_dataset/postprocess/OP50/")
OP50 <- CreateSeuratObject(counts = OP50.data, project = "OP50", min.cells = 3, min.features = 100)

Warning: Feature names cannot have underscores ('_'), replacing with dashes
('-')

Note: the following analyses use a list of mitochondrial genes that we generated with the following code in a bash terminal, using the [Ensembl v110 annotation](https://ftp.ensembl.org/pub/release-110/gtf/caenorhabditis_elegans/Caenorhabditis_elegans.WBcel235.110.gtf.gz)

cat Caenorhabditis_elegans.WBcel235.110.gtf | awk '$1 == "MtDNA"' | awk '$3 == "gene"' | cut -f 9 | cut -f 1 -d ';' | sed -e 's/gene_id "//g;s/"//g' > mt_gene_list.tsv

# Add mitochondrial features annotation
left_join(
 read_delim("mt_gene_list.tsv", delim = "\t", col_names = "gene"),
 read_delim("output/all_fed_dataset/postprocess/OP50/genes.tsv", delim = "\t",
 col_names = c("wb", "gene", "desc")),
 by = c("gene" = "wb")) %>%
 filter(., !is.na(gene.y)) %>%
 dplyr::select(., gene.y) %>%
 as_vector() %>%
 unname() -> mt_genes

Rows: 36 Columns: 1
── Column specification ────────────────────────────────────────────────────────
Delimiter: "\t"
chr (1): gene

ℹ Use `spec()` to retrieve the full column specification for this data.
ℹ Specify the column types or set `show_col_types = FALSE` to quiet this message.
Rows: 22113 Columns: 2
── Column specification ────────────────────────────────────────────────────────
Delimiter: "\t"
chr (2): wb, gene

ℹ Use `spec()` to retrieve the full column specification for this data.
ℹ Specify the column types or set `show_col_types = FALSE` to quiet this message.

# Set the “percent.mt” variable of the Seurat object
OP50[["percent.mt"]]<- PercentageFeatureSet(OP50, features = mt_genes)
# Visualize QC metrics as a violin plot before QC
VlnPlot(OP50, features = c("nFeature_RNA", "nCount_RNA", "percent.mt"), ncol = 3)

Warning: Default search for "data" layer in "RNA" assay yielded no results;
utilizing "counts" layer instead.

Warning: The `slot` argument of `FetchData()` is deprecated as of SeuratObject 5.0.0.
ℹ Please use the `layer` argument instead.
ℹ The deprecated feature was likely used in the Seurat package.
 Please report the issue at <https://github.com/satijalab/seurat/issues>.

Warning: `PackageCheck()` was deprecated in SeuratObject 5.0.0.
ℹ Please use `rlang::check_installed()` instead.
ℹ The deprecated feature was likely used in the Seurat package.
 Please report the issue at <https://github.com/satijalab/seurat/issues>.


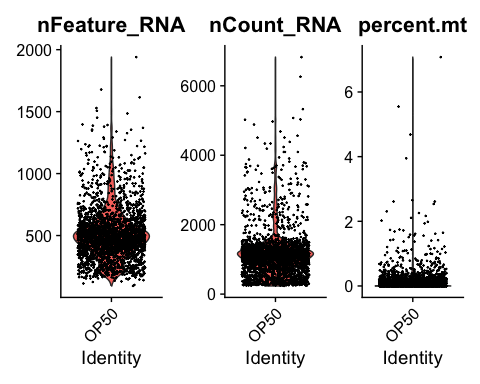


# Setting the “percent.mt” variable of the Seurat object
OP50 <- subset(OP50, subset = nFeature_RNA > 100 & nFeature_RNA < 1600 & nCount_RNA > 100 & nCount_RNA < 4000 & percent.mt < 5)
#Subset to the "nonempty" cells from the DropletUtils analysis
OP50 <- subset(OP50, cells = op50_nonempty)
# Visualize QC metrics as a violin plot post-QC
VlnPlot(OP50, features = c("nFeature_RNA", "nCount_RNA", "percent.mt"), ncol = 3)

Warning: Default search for "data" layer in "RNA" assay yielded no results;
utilizing "counts" layer instead.


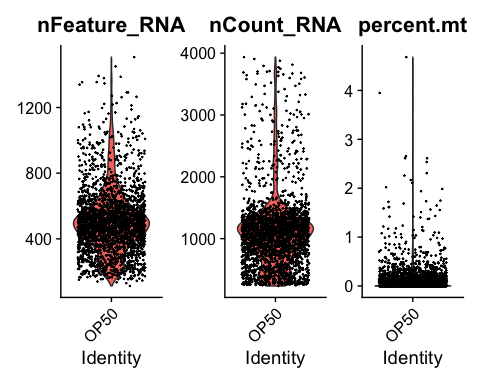


# PA14 Data (WT_Q_cells_2_PA14_14h)

PA14.data <- Read10X(data.dir = "cellranger_output/2024_07_22_data/count/PA14/outs/filtered_feature_bc_matrix")

PA14 <- CreateSeuratObject(counts = PA14.data, project = "PA14")

Warning: Feature names cannot have underscores ('_'), replacing with dashes
('-')

PA14

An object of class Seurat
22113 features across 2775 samples within 1 assay
Active assay: RNA (22113 features, 0 variable features)
 1 layer present: counts

PA14 <- NormalizeData(PA14)

Normalizing layer: counts

PA14 <- FindVariableFeatures(PA14)

Finding variable features for layer counts

PA14_features <- rownames(PA14)
PA14 <- ScaleData(PA14, features = PA14_features)

Centering and scaling data matrix

PA14 <- RunPCA(PA14)

PC_ 1
Positive: C27A2.8, mks-2, ZK616.1, F58B4.2, T04C12.9, arcp-1, flp-14, dylt-2, gcy-33, ptb-1
 egl-21, cng-1, cab-1, che-2, flp-21, sri-48, dyf-7, ZC449.5, R01H2.8, pde-1
 mks-3, flp-5, Y102A11A.9, bbs-5, F38B6.6, gnrr-2, mks-1, Y45G5AM.6, mam-8, msa-1
Negative: rpl-12, rpl-17, eef-1A.1, eef-2, rbm-3.2, rps-1, rpl-3, rpl-20, rps-8, rpl-1
 rpl-18, rpl-5, rps-0, rla-0, rps-7, rpl-7, rps-23, rpl-31, rpl-16, rpl-6
 rpl-35, rpl-23, rps-9, rps-24, rpl-2, rps-13, rpl-13, W01D2.1, rpl-21, rpl-33
PC_ 2
Positive: sams-1, nlp-29, pmt-2, nlp-24, col-10, grd-13, ttr-15, ram-2, upp-1, R09E12.9
 grd-3, T01C8.2, T25G12.3, gst-20, nlp-33, col-42, lips-10, K07E3.4, F17H10.2, gcy-33
 arcp-1, zipt-16, flp-14, cab-1, col-125, msa-1, fasn-1, fbxa-24, F58B4.2, cth-1
Negative: hil-2, mec-7, calu-1, his-32, hil-3, F33A8.7, hlh-14, F07C6.4, rpl-12, myrf-1
 ctf-8, C08F1.10, rbm-3.2, Y54H5A.2, nid-1, T05H4.11, kcc-1, thk-1, R07E3.7, F39B2.3
 C09B8.5, rhgf-2, C43D7.8, lipl-7, Y37E3.30, rpa-1, tsp-7, pkd-2, F36G3.1, cam-1
PC_ 3
Positive: nex-4, pde-1, T05A8.3, nlp-43, ins-18, WBGene00044308, snt-4, dgk-5, flp-21, F32B4.5
 aexr-2, F58F9.1, C08G9.1, gcy-33, hil-3, plc-1, glb-5, F49E10.4, glb-1, K02F2.5
 nlp-47, F38E9.6, Y45G5AM.6, T06G6.3, Y46D2A.1, lact-1, ost-1, hil-2, glna-3, C17H12.10
Negative: T01D3.3, dma-1, F58E2.5, gst-7, R01H2.8, M162.5, fmil-1, daf-10, tag-275, C04A11.2
 mam-8, ssq-1, rhgf-2, C27A2.8, mec-8, vab-8, che-2, pkd-2, dylt-2, lpr-6
 hyl-1, msa-1, F38B6.6, mks-1, mak-1, osm-12, hbl-1, tbc-18, F21D12.3, lin-32
PC_ 4
Positive: hlh-14, mec-7, F33A8.7, nid-1, unc-68, F36G3.1, tsp-7, C08F1.10, C15C8.5, lbp-3
 mec-3, myrf-1, WBGene00050903, C02B10.3, K04C2.3, WBGene00021130, C24H12.1, F39B2.3, nlp-29, sams-1
 Y17G9B.11, col-10, cam-1, ram-2, C09B8.5, pmt-2, his-32, nlp-33, cth-1, grd-3
Negative: lin-32, mig-21, che-2, R05H11.2, msa-1, pkd-2, Y54H5A.2, suro-1, cpn-1, lipl-7
 gst-7, C04A11.2, fbxb-74, ssq-1, R01H2.8, vab-8, C43D7.8, WBGene00022730, rps-26, srd-30
 emb-9, lgc-27, mam-8, rps-9, T01D3.3, rps-7, srd-29, dylt-2, rpl-3, rps-1
PC_ 5
Positive: srd-30, pkd-2, ssq-1, srd-29, flp-5, lipl-7, E04F6.10, Y54H5A.2, gst-7, cam-1
 lgc-27, magi-1, F07C6.4, mam-8, nlp-43, glna-3, plc-1, T05A8.3, col-114, C43D7.8
 lin-32, rhgf-2, WBGene00017849, dgk-5, C05E11.6, nlp-47, C08G9.1, WBGene00017202, F58F9.1, WBGene00044308
Negative: flp-4, mab-5, cutl-10, cyk-7, M162.5, npr-9, dct-14, C33A12.4, zig-8, flp-9
 sto-5, test-1, hyl-1, clc-4, ZC449.5, tiam-1, gnrr-2, far-2, F21D12.3, exp-2
 flp-27, F58B4.2, F58E2.5, T10B10.4, atln-2, ZK616.1, ncam-1, sto-1, K02B12.9, pqn-88

ElbowPlot(PA14, ndims = 50)


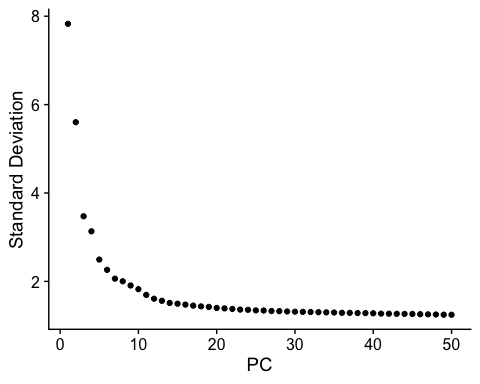


PA14 <- FindNeighbors(PA14, dims = 1:15, reduction = "pca")

Computing nearest neighbor graph

Computing SNN

PA14 <- FindClusters(PA14, resolution = 1.0, cluster.name = "PA14.unintegrated_clusters")

Modularity Optimizer version 1.3.0 by Ludo Waltman and Nees Jan van Eck

Number of nodes: 2775
Number of edges: 100085

Running Louvain algorithm...
Maximum modularity in 10 random starts: 0.8484
Number of communities: 13
Elapsed time: 0 seconds

PA14 <- RunUMAP(PA14, dims = 1:15, reduction = "pca", reduction.name = "umap.PA14.unintegrated")

11:05:28 UMAP embedding parameters a = 0.9922 b = 1.112

11:05:28 Read 2775 rows and found 15 numeric columns

11:05:28 Using Annoy for neighbor search, n_neighbors = 30

11:05:28 Building Annoy index with metric = cosine, n_trees = 50

0% 10 20 30 40 50 60 70 80 90 100%

[----|----|----|----|----|----|----|----|----|----|

**************************************************|
11:05:28 Writing NN index file to temp file /var/folders/2_/0b7d0hy11bd2g2nl32tfghfh981cxm/T//RtmpZVhw0e/file18f314689088
11:05:28 Searching Annoy index using 1 thread, search_k = 3000
11:05:28 Annoy recall = 100%
11:05:29 Commencing smooth kNN distance calibration using 1 thread with target n_neighbors = 30
11:05:30 Initializing from normalized Laplacian + noise (using RSpectra)
11:05:30 Commencing optimization for 500 epochs, with 111834 positive edges
11:05:30 Using rng type: pcg
11:05:34 Optimization finished

PA14_clusters <- as.character(PA14$seurat_clusters)
names(PA14_clusters) <- as.character(names(PA14$seurat_clusters))

## DropletUtils

PA14_mol_info <- read10xMolInfo("cellranger_output/2024_07_22_data/count/PA14/outs/molecule_info.h5")

### Downsampling

set.seed(100)
PA14_withsample <- downsampleReads("cellranger_output/2024_07_22_data/count/PA14/outs/molecule_info.h5", prop = 0.5)
sum(PA14_withsample)

[1] 4695698

pa_out <- barcodeRanks(PA14_withsample)
plot(pa_out$rank, pa_out$total, log="xy", xlab="Rank", ylab="Total")

Warning in xy.coords(x, y, xlabel, ylabel, log): 15056 y values <= 0 omitted
from logarithmic plot

o.PA14 <- order(pa_out$rank)
lines(pa_out$rank[o.PA14], pa_out$fitted[o.PA14], col="red")

abline(h=metadata(pa_out)$knee, col="dodgerblue", lty=2)
abline(h=metadata(pa_out)$inflection, col="forestgreen", lty=2)
legend("bottomleft", lty=2, col=c("dodgerblue", "forestgreen"),
 legend=c("knee", "inflection"))


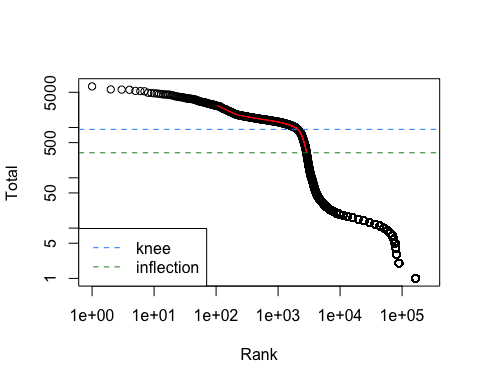


PA14_empty <- emptyDrops(PA14_withsample, lower = 50)

PA14_isCell <- PA14_empty$FDR <= 0.01

table(Limited=PA14_empty$Limited, Significant = PA14_isCell)

Significant
Limited FALSE TRUE
 FALSE 730 86
 TRUE 0 3275

plot(PA14_empty$Total, -PA14_empty$LogProb, col=ifelse(PA14_isCell, "red", "black"),
 xlab="Total UMI count", ylab="-Log Probability")


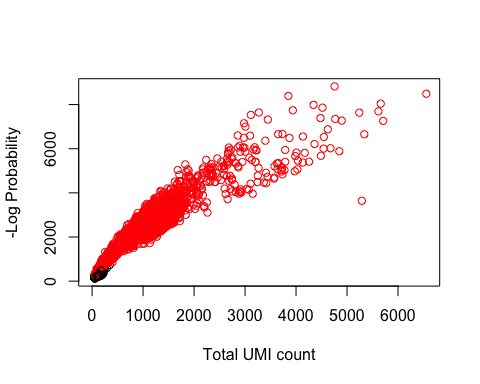


PA14_empty %>%
 as.data.frame() %>%
 rownames_to_column(., var = "barcode") %>%
 filter(., FDR <= 0.01) %>%
 dplyr::select(., barcode) %>%
 as_vector() %>%
 unname() -> PA14_nonempty

## SoupX

PA14_toc <- Seurat::Read10X("cellranger_output/2024_07_22_data/count/PA14/outs/filtered_feature_bc_matrix")
PA14_tod <- Seurat::Read10X("cellranger_output/2024_07_22_data/count/PA14/outs/raw_feature_bc_matrix/")
PA14_sc <- SoupChannel(PA14_tod, PA14_toc, calcSoupProfile = FALSE)
PA14_sc = estimateSoup(PA14_sc, soupRange = c(0, 25))

PA14_sc <- setClusters(PA14_sc, PA14_clusters)

PA14_sc <- autoEstCont(PA14_sc)

419 genes passed tf-idf cut-off and 278 soup quantile filter. Taking the top 100.

Using 996 independent estimates of rho.

Estimated global rho of 0.01


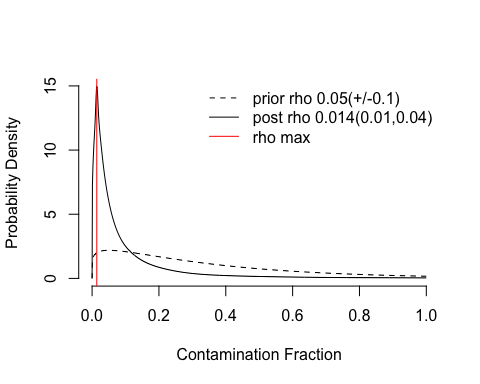


PA14_out <- adjustCounts(PA14_sc)

Warning in sparseMatrix(i = out@i[w] + 1, j = out@j[w] + 1, x = out@x[w], :
'giveCsparse' is deprecated; setting repr="T" for you

Expanding counts from 13 clusters to 2775 cells.

cntSoggy = rowSums(PA14_sc$toc > 0)
cntStrained = rowSums(PA14_out > 0)
mostZeroed = tail(sort((cntSoggy - cntStrained)/cntSoggy), n = 10)
mostZeroed

nlp-29 col-103 col-98 col-117 F46F2.3 col-3 snet-1
0.05128205 0.05500000 0.08648649 0.09090909 0.09574468 0.10869565 0.13636364
 grd-10 col-107 ram-2
0.15950920 0.57884232 0.66569767

tail(sort(rowSums(PA14_sc$toc > PA14_out)/rowSums(PA14_sc$toc > 0)), n = 20)

F54H12.10 E02H9.13 WBGene00306000 W04B5.8 Y39A3B.8
 1 1 1 1 1
 Y54F10BM.21 Y54F10BM.22 WBGene00306069 ctb-1 nduo-6
 1 1 1 1 1
WBGene00010958 WBGene00010959 atp-6 nduo-2 ctc-3
 1 1 1 1 1
 nduo-4 ctc-1 ctc-2 nduo-3 nduo-5
 1 1 1 1 1

DropletUtils::write10xCounts("output/all_fed_dataset/postprocess/PA14", PA14_out)

## Creating Seurat object and initial QC

PA14.data <- Read10X(data.dir = "output/all_fed_dataset/postprocess/PA14/")

PA14 <- CreateSeuratObject(counts = PA14.data, project = "PA14", min.cells = 3, min.features = 100)

Warning: Feature names cannot have underscores ('_'), replacing with dashes
('-')

left_join(
 read_delim("mt_gene_list.tsv", delim = "\t", col_names = "gene"),
 read_delim("output/all_fed_dataset/postprocess/PA14/genes.tsv", delim = "\t",
 col_names = c("wb", "gene", "desc")),
 by = c("gene" = "wb")) %>%
 filter(., !is.na(gene.y)) %>%
 dplyr::select(., gene.y) %>%
 as_vector() %>%
 unname() -> mt_genes

Rows: 36 Columns: 1
── Column specification ────────────────────────────────────────────────────────
Delimiter: "\t"
chr (1): gene

ℹ Use `spec()` to retrieve the full column specification for this data.
ℹ Specify the column types or set `show_col_types = FALSE` to quiet this message.
Rows: 22113 Columns: 2
── Column specification ────────────────────────────────────────────────────────
Delimiter: "\t"
chr (2): wb, gene

ℹ Use `spec()` to retrieve the full column specification for this data.
ℹ Specify the column types or set `show_col_types = FALSE` to quiet this message.

PA14[["percent.mt"]]<- PercentageFeatureSet(PA14, features = mt_genes)

VlnPlot(PA14, features = c("nFeature_RNA", "nCount_RNA", "percent.mt"), ncol = 3)

Warning: Default search for "data" layer in "RNA" assay yielded no results;
utilizing "counts" layer instead.


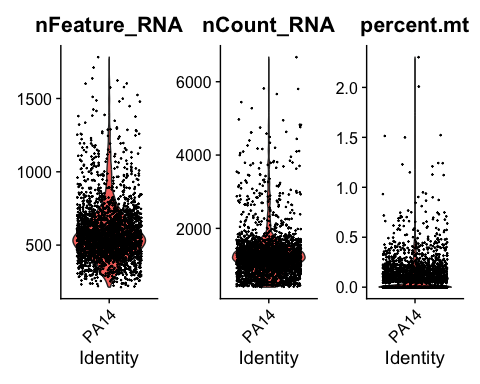


PA14 <- subset(PA14, subset = nFeature_RNA > 100 & nFeature_RNA < 1600 & nCount_RNA > 100 & nCount_RNA < 4000 & percent.mt < 5)
PA14 <- subset(PA14, cells = PA14_nonempty)

VlnPlot(PA14, features = c("nFeature_RNA", "nCount_RNA", "percent.mt"), ncol = 3)

Warning: Default search for "data" layer in "RNA" assay yielded no results;
utilizing "counts" layer instead.


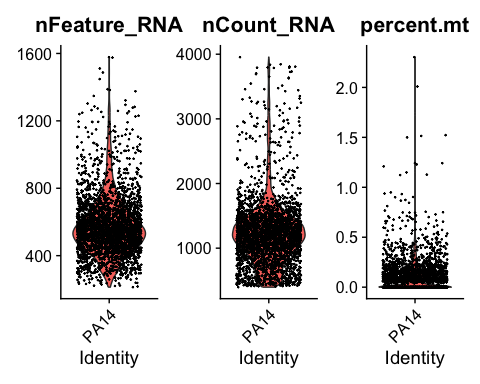


# OP50.2 Data (WT_Q_cells_3_OP50_12-16h)

OP50.2.data <- Read10X(data.dir = "cellranger_output/2025_03_07_data/count/LE4112/outs/filtered_feature_bc_matrix/")

OP50.2 <- CreateSeuratObject(counts = OP50.2.data, project = "OP50.2")

Warning: Feature names cannot have underscores ('_'), replacing with dashes
('-')

OP50.2

An object of class Seurat
22115 features across 8861 samples within 1 assay
Active assay: RNA (22115 features, 0 variable features)
 1 layer present: counts

OP50.2 <- NormalizeData(OP50.2)

Normalizing layer: counts

OP50.2 <- FindVariableFeatures(OP50.2)

Finding variable features for layer counts

OP50.2_features <- rownames(OP50.2)
OP50.2 <- ScaleData(OP50.2, features = OP50.2_features)

Centering and scaling data matrix

OP50.2 <- RunPCA(OP50.2)

PC_ 1
Positive: Y54E2A.8, T10B11.8, glh-1, ztf-1, pid-1, B0304.4, C46A5.6, iff-1, rmh-1, B0001.2
 ani-2, clec-87, F30F8.1, K09H9.7, F54D5.2, C05C10.7, R148.4, C01G8.1, W07E6.5, hil-4
 K07A1.1, fbxa-95, immt-2, fbxa-108, oef-1, apc-10, T28D9.4, fem-3, fbxa-210, F45F2.11
Negative: lec-9, C17F4.7, Y119D3B.21, lec-10, C50F4.1, K06G5.1, ctsa-2, nspg-9, pcp-3, nspg-14
 R07E3.1, clec-50, nep-17, C18H9.6, F53C11.1, ctsa-4.2, C49C8.5, C29F3.7, F31F7.1, T24C4.4
 nspg-6, clec-17, Y51A2D.13, W05H9.1, pept-1, T01D3.6, T07H8.11, lys-1, xdh-1, elo-5
PC_ 2
Positive: cnc-8, K07E1.1, col-91, C35C5.9, nlp-33, col-160, col-147, fip-5, pah-1, col-94
 nlp-27, haly-1, T10H9.8, C16E9.1, cest-24, col-39, far-7, col-166, F42A8.1, srap-1
 nspe-5, T27D12.1, Y38E10A.28, F15E6.3, ttr-32, C39E9.8, F46C5.10, col-80, F18E3.13, ttr-15
Negative: ctsa-2, nep-17, pept-1, pcp-3, T01D3.6, R07E3.1, ctsa-4.2, C18H9.6, lec-9, F53C11.1
 K06G5.1, Y51A2D.13, C29F3.7, W05H9.1, elo-5, Y119D3B.21, asp-6, lys-1, xdh-1, ads-1
 clec-50, cpr-6, C50F4.1, ugt-18, pgrn-1, ifc-1, folt-2, dod-17, clec-17, ugt-22
PC_ 3
Positive: ctsa-2, hgo-1, hpd-1, pept-1, dhs-21, nep-17, K07E1.1, T01D3.6, col-91, cnc-8
 oatr-1, F31F7.1, glna-2, cth-2, T10H9.8, C35C5.9, ctsa-4.2, pcp-3, fip-5, haly-1
 F15E6.3, Y38E10A.28, xdh-1, far-7, C16E9.1, ttr-32, ttr-31, ugt-46, nspe-5, C18H9.6
Negative: C27A2.8, mks-2, gcy-37, Y44A6D.2, ifta-2, F58B4.2, mec-12, gcy-34, mam-8, mlc-3
 C33A12.4, Y17D7B.10, cng-1, sri-48, flp-5, gcy-33, flp-14, ssq-1, ttn-1, pde-1
 far-1, mlc-2, K09F6.13, T03F1.11, flp-21, rbm-3.2, fip-2, Y45G5AM.6, C24A3.2, fipr-1
PC_ 4
Positive: rbm-3.2, C27A2.8, ram-2, mks-2, gcy-37, ifta-2, col-107, F58B4.2, mec-12, gcy-34
 mam-8, C17F4.7, Y44A6D.2, C33A12.4, grd-10, sri-48, Y17D7B.10, flp-14, ssq-1, grd-3
 gcy-33, flp-5, cng-1, grd-13, egl-21, pcbd-1, C08E3.1, flp-21, T25G12.3, F32H5.3
Negative: cpn-3, clik-1, B0379.1, ost-1, pqn-48, ttr-16, Y53H1B.2, let-2, mup-2, D1086.5
 pat-10, Y71H2B.4, unc-87, tnt-2, ttn-1, EGAP4.1, lev-11, R13H4.2, T10G3.1, pqn-24
 Y37D8A.2, T04A6.1, mlc-1, mlc-2, lbp-2, Y82E9BR.6, Y45F10B.13, cpna-2, anmt-3, F40A3.6
PC_ 5
Positive: Y32F6A.5, mct-4, ctsa-4.1, ugt-18, ctsa-1.2, K10D11.3, clec-166, ceh-37, acox-1.5, clec-41
 ugt-44, folt-2, asah-1, crn-6, clec-80, F01D5.1, gpn-1, cpr-1, F01D5.5, F49F1.5
 F01D5.3, C14C6.5, skr-5, pho-1, clec-67, ent-4, F32H5.1, lys-8, pho-4, D1054.18
Negative: T25C12.3, irg-7, pbo-4, gfi-1, hpo-6, PDB1.1, C17F4.7, B0035.13, C34H4.2, nspg-7.1
 nspg-10, T15B7.1, C08E3.1, C08E3.13, C50B6.7, C49C8.5, nspg-14, srw-86, pho-11, nspg-9
 dod-20, scl-2, Y14H12A.2, Y105C5A.12, nspg-6, F55G1.15, K06G5.1, Y39B6A.1, R09H10.5, C17H12.6

ElbowPlot(OP50.2, ndims = 50)


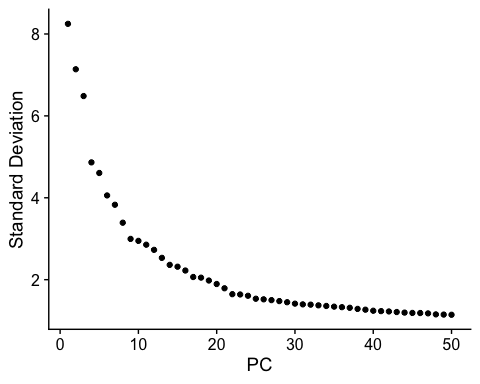


OP50.2 <- FindNeighbors(OP50.2, dims = 1:22, reduction = "pca")

Computing nearest neighbor graph

Computing SNN

OP50.2 <- FindClusters(OP50.2, resolution = 1.0, cluster.name = "OP50.2.unintegrated_clusters")

Modularity Optimizer version 1.3.0 by Ludo Waltman and Nees Jan van Eck

Number of nodes: 8861
Number of edges: 330783

Running Louvain algorithm...
Maximum modularity in 10 random starts: 0.8970
Number of communities: 25
Elapsed time: 0 seconds

OP50.2 <- RunUMAP(OP50.2, dims = 1:22, reduction = "pca", reduction.name = "umap.OP50.2.unintegrated")

11:06:48 UMAP embedding parameters a = 0.9922 b = 1.112

11:06:48 Read 8861 rows and found 22 numeric columns

11:06:48 Using Annoy for neighbor search, n_neighbors = 30

11:06:48 Building Annoy index with metric = cosine, n_trees = 50

0% 10 20 30 40 50 60 70 80 90 100%

[----|----|----|----|----|----|----|----|----|----|

**************************************************|
11:06:49 Writing NN index file to temp file /var/folders/2_/0b7d0hy11bd2g2nl32tfghfh981cxm/T//RtmpZVhw0e/file18f34fb108ce
11:06:49 Searching Annoy index using 1 thread, search_k = 3000
11:06:51 Annoy recall = 100%
11:06:52 Commencing smooth kNN distance calibration using 1 thread with target n_neighbors = 30
11:06:53 Initializing from normalized Laplacian + noise (using RSpectra)
11:06:54 Commencing optimization for 500 epochs, with 405084 positive edges
11:06:54 Using rng type: pcg
11:07:05 Optimization finished

OP50.2_clusters <- as.character(OP50.2$seurat_clusters)
names(OP50.2_clusters) <- as.character(names(OP50.2$seurat_clusters))

## DropletUtils

Loading the molecule info files

OP50.2_mol_info <- read10xMolInfo("cellranger_output/2025_03_07_data/count/LE4112/outs/molecule_info.h5")

### Downsampling

set.seed(100)
OP50.2_withsample <- downsampleReads("cellranger_output/2025_03_07_data/count/LE4112/outs/molecule_info.h5", prop = 0.5)
sum(OP50.2_withsample)

[1] 29678995

op_out.2 <- barcodeRanks(OP50.2_withsample)
plot(op_out.2$rank, op_out.2$total, log="xy", xlab="Rank", ylab="Total")

Warning in xy.coords(x, y, xlabel, ylabel, log): 67271 y values <= 0 omitted
from logarithmic plot

o.2 <- order(op_out.2$rank)
lines(op_out.2$rank[o.2], op_out.2$fitted[o.2], col="red")

abline(h=metadata(op_out.2)$knee, col="dodgerblue", lty=2)
abline(h=metadata(op_out.2)$inflection, col="forestgreen", lty=2)
legend("bottomleft", lty=2, col=c("dodgerblue", "forestgreen"),
 legend=c("knee", "inflection"))


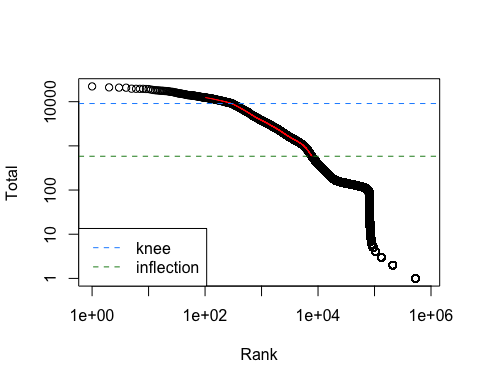


OP50.2_empty <- emptyDrops(OP50.2_withsample, lower = 50)

OP50.2_isCell <- OP50.2_empty$FDR <= 0.01

table(Limited=OP50.2_empty$Limited, Significant = OP50.2_isCell)

Significant
Limited FALSE TRUE
 FALSE 26724 45230
 TRUE 0 9338

plot(OP50.2_empty$Total, -OP50.2_empty$LogProb, col=ifelse(OP50.2_isCell, "red", "black"),
 xlab="Total UMI count", ylab="-Log Probability")


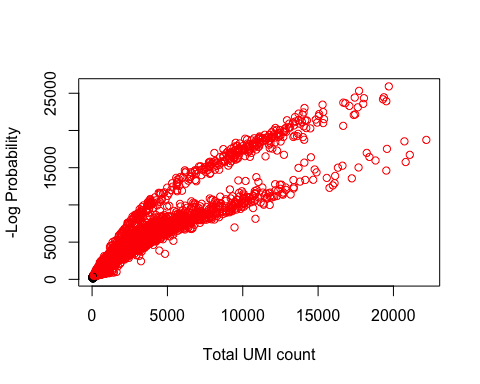


OP50.2_empty %>%
 as.data.frame() %>%
 rownames_to_column(., var = "barcode") %>%
 filter(., FDR <= 0.01) %>%
 dplyr::select(., barcode) %>%
 as_vector() %>%
 unname() -> OP50.2_nonempty

## SoupX

OP50.2_toc <- Seurat::Read10X("cellranger_output/2025_03_07_data/count/LE4112/outs/filtered_feature_bc_matrix")
OP50.2_tod <- Seurat::Read10X("cellranger_output/2025_03_07_data/count/LE4112/outs/raw_feature_bc_matrix")
OP50.2_sc <- SoupChannel(OP50.2_tod, OP50.2_toc, calcSoupProfile = FALSE)
OP50.2_sc = estimateSoup(OP50.2_sc, soupRange = c(0, 25))

OP50.2_sc <- setClusters(OP50.2_sc, OP50.2_clusters)

OP50.2_sc <- autoEstCont(OP50.2_sc)

6569 genes passed tf-idf cut-off and 1639 soup quantile filter. Taking the top 100.

Using 1971 independent estimates of rho.

Estimated global rho of 0.02


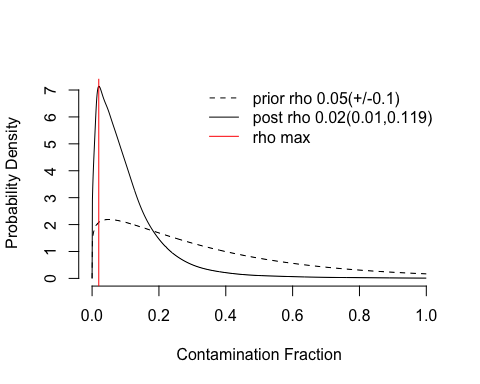


OP50.2_out <- adjustCounts(OP50.2_sc)

Warning in sparseMatrix(i = out@i[w] + 1, j = out@j[w] + 1, x = out@x[w], :
'giveCsparse' is deprecated; setting repr="T" for you

Expanding counts from 25 clusters to 8861 cells.

cntSoggy = rowSums(OP50.2_sc$toc > 0)
cntStrained = rowSums(OP50.2_out > 0)
mostZeroed = tail(sort((cntSoggy - cntStrained)/cntSoggy), n = 10)
mostZeroed

cnc-11 sip-1 Y53H1B.2 hil-4 C49F5.7 ttr-16 haly-1
0.06043956 0.06858974 0.06956522 0.07954545 0.08301344 0.08764940 0.11570248
 H34I24.2 flp-5 snet-1
0.13684211 0.14017094 0.41379310

tail(sort(rowSums(OP50.2_sc$toc > OP50.2_out)/rowSums(OP50.2_sc$toc > 0)), n = 20)

T24C4.10 W04B5.8 Y39A3B.8 Y54F10BM.21 Y54F10BM.22
 1 1 1 1 1
WBGene00306069 ctb-1 nduo-6 WBGene00010958 WBGene00010959
 1 1 1 1 1
 atp-6 nduo-2 ctc-3 nduo-4 ctc-1
 1 1 1 1 1
 ctc-2 nduo-3 nduo-5 mCherry GFP
 1 1 1 1 1

DropletUtils::write10xCounts("output/all_fed_dataset/postprocess/OP50.2", OP50.2_out)

## Creating Seurat object and initial QC

OP50.2.data <- Read10X(data.dir = "output/all_fed_dataset/postprocess/OP50.2/")

OP50.2 <- CreateSeuratObject(counts = OP50.2.data, project = "OP50.2", min.cells = 3, min.features = 100)

Warning: Feature names cannot have underscores ('_'), replacing with dashes
('-')

left_join(
 read_delim("mt_gene_list.tsv", delim = "\t", col_names = "gene"),
 read_delim("output/all_fed_dataset/postprocess/OP50.2/genes.tsv", delim = "\t",
 col_names = c("wb", "gene", "desc")),
 by = c("gene" = "wb")) %>%
 filter(., !is.na(gene.y)) %>%
 dplyr::select(., gene.y) %>%
 as_vector() %>%
 unname() -> mt_genes

Rows: 36 Columns: 1
── Column specification ────────────────────────────────────────────────────────
Delimiter: "\t"
chr (1): gene

ℹ Use `spec()` to retrieve the full column specification for this data.
ℹ Specify the column types or set `show_col_types = FALSE` to quiet this message.
Rows: 22115 Columns: 2
── Column specification ────────────────────────────────────────────────────────
Delimiter: "\t"
chr (2): wb, gene

ℹ Use `spec()` to retrieve the full column specification for this data.
ℹ Specify the column types or set `show_col_types = FALSE` to quiet this message.

OP50.2[["percent.mt"]]<- PercentageFeatureSet(OP50.2, features = mt_genes)

VlnPlot(OP50.2, features = c("nFeature_RNA", "nCount_RNA", "percent.mt"), ncol = 3)

Warning: Default search for "data" layer in "RNA" assay yielded no results;
utilizing "counts" layer instead.


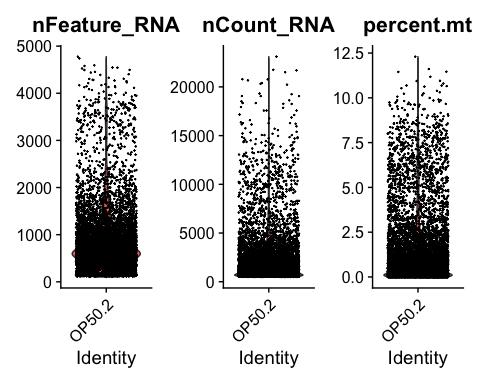


OP50.2 <- subset(OP50.2, subset = nFeature_RNA > 100 & nFeature_RNA < 1600 & nCount_RNA > 100 & nCount_RNA < 4000 & percent.mt < 5)
OP50.2 <- subset(OP50.2, cells = OP50.2_nonempty)

VlnPlot(OP50.2, features = c("nFeature_RNA", "nCount_RNA", "percent.mt"), ncol = 3)

Warning: Default search for "data" layer in "RNA" assay yielded no results;
utilizing "counts" layer instead.


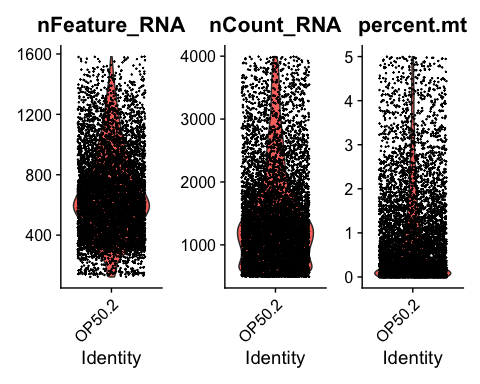


# -

# Processing individual experiments

## OP50 (WT_Q_cells_1_OP50_14h)

OP50 <- NormalizeData(OP50)

Normalizing layer: counts

OP50 <- FindVariableFeatures(OP50)

Finding variable features for layer counts

cele_features <- rownames(OP50)
OP50 <- ScaleData(OP50, features = cele_features)

Centering and scaling data matrix

OP50 <- RunPCA(OP50)

PC_ 1
Positive: C27A2.8, gcy-32, gcy-37, mks-2, pdl-1, arl-3, F58B4.2, dyf-6, F49H12.4, ZK616.1
 Y44A6D.2, F49E12.8, sbt-1, T04C12.9, arcp-1, WBGene00014307, T01D3.1, gcy-34, ifta-2, gcy-33
 guk-1, flp-14, Y17D7B.10, ptb-1, T10B5.4, che-2, M01H9.4, dyf-18, cng-1, gcy-25
Negative: rpl-12, rpl-7A, rpl-17, eef-1A.1, rpl-32, rbm-3.2, eef-2, rps-18, eef-1B.1, rps-1
 rpl-36, rps-8, rpl-5, eef-1G, rpl-20, rps-4, rps-5, rps-14, rpl-18, rpl-3
 rps-7, rpl-10, rps-23, rpl-15, rpl-1, rpl-41.2, rpl-33, rps-9, rpl-7, rpl-2
PC_ 2
Positive: mlc-3, cpn-3, ost-1, unc-15, EGAP4.1, let-2, lev-11, pat-10, sodh-1, mlc-2
 ttn-1, T21B6.3, pqn-48, clik-1, alh-8, cpl-1, cpz-1, Y71H2B.4, mlc-1, ttr-16
 mup-2, tnt-2, pck-1, csq-1, Y53H1B.2, T10G3.1, zyx-1, B0379.1, T04A6.1, spp-15
Negative: hil-2, his-24, calu-1, C50F4.6, rbm-3.2, mec-7, C09D4.2, F32H5.3, his-32, hil-3
 Y54H5A.2, cyd-1, hlh-14, F07C6.4, F41C3.7, ztf-11, F33A8.7, hphd-1, tbca-1, Y47A7.2
 tost-1, pkd-2, tbx-2, R05H5.3, ctf-8, smp-1, rpl-12, egl-46, Y47G6A.31, myrf-1
PC_ 3
Positive: sams-1, col-3, col-98, col-117, col-107, nlp-29, col-94, ram-2, col-103, nlp-24
 col-10, F46F2.3, grd-10, ttr-15, gst-20, F26G1.2, pmt-2, R09E12.9, cnc-4, lips-10
 nlp-33, col-42, upp-1, grd-13, grd-3, F59E11.7, W04H10.6, T01C8.2, nhr-270, col-125
Negative: pqn-48, Y71H2B.4, T21B6.3, pat-10, clik-1, mlc-2, csq-1, tnt-2, cpn-3, ttr-16
 cpz-1, Y53H1B.2, unc-15, mlc-3, T04A6.1, T10G3.1, cpl-1, let-2, zyx-1, spp-15
 lev-11, mlc-1, mup-2, ttn-1, unc-22, Y105C5B.5, D1086.5, Y17D7B.4, Y4C6B.7, unc-87
PC_ 4
Positive: dma-1, egl-46, T01D3.3, K09F6.13, C03C10.5, ram-2, F23B12.4, WBGene00023302, sams-1, col-117
 col-98, F44E5.1, daf-19, gst-7, nlp-29, grd-10, F58E2.5, col-94, col-107, ssq-1
 cnc-4, mam-8, fmil-1, gst-20, gasr-8, C08F1.10, col-10, pmt-2, grd-3, ccep-290
Negative: F46H5.3, pde-1, mig-21, flp-21, nex-4, Y47A7.2, cpn-1, ins-18, gcy-33, cyd-1
 nlp-43, snt-4, mcm-7, R05H11.2, pcn-1, mcm-5, T05A8.3, R05H5.3, F32H5.3, suro-1
 ctf-8, Y17D7B.10, WBGene00044308, mcm-6, smo-1, pgal-1, mcm-2, thk-1, C10C5.7, nlp-47
PC_ 5
Positive: unc-54, lin-32, che-2, Y7A9D.1, gst-7, ham-1, pkd-2, W07A12.4, mig-21, msa-1
 daf-19, WBGene00023302, F23B12.4, R05H11.2, tbx-2, T01D3.3, mam-8, ssq-1, Y54H5A.2, C03C10.5
 dma-1, K09F6.13, lipl-7, arl-3, suro-1, ccep-290, egl-17, cpn-1, gasr-8, dsh-2
Negative: mec-7, hlh-14, F33A8.7, his-24, C09D4.2, nid-1, unc-68, C01C4.3, sem-4, F36G3.1
 WBGene00018813, C06E7.2, tbca-1, hil-3, myrf-1, arrd-25, C08F1.10, tsp-7, mig-1, WBGene00044308
 Y47G6A.31, C40A11.6, F16F9.1, mec-3, C15C8.5, lbp-3, nlp-47, far-1, F58F9.1, snt-4

ElbowPlot(OP50, ndims = 50)


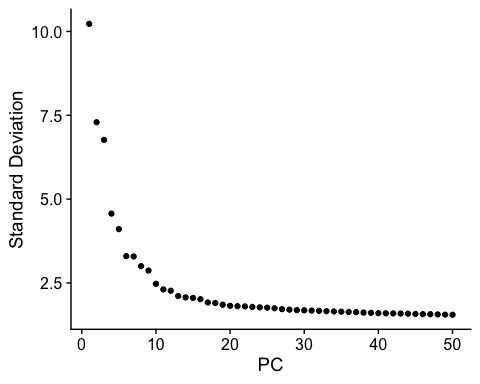


OP50 <- FindNeighbors(OP50, dims = 1:13, reduction = "pca")

Computing nearest neighbor graph

Computing SNN

OP50 <- FindClusters(OP50, resolution = 1.0, cluster.name = "OP50.unintegrated_clusters")

Modularity Optimizer version 1.3.0 by Ludo Waltman and Nees Jan van Eck

Number of nodes: 2502
Number of edges: 78261

Running Louvain algorithm...
Maximum modularity in 10 random starts: 0.8611
Number of communities: 17
Elapsed time: 0 seconds

OP50 <- RunUMAP(OP50, dims = 1:13, reduction = "pca", reduction.name = "umap.OP50.unintegrated")

11:09:40 UMAP embedding parameters a = 0.9922 b = 1.112

11:09:40 Read 2502 rows and found 13 numeric columns

11:09:40 Using Annoy for neighbor search, n_neighbors = 30

11:09:40 Building Annoy index with metric = cosine, n_trees = 50

0% 10 20 30 40 50 60 70 80 90 100%

[----|----|----|----|----|----|----|----|----|----|

**************************************************|
11:09:40 Writing NN index file to temp file /var/folders/2_/0b7d0hy11bd2g2nl32tfghfh981cxm/T//RtmpZVhw0e/file18f37e9962bc
11:09:40 Searching Annoy index using 1 thread, search_k = 3000
11:09:41 Annoy recall = 100%
11:09:42 Commencing smooth kNN distance calibration using 1 thread with target n_neighbors = 30
11:09:43 Initializing from normalized Laplacian + noise (using RSpectra)
11:09:44 Commencing optimization for 500 epochs, with 96084 positive edges
11:09:44 Using rng type: pcg
11:09:47 Optimization finished

DimPlot(OP50, reduction = "umap.OP50.unintegrated", label = T) + FeaturePlot(OP50, reduction = "umap.OP50.unintegrated",
 features = c("nCount_RNA"),
 label = F, order = T, cols = c("#efefef", "#4011ba"))


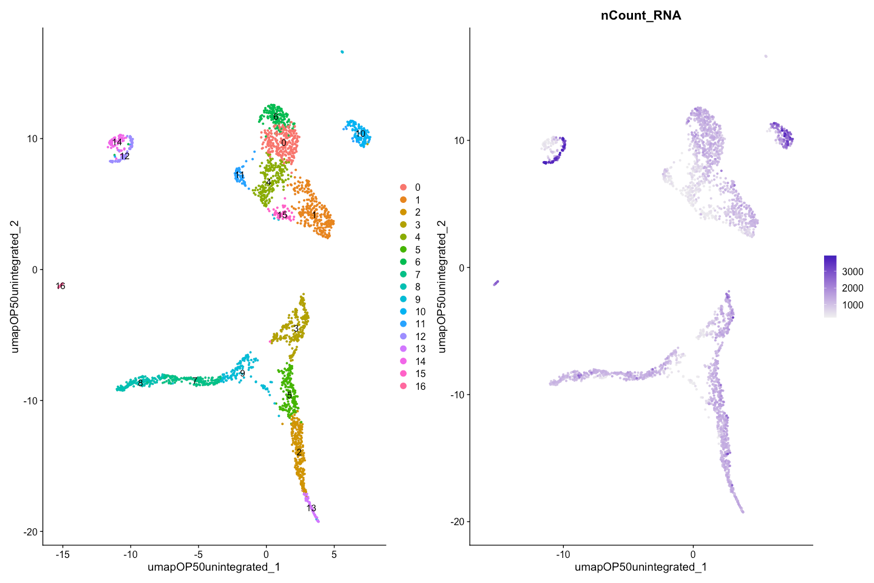


FeaturePlot(OP50, reduction = "umap.OP50.unintegrated",
 features = c("mig-21","mab-5", "lin-39", "gcy-32", "mec-7", "ajm-1"),
 label = F, order = T, cols = c("#efefef", "#4011ba"), ncol = 3)


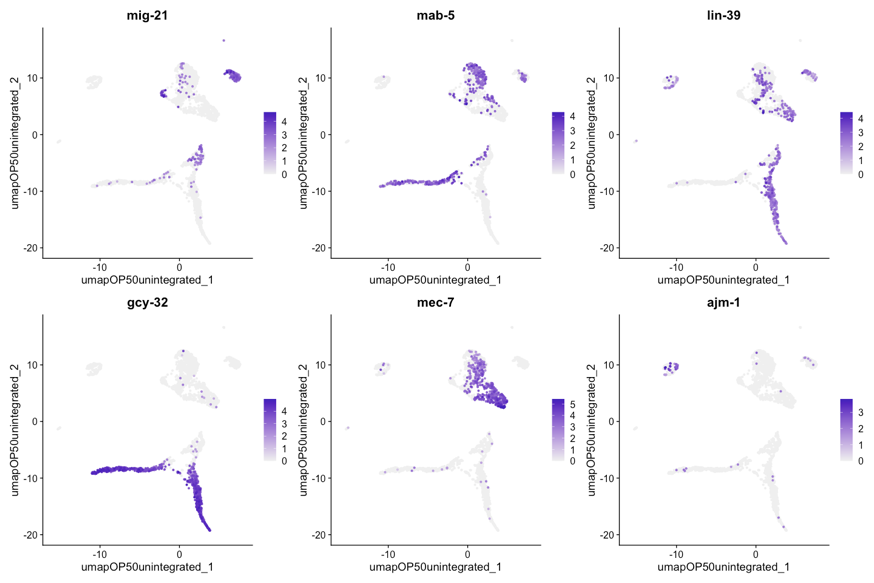


### 1st round of cleaning - removing non-Q cells

OP50.extra_cells_to_remove.OP50 <- WhichCells(OP50, idents = c("12", "14", "16"))
OP50_v2 <- subset(OP50, cells = setdiff(Cells(OP50), OP50.extra_cells_to_remove.OP50))

OP50_v2 <- NormalizeData(OP50_v2)

Normalizing layer: counts

OP50_v2 <- FindVariableFeatures(OP50_v2)

Finding variable features for layer counts

cele_features <- rownames(OP50_v2)
OP50_v2 <- ScaleData(OP50_v2, features = cele_features)

Centering and scaling data matrix

OP50_v2 <- RunPCA(OP50_v2)

PC_ 1
Positive: C27A2.8, gcy-32, gcy-37, mks-2, gcy-35, arl-3, F58B4.2, dyf-6, F49H12.4, ZK616.1
 F49E12.8, sbt-1, Y44A6D.2, T04C12.9, gcy-36, WBGene00014307, daf-25, arcp-1, tmem-231, T01D3.1
 K07C11.10, ceph-41, fmi-1, gcy-34, ifta-2, guk-1, gcy-33, dylt-2, flp-14, che-2
Negative: rpl-12, ctsa-1.1, rbm-3.2, rpl-7A, eef-1A.1, rpl-32, eef-2, rps-18, rpl-36, eef-1B.1
 rpl-41.2, rnr-1, eef-1G, rpl-5, rpl-18, rps-1, rpl-20, rps-5, rps-8, aldo-2
 rpl-2, rpl-43, cdk-1, rpl-10, rpl-3, his-24, rps-14, rps-23, rps-4, rps-9
PC_ 2
Positive: egl-46, hlh-14, mec-7, C08F1.10, F33A8.7, dma-1, nid-1, C40A11.6, ctsa-1.1, F44E5.1
 C09D4.2, rhgf-2, WBGene00023302, F39B2.3, cki-1, WBGene00018813, K09F6.13, C06E7.2, atf-2, C01C4.3
 F36G3.1, syd-9, smp-1, cank-26, tsp-7, unc-68, tbca-1, C03C10.5, R11G1.2, mec-3
Negative: cpn-1, mig-21, pde-1, flp-21, gcy-33, unc-54, lec-4, rps-6, suro-1, Y17D7B.10
 rpl-30, WBGene00022730, nex-4, rla-1, rpl-7, rpl-31, rps-13, rps-21, rps-8, flp-14
 rps-17, rla-0, ins-18, rpl-33, pcn-1, egl-17, rps-7, T25G12.3, rpl-11.2, W01D2.1
PC_ 3
Positive: lin-32, unc-54, gst-7, Y7A9D.1, che-2, F48E3.9, T01D3.3, ham-1, pkd-2, daf-19
 dma-1, WBGene00023302, K09F6.13, C03C10.5, mks-5, gcy-36, ssq-1, mam-8, fmi-1, R01H2.8
 msa-1, W07A12.4, ift-20, gasr-8, tmem-231, ccep-290, F58E2.5, syd-9, arl-3, dsh-2
Negative: his-24, mec-7, hlh-14, F33A8.7, pde-1, nex-4, WBGene00044308, snt-4, nlp-43, C09D4.2
 unc-68, flp-21, ins-18, hil-3, T05A8.3, nlp-47, F58F9.1, sem-4, aexr-2, nid-1
 gcy-33, hil-2, dgk-5, ampd-1, myrf-1, glb-5, srg-25, pgal-1, die-1, C01C4.3
PC_ 4
Positive: hil-2, F32H5.3, his-32, rbm-3.2, Y43F8B.2, hphd-1, his-60, pezo-1, ctsa-1.1, cyd-1
 cdk-1, myrf-1, mec-7, mab-5, M01H9.4, ZK742.7, ctf-8, rfc-4, Y43B11AR.1, tost-1
 C09D4.2, flp-4, Y47A7.2, zag-1, tbca-1, rnr-1, ztf-11, his-24, pmp-3, F33A8.7
Negative: WBGene00023209, Y39B6A.5, lbp-1, cpi-1, inos-1, dig-1, ccg-1, clec-178, cup-4, lgc-25
 C07G3.10, Y116A8C.3, lgc-26, T19C3.5, ttr-1, Y73F4A.1, lgc-28, K07E3.4, mig-6, B0393.9
 unc-122, hot-5, F56C4.4, Y54F10BM.12, F32E10.8, lgc-23, K02E7.11, fbxa-24, lbp-2, cof-2
PC_ 5
Positive: flp-4, ZK742.7, M162.5, mab-5, cutl-10, nlp-40, cyk-7, lat-1, npr-9, F35B3.7
 flp-9, glb-24, seb-3, H01M10.2, kvs-2, odr-4, test-1, nmr-1, grd-12, sto-5
 hyl-1, C33A12.4, cup-16, exp-2, wht-1, cle-1, C34D1.4, dct-14, ZK867.2, nlp-8
Negative: flp-5, E04F6.10, F44E5.1, mig-13, T05A8.3, nlp-43, plc-1, die-1, ssq-1, glna-3
 nlp-47, pkd-2, col-114, C24B5.1, C10C5.7, srg-25, dgk-5, mam-8, pdf-1, WBGene00044308
 magi-1, sre-4, F58F9.1, ric-4, Y45G5AM.6, syg-1, Y54H5A.2, srd-29, gst-7, F42A9.9

ElbowPlot(OP50_v2, ndims = 50)


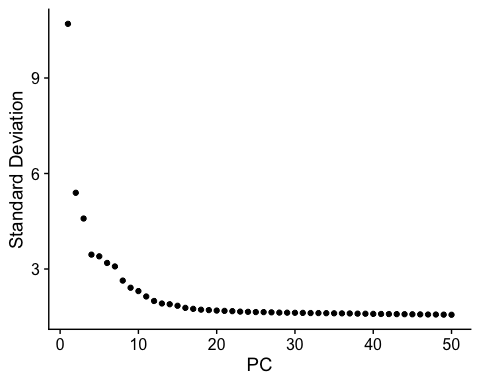


OP50_v2 <- FindNeighbors(OP50_v2, dims = 1:13, reduction = "pca")

Computing nearest neighbor graph

Computing SNN

OP50_v2 <- FindClusters(OP50_v2, resolution = 3.0, cluster.name = "OP50.unintegrated_clusters")

Modularity Optimizer version 1.3.0 by Ludo Waltman and Nees Jan van Eck

Number of nodes: 2361
Number of edges: 74357

Running Louvain algorithm...
Maximum modularity in 10 random starts: 0.7283
Number of communities: 26
Elapsed time: 0 seconds

OP50_v2 <- RunUMAP(OP50_v2, dims = 1:13, reduction = "pca", reduction.name = "umap.OP50.unintegrated")

11:09:57 UMAP embedding parameters a = 0.9922 b = 1.112

11:09:57 Read 2361 rows and found 13 numeric columns

11:09:57 Using Annoy for neighbor search, n_neighbors = 30

11:09:57 Building Annoy index with metric = cosine, n_trees = 50

0% 10 20 30 40 50 60 70 80 90 100%

[----|----|----|----|----|----|----|----|----|----|

**************************************************|
11:09:58 Writing NN index file to temp file /var/folders/2_/0b7d0hy11bd2g2nl32tfghfh981cxm/T//RtmpZVhw0e/file18f31b8ef7b
11:09:58 Searching Annoy index using 1 thread, search_k = 3000
11:09:58 Annoy recall = 100%
11:09:59 Commencing smooth kNN distance calibration using 1 thread with target n_neighbors = 30
11:10:01 Initializing from normalized Laplacian + noise (using RSpectra)
11:10:01 Commencing optimization for 500 epochs, with 89380 positive edges
11:10:01 Using rng type: pcg
11:10:04 Optimization finished

DimPlot(OP50_v2, reduction = "umap.OP50.unintegrated", label = T) + FeaturePlot(OP50_v2, reduction = "umap.OP50.unintegrated",
 features = c("nCount_RNA"),
 label = F, order = T, cols = c("#efefef", "#4011ba"))


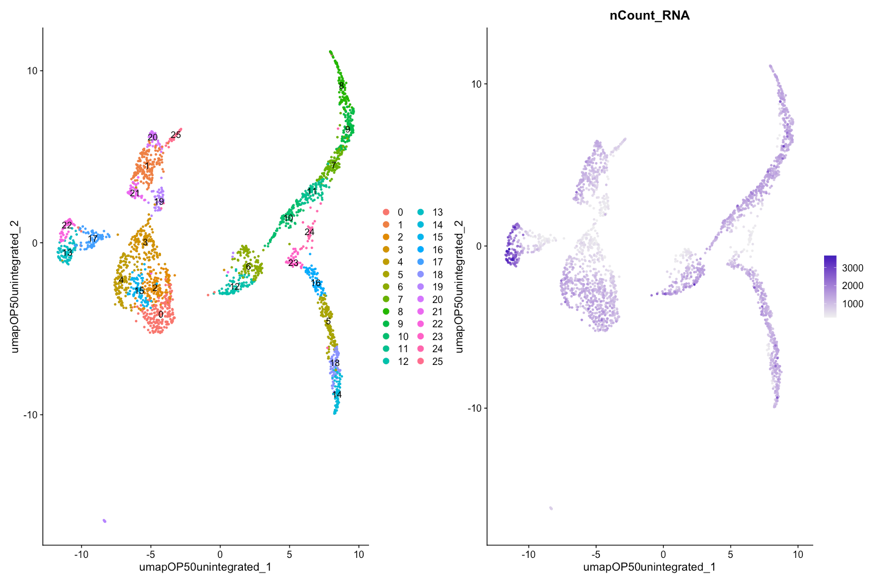


FeaturePlot(OP50_v2, reduction = "umap.OP50.unintegrated",
 features = c("mig-21","mab-5", "lin-39", "gcy-32", "mec-7", "ajm-1", "ast-1", "mec-3"),
 label = F, order = T, cols = c("#efefef", "#4011ba"), ncol = 4)


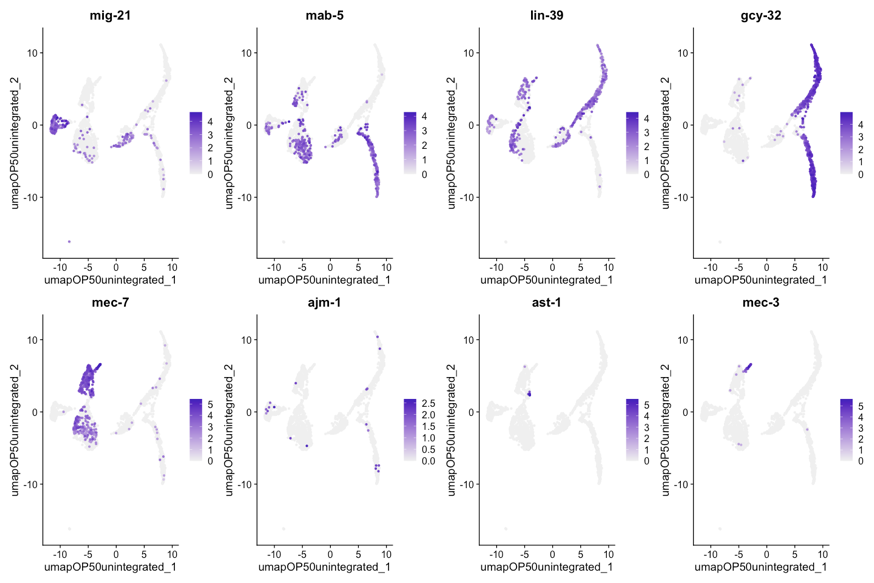


### 2nd round of cleaning - cluster-specific QC

*Note*: Arrested Q cells or QL.a/QL.p doublets might skew the data. Removing cells with low or high RNA count in specific clusters should prevent that.

DimPlot(OP50_v2, reduction = "umap.OP50.unintegrated", label = T) + VlnPlot(OP50_v2, features = "nCount_RNA")


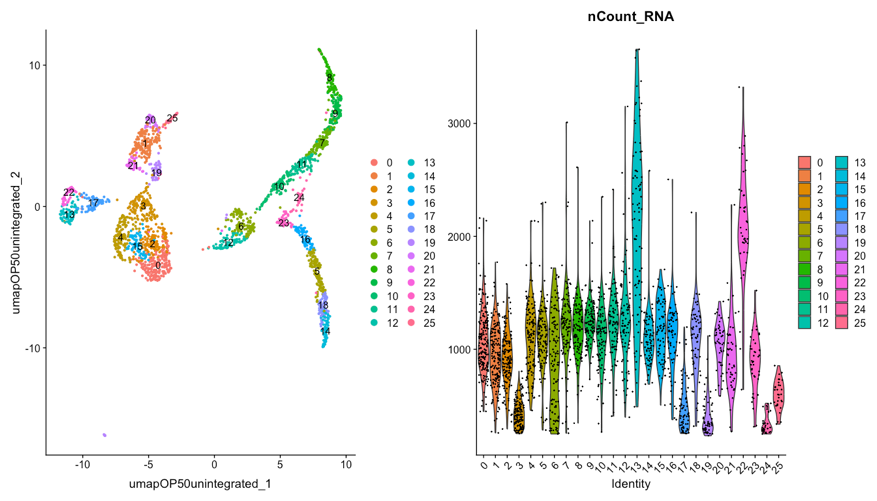


OP50.Q_to_remove <- WhichCells(OP50_v2, idents = c("13", "17", "22"), expression = nCount_RNA < 1300)
OP50.Q.d_to_remove_up <- WhichCells(OP50_v2, idents = c("0","1", "2", "3", "4", "5", "6", "7", "8", "9", "10", "11", "12", "14", "15", "16", "18", "19", "20", "21", "23", "25"), expression = nCount_RNA > 1900)
OP50.Q.d_to_remove_down <- WhichCells(OP50_v2, idents = c("0","1", "2", "3", "4", "5", "6", "7", "8", "9", "10", "11", "12", "14", "15", "16", "18", "20", "21", "23"), expression = nCount_RNA < 550)
OP50.extra_cells_to_remove.OP50_v2 <- WhichCells(OP50_v2, idents = c("24"))

OP50.Q_cells_to_remove <- c(OP50.Q_to_remove, OP50.Q.d_to_remove_up, OP50.Q.d_to_remove_down, OP50.extra_cells_to_remove.OP50_v2)

OP50_v3 <- subset(OP50_v2, cells = setdiff(Cells(OP50_v2), OP50.Q_cells_to_remove))

VlnPlot(OP50_v3, features = "nCount_RNA")


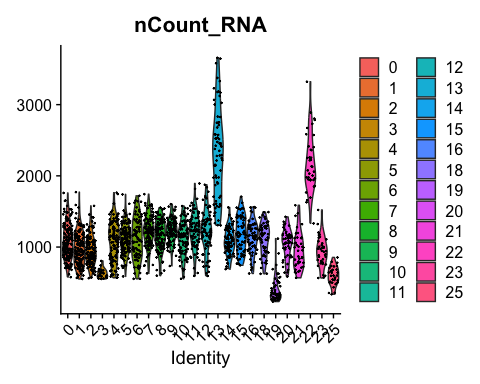


OP50_v3 <- NormalizeData(OP50_v3)

Normalizing layer: counts

OP50_v3 <- FindVariableFeatures(OP50_v3)

Finding variable features for layer counts

cele_features <- rownames(OP50_v3)
OP50_v3 <- ScaleData(OP50_v3, features = cele_features)

Centering and scaling data matrix

OP50_v3 <- RunPCA(OP50_v3)

PC_ 1
Positive: C27A2.8, mks-2, gcy-37, gcy-32, gcy-35, pdl-1, arl-3, dyf-6, F58B4.2, F49H12.4
 ZK616.1, F49E12.8, sbt-1, Y44A6D.2, T04C12.9, daf-25, WBGene00014307, arcp-1, fmi-1, T01D3.1
 ceph-41, gcy-34, ifta-2, che-2, dylt-2, guk-1, gcy-33, flp-14, Y17D7B.10, ptb-1
Negative: rpl-12, ctsa-1.1, rbm-3.2, rpl-7A, rpl-17, rpl-32, rpl-41.2, eef-1A.1, rps-18, eef-1B.1
 eef-2, mix-1, rnr-1, eef-1G, rpl-5, aldo-2, rpl-18, rps-1, rpl-20, rps-5
 rps-8, rpl-43, top-2, tba-2, cdk-1, rps-14, rpl-39, rpl-2, rps-23, rps-9
PC_ 2
Positive: hlh-14, egl-46, mec-7, F33A8.7, C08F1.10, ctsa-1.1, C09D4.2, dma-1, nid-1, C40A11.6
 F44E5.1, F39B2.3, WBGene00018813, C06E7.2, cki-1, WBGene00023302, atf-2, F36G3.1, tbca-1, C01C4.3
 K09F6.13, unc-68, smp-1, cank-26, tsp-7, Y47G6A.31, R11G1.2, C10A4.3, mec-3, C03C10.5
Negative: cpn-1, mig-21, pde-1, gcy-33, flp-21, suro-1, R05H11.2, unc-54, WBGene00022730, Y17D7B.10
 lec-4, flp-14, nex-4, T25G12.3, arcp-1, rps-25, nlp-43, ins-18, snt-4, rps-6
 egl-17, rpl-31, rla-1, pcn-1, rpl-30, mcm-6, rps-21, emb-9, rla-0, axl-1
PC_ 3
Positive: his-24, mec-7, hlh-14, F33A8.7, C09D4.2, pde-1, hil-3, unc-68, nex-4, snt-4
 WBGene00044308, flp-21, nlp-43, sem-4, ins-18, hil-2, T05A8.3, myrf-1, nid-1, nlp-47
 aexr-2, F58F9.1, zag-1, his-32, gcy-33, C01C4.3, ampd-1, hphd-1, srg-25, glb-5
Negative: unc-54, lin-32, gst-7, Y7A9D.1, che-2, F48E3.9, pkd-2, ham-1, T01D3.3, WBGene00023302
 F23B12.4, dma-1, K09F6.13, C03C10.5, ssq-1, mks-5, mam-8, fmi-1, msa-1, W07A12.4
 gasr-8, ccep-290, dsh-2, arl-3, smp-1, lipl-7, daf-25, F58E2.5, mig-21, eat-20
PC_ 4
Positive: F32H5.3, hil-2, rbm-3.2, pezo-1, hphd-1, mix-1, Y43F8B.2, cyd-1, ctsa-1.1, his-32
 top-2, Y47A7.2, cdk-1, cdl-1, myrf-1, his-60, tba-2, rfc-4, ztf-11, rnr-1
 ctf-8, tost-1, zag-1, Y43B11AR.1, C50F4.6, smc-4, mcm-7, tbb-1, T24C4.2, pmp-3
Negative: WBGene00023209, Y39B6A.5, lbp-1, cpi-1, dig-1, clec-178, lgc-25, C07G3.10, Y116A8C.3, cup-4
 Y73F4A.1, ttr-1, T19C3.5, lgc-28, lgc-26, ccg-1, mig-6, K07E3.4, inos-1, unc-122
 Y61A9LA.7, hot-5, B0393.9, Y54F10BM.12, F32E10.8, lgc-23, K02E7.11, F56C4.4, lbp-2, far-1
PC_ 5
Positive: flp-5, F44E5.1, T05A8.3, E04F6.10, die-1, nlp-43, mig-13, plc-1, nlp-47, ssq-1
 glna-3, pkd-2, col-114, C10C5.7, srg-25, dgk-5, C24B5.1, pdf-1, srd-30, WBGene00044308
 mig-39, F58F9.1, sre-4, magi-1, srd-29, smp-1, F32B4.5, F42A9.9, flp-7, mam-8
Negative: flp-4, mab-5, ZK742.7, M162.5, cutl-10, lat-1, cyk-7, nlp-40, glb-24, F35B3.7
 seb-3, npr-9, H01M10.2, flp-9, C33A12.4, grd-12, hyl-1, nmr-1, test-1, kvs-2
 cle-1, ZK867.2, sto-5, F32H5.3, fkh-5, dct-14, C34D1.4, flp-27, sorf-2, wht-1

ElbowPlot(OP50_v3, ndims = 50)


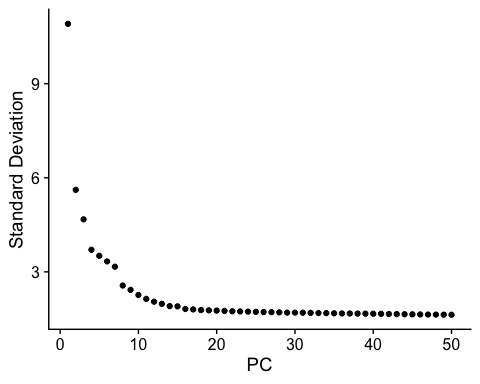


OP50_v3 <- FindNeighbors(OP50_v3, dims = 1:14, reduction = "pca")

Computing nearest neighbor graph

Computing SNN

OP50_v3 <- FindClusters(OP50_v3, resolution = 1.0, cluster.name = "OP50.unintegrated_clusters")

Modularity Optimizer version 1.3.0 by Ludo Waltman and Nees Jan van Eck

Number of nodes: 1995
Number of edges: 64117

Running Louvain algorithm...
Maximum modularity in 10 random starts: 0.8493
Number of communities: 13
Elapsed time: 0 seconds

OP50_v3 <- RunUMAP(OP50_v3, dims = 1:14, reduction = "pca", reduction.name = "umap.OP50.unintegrated")

11:10:16 UMAP embedding parameters a = 0.9922 b = 1.112

11:10:16 Read 1995 rows and found 14 numeric columns

11:10:16 Using Annoy for neighbor search, n_neighbors = 30

11:10:16 Building Annoy index with metric = cosine, n_trees = 50

0% 10 20 30 40 50 60 70 80 90 100%

[----|----|----|----|----|----|----|----|----|----|

**************************************************|
11:10:16 Writing NN index file to temp file /var/folders/2_/0b7d0hy11bd2g2nl32tfghfh981cxm/T//RtmpZVhw0e/file18f3743a528b
11:10:16 Searching Annoy index using 1 thread, search_k = 3000
11:10:16 Annoy recall = 100%
11:10:17 Commencing smooth kNN distance calibration using 1 thread with target n_neighbors = 30
11:10:19 Initializing from normalized Laplacian + noise (using RSpectra)
11:10:19 Commencing optimization for 500 epochs, with 75276 positive edges
11:10:19 Using rng type: pcg
11:10:22 Optimization finished

DimPlot(OP50_v3, reduction = "umap.OP50.unintegrated", label = T) + FeaturePlot(OP50_v3, reduction = "umap.OP50.unintegrated",
 features = c("nCount_RNA"),
 label = F, order = T, cols = c("#efefef", "#4011ba"))


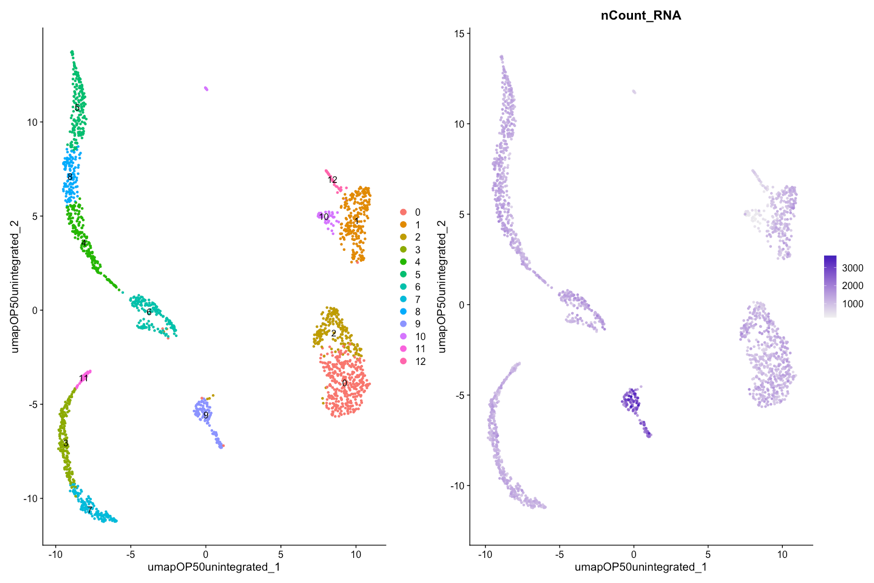


FeaturePlot(OP50_v3, reduction = "umap.OP50.unintegrated",
 features = c("mig-21","mab-5", "lin-39", "gcy-32", "mec-7", "ajm-1", "mec-3", "ast-1"),
 label = F, order = T, cols = c("#efefef", "#4011ba"), ncol = 4)


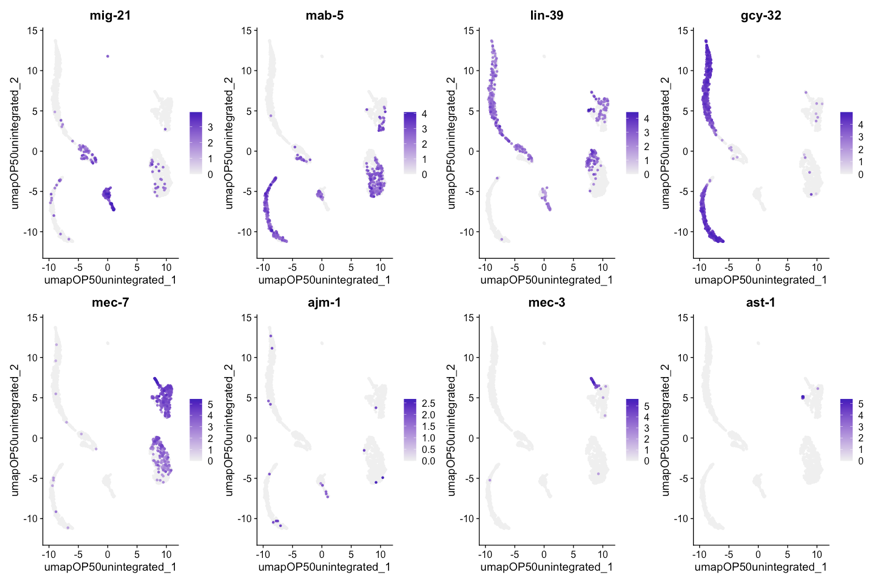


## PA14 (WT_Q_cells_2_PA14_14h)

PA14 <- NormalizeData(PA14)

Normalizing layer: counts

PA14 <- FindVariableFeatures(PA14)

Finding variable features for layer counts

cele_features <- rownames(PA14)
PA14 <- ScaleData(PA14, features = cele_features)

Centering and scaling data matrix

PA14 <- RunPCA(PA14)

PC_ 1
Positive: C27A2.8, mks-2, gcy-32, gcy-37, arl-3, gcy-35, F49H12.4, pdl-1, dyf-6, ZK616.1
 F58B4.2, F49E12.8, T04C12.9, sbt-1, Y44A6D.2, gcy-36, T01D3.1, fmi-1, WBGene00014307, K07C11.10
 daf-25, ifta-2, ceph-41, mksr-2, arcp-1, tmem-231, gcy-34, tmem-218, dylt-2, guk-1
Negative: rpl-12, rpl-7A, rbm-3.2, rpl-17, eef-1A.1, eef-2, rpl-32, rps-18, eef-1B.1, rpl-41.2
 rpl-36, eef-1G, rpl-18, rpl-10, rps-1, rps-8, rpl-20, rpl-5, rpl-1, rps-4
 pab-1, rpl-3, rpl-16, rps-23, rps-5, rpl-43, rps-0, rpl-7, rpl-2, rps-7
PC_ 2
Positive: ram-2, col-107, col-117, col-3, col-98, col-103, col-94, nlp-29, sams-1, F46F2.3
 grd-10, nlp-24, pmt-2, col-10, ttr-15, F26G1.2, grd-13, R09E12.9, grd-3, lips-10
 T25G12.3, upp-1, col-42, gst-20, F17H10.2, nlp-33, T01C8.2, zipt-16, fasn-1, nhr-270
Negative: his-24, mec-7, C09D4.2, hlh-14, hil-2, F33A8.7, tbca-1, egl-46, C08F1.10, calu-1
 his-60, Y47G6A.31, hil-3, nid-1, his-32, strl-1, F07C6.4, C50F4.6, mig-1, zag-1
 C40A11.6, R11G1.2, myrf-1, C01C4.3, tmed-13, smp-1, sem-4, rhgf-2, cank-26, WBGene00018813
PC_ 3
Positive: pde-1, nex-4, nlp-43, ins-18, T05A8.3, flp-21, WBGene00044308, gcy-33, snt-4, dgk-5
 nphp-2, pgal-1, C08G9.1, aexr-2, C10C5.7, Y17D7B.10, F32B4.5, F58F9.1, glb-1, ampd-1
 B0205.14, plc-1, glb-5, his-24, K02F2.5, Y119C1B.6, F58H1.7, pdf-1, F38E9.6, flp-14
Negative: T01D3.3, dma-1, C03C10.5, F23B12.4, F48E3.9, WBGene00023302, daf-19, K09F6.13, gst-7, Y7A9D.1
 ham-1, egl-46, F58E2.5, tctn-1, mks-5, C04A11.2, ssq-1, pkd-2, gasr-8, R01H2.8
 lin-32, tmem-231, eat-20, M162.5, che-2, ccep-290, vab-8, cki-1, fmil-1, fmi-1
PC_ 4
Positive: mig-21, unc-54, lin-32, R05H11.2, W07A12.4, cpn-1, tbx-2, egl-17, suro-1, Y54H5A.2
 che-2, lec-4, rad-51, mex-3, mcm-7, cdh-3, msa-1, mcm-6, afd-1, pcn-1
 rps-1, mcm-2, tsp-14, rps-7, rps-9, rps-26, rpl-3, rpl-36.A, WBGene00022730, rpl-7
Negative: hlh-14, mec-7, F33A8.7, nid-1, F36G3.1, C09D4.2, C01C4.3, C40A11.6, C08F1.10, unc-68
 tsp-7, WBGene00018813, tbca-1, C06E7.2, mig-1, sem-4, col-107, R11G1.2, F16F9.1, Y47G6A.31
 C15C8.5, F44E5.1, ram-2, W10D9.1, C10A4.3, nlp-29, tmed-13, WBGene00050903, unc-52, zag-1
PC_ 5
Positive: mab-5, flp-4, ZK742.7, lat-1, cutl-10, F32H5.3, C33A12.4, nlp-40, hphd-1, cyk-7
 M162.5, lin-24, F35B3.7, odr-4, dct-14, cyd-1, hyl-1, wht-1, npr-9, gcy-34
 fkh-5, ZK867.2, sto-5, ZC449.5, guk-1, glb-24, clc-4, M01H9.4, seb-3, zig-8
Negative: pkd-2, srd-30, ssq-1, srd-29, lipl-7, mig-13, smp-1, flp-5, lgc-27, tbx-2
 Y54H5A.2, lin-32, WBGene00023302, gst-7, E04F6.10, F07C6.4, Y7A9D.1, C43D7.8, nlp-43, T05A8.3
 cam-1, plc-1, magi-1, Y71F9AL.6, C10C5.7, F58H1.7, unc-54, lgc-52, eat-20, pdf-1

ElbowPlot(PA14, ndims = 50)


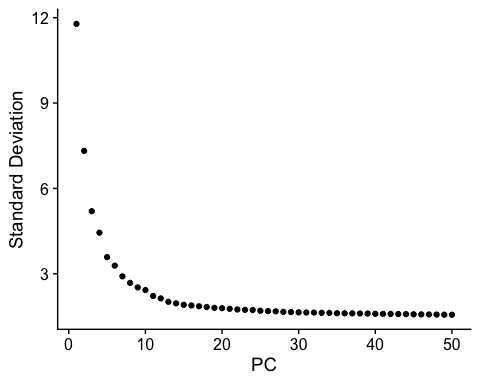


PA14 <- FindNeighbors(PA14, dims = 1:11, reduction = "pca")

Computing nearest neighbor graph

Computing SNN

PA14 <- FindClusters(PA14, resolution = 1.0, cluster.name = "PA14.unintegrated_clusters")

Modularity Optimizer version 1.3.0 by Ludo Waltman and Nees Jan van Eck

Number of nodes: 2745
Number of edges: 87205

Running Louvain algorithm...
Maximum modularity in 10 random starts: 0.8709
Number of communities: 13
Elapsed time: 0 seconds

PA14 <- RunUMAP(PA14, dims = 1:11, reduction = "pca", reduction.name = "umap.PA14.unintegrated")

11:10:33 UMAP embedding parameters a = 0.9922 b = 1.112

11:10:33 Read 2745 rows and found 11 numeric columns

11:10:33 Using Annoy for neighbor search, n_neighbors = 30

11:10:33 Building Annoy index with metric = cosine, n_trees = 50

0% 10 20 30 40 50 60 70 80 90 100%

[----|----|----|----|----|----|----|----|----|----|

**************************************************|
11:10:33 Writing NN index file to temp file /var/folders/2_/0b7d0hy11bd2g2nl32tfghfh981cxm/T//RtmpZVhw0e/file18f34f2d8722
11:10:33 Searching Annoy index using 1 thread, search_k = 3000
11:10:34 Annoy recall = 100%
11:10:35 Commencing smooth kNN distance calibration using 1 thread with target n_neighbors = 30
11:10:37 Initializing from normalized Laplacian + noise (using RSpectra)
11:10:37 Commencing optimization for 500 epochs, with 105748 positive edges
11:10:37 Using rng type: pcg
11:10:41 Optimization finished

DimPlot(PA14, reduction = "umap.PA14.unintegrated", label = T) + FeaturePlot(PA14, reduction = "umap.PA14.unintegrated",
 features = c("nCount_RNA"),
 label = F, order = T, cols = c("#efefef", "#4011ba"))


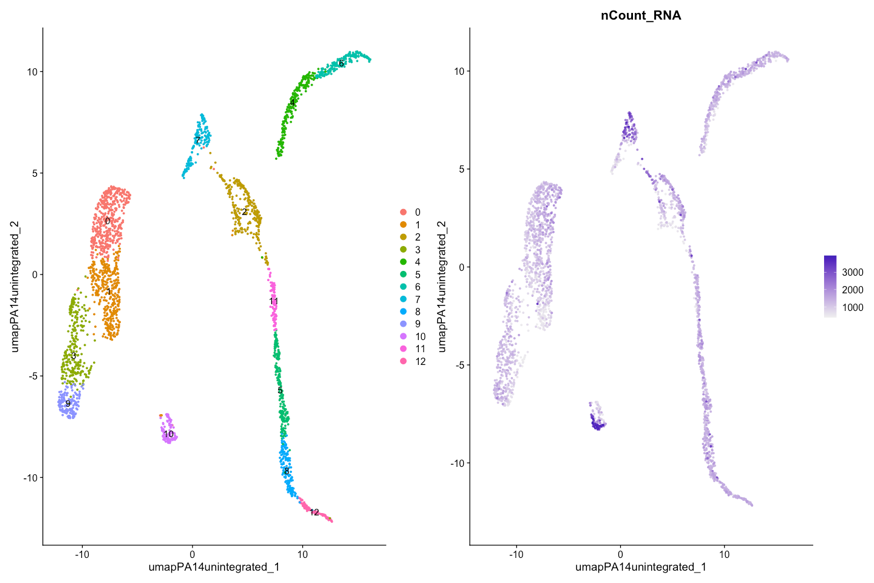


FeaturePlot(PA14, reduction = "umap.PA14.unintegrated",
 features = c("mig-21","mab-5", "lin-39", "gcy-32", "mec-7", "ajm-1", "mec-3", "ast-1"),
 label = F, order = T, cols = c("#efefef", "#4011ba"), ncol = 4)


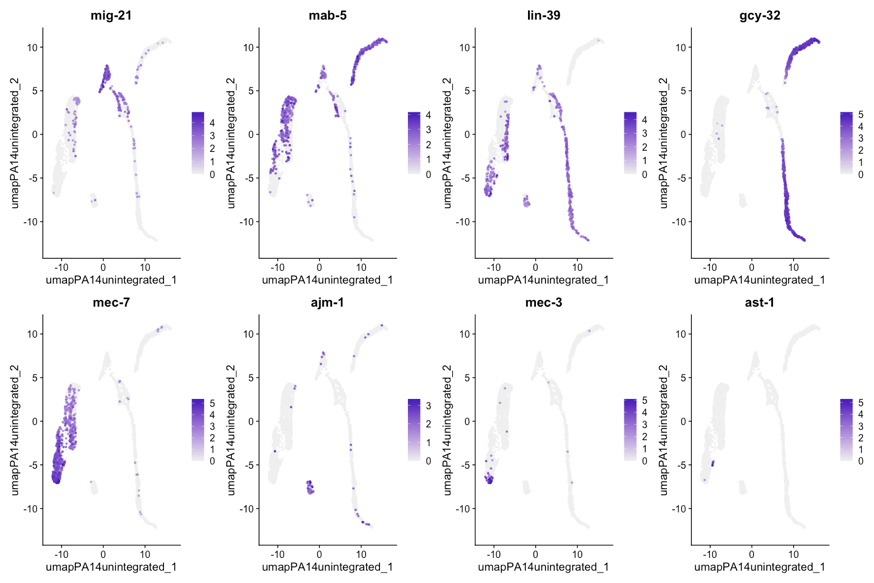


### 1st round of cleaning - removing non-Q cells

PA14.extra_cells_to_remove.PA14 <- WhichCells(PA14, idents = c("10"))
PA14_v2 <- subset(PA14, cells = setdiff(Cells(PA14), PA14.extra_cells_to_remove.PA14))

PA14_v2 <- NormalizeData(PA14_v2)

Normalizing layer: counts

PA14_v2 <- FindVariableFeatures(PA14_v2)

Finding variable features for layer counts

cele_features <- rownames(PA14_v2)
PA14_v2 <- ScaleData(PA14_v2, features = cele_features)

Centering and scaling data matrix

PA14_v2 <- RunPCA(PA14_v2)

PC_ 1
Positive: rpl-12, ctsa-1.1, rpl-7A, rbm-3.2, rpl-17, asp-4, eef-1A.1, ZK930.6, his-24, rpl-32
 rps-18, rpl-41.2, eef-1B.1, rpl-36, rnr-1, eef-1G, rpl-18, rpl-10, aldo-2, rps-1
 rps-8, rpl-20, rpl-5, fkb-2, rpl-43, rpl-1, his-32, rps-4, R05H5.3, rpl-16
Negative: mks-2, gcy-37, gcy-32, C27A2.8, arl-3, pdl-1, dyf-6, gcy-35, ZK616.1, F58B4.2
 F49E12.8, T04C12.9, sbt-1, gcy-36, Y44A6D.2, T01D3.1, fmi-1, WBGene00014307, K07C11.10, daf-25
 ifta-2, ceph-41, mksr-2, arcp-1, tmem-231, gcy-34, tmem-218, dylt-2, che-2, guk-1
PC_ 2
Positive: egl-46, dma-1, T01D3.3, C03C10.5, WBGene00023302, F23B12.4, syd-9, daf-19, K09F6.13, F48E3.9
 gst-7, ham-1, Y7A9D.1, rhgf-2, cki-1, ssq-1, jbts-14, F58E2.5, pkd-2, dgn-1
 C04A11.2, Y51H4A.1, eat-20, Y47H10A.4, gasr-8, F44E5.1, tag-275, tctn-1, smp-1, WBGene00018199
Negative: pde-1, nex-4, flp-21, nlp-43, ins-18, gcy-33, T05A8.3, Y17D7B.10, snt-4, pgal-1
 nphp-2, WBGene00044308, glb-1, flp-14, dgk-5, C08G9.1, C10C5.7, aexr-2, B0205.14, F32B4.5
 ampd-1, F58H1.7, lact-1, B0205.13, K02F2.5, plc-1, Y45G5AM.6, glb-5, F38E9.6, Y119C1B.6
PC_ 3
Positive: unc-54, lin-32, W07A12.4, mig-21, R05H11.2, che-2, cpn-1, msa-1, egl-17, Y7A9D.1
 rpl-36.A, lec-4, dpy-20, rps-7, rps-1, rpl-3, suro-1, rpl-31, rla-0, rpl-15
 tbx-2, rps-26, rps-0, rps-17, rpl-7, ham-1, rack-1, rps-9, F47B7.1, W01D2.1
Negative: mec-7, hlh-14, F33A8.7, nid-1, C09D4.2, unc-68, F36G3.1, C01C4.3, C40A11.6, tbca-1
 mig-1, sem-4, his-24, tsp-7, WBGene00018813, C06E7.2, Y47G6A.31, asp-4, zag-1, F16F9.1
 W10D9.1, C15C8.5, C10A4.3, lbp-3, tmed-13, unc-52, myrf-1, C08F1.6, C14H10.2, WBGene00050903
PC_ 4
Positive: srd-30, pkd-2, lipl-7, srd-29, ssq-1, lin-32, lgc-27, tbx-2, smp-1, Y54H5A.2
 mig-13, WBGene00023302, C43D7.8, Y7A9D.1, flp-5, F07C6.4, Y71F9AL.6, F58H1.7, gst-7, unc-54
 T05A8.3, cam-1, lgc-52, ZK131.11, magi-1, nlp-43, plc-1, pdf-1, ham-1, exc-9
Negative: mab-5, F32H5.3, hphd-1, lat-1, C33A12.4, flp-4, cutl-10, ZK742.7, cyd-1, Y43B11AR.1
 M162.5, cyk-7, F35B3.7, nlp-40, dct-14, odr-4, lin-24, pezo-1, hyl-1, gcy-34
 cle-1, fkh-5, cdl-1, ZC449.5, guk-1, fkb-2, riok-1, glb-24, unc-5, sto-5
PC_ 5
Positive: flp-4, flp-9, egl-46, ZK742.7, kvs-2, test-1, lin-32, npr-9, zig-8, nlp-40
 exp-2, wht-1, lin-24, vab-8, cutl-10, ham-1, lipl-7, Y71F9AL.6, ZK938.3, aexr-2
 ZK867.2, lgc-27, K02B12.9, F46F2.5, ten-1, flp-21, sto-5, cutl-26, glb-23, sorf-2
Negative: flp-5, E04F6.10, mam-8, F44E5.1, mig-39, glna-3, C24B5.1, syg-1, col-114, R09F10.5
 ric-4, mig-13, nlp-43, F54F12.2, C52B9.10, F32H5.3, hphd-1, C10C5.7, lin-39, C17B7.8
 igcm-4, T03G6.3, WBGene00019181, F17A2.16, cyd-1, C39D10.5, plc-1, Y45G5AM.6, tppp-1, cyp-31A2

ElbowPlot(PA14_v2, ndims = 50)


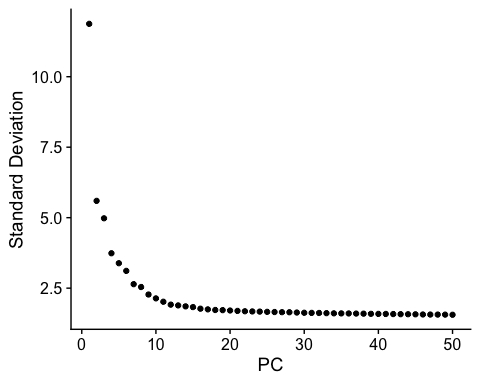


PA14_v2 <- FindNeighbors(PA14_v2, dims = 1:12, reduction = "pca")

Computing nearest neighbor graph

Computing SNN

PA14_v2 <- FindClusters(PA14_v2, resolution = 1.0, cluster.name = "PA14.unintegrated_clusters")

Modularity Optimizer version 1.3.0 by Ludo Waltman and Nees Jan van Eck

Number of nodes: 2640
Number of edges: 83887

Running Louvain algorithm...
Maximum modularity in 10 random starts: 0.8539
Number of communities: 14
Elapsed time: 0 seconds

PA14_v2 <- RunUMAP(PA14_v2, dims = 1:12, reduction = "pca", reduction.name = "umap.PA14.unintegrated")

11:10:52 UMAP embedding parameters a = 0.9922 b = 1.112

11:10:52 Read 2640 rows and found 12 numeric columns

11:10:52 Using Annoy for neighbor search, n_neighbors = 30

11:10:52 Building Annoy index with metric = cosine, n_trees = 50

0% 10 20 30 40 50 60 70 80 90 100%

[----|----|----|----|----|----|----|----|----|----|

**************************************************|
11:10:52 Writing NN index file to temp file /var/folders/2_/0b7d0hy11bd2g2nl32tfghfh981cxm/T//RtmpZVhw0e/file18f37bc8a05b
11:10:52 Searching Annoy index using 1 thread, search_k = 3000
11:10:53 Annoy recall = 100%
11:10:54 Commencing smooth kNN distance calibration using 1 thread with target n_neighbors = 30
11:10:55 Initializing from normalized Laplacian + noise (using RSpectra)
11:10:55 Commencing optimization for 500 epochs, with 101520 positive edges
11:10:55 Using rng type: pcg
11:10:59 Optimization finished

DimPlot(PA14_v2, reduction = "umap.PA14.unintegrated", label = T) + FeaturePlot(PA14_v2, reduction = "umap.PA14.unintegrated",
 features = c("nCount_RNA"),
 label = F, order = T, cols = c("#efefef", "#4011ba"))


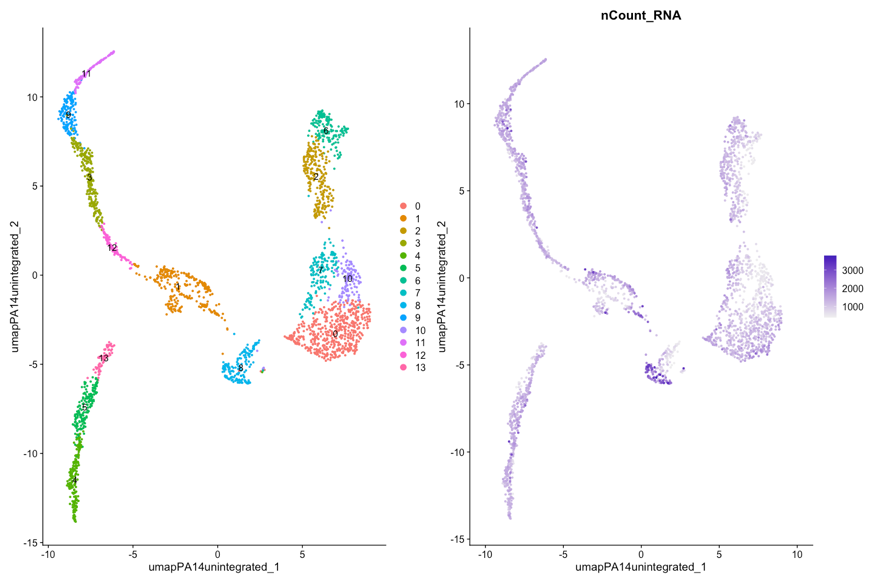


FeaturePlot(PA14_v2, reduction = "umap.PA14.unintegrated",
 features = c("mig-21","mab-5", "lin-39", "gcy-32", "mec-7", "ajm-1", "mec-3", "ast-1"),
 label = F, order = T, cols = c("#efefef", "#4011ba"), ncol = 3)


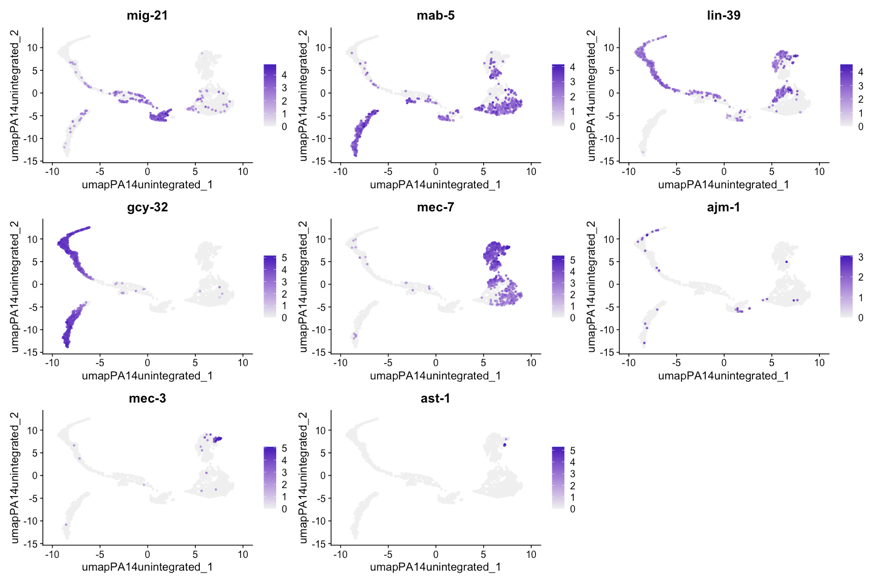


### 2nd round of cleaning - cluster-specific QC

DimPlot(PA14_v2, reduction = "umap.PA14.unintegrated", label = T) + VlnPlot(PA14_v2, features = "nCount_RNA")


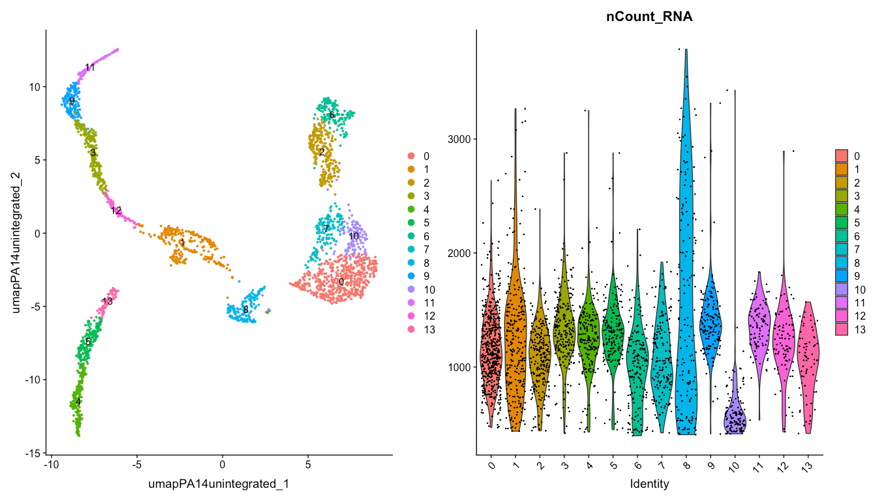


DimPlot(PA14_v2, reduction = "umap.PA14.unintegrated", label = T) + FeaturePlot(PA14_v2, reduction = "umap.PA14.unintegrated",
 features = c("nCount_RNA"),
 label = F, order = T, cols = c("#efefef", "#4011ba"))


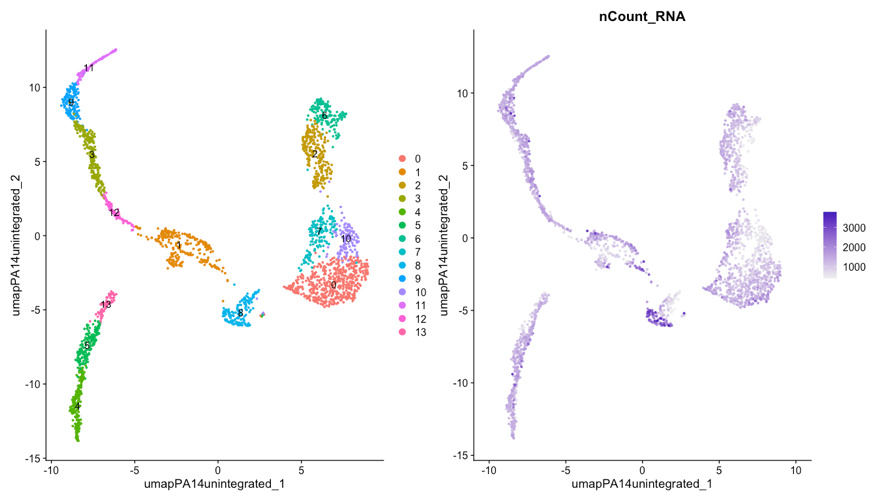


PA14.Q_to_remove <- WhichCells(PA14_v2, idents = c("8"), expression = nCount_RNA < 1300)
PA14.Q.d_to_remove_up <- WhichCells(PA14_v2, idents = c("0","1", "2", "3", "4", "5", "6", "7", "9", "10", "11", "12", "13"), expression = nCount_RNA > 1900)
PA14.Q.d_to_remove_down <- WhichCells(PA14_v2, idents = c("0","1", "2", "3", "4", "5", "7", "9", "10", "11", "12", "13"), expression = nCount_RNA < 550)

PA14.Q_cells_to_remove <- c(PA14.Q_to_remove, PA14.Q.d_to_remove_up, PA14.Q.d_to_remove_down)

PA14_v3 <- subset(PA14_v2, cells = setdiff(Cells(PA14_v2), PA14.Q_cells_to_remove))
VlnPlot(PA14_v3, features = "nCount_RNA")


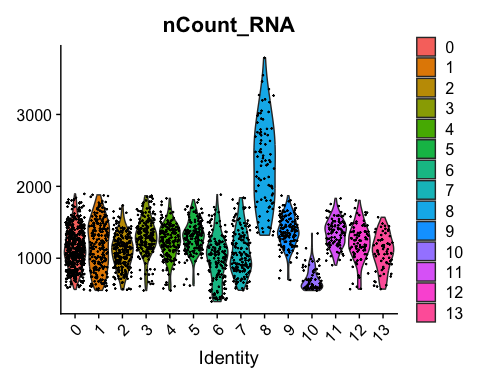


PA14_v3 <- NormalizeData(PA14_v3)

Normalizing layer: counts

PA14_v3 <- FindVariableFeatures(PA14_v3)

Finding variable features for layer counts

cele_features <- rownames(PA14_v3)
PA14_v3 <- ScaleData(PA14_v3, features = cele_features)

Centering and scaling data matrix

PA14_v3 <- RunPCA(PA14_v3)

PC_ 1
Positive: rpl-12, ctsa-1.1, rbm-3.2, rpl-7A, asp-4, ZK930.6, rpl-17, eef-1A.1, rpl-32, rpl-41.2
 his-24, eef-2, rps-18, eef-1B.1, rpl-36, rnr-1, eef-1G, aldo-2, rpl-18, rps-8
 rpl-10, his-32, rpl-43, rps-1, rpl-20, his-60, rpl-5, R05H5.3, rpl-1, hil-2
Negative: mks-2, gcy-32, gcy-37, C27A2.8, arl-3, F49H12.4, dyf-6, pdl-1, gcy-35, ZK616.1
 F58B4.2, F49E12.8, T04C12.9, gcy-36, Y44A6D.2, WBGene00014307, T01D3.1, fmi-1, daf-25, K07C11.10
 mksr-2, ceph-41, ifta-2, arcp-1, che-2, tmem-231, tmem-218, dylt-2, guk-1, flp-14
PC_ 2
Positive: pde-1, nex-4, flp-21, nlp-43, ins-18, gcy-33, T05A8.3, Y17D7B.10, snt-4, pgal-1
 nphp-2, WBGene00044308, glb-1, flp-14, C08G9.1, C10C5.7, aexr-2, B0205.14, ampd-1, F32B4.5
 F58H1.7, lact-1, B0205.13, K02F2.5, plc-1, Y45G5AM.6, glb-5, F58F9.1, Y119C1B.6, F38E9.6
Negative: egl-46, dma-1, T01D3.3, C03C10.5, WBGene00023302, F23B12.4, syd-9, daf-19, Y7A9D.1, K09F6.13
 F48E3.9, ham-1, gst-7, pkd-2, rhgf-2, jbts-14, F58E2.5, ssq-1, dgn-1, C04A11.2
 Y47H10A.4, gasr-8, tctn-1, eat-20, F44E5.1, smp-1, tag-275, Y71F9AL.6, mks-5, tmem-231
PC_ 3
Positive: unc-54, lin-32, R05H11.2, W07A12.4, mig-21, cpn-1, che-2, msa-1, egl-17, suro-1
 rpl-36.A, rpl-31, rps-1, lec-4, rps-7, Y7A9D.1, tbx-2, rps-26, rpl-3, ham-1
 WBGene00022730, rla-0, dpy-20, rps-0, Y37E3.8, rps-25, rps-17, rps-9, rps-14, W01D2.1
Negative: hlh-14, mec-7, F33A8.7, nid-1, C09D4.2, F36G3.1, unc-68, C01C4.3, mig-1, C40A11.6
 tbca-1, C08F1.10, sem-4, WBGene00018813, tsp-7, his-24, R11G1.2, C06E7.2, Y47G6A.31, zag-1
 asp-4, F16F9.1, W10D9.1, C15C8.5, C10A4.3, tmed-13, lbp-3, C08F1.6, WBGene00050903, myrf-1
PC_ 4
Positive: srd-30, pkd-2, lipl-7, srd-29, lin-32, ssq-1, lgc-27, tbx-2, smp-1, Y54H5A.2
 mig-13, C43D7.8, WBGene00023302, F07C6.4, Y7A9D.1, Y71F9AL.6, F58H1.7, lgc-52, gst-7, unc-54
 flp-5, cam-1, ZK131.11, exc-9, ham-1, T05A8.3, magi-1, pdf-1, W07A12.4, eat-20
Negative: F32H5.3, mab-5, hphd-1, lat-1, cyd-1, C33A12.4, cutl-10, M162.5, flp-4, cyk-7
 pezo-1, F35B3.7, ZK742.7, nlp-40, hyl-1, dct-14, odr-4, lin-24, fkh-5, ZC449.5
 cle-1, riok-1, guk-1, unc-5, myrf-1, glb-24, Y47A7.2, T22B7.22, mcm-7, seb-3
PC_ 5
Positive: flp-5, E04F6.10, mam-8, F44E5.1, mig-39, glna-3, C24B5.1, syg-1, col-114, mig-13
 ric-4, R09F10.5, nlp-43, lin-39, F54F12.2, C52B9.10, C10C5.7, C17B7.8, igcm-4, WBGene00019181
 F17A2.16, F32H5.3, cyp-31A2, T03G6.3, hphd-1, plc-1, cab-1, C39D10.5, Y45G5AM.6, tppp-1
Negative: flp-4, flp-9, ZK742.7, kvs-2, egl-46, test-1, lin-32, nlp-40, npr-9, zig-8
 exp-2, wht-1, lin-24, cutl-10, vab-8, ham-1, ZK938.3, Y71F9AL.6, lipl-7, aexr-2
 K02B12.9, ZK867.2, sorf-2, sto-5, ten-1, cutl-26, glb-23, flp-21, F46F2.5, nlp-8

ElbowPlot(PA14_v3, ndims = 50)


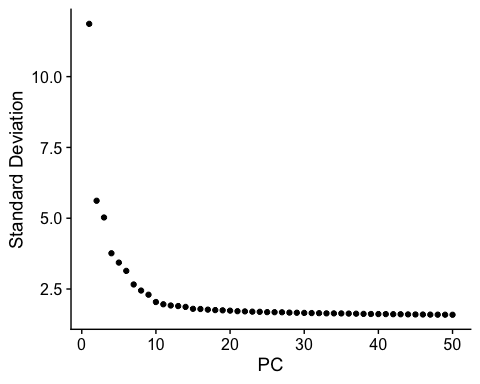


PA14_v3 <- FindNeighbors(PA14_v3, dims = 1:12, reduction = "pca")

Computing nearest neighbor graph

Computing SNN

PA14_v3 <- FindClusters(PA14_v3, resolution = 1.0, cluster.name = "PA14.unintegrated_clusters")

Modularity Optimizer version 1.3.0 by Ludo Waltman and Nees Jan van Eck

Number of nodes: 2384
Number of edges: 76465

Running Louvain algorithm...
Maximum modularity in 10 random starts: 0.8571
Number of communities: 13
Elapsed time: 0 seconds

PA14_v3 <- RunUMAP(PA14_v3, dims = 1:12, reduction = "pca", reduction.name = "umap.PA14.unintegrated")

11:11:12 UMAP embedding parameters a = 0.9922 b = 1.112

11:11:12 Read 2384 rows and found 12 numeric columns

11:11:12 Using Annoy for neighbor search, n_neighbors = 30

11:11:12 Building Annoy index with metric = cosine, n_trees = 50

0% 10 20 30 40 50 60 70 80 90 100%

[----|----|----|----|----|----|----|----|----|----|

**************************************************|
11:11:12 Writing NN index file to temp file /var/folders/2_/0b7d0hy11bd2g2nl32tfghfh981cxm/T//RtmpZVhw0e/file18f39fe578
11:11:12 Searching Annoy index using 1 thread, search_k = 3000
11:11:12 Annoy recall = 100%
11:11:13 Commencing smooth kNN distance calibration using 1 thread with target n_neighbors = 30
11:11:15 Initializing from normalized Laplacian + noise (using RSpectra)
11:11:15 Commencing optimization for 500 epochs, with 90472 positive edges
11:11:15 Using rng type: pcg
11:11:19 Optimization finished

DimPlot(PA14_v3, reduction = "umap.PA14.unintegrated", label = T) + FeaturePlot(PA14_v3, reduction = "umap.PA14.unintegrated",
 features = c("nCount_RNA"),
 label = F, order = T, cols = c("#efefef", "#4011ba"))


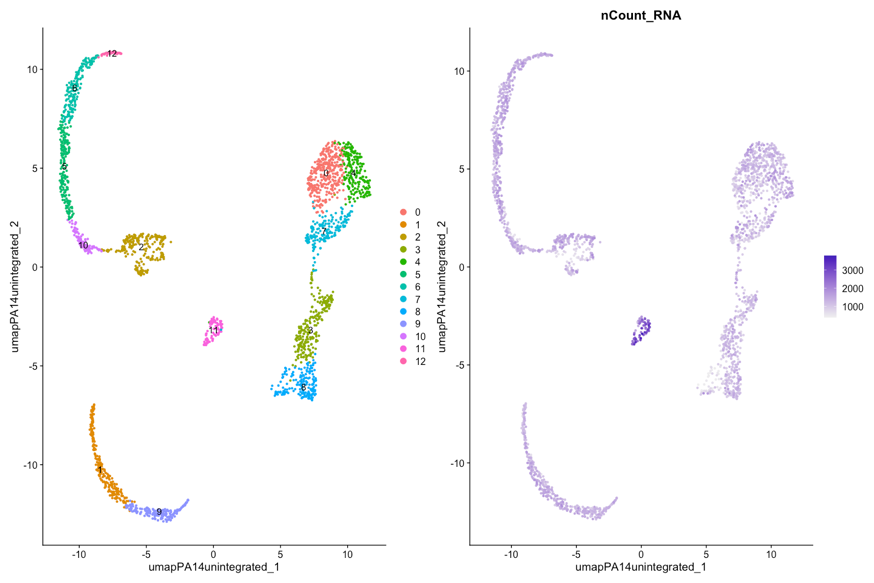


FeaturePlot(PA14_v3, reduction = "umap.PA14.unintegrated",
 features = c("mig-21","mab-5", "lin-39", "gcy-32", "mec-7", "ajm-1", "mec-3", "ast-1"),
 label = F, order = T, cols = c("#efefef", "#4011ba"), ncol = 4)


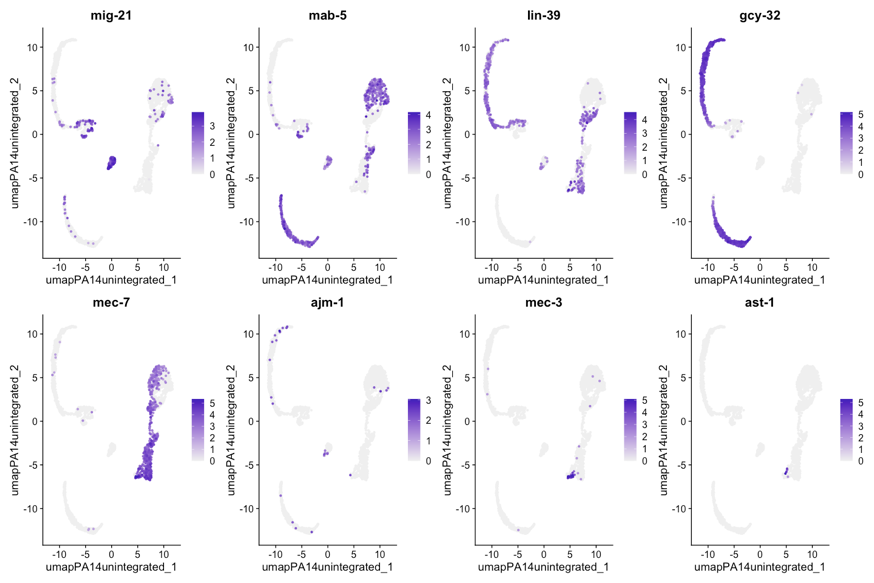


## OP50.2 (WT_Q_cells_3_OP50_12-16h)

OP50.2 <- NormalizeData(OP50.2)

Normalizing layer: counts

OP50.2 <- FindVariableFeatures(OP50.2)

Finding variable features for layer counts

cele_features <- rownames(OP50.2)
OP50.2 <- ScaleData(OP50.2, features = cele_features)

Centering and scaling data matrix

OP50.2 <- RunPCA(OP50.2)

PC_ 1
Positive: npa-1, vha-6, R08E3.1, haf-4, C49C3.4, H43E16.1, dsc-4, cpz-2, Y16B4A.2, pcp-3
 T02C5.1, ifo-1, K06G5.1, ctsa-2, pqn-52, hpo-34, tag-244, ifp-1, T25C12.3, Y102A11A.3
 C18H9.6, M04C3.1, Y51A2D.13, K06A9.1, nep-17, W05H9.1, irg-7, F56F10.1, ZK6.11, asp-1
Negative: mks-2, gcy-37, C27A2.8, arl-3, pdl-1, dyf-6, gcy-32, F49H12.4, gcy-35, F49E12.8
 ZK616.1, ceph-41, gcy-36, mksr-2, fmi-1, T04C12.9, tmem-231, daf-25, dylt-2, F58B4.2
 ifta-2, nphp-1, K07C11.10, tmem-218, T01D3.1, mksr-1, ift-81, arcp-1, ift-20, Y44A6D.2
PC_ 2
Positive: col-103, M60.4, col-94, nlp-33, brp-1, cest-24, ttr-15, sams-1, qdpr-1, col-98
 nlp-29, lips-10, col-166, col-42, col-147, lec-4, C39E9.8, col-39, pck-1, col-160
 lec-2, K02F3.9, col-10, fbxa-24, srap-1, ttr-18, pmt-2, C35C5.9, pah-1, nlp-27
Negative: che-2, npa-1, vha-6, haf-4, R08E3.1, C49C3.4, dsc-4, H43E16.1, gcy-37, mks-2
 gcy-32, F49H12.4, cpz-2, F49E12.8, dyf-6, ZK616.1, pdl-1, pcp-3, arl-3, ceph-41
 gcy-35, F58B4.2, T04C12.9, C27A2.8, Y16B4A.2, mksr-2, dylt-2, ctsa-2, ifta-2, T01D3.1
PC_ 3
Positive: col-103, col-94, nlp-33, ttr-15, sams-1, nlp-29, col-42, col-98, ram-2, col-3
 pmt-2, lips-10, cest-24, brp-1, nlp-24, col-166, srap-1, col-39, col-10, col-147
 col-107, F17H10.2, ttr-18, F46F2.3, hil-2, F18E3.13, msa-1, col-160, col-117, qdpr-1
Negative: cpn-3, B0379.1, ost-1, Y53H1B.2, D2092.4, clik-1, ttr-16, pqn-48, unc-15, D1086.5
 let-2, Y71H2B.4, mup-2, EGAP4.1, pat-10, unc-27, R13H4.2, tnt-2, unc-87, Y37D8A.2
 T10G3.1, tsp-8, pqn-24, T04A6.1, C18B2.3, D2063.1, txt-14, spp-15, gsnl-1, Y45F10B.13
PC_ 4
Positive: his-24, hil-2, hic-1, hil-3, lec-8, F13B6.3, F14D2.19, ZC53.22, his-32, T24C4.8
 tbx-2, thk-1, nspb-5, lin-32, mig-21, Y47A7.2, rad-51, C09D4.2, F53A9.7, C04C3.9
 mec-7, F32H5.3, M02H5.8, F46H5.3, T20B5.2, nspb-1, R05H11.2, txt-9, C43D7.8, C16B8.3
Negative: nlp-28, hgo-1, K07E1.1, C35C5.9, cnc-8, col-160, far-7, fip-5, nlp-33, F42A8.1
 col-91, F25E5.8, col-80, col-147, nlp-27, pah-1, F15E6.3, cth-2, F23F12.12, T27D12.1
 cest-24, col-166, haly-1, pmt-2, T10H9.8, col-94, B0410.3, col-39, F16C3.2, H40L08.2
PC_ 5
Positive: F21D5.3, K10D11.6, tag-10, Y32F6A.5, pmp-1, F09A5.2, ugt-18, ugt-44, mct-4, slc-17.3
 ctsa-4.1, K10D11.3, clec-166, clec-42, folt-2, acox-1.5, asah-1, ctsa-1.2, clec-160, Y32F6A.4
 mfsd-8, ceh-37, clec-80, clec-41, crn-6, T01D3.6, acd-5, ent-4, C14C11.4, oac-14
Negative: irg-7, T25C12.3, F14D2.19, F13B6.3, C12D12.1, ZC53.22, nspg-7.1, T24C4.8, nspg-14, F53A9.7
 hpo-6, C16B8.3, nspg-10, WBGene00018194, pbo-4, nspb-5, F35E12.6, Y46H3A.4, T25D10.1, K08D12.6
 T28F3.8, nspg-9, Y39B6A.1, F57F4.4, C04C3.9, nspg-13, mec-17, Y75B12B.3, lipl-2, gfi-1

ElbowPlot(OP50.2, ndims = 50)


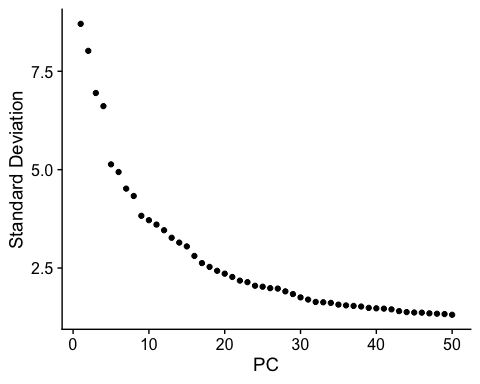


OP50.2 <- FindNeighbors(OP50.2, dims = 1:32, reduction = "pca")

Computing nearest neighbor graph

Computing SNN

OP50.2 <- FindClusters(OP50.2, resolution = 1.0, cluster.name = "OP50.2.unintegrated_clusters")

Modularity Optimizer version 1.3.0 by Ludo Waltman and Nees Jan van Eck

Number of nodes: 7092
Number of edges: 285882

Running Louvain algorithm...
Maximum modularity in 10 random starts: 0.9172
Number of communities: 28
Elapsed time: 0 seconds

OP50.2 <- RunUMAP(OP50.2, dims = 1:32, reduction = "pca", reduction.name = "umap.OP50.2.unintegrated")

11:11:41 UMAP embedding parameters a = 0.9922 b = 1.112

11:11:41 Read 7092 rows and found 32 numeric columns

11:11:41 Using Annoy for neighbor search, n_neighbors = 30

11:11:41 Building Annoy index with metric = cosine, n_trees = 50

0% 10 20 30 40 50 60 70 80 90 100%

[----|----|----|----|----|----|----|----|----|----|

**************************************************|
11:11:41 Writing NN index file to temp file /var/folders/2_/0b7d0hy11bd2g2nl32tfghfh981cxm/T//RtmpZVhw0e/file18f3391b2f3
11:11:41 Searching Annoy index using 1 thread, search_k = 3000
11:11:43 Annoy recall = 100%
11:11:44 Commencing smooth kNN distance calibration using 1 thread with target n_neighbors = 30
11:11:46 Initializing from normalized Laplacian + noise (using RSpectra)
11:11:46 Commencing optimization for 500 epochs, with 311176 positive edges
11:11:46 Using rng type: pcg
11:11:55 Optimization finished

DimPlot(OP50.2, reduction = "umap.OP50.2.unintegrated", label = T)


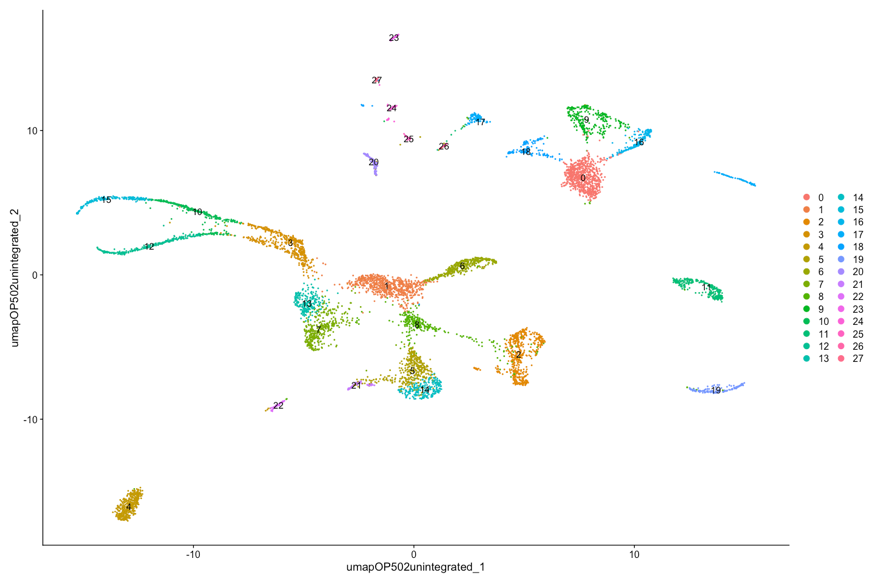


FeaturePlot(OP50.2, reduction = "umap.OP50.2.unintegrated",
 features = c("mig-21","mab-5", "lin-39", "gcy-32", "mec-7", "ajm-1", "mec-3", "ast-1"),
 label = F, order = T, cols = c("#efefef", "#4011ba"), ncol = 4)


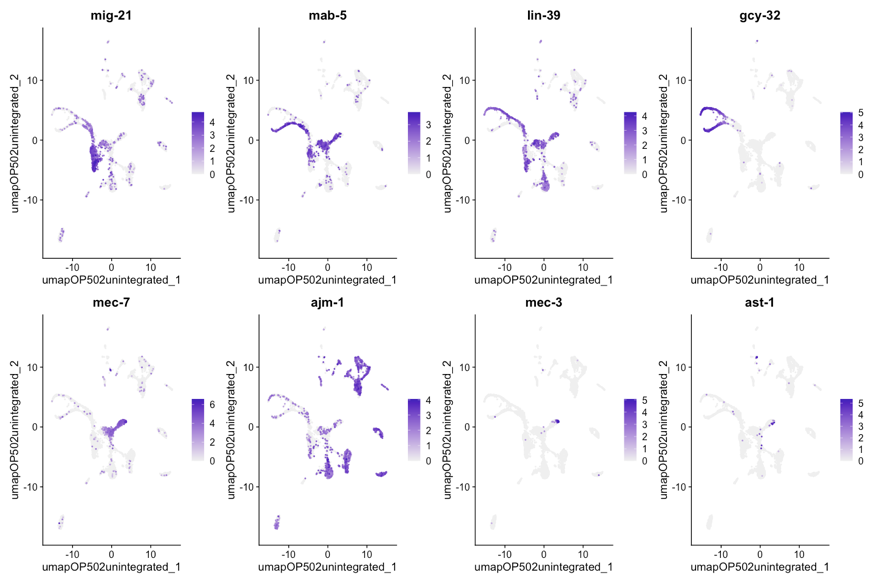


FeaturePlot(OP50.2, reduction = "umap.OP50.2.unintegrated",
 features = c("nCount_RNA","percent.mt"),
 label = F, order = T, cols = c("#efefef", "#4011ba"))


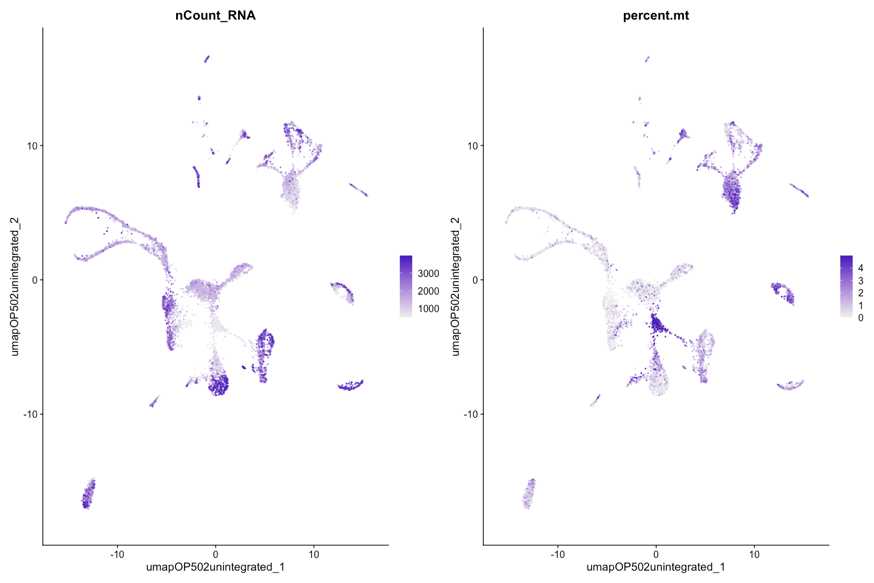


### 1st round of cleaning - removing non-Q cells

OP50.2.extra_cells_to_remove.OP50.2 <- WhichCells(OP50.2, idents = c("0", "2", "4", "5", "8", "9", "11", "14","16","17","18", "19", "20", "21", "21", "23", "24", "25", "26", "27"))
OP50.2_v2 <- subset(OP50.2, cells = setdiff(Cells(OP50.2), OP50.2.extra_cells_to_remove.OP50.2))

OP50.2_v2 <- NormalizeData(OP50.2_v2)

Normalizing layer: counts

OP50.2_v2 <- FindVariableFeatures(OP50.2_v2)

Finding variable features for layer counts

cele_features <- rownames(OP50.2_v2)
OP50.2_v2 <- ScaleData(OP50.2_v2, features = cele_features)

Centering and scaling data matrix

OP50.2_v2 <- RunPCA(OP50.2_v2)

PC_ 1
Positive: gcy-37, mks-2, gcy-32, F49H12.4, dyf-6, F49E12.8, arl-3, pdl-1, C27A2.8, gcy-35
 ceph-41, T04C12.9, sbt-1, F58B4.2, mksr-2, daf-25, dylt-2, T01D3.1, ifta-2, fmi-1
 ift-81, K07C11.10, arcp-1, tmem-231, gcy-36, tmem-218, nphp-1, mksr-1, egl-21, ptb-1
Negative: his-24, rpl-7A, rpl-17, rnr-1, hil-2, hil-3, calu-1, C50F4.6, eef-1G, rpl-20
 rps-8, F46H5.3, rps-0, rpl-3, rpl-32, rps-23, rps-1, cyd-1, R05H5.3, efhd-1
 mcm-7, mig-21, C30F12.5, rps-14, ctf-8, rps-4, rps-6, rps-7, pcn-1, rpl-33
PC_ 2
Positive: glp-1, Y54E2A.8, wht-2, msh-5, R11A8.1, B0304.4, T05F1.2, glh-2, pgl-1, F22D6.9
 pid-1, glh-1, T28D9.4, inx-14, tofu-2, T10B11.8, K09H9.7, ife-5, clec-87, mans-4
 M116.5, bath-36, sip-1, B0001.2, E02H9.3, Y49E10.4, C08F11.7, Y17G9B.4, spo-11, ego-1
Negative: hil-2, his-24, C50F4.6, calu-1, hil-3, F46H5.3, pkd-2, tbx-2, lin-32, Y7A9D.1
 shc-2, F41C3.7, F07C6.4, his-60, C04A11.2, gst-7, rnr-1, lipl-7, WBGene00023302, ham-1
 his-32, C09D4.2, lgc-27, Y71F9AL.6, T04F8.9, C43D7.8, ssq-1, cki-1, daf-19, dsh-2
PC_ 3
Positive: dma-1, WBGene00023302, F07C6.4, daf-19, mec-7, shc-2, hlh-14, ssq-1, ham-1, C04A11.2
 F33A8.7, Y71F9AL.6, C08F1.10, pkd-2, eat-20, C40A11.6, cki-1, C03C10.5, lipl-7, F44E5.1
 pat-2, T01D3.3, F58E2.5, F23B12.4, atf-2, mig-13, F39B2.3, tag-275, tbca-1, F48E3.9
Negative: mig-21, unc-54, GFP, suro-1, cpn-1, egl-17, F47B7.1, F49E2.5, tsp-14, pde-1
 rps-2, gcy-33, W07A12.4, rpl-11.2, rpl-3, emb-9, flp-21, rps-1, rps-6, rpl-31
 mcm-2, rpl-22, rps-4, bar-1, rpl-33, rps-25, C30F12.5, rpl-30, rla-0, W01D2.1
PC_ 4
Positive: mec-7, his-24, hlh-14, F33A8.7, pde-1, nex-4, flp-21, sem-4, gcy-33, nlp-43
 hil-3, WBGene00044308, ins-18, C08G9.1, pgal-1, flp-14, snt-4, myrf-1, aexr-2, T05A8.3
 unc-68, tbca-1, M01H9.4, nphp-2, zag-1, hphd-1, hil-2, F32H5.3, F58F9.1, Y43B11AR.1
Negative: lin-32, Y7A9D.1, unc-54, che-2, T01D3.3, C03C10.5, F48E3.9, daf-19, W07A12.4, K09F6.13
 ham-1, gst-7, C04A11.2, mig-21, jbts-14, mks-5, GFP, WBGene00023302, pkd-2, ccep-290
 gcy-36, R07E5.17, ssq-1, egl-13, egl-17, tctn-1, dma-1, nphp-1, msa-1, suro-1
PC_ 5
Positive: mec-7, hlh-14, mig-1, F33A8.7, tbca-1, Y51H4A.1, unc-68, far-1, nid-1, sem-4
 M01H9.4, mec-3, T01D3.3, C09D4.2, zag-1, myrf-1, C03A3.1, cmk-1, bkip-1, atf-2
 unc-24, C03C10.5, ric-19, fbxa-137, C33A12.4, fmil-1, C01C4.3, lbp-3, pag-3, arrd-25
Negative: lipl-7, tbx-2, lgc-27, Y54H5A.2, F07C6.4, C43D7.8, ham-1, lin-32, pkd-2, srd-30
 srd-29, Y71F9AL.6, egl-13, ssq-1, lgc-52, WBGene00023302, Y7A9D.1, F58H1.7, rgl-1, C04A11.2
 daf-19, hil-2, eat-20, gst-7, F41C3.7, rad-51, gcy-36, dsh-2, exc-9, magi-1

ElbowPlot(OP50.2_v2, ndims = 50)


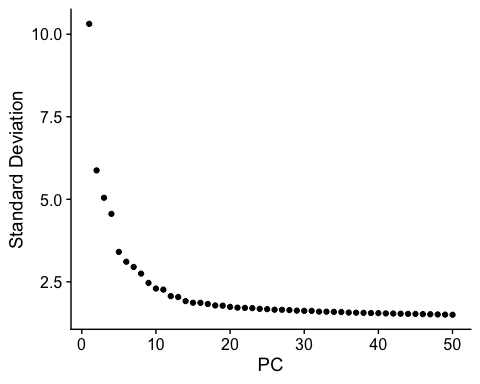


OP50.2_v2 <- FindNeighbors(OP50.2_v2, dims = 1:14, reduction = "pca")

Computing nearest neighbor graph

Computing SNN

OP50.2_v2 <- FindClusters(OP50.2_v2, resolution = 2.0, cluster.name = "OP50.2.unintegrated_clusters")

Modularity Optimizer version 1.3.0 by Ludo Waltman and Nees Jan van Eck

Number of nodes: 2942
Number of edges: 99509

Running Louvain algorithm...
Maximum modularity in 10 random starts: 0.7946
Number of communities: 20
Elapsed time: 0 seconds

OP50.2_v2 <- RunUMAP(OP50.2_v2, dims = 1:14, reduction = "pca", reduction.name = "umap.OP50.2.unintegrated")

11:12:10 UMAP embedding parameters a = 0.9922 b = 1.112

11:12:10 Read 2942 rows and found 14 numeric columns

11:12:10 Using Annoy for neighbor search, n_neighbors = 30

11:12:10 Building Annoy index with metric = cosine, n_trees = 50

0% 10 20 30 40 50 60 70 80 90 100%

[----|----|----|----|----|----|----|----|----|----|

**************************************************|
11:12:10 Writing NN index file to temp file /var/folders/2_/0b7d0hy11bd2g2nl32tfghfh981cxm/T//RtmpZVhw0e/file18f330121ebf
11:12:10 Searching Annoy index using 1 thread, search_k = 3000
11:12:11 Annoy recall = 100%
11:12:12 Commencing smooth kNN distance calibration using 1 thread with target n_neighbors = 30
11:12:14 Initializing from normalized Laplacian + noise (using RSpectra)
11:12:14 Commencing optimization for 500 epochs, with 115284 positive edges
11:12:14 Using rng type: pcg
11:12:18 Optimization finished

DimPlot(OP50.2_v2, reduction = "umap.OP50.2.unintegrated", label = T) + FeaturePlot(OP50.2_v2, reduction = "umap.OP50.2.unintegrated",
 features = c("nCount_RNA"),
 label = F, order = T, cols = c("#efefef", "#4011ba"))


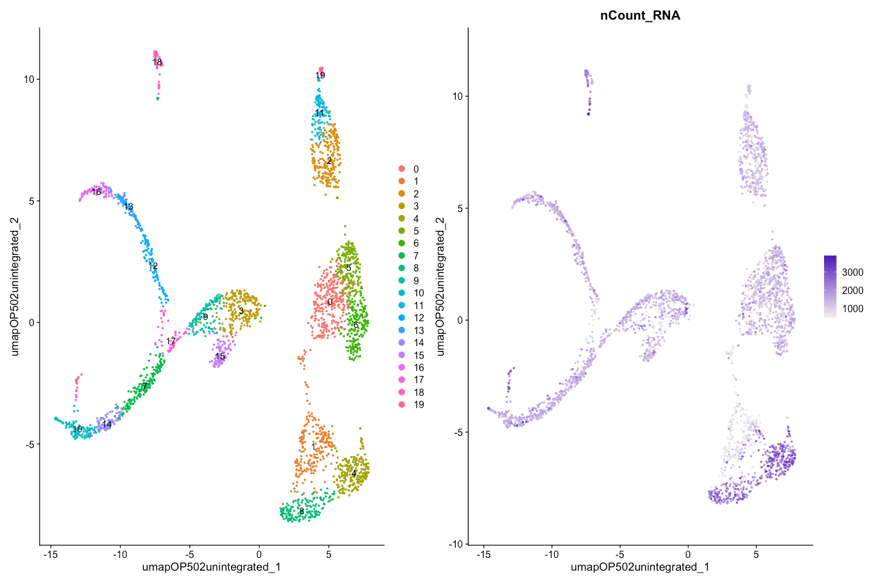


FeaturePlot(OP50.2_v2, reduction = "umap.OP50.2.unintegrated",
 features = c("mig-21","mab-5", "lin-39", "gcy-32", "mec-7", "ajm-1", "mec-3","ast-1"),
 label = F, order = T, cols = c("#efefef", "#4011ba"), ncol = 4)


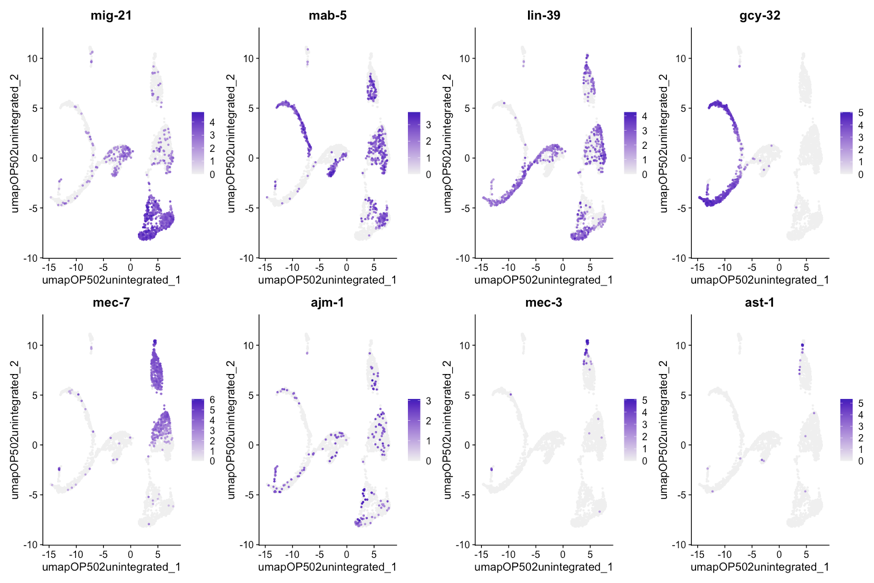


### 2nd round of cleaning - cluster-specific QC

Note: Qx.pax has a low RNA content compared to their parents, which would put them under the low-quality threshold. To avoid removing them, we identified in which cluster they are in and worked around them for this QC step. This was not an issue in the previous experiments because they had a lower complexity.

DimPlot(OP50.2_v2, reduction = "umap.OP50.2.unintegrated", label = T) + VlnPlot(OP50.2_v2, features = "nCount_RNA")


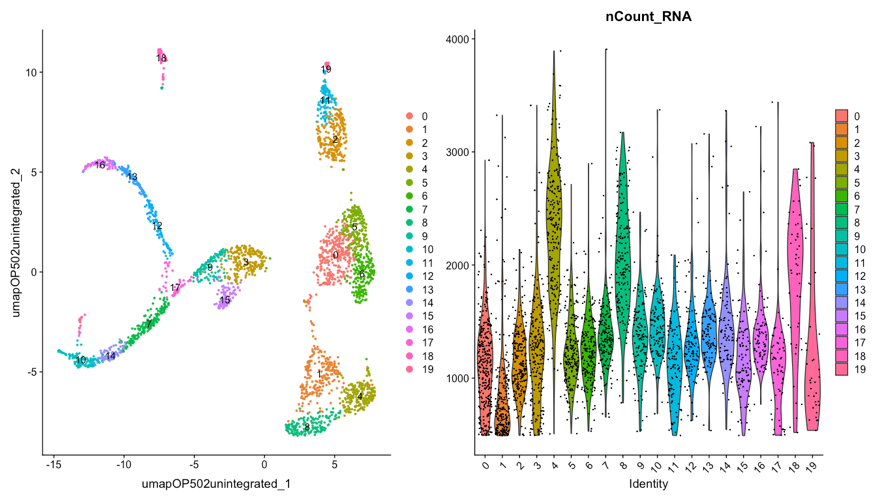


FeaturePlot(OP50.2_v2, reduction = "umap.OP50.2.unintegrated",
 features = c("mec-3","ast-1"),
 label = F, order = T, cols = c("#efefef", "#4011ba"), ncol = 2)


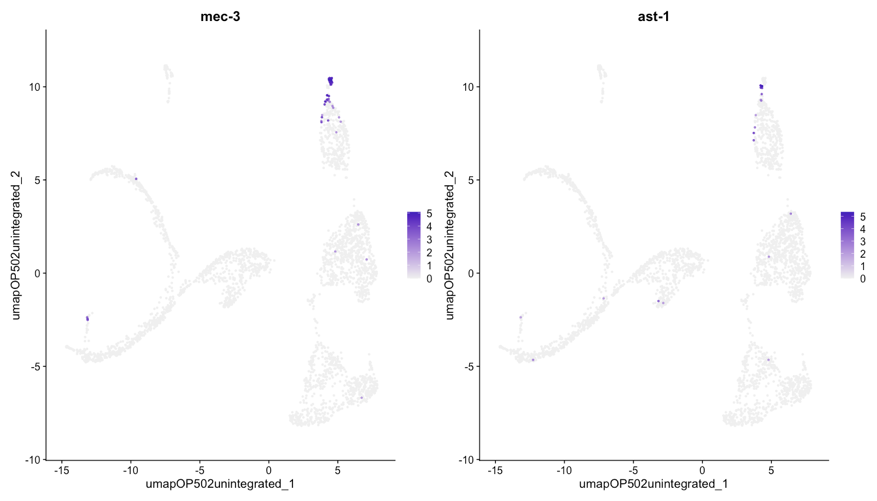


FeaturePlot(OP50.2_v2, reduction = "umap.OP50.2.unintegrated", features = c("nCount_RNA"), label = F, order = T) + scale_color_gradientn(colors = c("black", "black", "yellow", "yellow","red", "red", "red", "blue", "blue", "blue", "blue", "blue", "blue", "blue", "blue", "blue"))

Scale for colour is already present.
Adding another scale for colour, which will replace the existing scale.


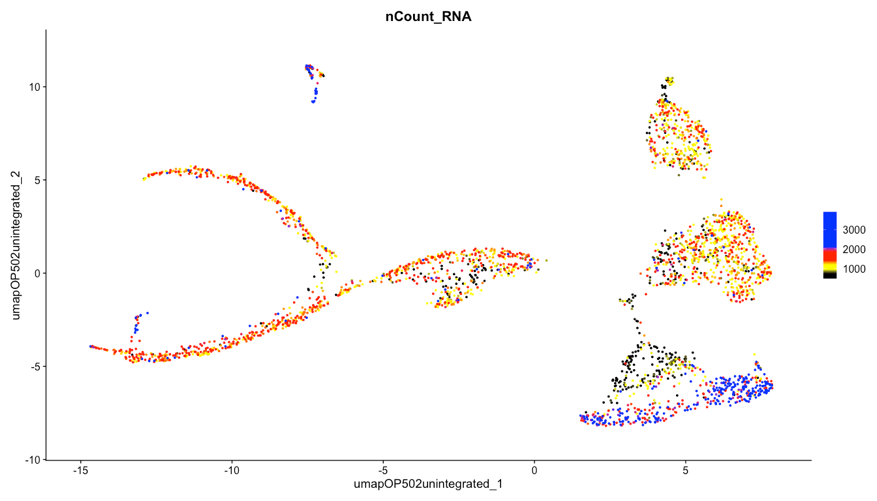


OP50.2.Q_to_remove <- WhichCells(OP50.2_v2, idents = c("1", "4", "8"), expression = nCount_RNA < 1300)
OP50.2.Q.d_to_remove_up <- WhichCells(OP50.2_v2, idents = c("0","2", "3", "5", "6", "7", "9", "10", "11", "12", "13","14", "15", "16", "17", "19"), expression = nCount_RNA > 1900)
OP50.2.Q.d_to_remove_down <- WhichCells(OP50.2_v2, idents = c("0","2", "3", "5", "6", "7", "9", "10", "12", "13","14", "15", "16", "17"), expression = nCount_RNA < 550)
OP50.2.Q.d_to_remove_down_2 <- WhichCells(OP50.2_v2, idents = c("19"), expression = nCount_RNA > 1400)
OP50.2.extra_cells_to_remove.OP50.2_v2 <- WhichCells(OP50.2_v2, idents = c("18"))


OP50.2.Q_cells_to_remove <- c(OP50.2.Q_to_remove, OP50.2.Q.d_to_remove_up, OP50.2.Q.d_to_remove_down, OP50.2.Q.d_to_remove_down_2
 , OP50.2.extra_cells_to_remove.OP50.2_v2
 )

OP50.2_v3 <- subset(OP50.2_v2, cells = setdiff(Cells(OP50.2_v2), OP50.2.Q_cells_to_remove))

VlnPlot(OP50.2_v3, features = "nCount_RNA")


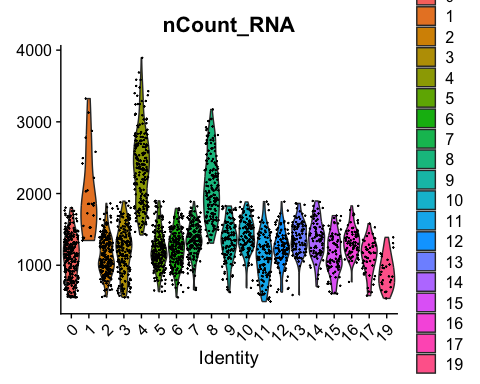


OP50.2_v3 <- NormalizeData(OP50.2_v3)

Normalizing layer: counts

OP50.2_v3 <- FindVariableFeatures(OP50.2_v3)

Finding variable features for layer counts

cele_features <- rownames(OP50.2_v3)
OP50.2_v3 <- ScaleData(OP50.2_v3, features = cele_features)

Centering and scaling data matrix

OP50.2_v3 <- RunPCA(OP50.2_v3)

PC_ 1
Positive: gcy-37, mks-2, gcy-32, C27A2.8, F49H12.4, arl-3, pdl-1, F49E12.8, gcy-35, ZK616.1
 T04C12.9, sbt-1, F58B4.2, daf-25, dylt-2, T01D3.1, Y44A6D.2, fmi-1, ifta-2, K07C11.10
 ift-81, arcp-1, nphp-1, tmem-218, mksr-1, ptb-1, egl-21, T02G5.3, gcy-34, F01E11.3
Negative: his-24, rpl-7A, hil-2, rnr-1, rpl-17, eef-1A.1, hil-3, C50F4.6, calu-1, rpl-41.2
 rps-18, eef-1B.1, eef-1G, F46H5.3, rpl-32, cdl-1, R05H5.3, rpl-20, rps-0, rps-9
 C30F12.5, rps-23, rps-5, mcm-5, R05H11.2, mcm-7, rpl-5, rpl-13, rps-14, rpl-43
PC_ 2
Positive: unc-54, R05H11.2, cpn-1, egl-17, suro-1, F47B7.1, T25G12.3, W07A12.4, tsp-14, mcm-6
 bar-1, emb-9, F49E2.5, F14H12.8, R05D3.9, rps-2, WBGene00022730, W01D2.1, rpl-15, rpl-31
 T23F2.5, clp-1, rps-22, rps-6, rpl-22, ztf-16, rpl-33, rpl-11.2, M60.4, rpl-3
Negative: egl-46, mec-7, hlh-14, F33A8.7, shc-2, his-24, F07C6.4, rhgf-2, hil-2, C08F1.10
 dma-1, tbca-1, C40A11.6, WBGene00023302, sem-4, unc-68, Y71F9AL.6, C09D4.2, syd-9, E01G4.5
 hum-5, F39B2.3, lipl-7, tsp-7, atf-2, cki-1, lgc-27, WBGene00018813, ssq-1, hil-3
PC_ 3
Positive: Y7A9D.1, lin-32, daf-19, ham-1, C04A11.2, WBGene00023302, T01D3.3, F48E3.9, C03C10.5, pkd-2
 gst-7, che-2, jbts-14, ssq-1, K09F6.13, egl-13, dma-1, unc-54, mks-5, ccep-290
 syd-9, R07E5.17, Y71F9AL.6, eat-20, F23B12.4, tctn-1, fmi-1, gasr-8, lipl-7, dsh-2
Negative: mec-7, pde-1, flp-21, nex-4, gcy-33, his-24, ins-18, nlp-43, pgal-1, flp-14
 WBGene00044308, C08G9.1, snt-4, hlh-14, hil-3, aexr-2, hphd-1, sem-4, T05A8.3, nphp-2
 myrf-1, F33A8.7, F32H5.3, Y17D7B.10, lact-1, M01H9.4, ilcr-1, B0205.14, F58F9.1, hot-3
PC_ 4
Positive: tbx-2, lipl-7, lgc-27, Y54H5A.2, F07C6.4, C43D7.8, ham-1, lin-32, srd-30, pkd-2
 Y71F9AL.6, egl-13, srd-29, F58H1.7, lgc-52, Y7A9D.1, ssq-1, WBGene00023302, rgl-1, hil-2
 cutl-16, ZK131.11, C04A11.2, T01B10.5, daf-19, F58D2.2, nex-4, eat-20, F41C3.7, egl-46
Negative: mec-7, hlh-14, mig-1, F33A8.7, tbca-1, Y51H4A.1, T01D3.3, tsp-7, unc-68, sem-4
 hum-5, far-1, C03C10.5, C09D4.2, zag-1, F44E5.1, myrf-1, mec-3, fmil-1, cmk-1
 C03A3.1, bkip-1, M01H9.4, atf-2, ric-19, fbxa-137, K09F6.13, pag-3, C06E7.2, mksr-1
PC_ 5
Positive: F32H5.3, hphd-1, Y47A7.2, cdl-1, mcm-7, ctf-8, shc-2, thk-1, pezo-1, T24C4.2
 tost-1, lec-4, rpa-1, mcm-3, R05H5.3, sem-2, rnr-1, mab-5, hil-3, zwl-1
 H37A05.4, hlh-3, pcn-1, mcm-5, sto-1, cdh-3, mcm-2, pid-3, T22B7.22, isw-1
Negative: hlh-14, mig-1, egl-46, C09D4.2, F33A8.7, unc-68, mec-7, tsp-7, mec-3, C40A11.6
 F14H12.8, C08F1.10, WBGene00018813, bar-1, ttr-18, C06E7.2, T04C12.11, nhr-25, fipr-21, col-167
 C34B4.2, snf-3, M60.4, Y47G6A.31, lbp-3, F36G3.1, T25G12.3, F39B2.3, nlp-24, atf-2

ElbowPlot(OP50.2_v3, ndims = 50)


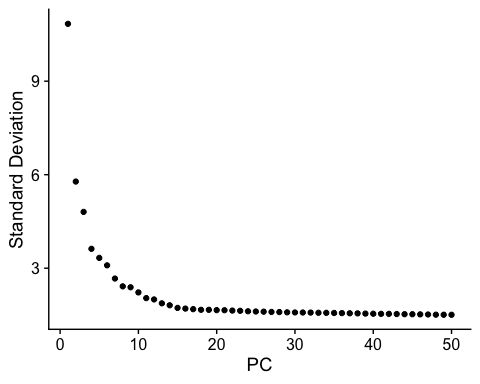


OP50.2_v3 <- FindNeighbors(OP50.2_v3, dims = 1:15, reduction = "pca")

Computing nearest neighbor graph

Computing SNN

OP50.2_v3 <- FindClusters(OP50.2_v3, resolution = 2.0, cluster.name = "OP50.2.unintegrated_clusters")

Modularity Optimizer version 1.3.0 by Ludo Waltman and Nees Jan van Eck

Number of nodes: 2519
Number of edges: 83589

Running Louvain algorithm...
Maximum modularity in 10 random starts: 0.7891
Number of communities: 21
Elapsed time: 0 seconds

OP50.2_v3 <- RunUMAP(OP50.2_v3, dims = 1:15, reduction = "pca", reduction.name = "umap.OP50.2.unintegrated")

11:12:33 UMAP embedding parameters a = 0.9922 b = 1.112

11:12:33 Read 2519 rows and found 15 numeric columns

11:12:33 Using Annoy for neighbor search, n_neighbors = 30

11:12:33 Building Annoy index with metric = cosine, n_trees = 50

0% 10 20 30 40 50 60 70 80 90 100%

[----|----|----|----|----|----|----|----|----|----|

**************************************************|
11:12:33 Writing NN index file to temp file /var/folders/2_/0b7d0hy11bd2g2nl32tfghfh981cxm/T//RtmpZVhw0e/file18f312dd1599
11:12:33 Searching Annoy index using 1 thread, search_k = 3000
11:12:33 Annoy recall = 100%
11:12:34 Commencing smooth kNN distance calibration using 1 thread with target n_neighbors = 30
11:12:36 Initializing from normalized Laplacian + noise (using RSpectra)
11:12:36 Commencing optimization for 500 epochs, with 97080 positive edges
11:12:36 Using rng type: pcg
11:12:40 Optimization finished

DimPlot(OP50.2_v3, reduction = "umap.OP50.2.unintegrated", label = T) + FeaturePlot(OP50.2_v3, reduction = "umap.OP50.2.unintegrated",
 features = c("nCount_RNA"),
 label = F, order = T, cols = c("#efefef", "#4011ba"))


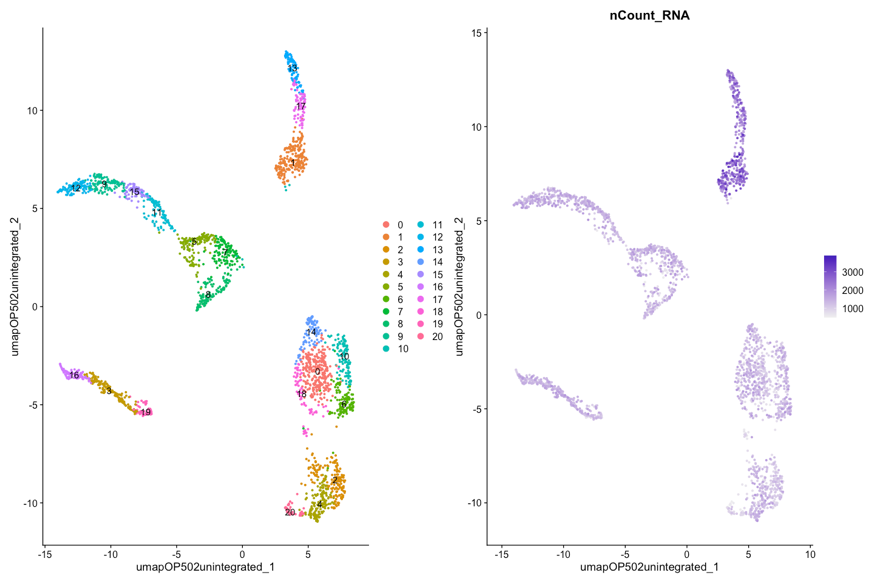


FeaturePlot(OP50.2_v3, reduction = "umap.OP50.2.unintegrated",
 features = c("mig-21","mab-5", "lin-39", "gcy-32", "mec-7", "ajm-1", "mec-3","ast-1"),
 label = F, order = T, cols = c("#efefef", "#4011ba"), ncol = 4)


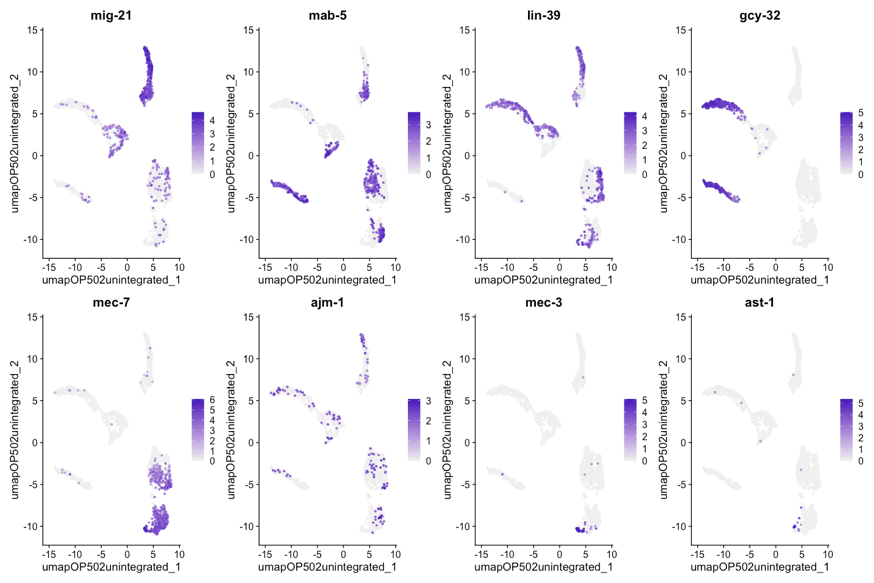


### 3rd round of cleaning - cluster-specific QC_v2

Note: An extra round of QC was performed to improve cleaning after working around Qx.pax

DimPlot(OP50.2_v3, reduction = "umap.OP50.2.unintegrated", label = T) + VlnPlot(OP50.2_v3, features = "nCount_RNA")


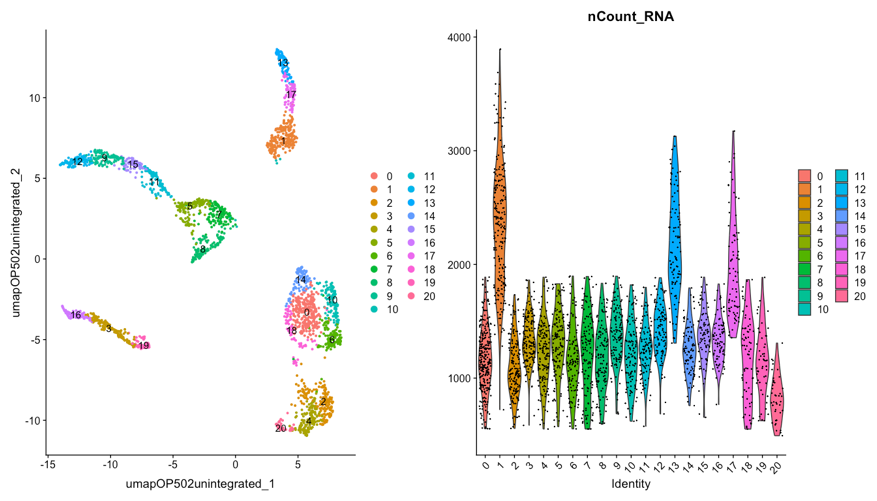


FeaturePlot(OP50.2_v3, reduction = "umap.OP50.2.unintegrated", features = c("nCount_RNA"), label = F, order = T) + scale_color_gradientn(colors = c("black", "black", "yellow", "yellow","red", "red", "red", "blue", "blue", "blue", "blue", "blue", "blue", "blue", "green", "green"))

Scale for colour is already present.
Adding another scale for colour, which will replace the existing scale.


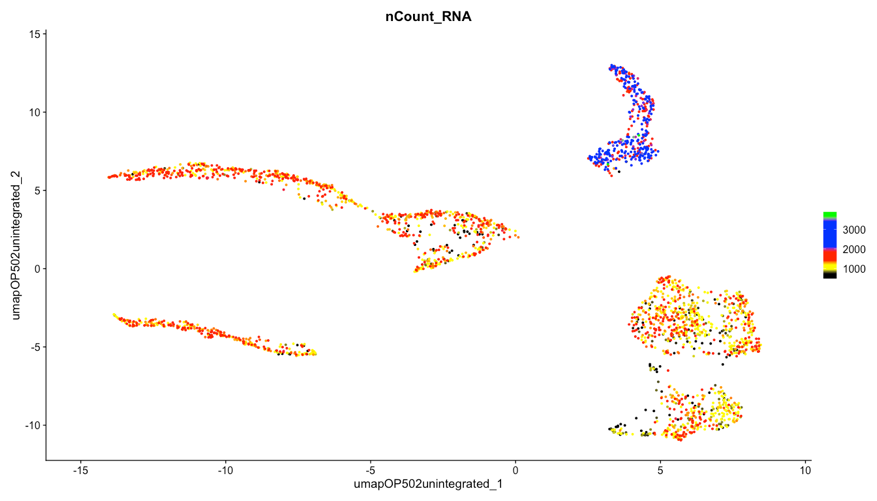


OP50.2.Q.ap_to_remove <- WhichCells(OP50.2_v3, idents = c("3","5", "7", "8", "9", "11", "12", "15", "16", "19"), expression = nCount_RNA < 700)
OP50.2.Q.p_to_remove <- WhichCells(OP50.2_v3, idents = c("0","6","10","14","18"), expression = nCount_RNA < 700)
OP50.2.Q.pax_to_remove <- WhichCells(OP50.2_v3, idents = c("20"), expression = nCount_RNA > 1300)


OP50.2.Q_cells_to_remove2 <- c(OP50.2.Q.ap_to_remove, OP50.2.Q.p_to_remove, OP50.2.Q.pax_to_remove)

OP50.2_v4 <- subset(OP50.2_v3, cells = setdiff(Cells(OP50.2_v3), OP50.2.Q_cells_to_remove2))

VlnPlot(OP50.2_v3, features = "nCount_RNA")


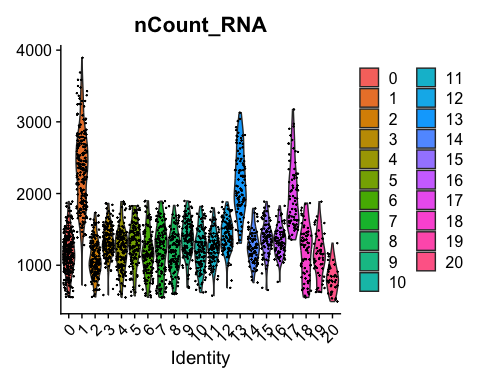


VlnPlot(OP50.2_v4, features = "nCount_RNA")


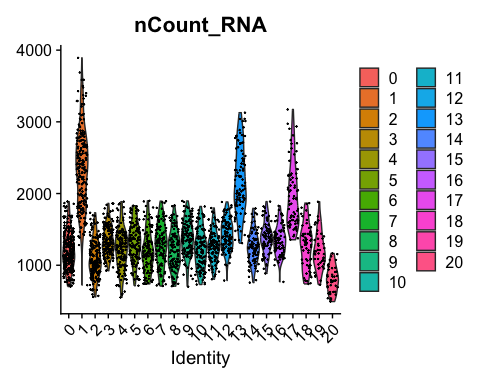


OP50.2_v4 <- NormalizeData(OP50.2_v4)

Normalizing layer: counts

OP50.2_v4 <- FindVariableFeatures(OP50.2_v4)

Finding variable features for layer counts

cele_features <- rownames(OP50.2_v4)
OP50.2_v4 <- ScaleData(OP50.2_v4, features = cele_features)

Centering and scaling data matrix

OP50.2_v4 <- RunPCA(OP50.2_v4)

PC_ 1
Positive: gcy-37, mks-2, gcy-32, dyf-6, F49H12.4, arl-3, gcy-35, F49E12.8, ZK616.1, ceph-41
 T04C12.9, sbt-1, F58B4.2, mksr-2, daf-25, dylt-2, fmi-1, Y44A6D.2, T01D3.1, ifta-2
 tmem-231, K07C11.10, ift-81, arcp-1, gcy-36, nphp-1, tmem-218, mksr-1, ptb-1, egl-21
Negative: his-24, eef-2, rpl-7A, rnr-1, hil-2, rpl-17, eef-1A.1, C50F4.6, aldo-2, hil-3
 calu-1, rpl-41.2, rps-18, eef-1B.1, eef-1G, F46H5.3, efhd-1, rpl-32, rps-8, cdl-1
 rpl-20, rps-23, rps-0, C30F12.5, rps-5, mig-21, mcm-5, R05H11.2, rpl-43, mcm-7
PC_ 2
Positive: egl-46, mec-7, hlh-14, F33A8.7, his-24, shc-2, F07C6.4, hil-2, C08F1.10, tbca-1
 C40A11.6, WBGene00023302, unc-68, C09D4.2, nid-1, hum-5, E01G4.5, syd-9, F39B2.3, tsp-7
 atf-2, hil-3, cki-1, lipl-7, smp-1, ssq-1, C01C4.3, lgc-27, pat-2, C04A11.2
Negative: mig-21, unc-54, R05H11.2, cpn-1, suro-1, egl-17, F47B7.1, T25G12.3, W07A12.4, mcm-6
 bar-1, emb-9, R05D3.9, F14H12.8, F49E2.5, rps-2, clp-1, rpl-31, rps-6, rpl-15
 T23F2.5, rpl-22, M60.4, rpl-30, rpl-11.2, rpl-33, rpl-3, rla-0, ztf-16, rps-1
PC_ 3
Positive: Y7A9D.1, lin-32, daf-19, ham-1, C04A11.2, T01D3.3, C03C10.5, WBGene00023302, F48E3.9, gst-7
 pkd-2, jbts-14, ssq-1, K09F6.13, egl-13, unc-54, ccep-290, R07E5.17, gcy-36, syd-9
 F23B12.4, tctn-1, tmem-231, eat-20, gasr-8, fmi-1, dsh-2, lipl-7, nphp-1, F58E2.5
Negative: pde-1, mec-7, flp-21, nex-4, gcy-33, ins-18, nlp-43, his-24, WBGene00044308, pgal-1
 flp-14, C08G9.1, snt-4, aexr-2, T05A8.3, hlh-14, nphp-2, hil-3, ncam-1, Y17D7B.10
 F33A8.7, hphd-1, myrf-1, lact-1, F58F9.1, F32H5.3, B0205.14, ilcr-1, glb-5, hot-3
PC_ 4
Positive: tbx-2, lipl-7, Y54H5A.2, lgc-27, C43D7.8, F07C6.4, ham-1, pkd-2, lin-32, lgc-52
 egl-13, srd-29, smp-1, F58H1.7, Y7A9D.1, ssq-1, hil-2, rgl-1, WBGene00023302, C04A11.2
 cutl-16, T01B10.5, daf-19, eat-20, gst-7, T05H4.11, F41C3.7, hbl-1, dsh-2, gcy-36
Negative: mec-7, hlh-14, mig-1, F33A8.7, tbca-1, Y51H4A.1, tsp-7, unc-68, C09D4.2, nid-1
 hum-5, mec-3, far-1, C06E7.2, atf-2, T01D3.3, F44E5.1, lbp-3, F14H12.8, bar-1
 Y47G6A.31, C01C4.3, cmk-1, ric-19, zag-1, arrd-25, lpin-1, col-167, T25G12.3, lbp-1
PC_ 5
Positive: egl-46, hlh-14, C09D4.2, mig-1, F33A8.7, C08F1.10, nid-1, C40A11.6, unc-68, tsp-7
 F14H12.8, mec-3, lgc-27, cki-1, C06E7.2, syd-9, F39B2.3, bar-1, mec-7, C10A4.3
 lipl-7, prkl-1, ttr-18, F36G3.1, arrd-25, pat-2, Y47G6A.31, T04C12.11, snf-3, nhr-25
Negative: cyd-1, F32H5.3, hphd-1, cdl-1, Y47A7.2, mcm-7, shc-2, pezo-1, T24C4.2, tost-1
 grl-10, mab-5, mcm-3, rgs-11, rpa-1, thk-1, lec-4, H37A05.4, sem-2, hlh-3
 sto-1, C45G7.4, pcn-1, mcm-2, cdt-1, mcm-5, Y43B11AR.1, rnr-1, T22B7.22, zwl-1

ElbowPlot(OP50.2_v4, ndims = 50)


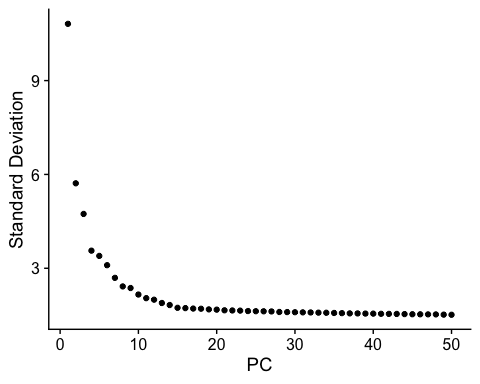


OP50.2_v4 <- FindNeighbors(OP50.2_v4, dims = 1:15, reduction = "pca")

Computing nearest neighbor graph

Computing SNN

OP50.2_v4 <- FindClusters(OP50.2_v4, resolution = 1.6, cluster.name = "OP50.2.unintegrated_clusters")

Modularity Optimizer version 1.3.0 by Ludo Waltman and Nees Jan van Eck

Number of nodes: 2443
Number of edges: 80938

Running Louvain algorithm...
Maximum modularity in 10 random starts: 0.8214
Number of communities: 18
Elapsed time: 0 seconds

OP50.2_v4 <- RunUMAP(OP50.2_v4, dims = 1:15, reduction = "pca", reduction.name = "umap.OP50.2.unintegrated")

11:12:54 UMAP embedding parameters a = 0.9922 b = 1.112

11:12:54 Read 2443 rows and found 15 numeric columns

11:12:54 Using Annoy for neighbor search, n_neighbors = 30

11:12:54 Building Annoy index with metric = cosine, n_trees = 50

0% 10 20 30 40 50 60 70 80 90 100%

[----|----|----|----|----|----|----|----|----|----|

**************************************************|
11:12:54 Writing NN index file to temp file /var/folders/2_/0b7d0hy11bd2g2nl32tfghfh981cxm/T//RtmpZVhw0e/file18f32379a199
11:12:54 Searching Annoy index using 1 thread, search_k = 3000
11:12:55 Annoy recall = 100%
11:12:56 Commencing smooth kNN distance calibration using 1 thread with target n_neighbors = 30
11:12:58 Initializing from normalized Laplacian + noise (using RSpectra)
11:12:58 Commencing optimization for 500 epochs, with 93902 positive edges
11:12:58 Using rng type: pcg
11:13:01 Optimization finished

DimPlot(OP50.2_v4, reduction = "umap.OP50.2.unintegrated", label = T) + FeaturePlot(OP50.2_v4, reduction = "umap.OP50.2.unintegrated",
 features = c("nCount_RNA"),
 label = F, order = T, cols = c("#efefef", "#4011ba"))


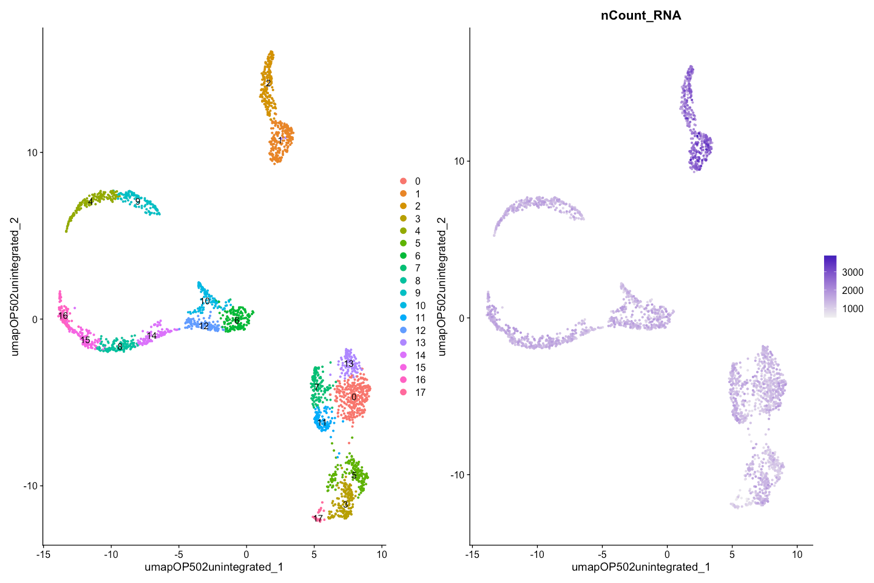


FeaturePlot(OP50.2_v4, reduction = "umap.OP50.2.unintegrated",
 features = c("mig-21","mab-5", "lin-39", "gcy-32", "mec-7", "ajm-1", "mec-3", "ast-1"),
 label = F, order = T, cols = c("#efefef", "#4011ba"), ncol = 4)


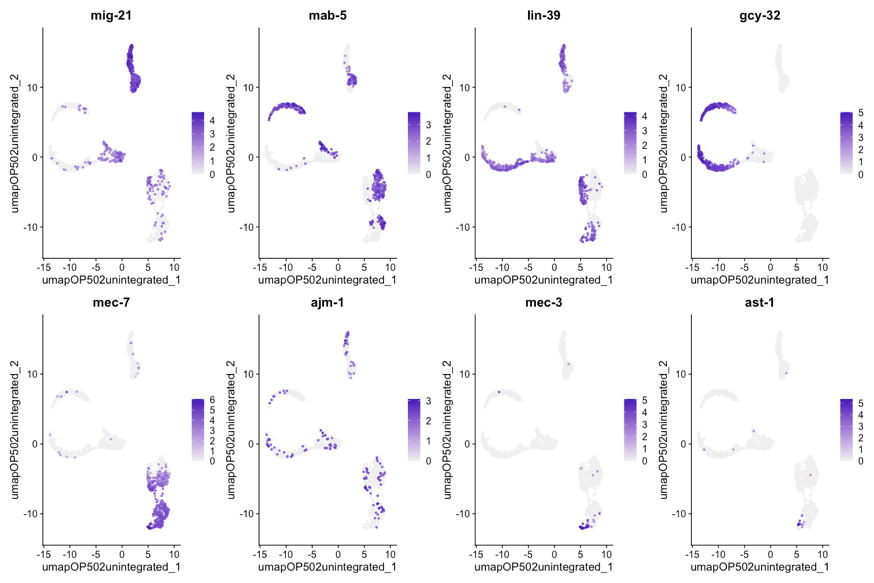


# -

# Merging unintegrated data

DimPlot(OP50_v3, reduction = "umap.OP50.unintegrated", label = T) +
DimPlot(PA14_v3, reduction = "umap.PA14.unintegrated", label = T) +
DimPlot(OP50.2_v4, reduction = "umap.OP50.2.unintegrated", label = T)


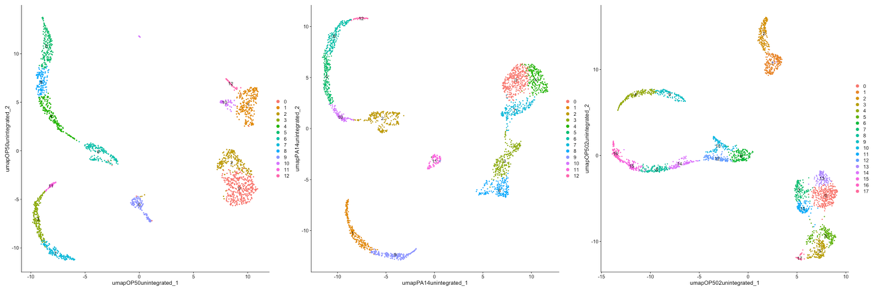


Fed_unint <- merge(x = OP50.2_v4, y = c(OP50_v3, PA14_v3), add.cell.ids = c("OP50.2", "OP50", "PA14"), project = "Fed.individual")

Fed_unint <- NormalizeData(Fed_unint)

Normalizing layer: counts.OP50.2

Normalizing layer: counts.OP50

Normalizing layer: counts.PA14

Fed_unint <- FindVariableFeatures(Fed_unint)

Finding variable features for layer counts.OP50.2

Finding variable features for layer counts.OP50

Finding variable features for layer counts.PA14

cele_features <- rownames(Fed_unint)
Fed_unint <- ScaleData(Fed_unint, features = cele_features)

Centering and scaling data matrix

Fed_unint <- RunPCA(Fed_unint)

PC_ 1
Positive: mks-2, gcy-37, C27A2.8, gcy-32, arl-3, dyf-6, pdl-1, gcy-35, F49H12.4, ZK616.1
 F58B4.2, F49E12.8, sbt-1, T04C12.9, Y44A6D.2, daf-25, gcy-36, ceph-41, fmi-1, K07C11.10
 mksr-2, T01D3.1, tmem-231, arcp-1, ifta-2, dylt-2, gcy-34, tmem-218, WBGene00014307, che-2
Negative: rpl-12, ctsa-1.1, rbm-3.2, rpl-7A, rpl-17, eef-1A.1, ZK930.6, rpl-41.2, rps-18, rpl-32
 eef-1B.1, rnr-1, his-24, aldo-2, eef-1G, rpl-36, rpl-18, rps-8, rpl-20, rps-1
 rps-5, rpl-43, rps-23, rpl-10, rps-9, C50F4.6, rps-14, hil-2, rpl-3, R05H5.3
PC_ 2
Positive: mig-21, R05H11.2, unc-54, cpn-1, suro-1, T25G12.3, F47B7.1, egl-17, WBGene00022730, emb-9
 W07A12.4, rpl-30, mcm-6, rpl-31, bar-1, rps-25, R05D3.9, tsp-14, rps-6, rla-0
 lec-4, rps-22, F49E2.5, rps-17, rla-1, ztf-16, rps-2, T23F2.5, M60.4, W01D2.1
Negative: mec-7, hlh-14, ctsa-1.1, C08F1.10, C09D4.2, nid-1, tbca-1, F44E5.1, C01C4.3, dma-1
 WBGene00018813, his-24, atf-2, R11G1.2, tsp-7, F36G3.1, unc-68, C06E7.2, F39B2.3, cank-26
 cki-1, WBGene00050903, C10A4.3, cam-1, arrd-25, F16F9.1, F07C6.4, C01B10.6, zag-1, lbp-3
PC_ 3
Positive: T01D3.3, Y7A9D.1, lin-32, C03C10.5, F48E3.9, dma-1, daf-19, unc-54, K09F6.13, ham-1
 che-2, F23B12.4, mks-5, C04A11.2, tctn-1, F58E2.5, egl-13, R01H2.8, ccep-290, ssq-1
 gasr-8, gcy-36, ift-20, mksr-1, nphp-1, tmem-231, fmi-1, W07A12.4, vab-8, M162.5
Negative: his-24, nex-4, nlp-43, flp-21, WBGene00044308, T05A8.3, mec-7, gcy-33, C08G9.1, aexr-2
 F58F9.1, pgal-1, C10C5.7, nphp-2, ampd-1, Y17D7B.10, nlp-47, flp-14, F32B4.5, die-1
 glb-1, glb-5, hlh-14, plc-1, B0205.14, F58H1.7, srg-25, F49E10.4, Y119C1B.6, lact-1
PC_ 4
Positive: F44E5.1, ssq-1, C08F1.10, lin-32, hlh-14, srd-30, Y7A9D.1, lipl-7, cam-1, flp-5
 lgc-27, cank-26, ZK131.11, lin-39, F39B2.3, cki-1, nid-1, T05A8.3, mig-1, plc-1
 nlp-43, unc-54, F36G3.1, E04F6.10, C10A4.3, C43D7.8, glna-3, die-1, nlp-47, ram-2
Negative: F32H5.3, hphd-1, cyd-1, mab-5, Y47A7.2, cdl-1, mcm-7, Y43B11AR.1, flp-4, T22B7.22
 lat-1, cutl-10, ZK742.7, H37A05.4, M162.5, ZK930.6, F35B3.7, tost-1, sto-1, cyk-7
 C33A12.4, ztf-11, sem-2, dct-14, grl-10, odr-4, gcy-34, bath-15, glb-24, fkh-5
PC_ 5
Positive: hlh-14, mig-1, mec-7, nid-1, M01H9.4, C09D4.2, tsp-7, unc-68, mec-3, C06E7.2
 F36G3.1, C01C4.3, flp-4, WBGene00018813, tbca-1, M01H9.10, far-1, bar-1, atf-2, ttr-18
 T25G12.3, ZK742.7, arrd-25, F14H12.8, F46F2.3, C08F1.10, col-107, lbp-3, F16F9.1, ram-2
Negative: ssq-1, flp-5, F07C6.4, lipl-7, srd-30, E04F6.10, lgc-27, F32H5.3, mam-8, C43D7.8
 nlp-43, glna-3, egl-13, magi-1, T05A8.3, ham-1, eat-20, plc-1, die-1, C24B5.1
 dsh-2, ZK930.6, C10C5.7, Y71F9AL.6, daf-19, C04A11.2, col-114, F23B12.4, cutl-16, fmi-1

ElbowPlot(Fed_unint, ndims = 50)


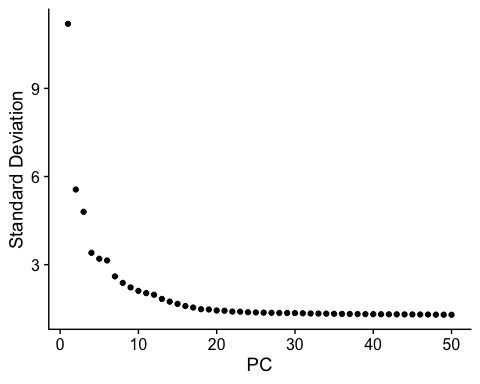


Fed_unint <- FindNeighbors(Fed_unint, dims = 1:20, reduction = "pca")

Computing nearest neighbor graph

Computing SNN

Fed_unint <- FindClusters(Fed_unint, resolution = 1.0, cluster.name = "unintegrated_clusters")

Modularity Optimizer version 1.3.0 by Ludo Waltman and Nees Jan van Eck

Number of nodes: 6822
Number of edges: 250708

Running Louvain algorithm...
Maximum modularity in 10 random starts: 0.8831
Number of communities: 18
Elapsed time: 0 seconds

Fed_unint <- RunUMAP(Fed_unint, dims = 1:20, reduction = "pca", reduction.name = "umap.unintegrated.fed")

11:13:29 UMAP embedding parameters a = 0.9922 b = 1.112

11:13:29 Read 6822 rows and found 20 numeric columns

11:13:29 Using Annoy for neighbor search, n_neighbors = 30

11:13:29 Building Annoy index with metric = cosine, n_trees = 50

0% 10 20 30 40 50 60 70 80 90 100%

[----|----|----|----|----|----|----|----|----|----|

**************************************************|
11:13:29 Writing NN index file to temp file /var/folders/2_/0b7d0hy11bd2g2nl32tfghfh981cxm/T//RtmpZVhw0e/file18f31eb9de7
11:13:29 Searching Annoy index using 1 thread, search_k = 3000
11:13:31 Annoy recall = 100%
11:13:32 Commencing smooth kNN distance calibration using 1 thread with target n_neighbors = 30
11:13:34 Initializing from normalized Laplacian + noise (using RSpectra)
11:13:34 Commencing optimization for 500 epochs, with 273288 positive edges
11:13:34 Using rng type: pcg
11:13:43 Optimization finished

DimPlot(Fed_unint, reduction = "umap.unintegrated.fed", group.by = "orig.ident",
 cols = c("#e8000d", "gold", "#0051ba")) +
 DimPlot(Fed_unint, reduction = "umap.unintegrated.fed", label = T)


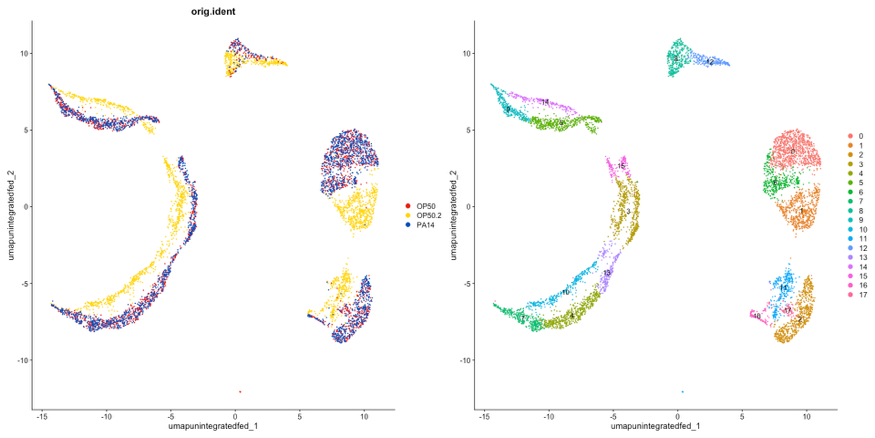


FeaturePlot(Fed_unint, reduction = "umap.unintegrated.fed",
 features = c("mig-21","mab-5", "lin-39", "gcy-32", "mec-7", "ajm-1", "mec-3", "ast-1"),
 label = F, order = T, cols = c("#efefef", "#4011ba"), ncol = 4)


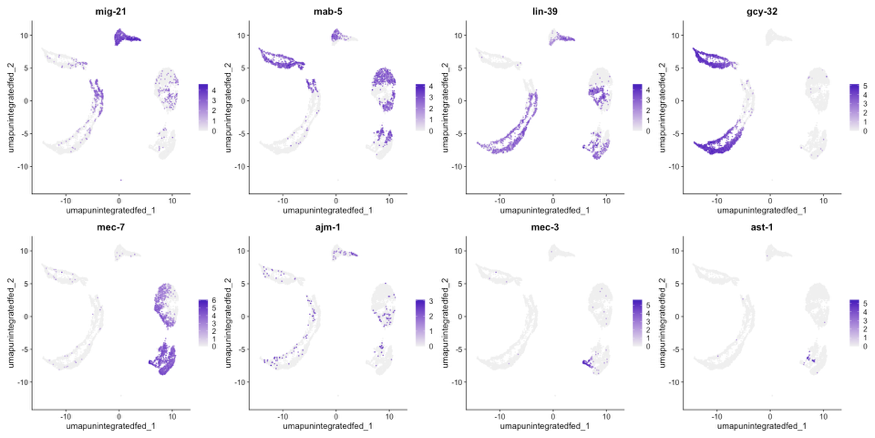


DimPlot(Fed_unint, reduction = "umap.unintegrated.fed", label = T) +
 FeaturePlot(Fed_unint, reduction = "umap.unintegrated.fed",
 features = c("nCount_RNA"),
 label = F, order = T, cols = c("#efefef", "#4011ba"))


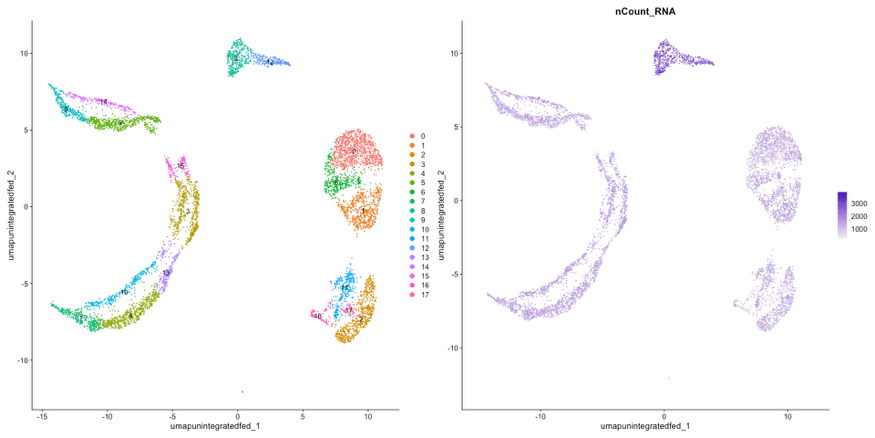


# -

# Manual cleaning

Note: Some cells with abnormal RNA count for their cluster, likely doublets (e.g., QL+QL.a) or low quality cells missed on individual QCs, might be difficult to remove with Seurat. Exporting the object and opening on Loupe browser makes it easier to remove individual cells that might skew the analysis manually. ## Integration (RPCA) for cleaning

Fed_rpca <- IntegrateLayers(
 object = Fed_unint, method = RPCAIntegration,
 orig.reduction = "pca", new.reduction = "integrated.rpca"
)

Computing within dataset neighborhoods

Finding all pairwise anchors

Warning: The `slot` argument of `GetAssayData()` is deprecated as of SeuratObject 5.0.0.
ℹ Please use the `layer` argument instead.
ℹ The deprecated feature was likely used in the Seurat package.
 Please report the issue at <https://github.com/satijalab/seurat/issues>.

Projecting new data onto SVD

Projecting new data onto SVD

Finding neighborhoods

Finding anchors

Found 1356 anchors

Projecting new data onto SVD
Projecting new data onto SVD

Finding neighborhoods

Finding anchors

Found 1389 anchors

Projecting new data onto SVD
Projecting new data onto SVD

Finding neighborhoods

Finding anchors

Found 1622 anchors

Merging dataset 2 into 3

Extracting anchors for merged samples

Finding integration vectors

Finding integration vector weights

Integrating data

Merging dataset 1 into 3 2

Extracting anchors for merged samples

Finding integration vectors

Finding integration vector weights

Integrating data

Fed_rpca[["RNA"]] <- JoinLayers(Fed_rpca[["RNA"]])

ElbowPlot(Fed_rpca , ndims = 50)


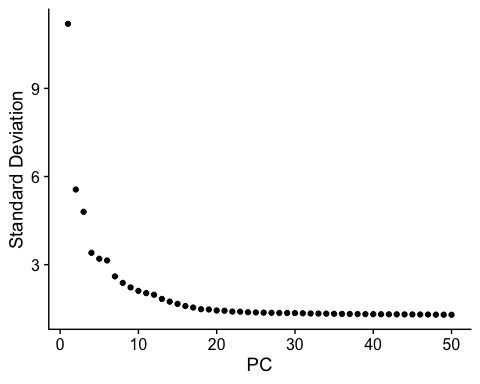


Fed_rpca <- FindNeighbors(Fed_rpca, reduction = "integrated.rpca", dims = 1:23)

Computing nearest neighbor graph

Computing SNN

Fed_rpca <- FindClusters(Fed_rpca, resolution = 1.2, cluster.name = "rpca_clusters")

Modularity Optimizer version 1.3.0 by Ludo Waltman and Nees Jan van Eck

Number of nodes: 6822
Number of edges: 286845

Running Louvain algorithm...
Maximum modularity in 10 random starts: 0.8609
Number of communities: 19
Elapsed time: 0 seconds

Fed_rpca <- RunUMAP(Fed_rpca, dims = 1:23, reduction = "integrated.rpca", reduction.name = "umap.rpca")

11:14:16 UMAP embedding parameters a = 0.9922 b = 1.112

11:14:16 Read 6822 rows and found 23 numeric columns

11:14:16 Using Annoy for neighbor search, n_neighbors = 30

11:14:16 Building Annoy index with metric = cosine, n_trees = 50

0% 10 20 30 40 50 60 70 80 90 100%

[----|----|----|----|----|----|----|----|----|----|

**************************************************|
11:14:17 Writing NN index file to temp file /var/folders/2_/0b7d0hy11bd2g2nl32tfghfh981cxm/T//RtmpZVhw0e/file18f34ffd7da7
11:14:17 Searching Annoy index using 1 thread, search_k = 3000
11:14:18 Annoy recall = 100%
11:14:19 Commencing smooth kNN distance calibration using 1 thread with target n_neighbors = 30
11:14:21 Initializing from normalized Laplacian + noise (using RSpectra)
11:14:21 Commencing optimization for 500 epochs, with 285388 positive edges
11:14:21 Using rng type: pcg
11:14:30 Optimization finished

DimPlot(Fed_rpca, reduction = "umap.rpca", label = T)


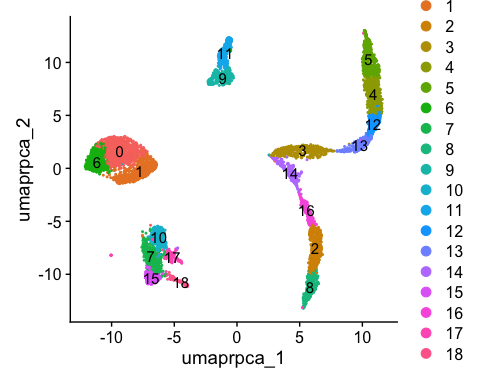


DimPlot(Fed_rpca, reduction = "umap.rpca", group.by = "orig.ident",
 cols = c("#e8000d", "gold", "#0051ba")) +
 DimPlot(Fed_rpca, reduction = "umap.rpca", label = T)


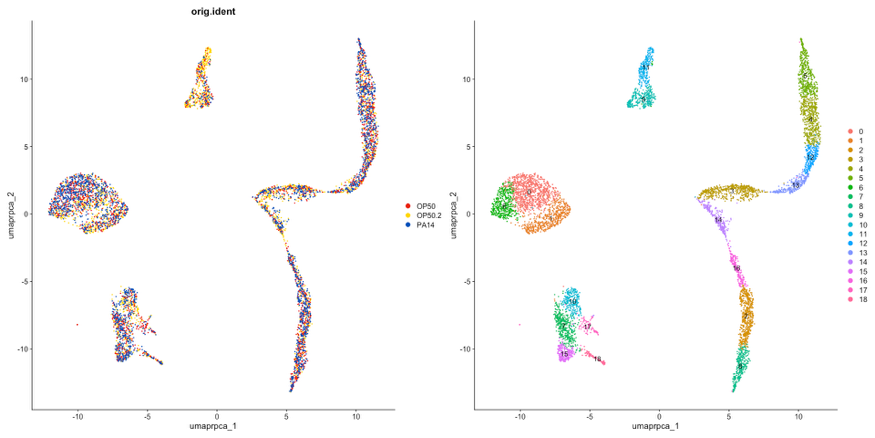


DimPlot(Fed_rpca, reduction = "umap.rpca", label = T)+FeaturePlot(Fed_rpca, reduction = "umap.rpca",
 features = c("nCount_RNA"),
 label = F, order = T, cols = c("#efefef", "#4011ba"))


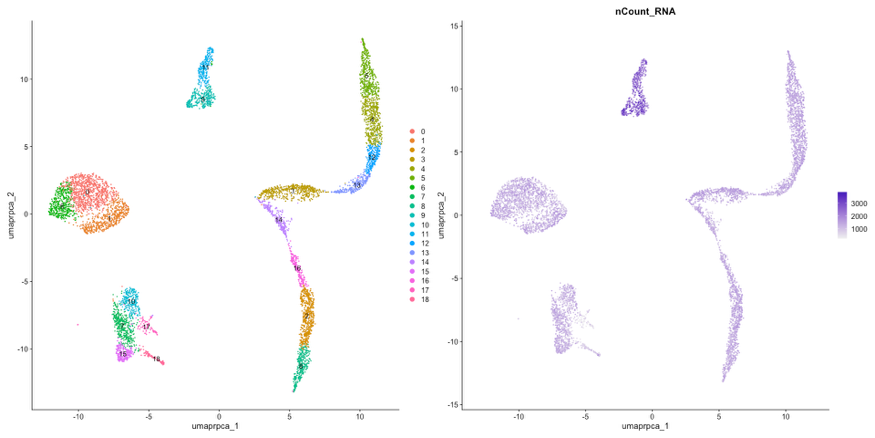


FeaturePlot(Fed_rpca, reduction = "umap.rpca",
 features = c("mig-21","mab-5", "lin-39", "mec-7", "mec-3","ast-1"),
 label = F, order = T, cols = c("#efefef", "#4011ba"), ncol = 3)


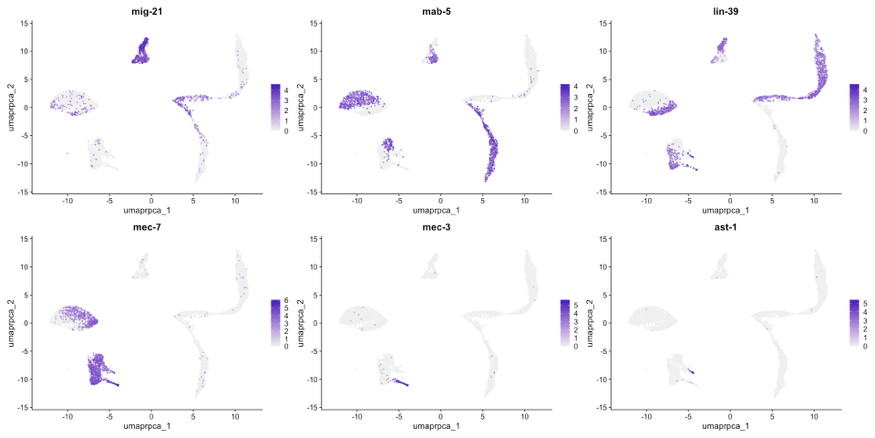


## Creating Idents based on nCount_RNA

Useful to identify cell with lower or higher RNA count on Loupe Browser

Fed_rpca$rna_count_cluster <- with(Fed_rpca@meta.data, case_when(
 nCount_RNA < 500 ~ "RNAcluster_1",
 nCount_RNA >= 500 & nCount_RNA < 1000 ~ "RNAcluster_2",
 nCount_RNA >= 1000 & nCount_RNA < 1500 ~ "RNAcluster_3",
 nCount_RNA >= 1500 & nCount_RNA < 2000 ~ "RNAcluster_4",
 nCount_RNA >= 2000 & nCount_RNA < 2500 ~ "RNAcluster_5",
 nCount_RNA >= 2500 & nCount_RNA < 3000 ~ "RNAcluster_6",
 nCount_RNA >= 3000 ~ "RNAcluster_7"
))

head(Fed_rpca)

orig.ident nCount_RNA nFeature_RNA percent.mt
OP50.2_AAACCCAAGACGGTTG-1 OP50.2 2740.6183 1215 0.09390095
OP50.2_AAACGAACAGAGGCTA-1 OP50.2 718.2502 499 0.39522174
OP50.2_AAACGAACAGCAGTTT-1 OP50.2 1128.1375 633 0.00000000
OP50.2_AAACGAAGTCTCTCTG-1 OP50.2 1434.6215 610 0.00000000
OP50.2_AAACGCTTCGCACGGT-1 OP50.2 1220.9162 666 0.00000000
OP50.2_AAAGAACGTTAGCGGA-1 OP50.2 1315.4451 802 0.05390921
OP50.2_AAAGGATTCATTGTTC-1 OP50.2 821.1808 564 0.00000000
OP50.2_AAAGGATTCGGTAACT-1 OP50.2 1498.6509 740 0.24480711
OP50.2_AAAGGGCAGTATTAGG-1 OP50.2 1606.9547 840 0.00000000
OP50.2_AAAGGTAGTATCTCTT-1 OP50.2 1872.8410 1017 0.00000000
 OP50.2.unintegrated_clusters seurat_clusters
OP50.2_AAACCCAAGACGGTTG-1 2 11
OP50.2_AAACGAACAGAGGCTA-1 10 14
OP50.2_AAACGAACAGCAGTTT-1 6 14
OP50.2_AAACGAAGTCTCTCTG-1 16 5
OP50.2_AAACGCTTCGCACGGT-1 7 1
OP50.2_AAAGAACGTTAGCGGA-1 11 0
OP50.2_AAAGGATTCATTGTTC-1 5 10
OP50.2_AAAGGATTCGGTAACT-1 13 6
OP50.2_AAAGGGCAGTATTAGG-1 7 1
OP50.2_AAAGGTAGTATCTCTT-1 1 9
 OP50.unintegrated_clusters PA14.unintegrated_clusters
OP50.2_AAACCCAAGACGGTTG-1 <NA> <NA>
OP50.2_AAACGAACAGAGGCTA-1 <NA> <NA>
OP50.2_AAACGAACAGCAGTTT-1 <NA> <NA>
OP50.2_AAACGAAGTCTCTCTG-1 <NA> <NA>
OP50.2_AAACGCTTCGCACGGT-1 <NA> <NA>
OP50.2_AAAGAACGTTAGCGGA-1 <NA> <NA>
OP50.2_AAAGGATTCATTGTTC-1 <NA> <NA>
OP50.2_AAAGGATTCGGTAACT-1 <NA> <NA>
OP50.2_AAAGGGCAGTATTAGG-1 <NA> <NA>
OP50.2_AAAGGTAGTATCTCTT-1 <NA> <NA>
 unintegrated_clusters rpca_clusters rna_count_cluster
OP50.2_AAACCCAAGACGGTTG-1 12 11 RNAcluster_6
OP50.2_AAACGAACAGAGGCTA-1 15 14 RNAcluster_2
OP50.2_AAACGAACAGCAGTTT-1 3 14 RNAcluster_3
OP50.2_AAACGAAGTCTCTCTG-1 7 5 RNAcluster_3
OP50.2_AAACGCTTCGCACGGT-1 1 1 RNAcluster_3
OP50.2_AAAGAACGTTAGCGGA-1 1 0 RNAcluster_3
OP50.2_AAAGGATTCATTGTTC-1 11 10 RNAcluster_2
OP50.2_AAAGGATTCGGTAACT-1 1 6 RNAcluster_3
OP50.2_AAAGGGCAGTATTAGG-1 1 1 RNAcluster_4
OP50.2_AAAGGTAGTATCTCTT-1 8 9 RNAcluster_4

## Creating Loupe file

create_loupe_from_seurat(Fed_rpca, output_name = "Fed_rpca_for_cleaning")

2026/01/13 11:14:35 extracting matrix, clusters, and projections

2026/01/13 11:14:35 selected assay: RNA

2026/01/13 11:14:35 selected clusters: active_cluster orig.ident OP50.2.unintegrated_clusters seurat_clusters OP50.unintegrated_clusters PA14.unintegrated_clusters unintegrated_clusters rpca_clusters rna_count_cluster

2026/01/13 11:14:35 selected projections: umap.unintegrated.fed umap.rpca

2026/01/13 11:14:35 validating count matrix

2026/01/13 11:14:36 validating clusters

2026/01/13 11:14:36 validating projections

2026/01/13 11:14:36 creating temporary hdf5 file: /var/folders/2_/0b7d0hy11bd2g2nl32tfghfh981cxm/T//RtmpZVhw0e/file18f37b9b01f6.h5

2026/01/13 11:14:37 invoking louper executable

2026/01/13 11:14:37 running command: "/Users/f403l178/Library/Application Support/org.R-project.R/R/loupeR/louper create --input='/private/var/folders/2_/0b7d0hy11bd2g2nl32tfghfh981cxm/T/RtmpZVhw0e/file18f37b9b01f6.h5' --output='Fed_rpca_for_cleaning.cloupe'"

## Importing manual clustering from Loupe file

Note: The barcode grouping of cells selected for removal were downloaded as csv from Loupe browser and imported back at this step as “cells_to_remove.csv”

Loupe_to_remove <- read_csv("input/cells_to_remove.csv")

Rows: 6822 Columns: 2
── Column specification ────────────────────────────────────────────────────────
Delimiter: ","
chr (2): Barcode, Manual

ℹ Use `spec()` to retrieve the full column specification for this data.
ℹ Specify the column types or set `show_col_types = FALSE` to quiet this message.

Fed_unint$cells_to_remove <- Loupe_to_remove$Manual
head(Fed_unint)

orig.ident nCount_RNA nFeature_RNA percent.mt
OP50.2_AAACCCAAGACGGTTG-1 OP50.2 2740.6183 1215 0.09390095
OP50.2_AAACGAACAGAGGCTA-1 OP50.2 718.2502 499 0.39522174
OP50.2_AAACGAACAGCAGTTT-1 OP50.2 1128.1375 633 0.00000000
OP50.2_AAACGAAGTCTCTCTG-1 OP50.2 1434.6215 610 0.00000000
OP50.2_AAACGCTTCGCACGGT-1 OP50.2 1220.9162 666 0.00000000
OP50.2_AAAGAACGTTAGCGGA-1 OP50.2 1315.4451 802 0.05390921
OP50.2_AAAGGATTCATTGTTC-1 OP50.2 821.1808 564 0.00000000
OP50.2_AAAGGATTCGGTAACT-1 OP50.2 1498.6509 740 0.24480711
OP50.2_AAAGGGCAGTATTAGG-1 OP50.2 1606.9547 840 0.00000000
OP50.2_AAAGGTAGTATCTCTT-1 OP50.2 1872.8410 1017 0.00000000
 OP50.2.unintegrated_clusters seurat_clusters
OP50.2_AAACCCAAGACGGTTG-1 2 12
OP50.2_AAACGAACAGAGGCTA-1 10 15
OP50.2_AAACGAACAGCAGTTT-1 6 3
OP50.2_AAACGAAGTCTCTCTG-1 16 7
OP50.2_AAACGCTTCGCACGGT-1 7 1
OP50.2_AAAGAACGTTAGCGGA-1 11 1
OP50.2_AAAGGATTCATTGTTC-1 5 11
OP50.2_AAAGGATTCGGTAACT-1 13 1
OP50.2_AAAGGGCAGTATTAGG-1 7 1
OP50.2_AAAGGTAGTATCTCTT-1 1 8
 OP50.unintegrated_clusters PA14.unintegrated_clusters
OP50.2_AAACCCAAGACGGTTG-1 <NA> <NA>
OP50.2_AAACGAACAGAGGCTA-1 <NA> <NA>
OP50.2_AAACGAACAGCAGTTT-1 <NA> <NA>
OP50.2_AAACGAAGTCTCTCTG-1 <NA> <NA>
OP50.2_AAACGCTTCGCACGGT-1 <NA> <NA>
OP50.2_AAAGAACGTTAGCGGA-1 <NA> <NA>
OP50.2_AAAGGATTCATTGTTC-1 <NA> <NA>
OP50.2_AAAGGATTCGGTAACT-1 <NA> <NA>
OP50.2_AAAGGGCAGTATTAGG-1 <NA> <NA>
OP50.2_AAAGGTAGTATCTCTT-1 <NA> <NA>
 unintegrated_clusters cells_to_remove
OP50.2_AAACCCAAGACGGTTG-1 12 Cells_to_keep
OP50.2_AAACGAACAGAGGCTA-1 15 Cells_to_keep
OP50.2_AAACGAACAGCAGTTT-1 3 Cells_to_keep
OP50.2_AAACGAAGTCTCTCTG-1 7 Cells_to_keep
OP50.2_AAACGCTTCGCACGGT-1 1 Cells_to_keep
OP50.2_AAAGAACGTTAGCGGA-1 1 Cells_to_keep
OP50.2_AAAGGATTCATTGTTC-1 11 Cells_to_keep
OP50.2_AAAGGATTCGGTAACT-1 1 Cells_to_keep
OP50.2_AAAGGGCAGTATTAGG-1 1 Cells_to_keep
OP50.2_AAAGGTAGTATCTCTT-1 8 Cells_to_keep

DimPlot(Fed_unint, reduction = "umap.unintegrated.fed", label = T, group.by = "cells_to_remove")


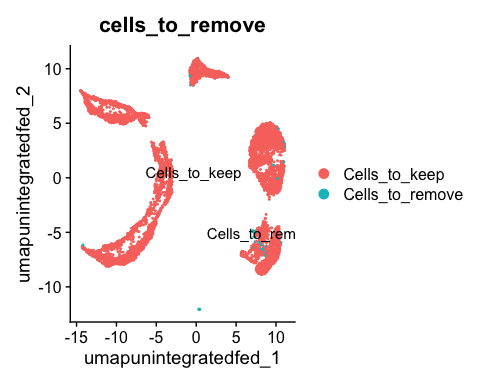


## Removing low quality cells

Idents(Fed_unint) <- "cells_to_remove"

manual_cells_to_remove.unint <- WhichCells(Fed_unint, idents = c("Cells_to_remove"))

Fed_unint_v2 <- subset(Fed_unint, cells = setdiff(Cells(Fed_unint), manual_cells_to_remove.unint))

Idents(Fed_unint) <- "seurat_clusters"
Idents(Fed_unint_v2) <- "seurat_clusters"

Fed_unint_v2 <- NormalizeData(Fed_unint_v2)

Normalizing layer: counts.OP50.2

Normalizing layer: counts.OP50

Normalizing layer: counts.PA14

Fed_unint_v2 <- FindVariableFeatures(Fed_unint_v2)

Finding variable features for layer counts.OP50.2

Finding variable features for layer counts.OP50

Finding variable features for layer counts.PA14

cele_features <- rownames(Fed_unint_v2)
Fed_unint_v2 <- ScaleData(Fed_unint_v2, features = cele_features)

Centering and scaling data matrix

Fed_unint_v2 <- RunPCA(Fed_unint_v2)

PC_ 1
Positive: rpl-12, ctsa-1.1, rbm-3.2, rpl-7A, rpl-17, eef-1A.1, eef-2, ZK930.6, rps-18, rpl-32
 eef-1B.1, rnr-1, his-24, eef-1G, rpl-36, rpl-18, rps-8, rpl-20, rps-1, rpl-5
 rps-5, rps-23, rpl-10, C50F4.6, rpl-3, rpl-16, R05H5.3, rps-0, rpl-2, hil-2
Negative: mks-2, C27A2.8, gcy-37, arl-3, pdl-1, gcy-35, ZK616.1, F49E12.8, sbt-1, T04C12.9
 gcy-36, fmi-1, ceph-41, K07C11.10, mksr-2, tmem-231, arcp-1, ifta-2, dylt-2, gcy-34
 WBGene00014307, guk-1, che-2, nphp-1, ift-81, ptb-1, gcy-33, dyf-18, flp-14, mksr-1
PC_ 2
Positive: mig-21, R05H11.2, unc-54, cpn-1, suro-1, T25G12.3, F47B7.1, egl-17, WBGene00022730, emb-9
 W07A12.4, rpl-30, mcm-6, bar-1, rpl-31, rps-25, tsp-14, R05D3.9, rps-6, pde-1
 ztf-16, gcy-33, rla-0, F49E2.5, rps-22, M60.4, rla-1, rps-2, rps-17, T23F2.5
Negative: hlh-14, mec-7, egl-46, F33A8.7, ctsa-1.1, C08F1.10, C09D4.2, nid-1, C40A11.6, tbca-1
 rhgf-2, dma-1, F44E5.1, C01C4.3, his-24, tmed-13, atf-2, F36G3.1, tsp-7, C06E7.2
 R11G1.2, unc-68, cank-26, WBGene00023302, cki-1, WBGene00050903, F07C6.4, C10A4.3, syd-9, pat-2
PC_ 3
Positive: lin-32, Y7A9D.1, gst-7, T01D3.3, unc-54, ham-1, F48E3.9, C03C10.5, WBGene00023302, dma-1
 pkd-2, K09F6.13, che-2, F23B12.4, ssq-1, egl-13, mks-5, tctn-1, gcy-36, ccep-290
 gasr-8, syd-9, F58E2.5, R01H2.8, W07A12.4, fmi-1, tmem-231, nphp-1, msa-1, eat-20
Negative: his-24, pde-1, mec-7, nlp-43, flp-21, ins-18, WBGene00044308, T05A8.3, snt-4, gcy-33
 hil-3, aexr-2, C08G9.1, hlh-14, pgal-1, F58F9.1, nphp-2, F33A8.7, C10C5.7, ampd-1
 flp-14, nlp-47, F32B4.5, glb-1, die-1, unc-68, plc-1, B0205.14, srg-25, F49E10.4
PC_ 4
Positive: F32H5.3, hphd-1, cyd-1, mab-5, lat-1, pezo-1, M162.5, cdl-1, C33A12.4, Y47A7.2
 cutl-10, F35B3.7, Y43B11AR.1, mcm-7, cyk-7, hyl-1, odr-4, dct-14, flp-4, unc-5
 T22B7.22, H37A05.4, fkh-5, sto-1, glb-24, gcy-34, ZK742.7, guk-1, T01D3.3, seb-3
Negative: lipl-7, srd-30, lgc-27, pkd-2, lin-32, ssq-1, srd-29, C43D7.8, smp-1, tbx-2
 egl-46, mig-13, Y54H5A.2, F07C6.4, ZK131.11, Y7A9D.1, cank-26, F58H1.7, T05A8.3, WBGene00023302
 Y71F9AL.6, ham-1, nlp-43, lgc-52, flp-5, exc-9, plc-1, magi-1, F58F9.1, nlp-47
PC_ 5
Positive: F32H5.3, Y54H5A.2, tbx-2, pkd-2, hphd-1, cyd-1, F07C6.4, ZK930.6, lipl-7, ssq-1
 srd-30, Y47A7.2, T22B7.22, ztf-11, srd-29, smp-1, cdl-1, C43D7.8, lgc-27, grl-10
 flp-5, gst-7, ham-1, mcm-7, thk-1, egl-13, WBGene00023302, sem-2, pezo-1, H37A05.4
Negative: hlh-14, mig-1, F33A8.7, mec-7, nid-1, C09D4.2, unc-68, tsp-7, F36G3.1, M01H9.4
 C06E7.2, C01C4.3, C40A11.6, mec-3, C08F1.10, bar-1, T25G12.3, F14H12.8, ttr-18, arrd-25
 C15C8.5, F46F2.3, atf-2, nhr-25, F16F9.1, M60.4, tbca-1, C10A4.3, nlp-24, wrt-3

ElbowPlot(Fed_unint_v2, ndims = 50)


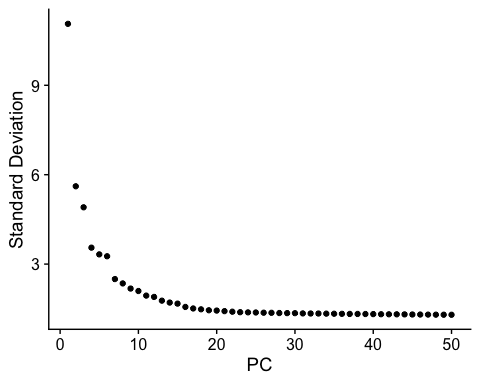


Fed_unint_v2 <- FindNeighbors(Fed_unint_v2, dims = 1:21, reduction = "pca")

Computing nearest neighbor graph

Computing SNN

Fed_unint_v2 <- FindClusters(Fed_unint_v2, resolution = 1.0, cluster.name = "unintegrated_clusters")

Modularity Optimizer version 1.3.0 by Ludo Waltman and Nees Jan van Eck

Number of nodes: 6743
Number of edges: 258478

Running Louvain algorithm...
Maximum modularity in 10 random starts: 0.8738
Number of communities: 18
Elapsed time: 0 seconds

Fed_unint_v2 <- RunUMAP(Fed_unint_v2, dims = 1:21, reduction = "pca", reduction.name = "umap.unintegrated.fed")

11:15:09 UMAP embedding parameters a = 0.9922 b = 1.112

11:15:09 Read 6743 rows and found 21 numeric columns

11:15:09 Using Annoy for neighbor search, n_neighbors = 30

11:15:09 Building Annoy index with metric = cosine, n_trees = 50

0% 10 20 30 40 50 60 70 80 90 100%

[----|----|----|----|----|----|----|----|----|----|

**************************************************|
11:15:10 Writing NN index file to temp file /var/folders/2_/0b7d0hy11bd2g2nl32tfghfh981cxm/T//RtmpZVhw0e/file18f32366a89d
11:15:10 Searching Annoy index using 1 thread, search_k = 3000
11:15:11 Annoy recall = 100%
11:15:13 Commencing smooth kNN distance calibration using 1 thread with target n_neighbors = 30
11:15:14 Initializing from normalized Laplacian + noise (using RSpectra)
11:15:15 Commencing optimization for 500 epochs, with 275048 positive edges
11:15:15 Using rng type: pcg
11:15:23 Optimization finished

DimPlot(Fed_unint, reduction = "umap.unintegrated.fed", group.by = "orig.ident",
 cols = c("#e8000d", "gold", "#0051ba")) +
 DimPlot(Fed_unint, reduction = "umap.unintegrated.fed", label = T)


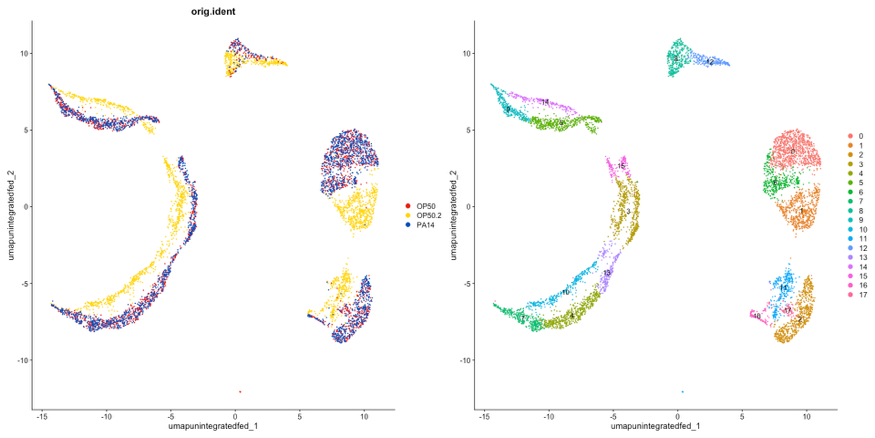


DimPlot(Fed_unint_v2, reduction = "umap.unintegrated.fed", group.by = "orig.ident",
 cols = c("#e8000d", "gold", "#0051ba")) +
 DimPlot(Fed_unint_v2, reduction = "umap.unintegrated.fed", label = T)


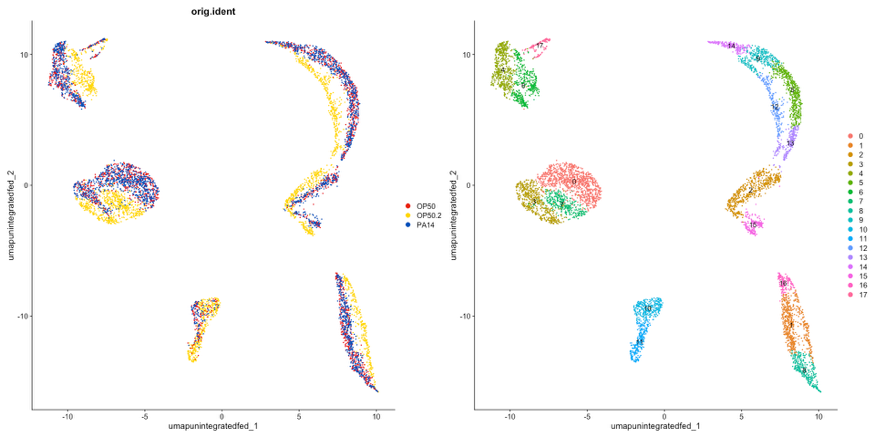


FeaturePlot(Fed_unint_v2, reduction = "umap.unintegrated.fed",
 features = c("mig-21","mab-5", "lin-39", "gcy-32", "mec-7", "mec-3", "ast-1", "ajm-1"),
 label = F, order = T, cols = c("#efefef", "#4011ba"), ncol = 4)


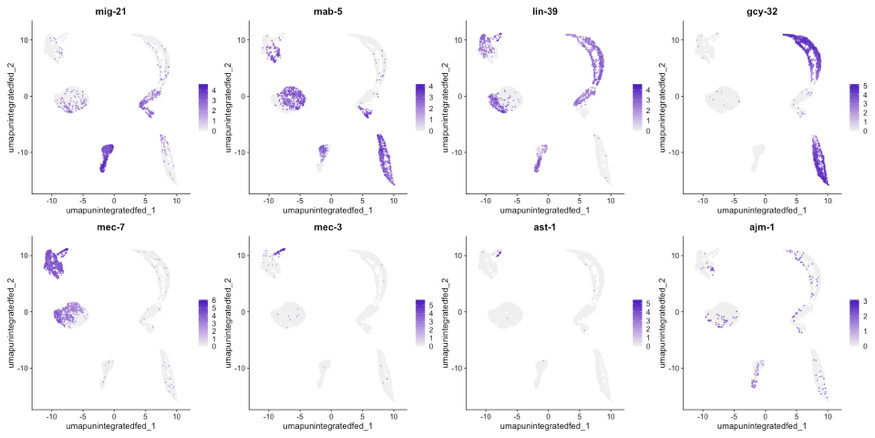


# -

# Integration (RPCA) AFTER manual cleaning

Fed_rpca_v2 <- IntegrateLayers(
 object = Fed_unint_v2, method = RPCAIntegration,
 orig.reduction = "pca", new.reduction = "integrated.rpca"
)

Computing within dataset neighborhoods

Finding all pairwise anchors

Projecting new data onto SVD
Projecting new data onto SVD

Finding neighborhoods

Finding anchors

Found 1284 anchors

Projecting new data onto SVD
Projecting new data onto SVD

Finding neighborhoods

Finding anchors

Found 1355 anchors

Projecting new data onto SVD
Projecting new data onto SVD

Finding neighborhoods

Finding anchors

Found 1525 anchors

Merging dataset 2 into 3

Extracting anchors for merged samples

Finding integration vectors

Finding integration vector weights

Integrating data

Merging dataset 1 into 3 2

Extracting anchors for merged samples

Finding integration vectors

Finding integration vector weights

Integrating data

Fed_rpca_v2[["RNA"]] <- JoinLayers(Fed_rpca_v2[["RNA"]])

ElbowPlot(Fed_rpca_v2 , ndims = 50)


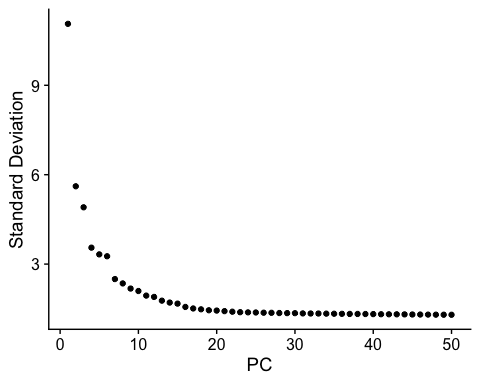


Fed_rpca_v2 <- FindNeighbors(Fed_rpca_v2, reduction = "integrated.rpca", dims = 1:11)

Computing nearest neighbor graph

Computing SNN

Fed_rpca_v2 <- FindClusters(Fed_rpca_v2, resolution = 1.2, cluster.name = "rpca_clusters")

Modularity Optimizer version 1.3.0 by Ludo Waltman and Nees Jan van Eck

Number of nodes: 6743
Number of edges: 242426

Running Louvain algorithm...
Maximum modularity in 10 random starts: 0.8699
Number of communities: 18
Elapsed time: 0 seconds

Fed_rpca_v2 <- RunUMAP(Fed_rpca_v2, dims = 1:11, reduction = "integrated.rpca", reduction.name = "umap.rpca")

11:15:57 UMAP embedding parameters a = 0.9922 b = 1.112

11:15:57 Read 6743 rows and found 11 numeric columns

11:15:57 Using Annoy for neighbor search, n_neighbors = 30

11:15:57 Building Annoy index with metric = cosine, n_trees = 50

0% 10 20 30 40 50 60 70 80 90 100%

[----|----|----|----|----|----|----|----|----|----|

**************************************************|
11:15:57 Writing NN index file to temp file /var/folders/2_/0b7d0hy11bd2g2nl32tfghfh981cxm/T//RtmpZVhw0e/file18f34ae0b3e3
11:15:57 Searching Annoy index using 1 thread, search_k = 3000
11:15:59 Annoy recall = 100%
11:16:00 Commencing smooth kNN distance calibration using 1 thread with target n_neighbors = 30
11:16:02 Initializing from normalized Laplacian + noise (using RSpectra)
11:16:02 Commencing optimization for 500 epochs, with 269234 positive edges
11:16:02 Using rng type: pcg
11:16:11 Optimization finished

DimPlot(Fed_rpca_v2, reduction = "umap.rpca", label = T)


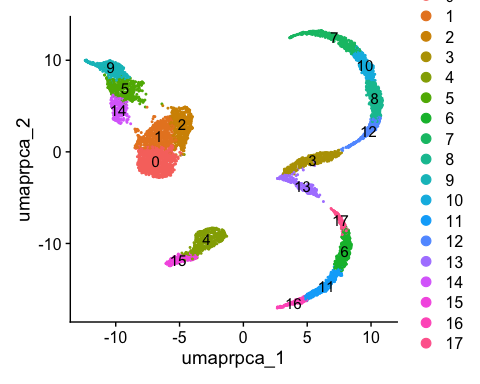


DimPlot(Fed_rpca_v2, reduction = "umap.rpca", label = T) + FeaturePlot(Fed_rpca_v2, reduction = "umap.rpca",
 features = c("mab-5", "lin-39"),
 label = F, order = T, cols = c("#efefef", "#4011ba"), ncol = 1)


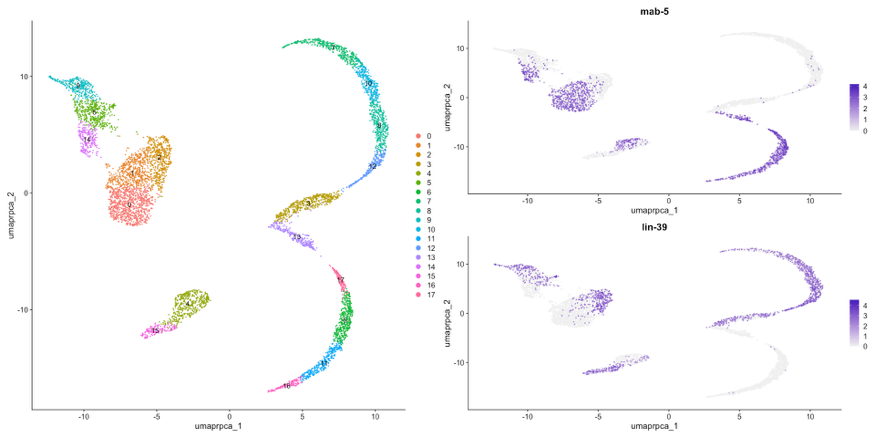


FeaturePlot(Fed_rpca_v2, reduction = "umap.rpca",
 features = c("mig-21","mab-5", "lin-39", "gcy-32", "mec-7", "mec-3","ast-1", "nCount_RNA"),
 label = F, order = T, cols = c("#efefef", "#4011ba"), ncol = 4)


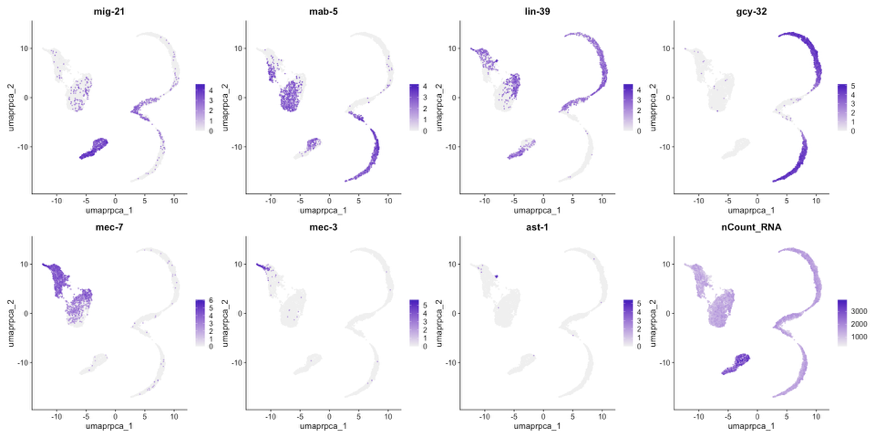


## Adding custom metadata to object through Loupe browser

Note: Loupe browser makes it easier to add custom cluster information based on marker genes that wouldn’t be detected using Seurat clustering (e.g., Qx.pax clusters)

### Adjusting object metadata before exporting - adding info (and removing extra info) to loupe file

# Create new column on meta data based on orig.ident
Fed_rpca_v2$experiment_clusters <- Fed_rpca_v2$orig.ident

# Remove unwanted column from meta data
Fed_rpca_v2$OP50.unintegrated_clusters <- NULL
Fed_rpca_v2$PA14.unintegrated_clusters <- NULL
Fed_rpca_v2$OP50.2.unintegrated_clusters <- NULL
Fed_rpca_v2$unintegrated_clusters <- NULL
Fed_rpca_v2$cells_to_remove <- NULL
Fed_rpca_v2$rpca_clusters <- NULL
Fed_rpca_v2$orig.ident <- NULL

head(Fed_rpca_v2)

nCount_RNA nFeature_RNA percent.mt seurat_clusters
OP50.2_AAACCCAAGACGGTTG-1 2740.6183 1215 0.09390095 15
OP50.2_AAACGAACAGAGGCTA-1 718.2502 499 0.39522174 13
OP50.2_AAACGAACAGCAGTTT-1 1128.1375 633 0.00000000 13
OP50.2_AAACGAAGTCTCTCTG-1 1434.6215 610 0.00000000 7
OP50.2_AAACGCTTCGCACGGT-1 1220.9162 666 0.00000000 2
OP50.2_AAAGAACGTTAGCGGA-1 1315.4451 802 0.05390921 2
OP50.2_AAAGGATTCATTGTTC-1 821.1808 564 0.00000000 14
OP50.2_AAAGGATTCGGTAACT-1 1498.6509 740 0.24480711 0
OP50.2_AAAGGGCAGTATTAGG-1 1606.9547 840 0.00000000 2
OP50.2_AAAGGTAGTATCTCTT-1 1872.8410 1017 0.00000000 4
 experiment_clusters
OP50.2_AAACCCAAGACGGTTG-1 OP50.2
OP50.2_AAACGAACAGAGGCTA-1 OP50.2
OP50.2_AAACGAACAGCAGTTT-1 OP50.2
OP50.2_AAACGAAGTCTCTCTG-1 OP50.2
OP50.2_AAACGCTTCGCACGGT-1 OP50.2
OP50.2_AAAGAACGTTAGCGGA-1 OP50.2
OP50.2_AAAGGATTCATTGTTC-1 OP50.2
OP50.2_AAAGGATTCGGTAACT-1 OP50.2
OP50.2_AAAGGGCAGTATTAGG-1 OP50.2
OP50.2_AAAGGTAGTATCTCTT-1 OP50.2

### Creating Loupe file

create_loupe_from_seurat(Fed_rpca_v2, output_name = "Fed_rpca_clustering")

2026/01/13 11:16:15 extracting matrix, clusters, and projections

2026/01/13 11:16:16 selected assay: RNA

2026/01/13 11:16:16 selected clusters: active_cluster seurat_clusters experiment_clusters

2026/01/13 11:16:16 selected projections: umap.unintegrated.fed umap.rpca

2026/01/13 11:16:16 validating count matrix

2026/01/13 11:16:16 validating clusters

2026/01/13 11:16:16 validating projections

2026/01/13 11:16:16 creating temporary hdf5 file: /var/folders/2_/0b7d0hy11bd2g2nl32tfghfh981cxm/T//RtmpZVhw0e/file18f32a547a4d.h5

2026/01/13 11:16:17 invoking louper executable

2026/01/13 11:16:17 running command: "/Users/f403l178/Library/Application Support/org.R-project.R/R/loupeR/louper create --input='/private/var/folders/2_/0b7d0hy11bd2g2nl32tfghfh981cxm/T/RtmpZVhw0e/file18f32a547a4d.h5' --output='Fed_rpca_clustering.cloupe'"

### Importing manual clustering from Loupe file

Note: We manually labeled clusters as different custom groups (e.g., with or without L-R identity) in Loupe browser, downloaded barcode grouping as csv, and imported back to use in downstream analyses.

Manual_clustering <- read_csv("input/Manual_clustering.csv")

Rows: 6743 Columns: 2
── Column specification ────────────────────────────────────────────────────────
Delimiter: ","
chr (2): Barcode, Manual_clustering

ℹ Use `spec()` to retrieve the full column specification for this data.
ℹ Specify the column types or set `show_col_types = FALSE` to quiet this message.

Fed_rpca_v2$Q.cell_clusters <- Manual_clustering$Manual_clustering

Manual_clustering <- read_csv("input/Manual_clustering_v2.csv")

Rows: 6743 Columns: 2
── Column specification ────────────────────────────────────────────────────────
Delimiter: ","
chr (2): Barcode, Manual_clustering_v2

ℹ Use `spec()` to retrieve the full column specification for this data.
ℹ Specify the column types or set `show_col_types = FALSE` to quiet this message.

Fed_rpca_v2$Q.cell_v2_clusters <- Manual_clustering$Manual_clustering_v2

Manual_clustering <- read_csv("input/Manual_clustering_v3.csv")

Rows: 6743 Columns: 2
── Column specification ────────────────────────────────────────────────────────
Delimiter: ","
chr (2): Barcode, Manual_clustering_v3

ℹ Use `spec()` to retrieve the full column specification for this data.
ℹ Specify the column types or set `show_col_types = FALSE` to quiet this message.

Fed_rpca_v2$Q.cell_v3_clusters <- Manual_clustering$Manual_clustering_v3

Manual_clustering <- read_csv("input/Manual_clustering_v4.csv")

Rows: 6743 Columns: 2
── Column specification ────────────────────────────────────────────────────────
Delimiter: ","
chr (2): Barcode, Manual_clustering_v4

ℹ Use `spec()` to retrieve the full column specification for this data.
ℹ Specify the column types or set `show_col_types = FALSE` to quiet this message.

Fed_rpca_v2$Q.cell_v4_clusters <- Manual_clustering$Manual_clustering_v4

HighRes <- read_csv("input/HighRes.csv")

Rows: 6743 Columns: 2
── Column specification ────────────────────────────────────────────────────────
Delimiter: ","
chr (2): Barcode, HighRes

ℹ Use `spec()` to retrieve the full column specification for this data.
ℹ Specify the column types or set `show_col_types = FALSE` to quiet this message.

Fed_rpca_v2$Q.cell_v5_clusters <- HighRes$HighRes

DimPlot(Fed_rpca_v2, reduction = "umap.rpca", label = T)


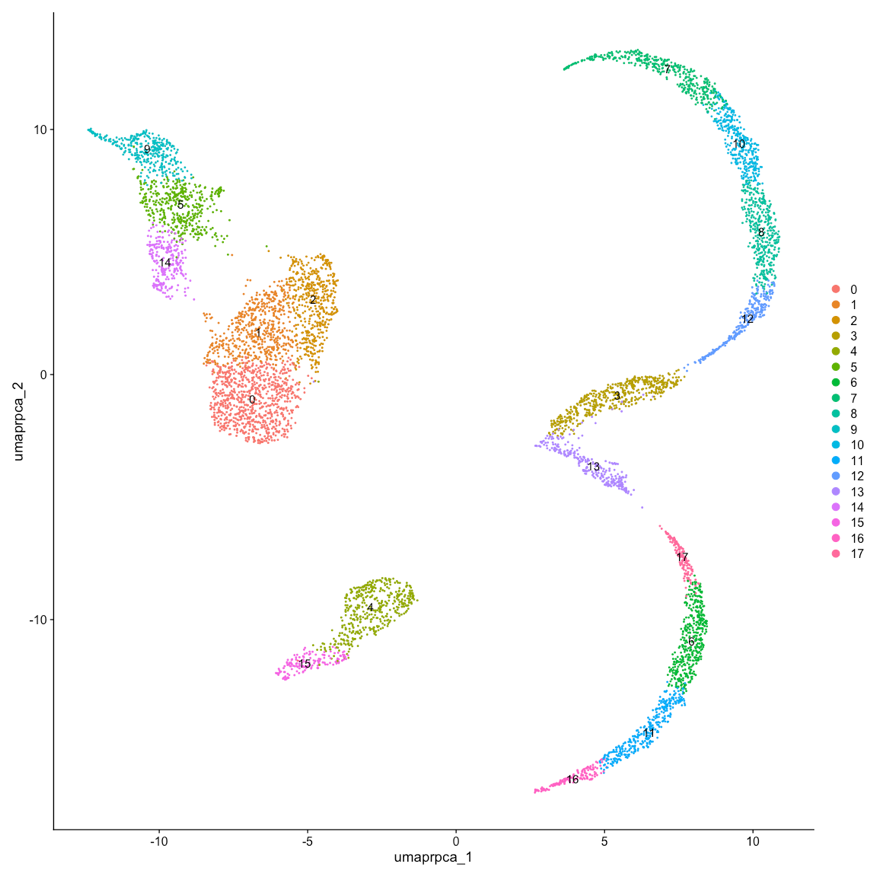


DimPlot(Fed_rpca_v2, reduction = "umap.rpca", label = T, group.by = "Q.cell_clusters")


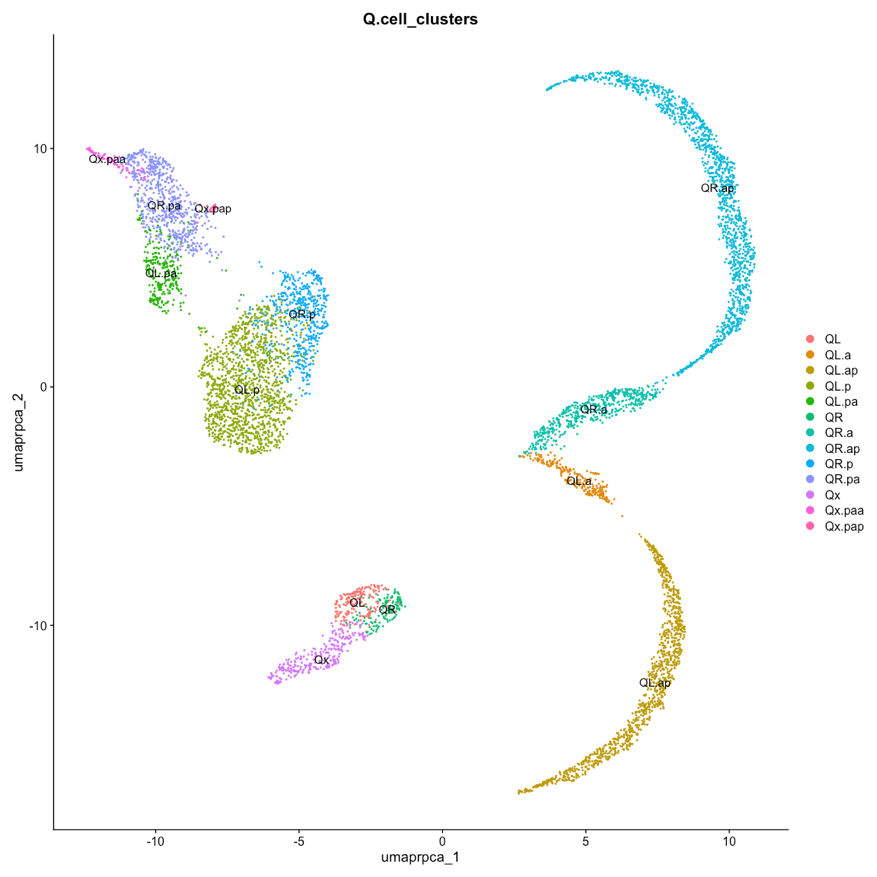


DimPlot(Fed_rpca_v2, reduction = "umap.rpca", label = T, group.by = "Q.cell_v2_clusters")


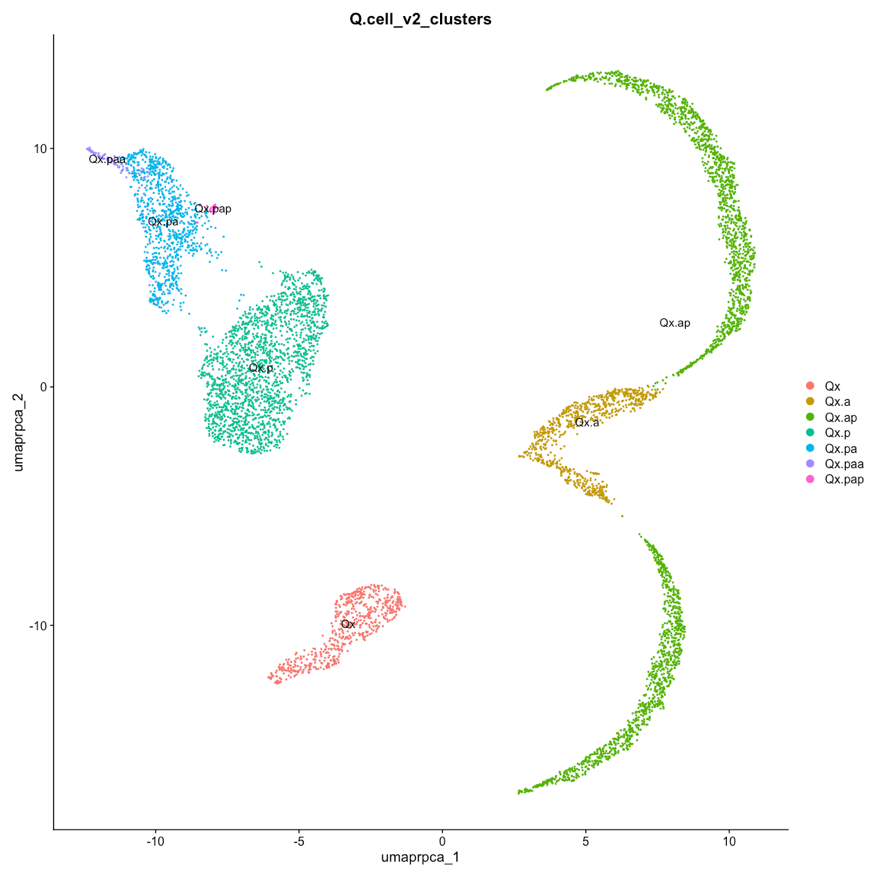


DimPlot(Fed_rpca_v2, reduction = "umap.rpca", label = T, group.by = "Q.cell_v3_clusters")


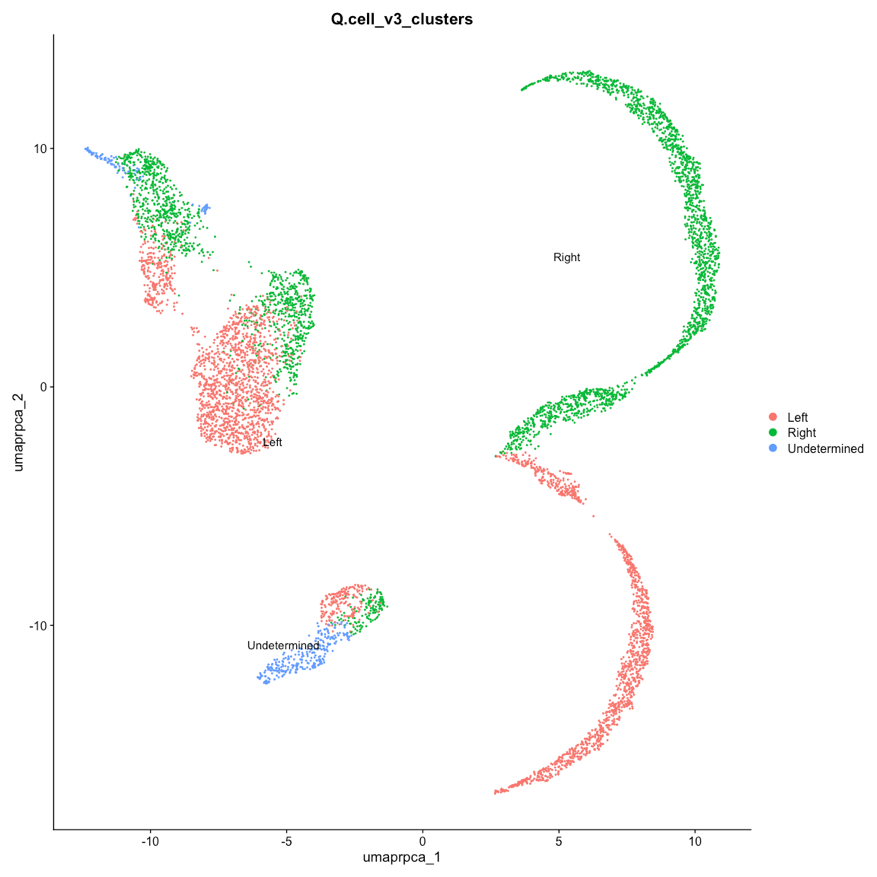


DimPlot(Fed_rpca_v2, reduction = "umap.rpca", label = T, group.by = "Q.cell_v4_clusters")


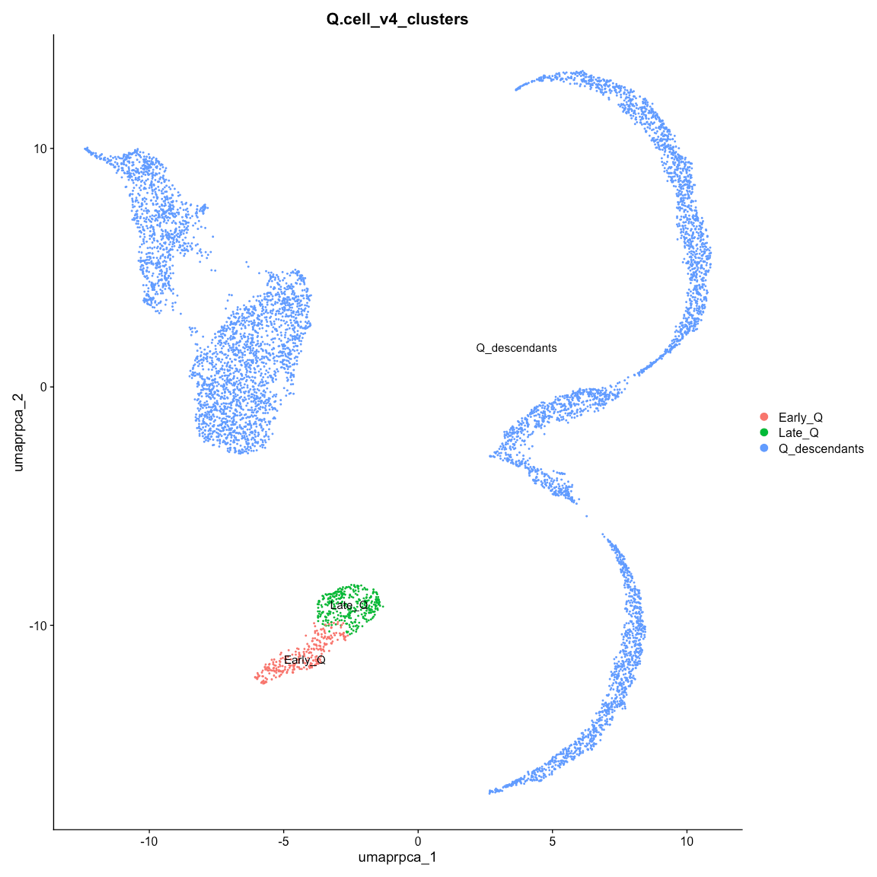


DimPlot(Fed_rpca_v2, reduction = "umap.rpca", label = T, group.by = "Q.cell_v5_clusters")


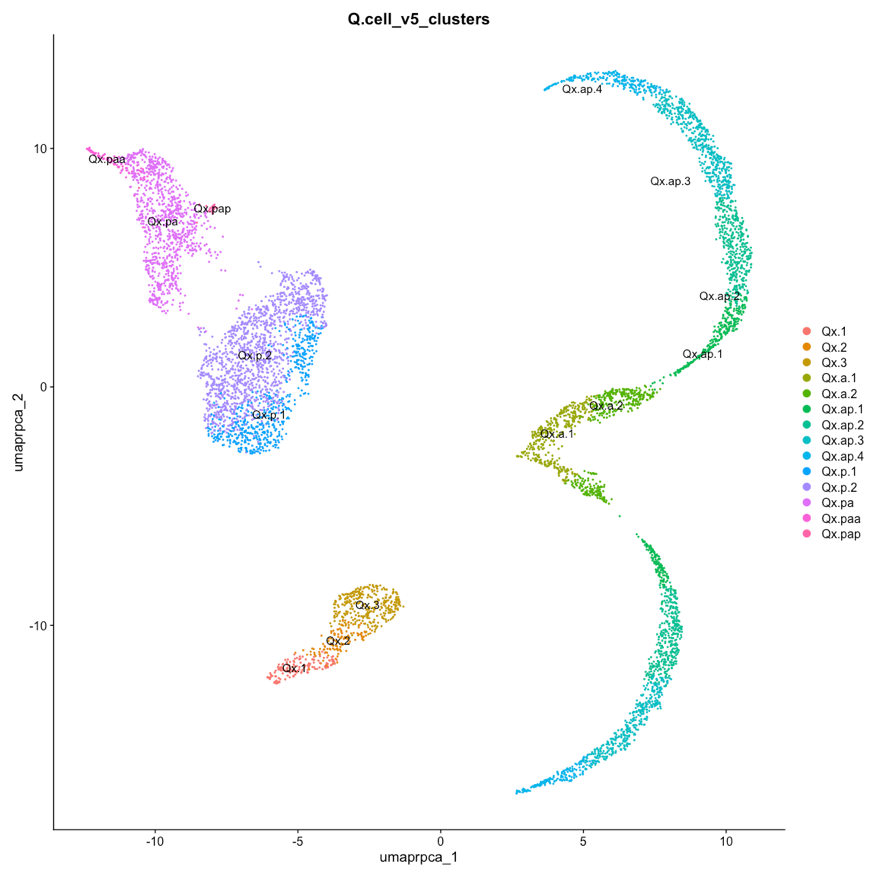


## Reordering clusters based (kind of) on cell hierarchy

Idents(Fed_rpca_v2) <- "Q.cell_clusters"
levels(Fed_rpca_v2)

[1] "Qx" "QL.a" "QR.ap" "QR.p" "QL.p" "QL.pa" "QR" "QL.ap"
 [9] "QR.pa" "QR.a" "Qx.paa" "QL" "Qx.pap"

cluster_order <- c("Qx", "QL", "QR", "QL.a","QR.a", "QL.ap", "QR.ap", "QL.p", "QR.p", "QL.pa", "QR.pa", "Qx.paa", "Qx.pap")
levels(Fed_rpca_v2) <- cluster_order
DimPlot(Fed_rpca_v2, reduction = "umap.rpca", label = T)


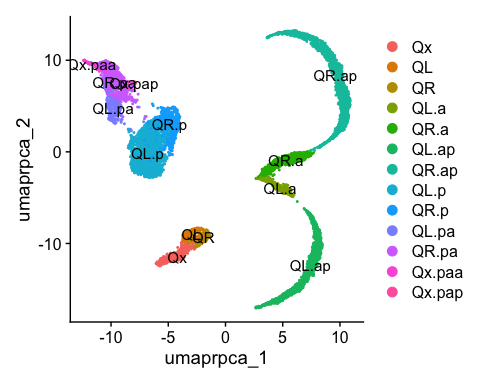


Fed_rpca_v2$Q.cell_clusters <- factor(Fed_rpca_v2$Q.cell_clusters,
 levels = c("Qx", "QL", "QR", "QL.a","QR.a", "QL.p", "QR.p", "QL.ap", "QR.ap", "QL.pa", "QR.pa", "Qx.paa", "Qx.pap"))


cluster_order_v2 <- c("Qx", "Qx.a", "Qx.ap", "Qx.p", "Qx.pa", "Qx.paa", "Qx.pap")
cluster_order_v2.2 <- c("Qx", "Qx.a", "Qx.p", "Qx.ap", "Qx.pa", "Qx.paa", "Qx.pap")
cluster_order_v2.3 <- c("Qx.ap", "Qx.a", "Qx", "Qx.p", "Qx.pa", "Qx.paa", "Qx.pap")
#Fed_rpca_v2$Q.cell_v2_clusters <- factor(Fed_rpca_v2$Q.cell_v2_clusters,
# levels = c("Qx", "Qx.a", "Qx.ap", "Qx.p", "Qx.pa", "Qx.paa", "Qx.pap"))
Fed_rpca_v2$Q.cell_v2_clusters <- factor(Fed_rpca_v2$Q.cell_v2_clusters,
 levels = c("Qx", "Qx.a", "Qx.p", "Qx.ap", "Qx.pa", "Qx.paa", "Qx.pap"))
#Fed_rpca_v2$Q.cell_v2_clusters <- factor(Fed_rpca_v2$Q.cell_v2_clusters,
# levels = c("Qx.ap", "Qx.a", "Qx", "Qx.p", "Qx.pa", "Qx.paa", "Qx.pap"))

Fed_rpca_v2$Q.cell_v5_clusters <- factor(Fed_rpca_v2$Q.cell_v5_clusters,
 levels = c("Qx.1","Qx.2","Qx.3", "Qx.a.1","Qx.a.2", "Qx.p.1", "Qx.p.2", "Qx.ap.1","Qx.ap.2","Qx.ap.3","Qx.ap.4", "Qx.pa", "Qx.paa", "Qx.pap"))

## Recoloring clusters

cluster_colors <- c(
 "Qx" = "#96b04a",
 "QL" = "#69e14b",
 "QR" = "#257525",
 "QL.a" = "#FF4C34",
 "QR.a" = "#b73737",
 "QL.ap" = "#f7c360",
 "QR.ap" = "#c48d25",
 "QL.p" = "#4aabff",
 "QR.p" = "#256aa6",
 "QL.pa" = "#fc53c1",
 "QR.pa" = "#9c3aa9",
 "Qx.paa" = "#377f6f",
 "Qx.pap" = "#28afaa"
)


cluster_colors_v2 <- c(
 "Qx" = "#7cbb5d",
 "Qx.a" = "#d55735",
 "Qx.ap" = "#ecb24d",
 "Qx.p" = "#5793d7",
 "Qx.pa" = "#b44a93",
 "Qx.paa" = "#489d97",
 "Qx.pap" = "#489d97"
)

cluster_colors_v3 <- c(
 "Left" = "red",
 "Right" = "royalblue",
 "Undetermined" = "grey"

)


cluster_colors_v4 <- c(
 "Early_Q" = "#028A0F",
 "Late_Q" = "purple",
 "Q_descendants" = "grey"

)

#### - Figs 1, 3, 4, 5 - UMAP plots

Fig.1_UMAP <- DimPlot(Fed_rpca_v2, reduction = "umap.rpca", label = F, repel = T, label.size = 5, label.color = "black", pt.size = 1) + scale_color_manual(values = cluster_colors) + NoLegend()
Fig.5_UMAP <- DimPlot(Fed_rpca_v2, reduction = "umap.rpca", label = F, group.by = "Q.cell_v2_clusters")+ scale_color_manual(values = cluster_colors_v2) + ggtitle(NULL)
Fig.4_UMAP <- DimPlot(Fed_rpca_v2, reduction = "umap.rpca", label = F, group.by = "Q.cell_v3_clusters")+ scale_color_manual(values = cluster_colors_v3) + ggtitle(NULL)
Fig.3_UMAP <- DimPlot(Fed_rpca_v2, reduction = "umap.rpca", label = F, group.by = "Q.cell_v4_clusters")+ scale_color_manual(values = cluster_colors_v4) + ggtitle(NULL)


Fig.1_UMAP


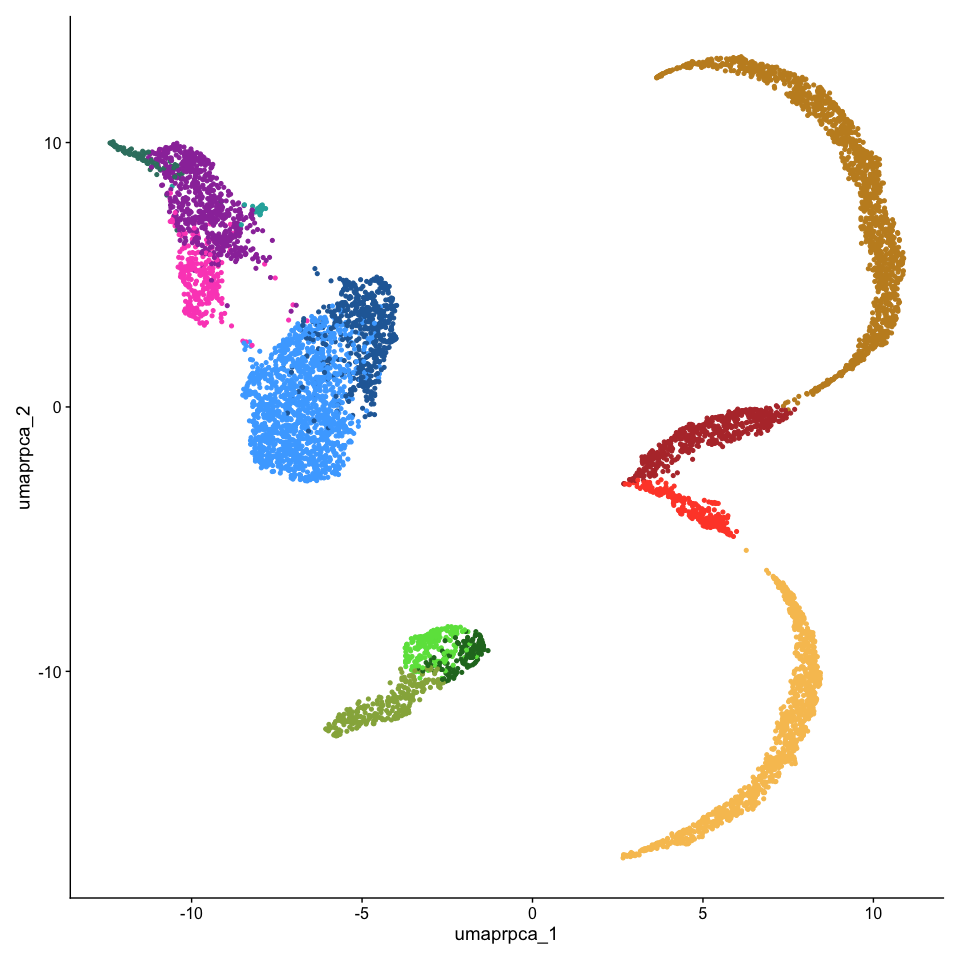


Fig.3_UMAP


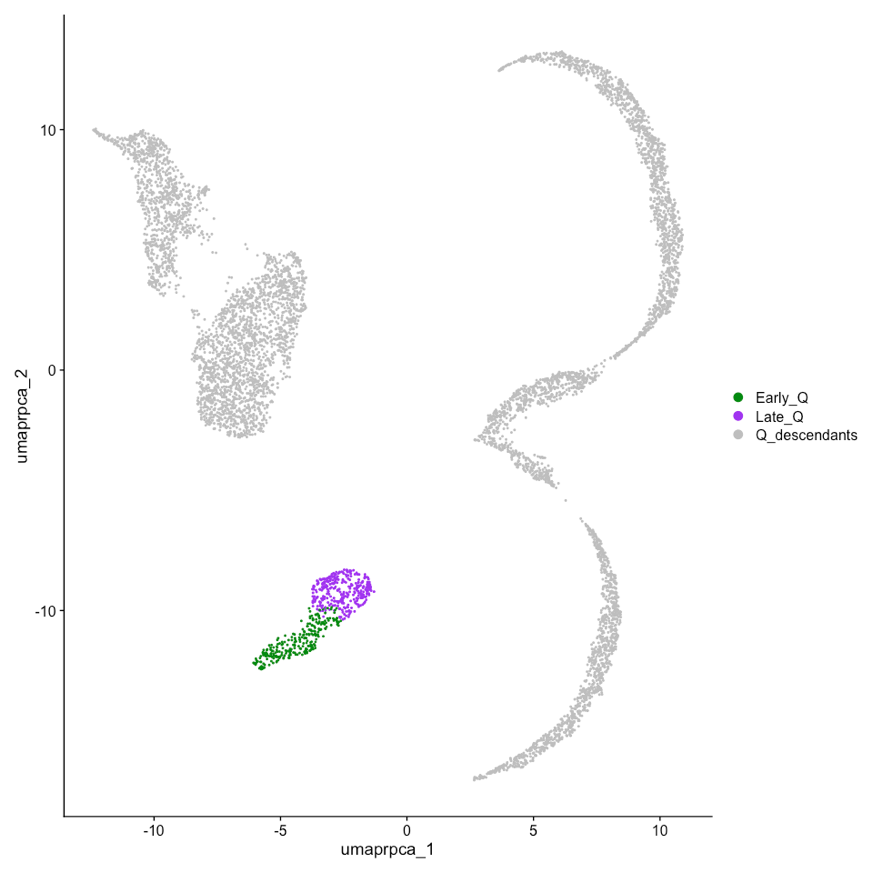


Fig.4_UMAP


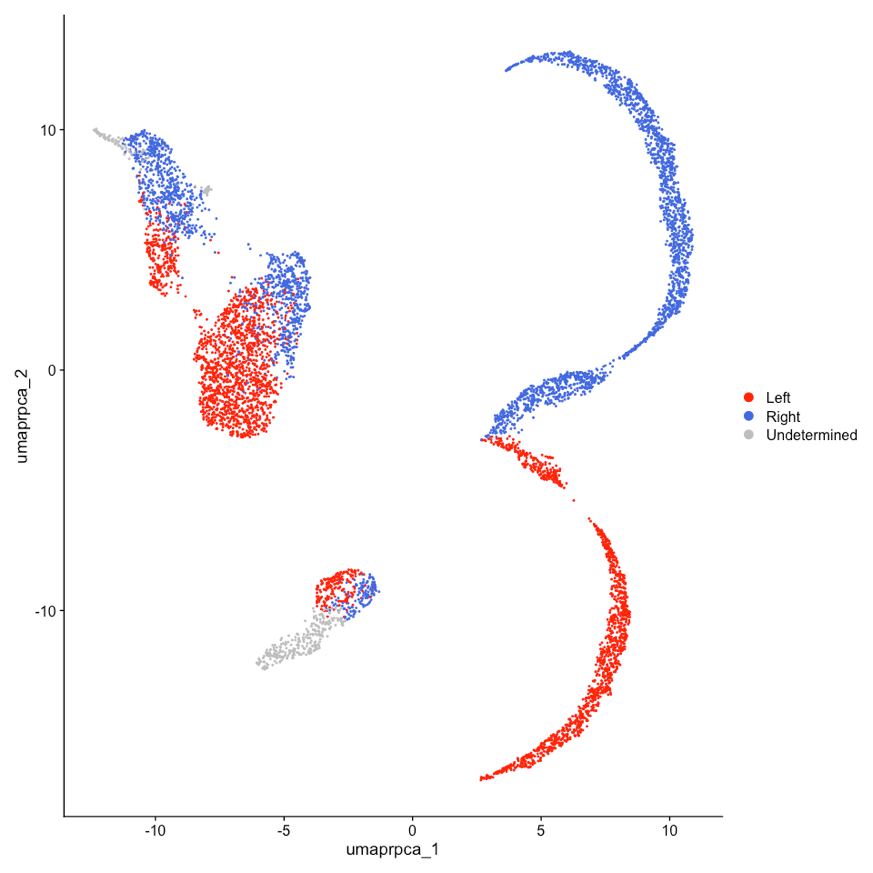


Fig.5_UMAP


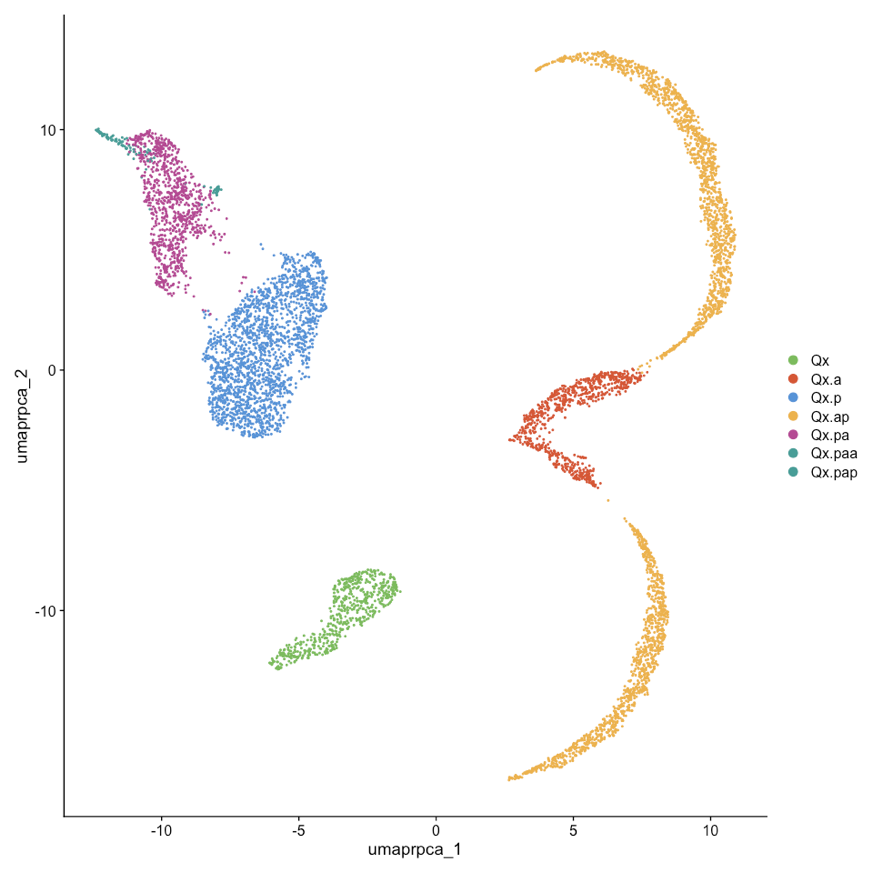


#### – S1 Table - UMI and gene count/cluster

meta <- Fed_rpca_v2@meta.data

meta$nUMI <- Matrix::colSums(GetAssayData(Fed_rpca_v2, slot = "counts"))

median_umi <- meta %>%
 summarise(median_UMI = median(nUMI))
print(median_umi)

median_UMI
1 1216.552

mean_umi <- meta %>%
 summarise(mean_UMI = mean(nUMI))
print(mean_umi)

mean_UMI
1 1268.816

median_umi_per_cluster <- meta %>%
 group_by(Q.cell_clusters) %>%
 summarise(median_UMI = median(nUMI)) %>%
 arrange(Q.cell_clusters)
print(median_umi_per_cluster)

# A tibble: 13 × 2
 Q.cell_clusters median_UMI
 <fct> <dbl>
 1 Qx 2104.
 2 QL 2313.
 3 QR 2422.
 4 QL.a 1078.
 5 QR.a 1301.
 6 QL.p 1106.
 7 QR.p 1134.
 8 QL.ap 1217.
 9 QR.ap 1290.
10 QL.pa 1031.
11 QR.pa 1099.
12 Qx.paa 714.
13 Qx.pap 452.

mean_umi_per_cluster <- meta %>%
 group_by(Q.cell_clusters) %>%
 summarise(mean_UMI = mean(nUMI)) %>%
 arrange(Q.cell_clusters)
print(mean_umi_per_cluster)

# A tibble: 13 × 2
 Q.cell_clusters mean_UMI
 <fct> <dbl>
 1 Qx 2170.
 2 QL 2291.
 3 QR 2407.
 4 QL.a 1120.
 5 QR.a 1274.
 6 QL.p 1122.
 7 QR.p 1128.
 8 QL.ap 1213.
 9 QR.ap 1289.
10 QL.pa 1040.
11 QR.pa 1091.
12 Qx.paa 724.
13 Qx.pap 454.

meta$nGene <- Matrix::colSums(GetAssayData(Fed_rpca_v2, slot = "counts") > 0)

median_genes <- meta %>%
 summarise(median_genes = median(nGene))
print(median_genes)

median_genes
1 580

median_genes_per_cluster <- meta %>%
 group_by(Q.cell_clusters) %>%
 summarise(median_genes = median(nGene)) %>%
 arrange(Q.cell_clusters)
print(median_genes_per_cluster)

# A tibble: 13 × 2
 Q.cell_clusters median_genes
 <fct> <dbl>
 1 Qx 930.
 2 QL 1050
 3 QR 1114.
 4 QL.a 595
 5 QR.a 649
 6 QL.p 610.
 7 QR.p 636
 8 QL.ap 531
 9 QR.ap 519
10 QL.pa 560
11 QR.pa 587
12 Qx.paa 421
13 Qx.pap 240

#### - Fig.1_VlnUMI

Fig.1_VlnUMI<-VlnPlot(Fed_rpca_v2, features = "nCount_RNA", group.by = "Q.cell_v2_clusters",cols = c(
 "Qx" = "#7cbb5d",
 "Qx.a" = "#d55735",
 "Qx.ap" = "#ecb24d",
 "Qx.p" = "#5793d7",
 "Qx.pa" = "#b44a93",
 "Qx.paa" = "#377f6f",
 "Qx.pap" = "#28afaa"
)) +
 ylab("UMI count") +
 xlab("Cell identity") +
 NoLegend() +
 ggtitle(NULL) +
 stat_summary(fun = median, geom = "crossbar",
 width = 0.6, color = "#828282", size = 0.8)

Warning: Using `size` aesthetic for lines was deprecated in ggplot2 3.4.0.
ℹ Please use `linewidth` instead.

Fig.1_VlnUMI


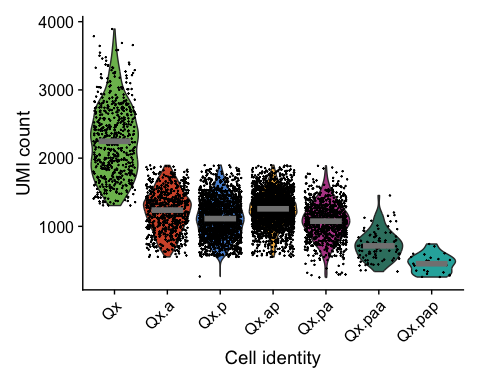


Fig.1_VlnFeature<-VlnPlot(Fed_rpca_v2, features = "nFeature_RNA", group.by = "Q.cell_v2_clusters",cols = c(
 "Qx" = "#7cbb5d",
 "Qx.a" = "#d55735",
 "Qx.ap" = "#ecb24d",
 "Qx.p" = "#5793d7",
 "Qx.pa" = "#b44a93",
 "Qx.paa" = "#377f6f",
 "Qx.pap" = "#28afaa"
)) +
 ylab("Gene count") +
 xlab("Cell identity") +
 NoLegend() +
 ggtitle(NULL) +
 stat_summary(fun = median, geom = "crossbar",
 width = 0.6, color = "#828282", size = 0.8)
Fig.1_VlnFeature


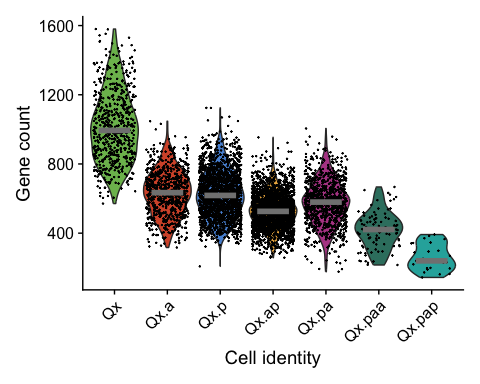


#### – S1 Table - Cell counts in each cluster

table(Idents(Fed_rpca_v2))

Qx QL QR QL.a QR.a QL.ap QR.ap QL.p QR.p QL.pa QR.pa
 272 183 132 231 511 1054 1451 1328 533 284 642
Qx.paa Qx.pap
 97 25

table(Fed_rpca_v2$Q.cell_v2_clusters)

Qx Qx.a Qx.p Qx.ap Qx.pa Qx.paa Qx.pap
 587 742 1861 2505 926 97 25

table(Fed_rpca_v2$experiment_clusters)

OP50 OP50.2 PA14
 1964 2418 2361

# -

# Monocle3 trajectory analysis

Fed_cds <- as.cell_data_set(Fed_rpca_v2)

Warning: Monocle 3 trajectories require cluster partitions, which Seurat does
not calculate. Please run 'cluster_cells' on your cell_data_set object

recreate.partitions <- c(rep(1, length(Fed_cds@colData@rownames)))
names(recreate.partitions) <- Fed_cds@colData@rownames
recreate.partitions <- as.factor(recreate.partitions)

Fed_cds@clusters@listData[["UMAP"]][["partitions"]] <- recreate.partitions

Fed_cds@clusters@listData[["UMAP"]][["clusters"]] <- Fed_rpca_v2@active.ident

Fed_cds@int_colData@listData[["reducedDims"]]@listData[["UMAP.HARM"]]

NULL

Fed_cds@int_colData@listData[["reducedDims"]]@listData[["UMAP"]] <- Fed_rpca_v2@reductions$umap.rpca@cell.embeddings

Fed_cds<- preprocess_cds(Fed_cds, num_dim = 50)

Fed_cds <- learn_graph(Fed_cds, use_partition = F)

|
 | | 0%
 |
 |======================================================================| 100%

plot_cells(Fed_cds, label_groups_by_cluster = F,
 label_branch_points = F, label_roots = F, label_leaves = F,
 trajectory_graph_color = "black", trajectory_graph_segment_size = 1,
 group_label_size = 7, cell_size = 0.9)


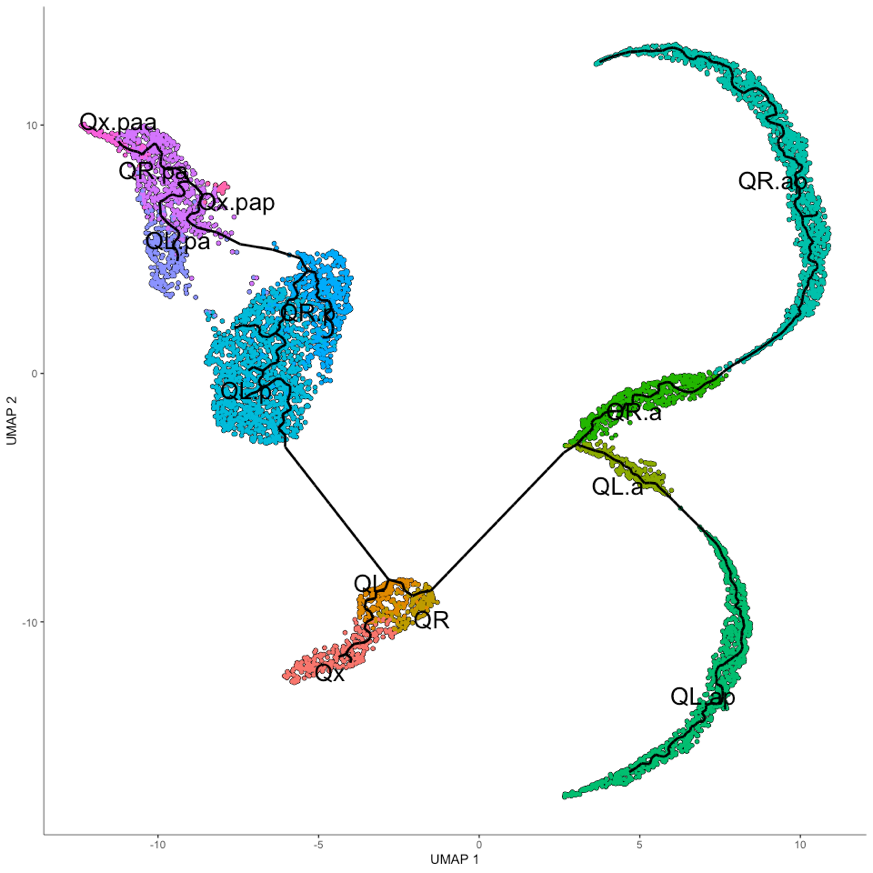


plot_cells(Fed_cds, label_cell_groups = F, label_groups_by_cluster = F,
 label_branch_points = F, label_roots = F, label_leaves = F,
 trajectory_graph_color = "black", trajectory_graph_segment_size = 1,
 group_label_size = 7, cell_size = 0.9) + scale_color_manual(values = cluster_colors) + NoLegend()


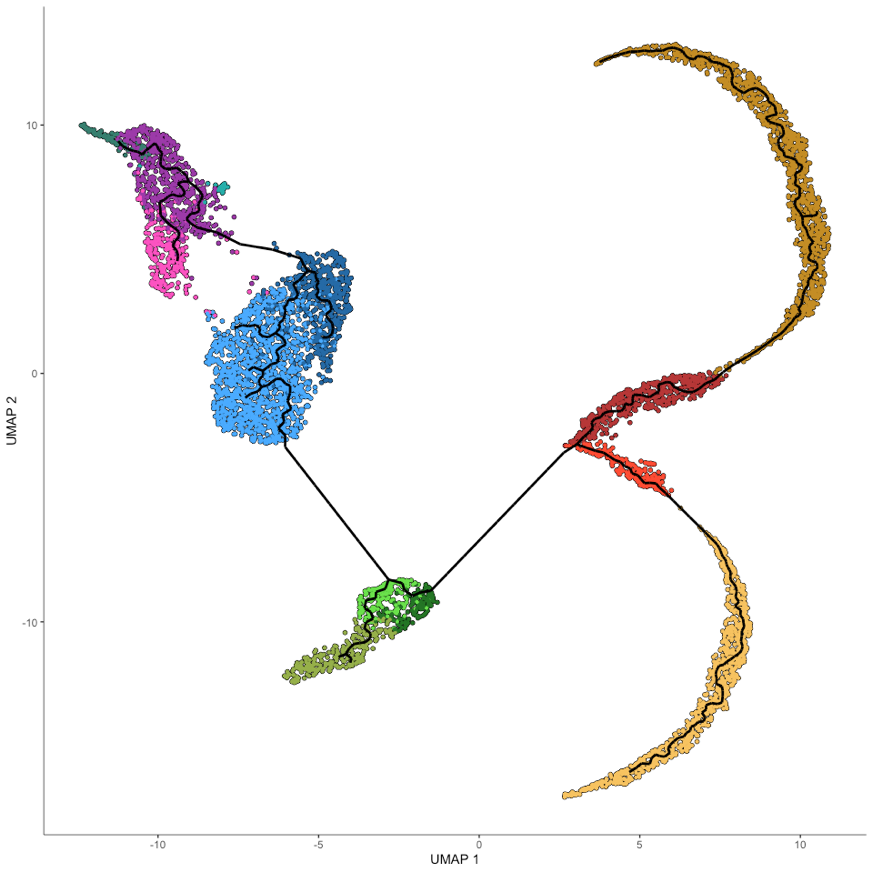


Fed_cds <- order_cells(Fed_cds, reduction_method = "UMAP", root_cells = colnames(Fed_cds[, clusters(Fed_cds) == "Qx"]))

plot_cells(Fed_cds, color_cells_by = "pseudotime", label_groups_by_cluster = T,
 label_branch_points = T, label_roots = F, label_leaves = F) +
 ggtitle("Pseudo-time rooted by Qx")

Cells aren't colored in a way that allows them to be grouped.


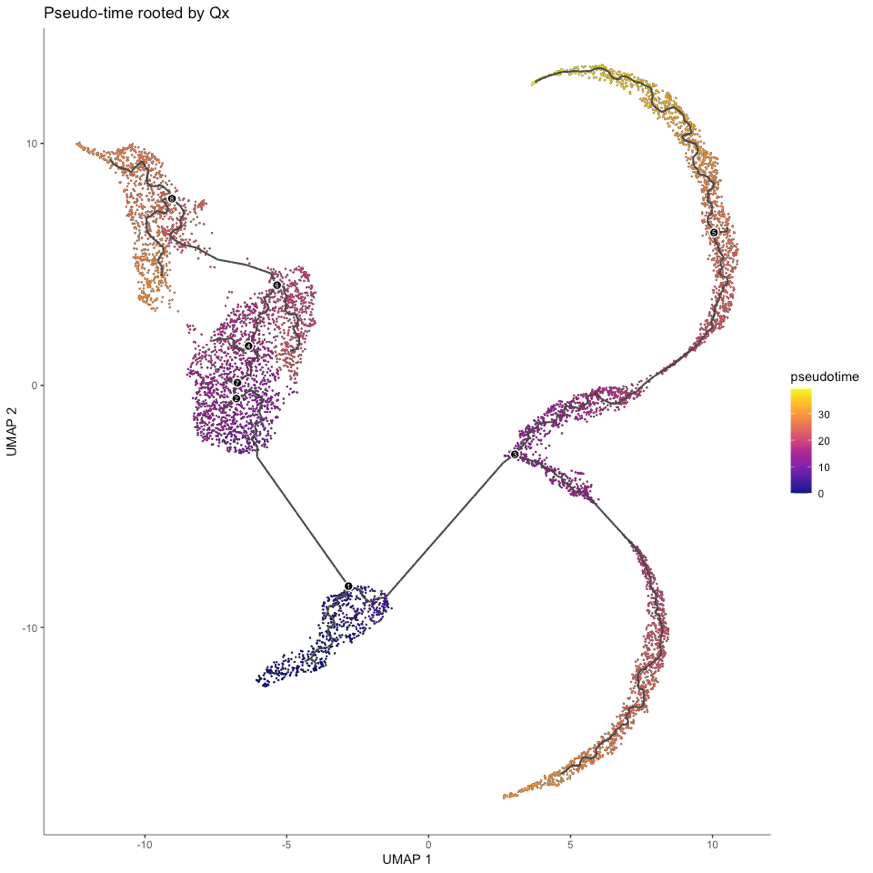


mig21.max <- which.max(unlist(FetchData(Fed_rpca, "mig-21")))
mig21.max <- colnames(Fed_rpca)[mig21.max]
Fed_cds_mig21 <- order_cells(Fed_cds, root_cells = mig21.max)

### - Fig.2_Pseudotime

Fig.2_Pseudotime <- plot_cells(Fed_cds_mig21, color_cells_by = "pseudotime", label_groups_by_cluster = T,
 label_branch_points = T, label_roots = F, label_leaves = F,
 trajectory_graph_color = "green3", trajectory_graph_segment_size = 1,
 graph_label_size = 2.5, cell_size = 0.9 )

Cells aren't colored in a way that allows them to be grouped.

Fig.2_Pseudotime


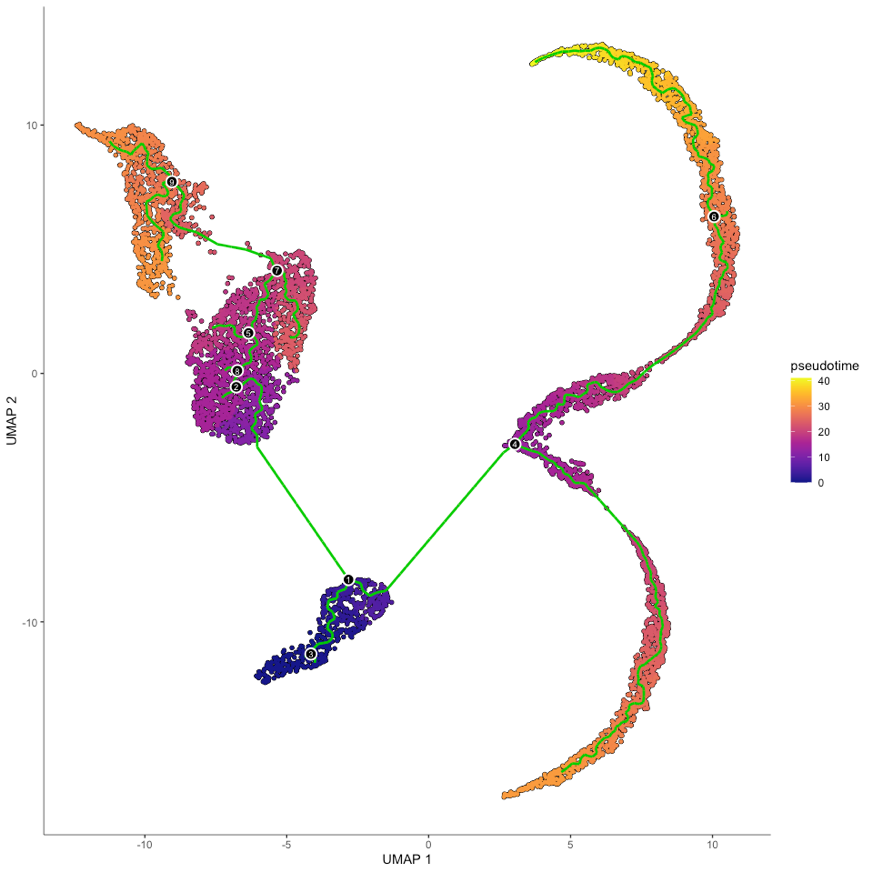


plot_cells(Fed_cds_mig21, color_cells_by = "pseudotime", label_groups_by_cluster = F,
 label_branch_points = F, label_roots = F, label_leaves = F,
 trajectory_graph_color = "green3", trajectory_graph_segment_size = 0,
 graph_label_size = 2.5, cell_size = 0.9, ) +
 ggtitle("Pseudo-time rooted by mig-21 expression")

Cells aren't colored in a way that allows them to be grouped.


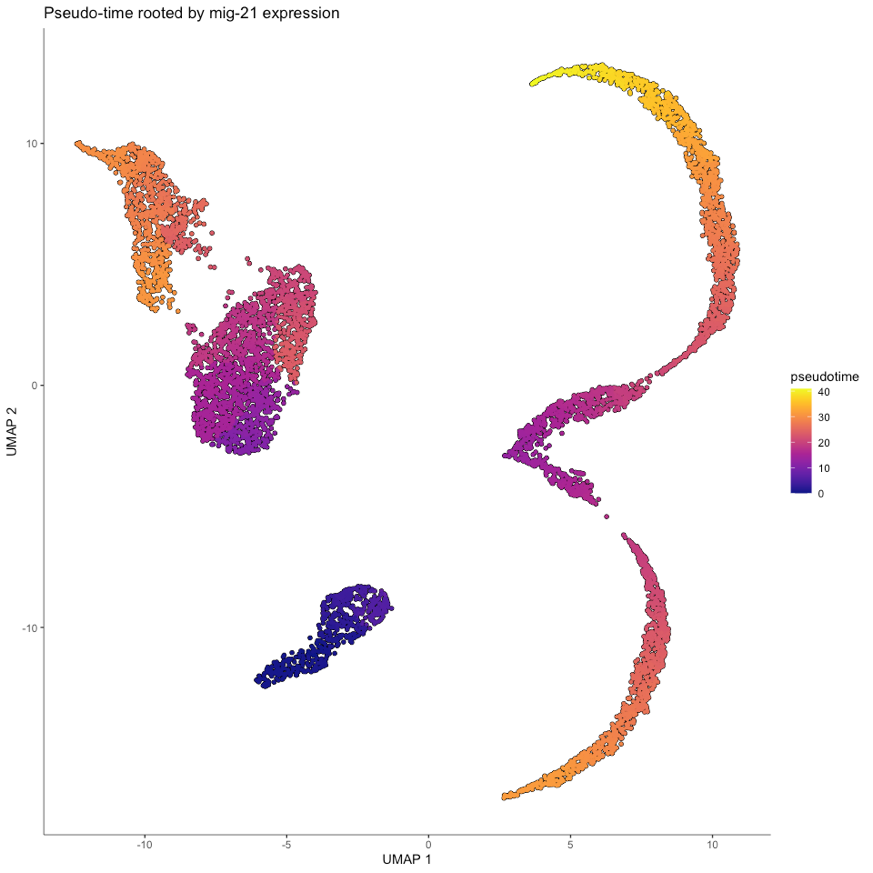


# -

# Gene expression dynamics

## Finding modules of co-regulated genes

**Note:** The find module analysis is stochastic. The module #s may change each time you run the analysis if you don’t set the seed. #### – S2 File

set.seed(319876)
pr_graph_test_res <- graph_test(Fed_cds, neighbor_graph="knn", cores=8)
pr_deg_ids <- row.names(subset(pr_graph_test_res, q_value < 0.05))

gene_module_df_res <- find_gene_modules(Fed_cds[pr_deg_ids,], resolution=5e-3, random_seed = 319876)

write.csv(gene_module_df_res, file = "gene_modules.csv", row.names = FALSE)

cell_group_df <- tibble::tibble(cell=row.names(colData(Fed_cds)),
 # cell_group=partitions(Fed_cds)[colnames(Fed_cds)])
 cell_group=colData(Fed_cds)$Q.cell_clusters)
agg_mat_res <- aggregate_gene_expression(Fed_cds, gene_module_df_res, cell_group_df)
row.names(agg_mat_res) <- stringr::str_c("Module ", row.names(agg_mat_res))

### - Fig.2 Heatmap modules

agg_mat_res_ordered <- agg_mat_res[c("Module 2", "Module 21", "Module 7","Module 12", "Module 5", "Module 9","Module 11", "Module 17", "Module 25","Module 23", "Module 20", "Module 4","Module 1", "Module 14", "Module 19","Module 16", "Module 22", "Module 6","Module 13", "Module 10", "Module 3","Module 18", "Module 8", "Module 24","Module 15"), ]

module_groups_df <- data.frame(
 module_groups = rep(c('Group 1',
 'Group 2',
 'Group 3',
 'Group 4'),
 times = c(4, 10, 2, 9))
)

row.names(module_groups_df) <- rownames(agg_mat_res_ordered)


ann_colors <- list(
 module_groups = c("Group 1" = "#028A0F",
 "Group 2" = "#b44a93",
 "Group 3" = "#c48d25",
 "Group 4" = "#256aa6"))


pheatmap::pheatmap(t(agg_mat_res_ordered),
 scale="row",
 clustering_method="ward.D2",
 angle_col = 45,
 cutree_rows = 4,
 cutree_cols = 4,
 legend_breaks = c(-2, 0, 2),
 legend_labels = c("Low", "Medium", "High"),
 annotation_col = module_groups_df,
 annotation_colors = ann_colors,
 main = "Co-regulated genes",
 col = c("#0065ad", "#2b95e1","#68b3e9","#a4d2f2","#e1f0fb","#fbe6e6","#f3b4b4","#eb8383","#d93e3e","#a11212"),
 # filename = "output/figures/Fig.2_Heatmap_row.pdf",
 width = 12,
 height = 5)


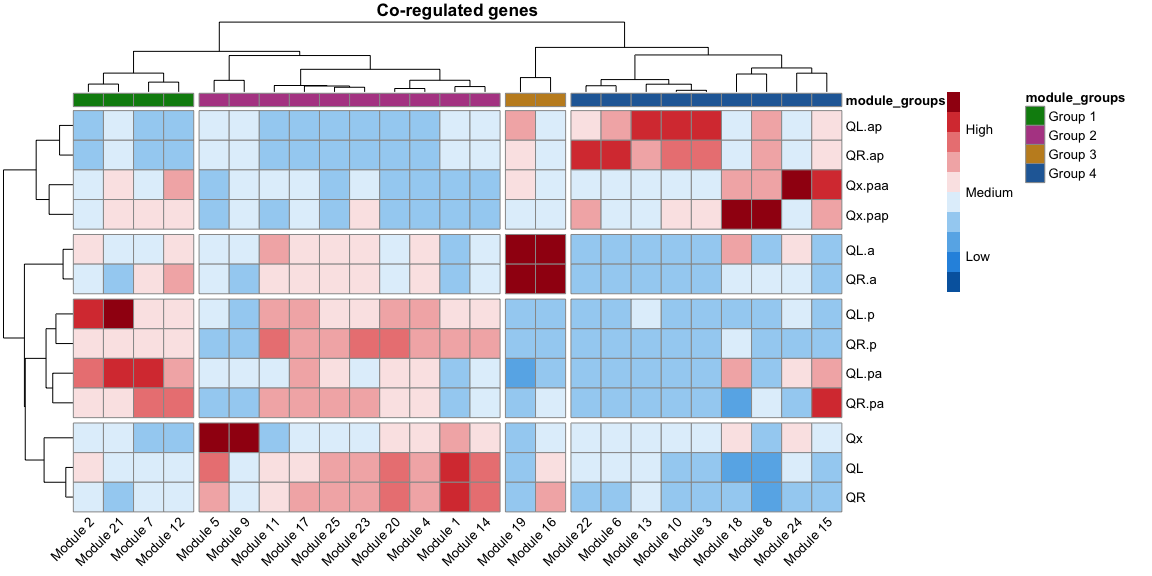


### Defining modules of interest

modules.Qx <- c(5,9)

## TF analysis

Note: We used the list of C. elegans TFs available at AnimalTFDB v4.0 (https://guolab.wchscu.cn/AnimalTFDB4/#/) for this analysis

TF <- read.csv("input/Caenorhabditis_elegans_TF_grouped.csv")
TF_list <- TF$Symbol

TF_list_filtered <- intersect(TF_list, rownames(Fed_rpca_v2))
cat("Number of matching genes:", length(TF_list_filtered),"of", length(TF_list), "\n")

Number of matching genes: 471 of 590

dp_data_TF <- DotPlot(Fed_rpca_v2, features = TF_list_filtered)$data

expressed_TF <- dp_data_TF %>%
 filter(pct.exp > 5) %>%
 arrange(desc(features.plot)) %>%
 pull(features.plot) %>%
 unique()

TF_graph_test_res <- graph_test(Fed_cds[TF_list_filtered, ],
 neighbor_graph = "knn",
 cores = 8)

TF_deg_ids <- rownames(subset(TF_graph_test_res, q_value < 0.05))

TF_modules <- find_gene_modules(Fed_cds[TF_list_filtered, ], resolution = 1e-1)

### Filtering TFs in each module

# Filtering genes expressed in >5% of cells in any cluster
dp_data_TF <- DotPlot(Fed_rpca_v2, features = TF_list_filtered)$data

expressed_TF.5 <- dp_data_TF %>%
 filter(pct.exp > 5) %>%
 arrange(desc(features.plot)) %>%
 pull(features.plot) %>%
 unique()

TF_modules.5 <- gene_module_df_res %>%
 filter(id %in% expressed_TF.5)

TFs.Qx <- TF_modules %>%
 filter(module %in% modules.Qx) %>%
 pull(id) %>%
 unique()

TFs.Qx.5 <- TF_modules.5 %>%
 filter(module %in% modules.Qx) %>%
 pull(id) %>%
 unique()

### - Fig.3_Qx_TFs - DotPlot

Fig.3_Qx_TFs <- DotPlot(Fed_rpca_v2, features = rev(sort(TFs.Qx.5)), dot.min = 0.05) +
 scale_colour_gradientn(colors = c(low = "yellow", mid = "red", high = "black")) +
 theme(
 axis.text.x = element_text(angle = 45, hjust = 1, size = 16),
 axis.text.x.top = element_text(angle = 45, hjust = 0),
 axis.line = element_blank(),
 axis.text.y = element_text(size = 16),
 axis.title.x = element_text(size = 18),
 axis.title.y = element_text(size = 18),
 panel.grid.major = element_line(color = "grey90", size = 0.5),
 panel.background = element_blank(),
 panel.border = element_rect(colour = "black", fill = NA, size = 1)
 ) +
 coord_flip() +
 labs(x = "Transcription factors", y = "Cell identity")

Scale for colour is already present.
Adding another scale for colour, which will replace the existing scale.

Warning: The `size` argument of `element_rect()` is deprecated as of ggplot2 3.4.0.
ℹ Please use the `linewidth` argument instead.

Warning: The `size` argument of `element_line()` is deprecated as of ggplot2 3.4.0.
ℹ Please use the `linewidth` argument instead.

Fig.3_Qx_TFs

Warning: Removed 191 rows containing missing values or values outside the scale range
(`geom_point()`).

# -

# Differential expression analysis

## [Experiment comparison] Checking major markers for each cluster across experiments

Note: Checking if top markers are conserved across different experiments (ignoring L-R) to analyze reproducibility across experiments.

# Adding cluster info to individual experiments
OP50_v3$Q.cell_clusters <- Manual_clustering$Manual_clustering

Warning: Unknown or uninitialised column: `Manual_clustering`.

Warning: Cannot find cell-level meta data named Q.cell_clusters

PA14_v3$Q.cell_clusters <- Manual_clustering$Manual_clustering

Warning: Unknown or uninitialised column: `Manual_clustering`.
Cannot find cell-level meta data named Q.cell_clusters

OP50.2_v4$Q.cell_clusters <- Manual_clustering$Manual_clustering

Warning: Unknown or uninitialised column: `Manual_clustering`.
Cannot find cell-level meta data named Q.cell_clusters

head(OP50_v3)

orig.ident nCount_RNA nFeature_RNA percent.mt
AAACCCATCATCACCC-1 OP50 2059.6005 858 0.04672944
AAACGCTCAAACTCTG-1 OP50 1350.5616 540 0.00000000
AAAGAACAGTTGAAGT-1 OP50 1306.1377 734 0.06843629
AAAGAACCATGGCACC-1 OP50 1122.8362 569 0.25907802
AAAGAACGTTAACAGA-1 OP50 1094.9354 554 0.08323906
AAAGAACTCGATGCAT-1 OP50 264.8511 208 1.12740905
AAAGGATCAGCACGAA-1 OP50 1224.1375 419 0.23276661
AAAGGATGTCAAAGAT-1 OP50 579.5433 255 0.15538629
AAAGGGCCATTCCTCG-1 OP50 1133.1972 445 0.00000000
AAAGGTACAATAGGAT-1 OP50 731.7300 400 0.13162421
 OP50.unintegrated_clusters seurat_clusters
AAACCCATCATCACCC-1 9 9
AAACGCTCAAACTCTG-1 3 3
AAAGAACAGTTGAAGT-1 1 1
AAAGAACCATGGCACC-1 1 1
AAAGAACGTTAACAGA-1 1 1
AAAGAACTCGATGCAT-1 10 10
AAAGGATCAGCACGAA-1 8 8
AAAGGATGTCAAAGAT-1 10 10
AAAGGGCCATTCCTCG-1 7 7
AAAGGTACAATAGGAT-1 0 0

head(PA14_v3)

orig.ident nCount_RNA nFeature_RNA percent.mt
AAACCCAAGCGCCTAC-1 PA14 685.967 443 0.28750346
AAACCCATCCTTGAAG-1 PA14 1160.953 518 0.08164294
AAACGAATCATGCCCT-1 PA14 1296.330 590 0.22217884
AAACGAATCTTCGACC-1 PA14 1558.531 757 0.00000000
AAAGAACAGCAACCAG-1 PA14 1154.794 663 0.34168399
AAAGAACAGGCCTTGC-1 PA14 1445.570 568 0.00000000
AAAGAACCAGGTTCAT-1 PA14 1176.214 502 0.00000000
AAAGGATCATCGCTGG-1 PA14 1459.290 484 0.13178629
AAAGGATTCCCGTTGT-1 PA14 1395.715 527 0.00000000
AAAGGGCCAGCCATTA-1 PA14 1101.236 671 0.00000000
 PA14.unintegrated_clusters seurat_clusters
AAACCCAAGCGCCTAC-1 2 2
AAACCCATCCTTGAAG-1 10 10
AAACGAATCATGCCCT-1 5 5
AAACGAATCTTCGACC-1 3 3
AAAGAACAGCAACCAG-1 0 0
AAAGAACAGGCCTTGC-1 9 9
AAAGAACCAGGTTCAT-1 9 9
AAAGGATCATCGCTGG-1 12 12
AAAGGATTCCCGTTGT-1 12 12
AAAGGGCCAGCCATTA-1 8 8

head(OP50.2_v4)

orig.ident nCount_RNA nFeature_RNA percent.mt
AAACCCAAGACGGTTG-1 OP50.2 2740.6183 1215 0.09390095
AAACGAACAGAGGCTA-1 OP50.2 718.2502 499 0.39522174
AAACGAACAGCAGTTT-1 OP50.2 1128.1375 633 0.00000000
AAACGAAGTCTCTCTG-1 OP50.2 1434.6215 610 0.00000000
AAACGCTTCGCACGGT-1 OP50.2 1220.9162 666 0.00000000
AAAGAACGTTAGCGGA-1 OP50.2 1315.4451 802 0.05390921
AAAGGATTCATTGTTC-1 OP50.2 821.1808 564 0.00000000
AAAGGATTCGGTAACT-1 OP50.2 1498.6509 740 0.24480711
AAAGGGCAGTATTAGG-1 OP50.2 1606.9547 840 0.00000000
AAAGGTAGTATCTCTT-1 OP50.2 1872.8410 1017 0.00000000
 OP50.2.unintegrated_clusters seurat_clusters
AAACCCAAGACGGTTG-1 2 2
AAACGAACAGAGGCTA-1 10 10
AAACGAACAGCAGTTT-1 6 6
AAACGAAGTCTCTCTG-1 16 16
AAACGCTTCGCACGGT-1 7 7
AAAGAACGTTAGCGGA-1 11 11
AAAGGATTCATTGTTC-1 5 5
AAAGGATTCGGTAACT-1 13 13
AAAGGGCAGTATTAGG-1 7 7
AAAGGTAGTATCTCTT-1 1 1

head(Fed_unint_v2)

orig.ident nCount_RNA nFeature_RNA percent.mt
OP50.2_AAACCCAAGACGGTTG-1 OP50.2 2740.6183 1215 0.09390095
OP50.2_AAACGAACAGAGGCTA-1 OP50.2 718.2502 499 0.39522174
OP50.2_AAACGAACAGCAGTTT-1 OP50.2 1128.1375 633 0.00000000
OP50.2_AAACGAAGTCTCTCTG-1 OP50.2 1434.6215 610 0.00000000
OP50.2_AAACGCTTCGCACGGT-1 OP50.2 1220.9162 666 0.00000000
OP50.2_AAAGAACGTTAGCGGA-1 OP50.2 1315.4451 802 0.05390921
OP50.2_AAAGGATTCATTGTTC-1 OP50.2 821.1808 564 0.00000000
OP50.2_AAAGGATTCGGTAACT-1 OP50.2 1498.6509 740 0.24480711
OP50.2_AAAGGGCAGTATTAGG-1 OP50.2 1606.9547 840 0.00000000
OP50.2_AAAGGTAGTATCTCTT-1 OP50.2 1872.8410 1017 0.00000000
 OP50.2.unintegrated_clusters seurat_clusters
OP50.2_AAACCCAAGACGGTTG-1 2 11
OP50.2_AAACGAACAGAGGCTA-1 10 15
OP50.2_AAACGAACAGCAGTTT-1 6 2
OP50.2_AAACGAAGTCTCTCTG-1 16 9
OP50.2_AAACGCTTCGCACGGT-1 7 3
OP50.2_AAAGAACGTTAGCGGA-1 11 7
OP50.2_AAAGGATTCATTGTTC-1 5 6
OP50.2_AAAGGATTCGGTAACT-1 13 7
OP50.2_AAAGGGCAGTATTAGG-1 7 3
OP50.2_AAAGGTAGTATCTCTT-1 1 10
 OP50.unintegrated_clusters PA14.unintegrated_clusters
OP50.2_AAACCCAAGACGGTTG-1 <NA> <NA>
OP50.2_AAACGAACAGAGGCTA-1 <NA> <NA>
OP50.2_AAACGAACAGCAGTTT-1 <NA> <NA>
OP50.2_AAACGAAGTCTCTCTG-1 <NA> <NA>
OP50.2_AAACGCTTCGCACGGT-1 <NA> <NA>
OP50.2_AAAGAACGTTAGCGGA-1 <NA> <NA>
OP50.2_AAAGGATTCATTGTTC-1 <NA> <NA>
OP50.2_AAAGGATTCGGTAACT-1 <NA> <NA>
OP50.2_AAAGGGCAGTATTAGG-1 <NA> <NA>
OP50.2_AAAGGTAGTATCTCTT-1 <NA> <NA>
 unintegrated_clusters cells_to_remove
OP50.2_AAACCCAAGACGGTTG-1 11 Cells_to_keep
OP50.2_AAACGAACAGAGGCTA-1 15 Cells_to_keep
OP50.2_AAACGAACAGCAGTTT-1 2 Cells_to_keep
OP50.2_AAACGAAGTCTCTCTG-1 9 Cells_to_keep
OP50.2_AAACGCTTCGCACGGT-1 3 Cells_to_keep
OP50.2_AAAGAACGTTAGCGGA-1 7 Cells_to_keep
OP50.2_AAAGGATTCATTGTTC-1 6 Cells_to_keep
OP50.2_AAAGGATTCGGTAACT-1 7 Cells_to_keep
OP50.2_AAAGGGCAGTATTAGG-1 3 Cells_to_keep
OP50.2_AAAGGTAGTATCTCTT-1 10 Cells_to_keep

DimPlot(OP50_v3, reduction = "umap.OP50.unintegrated", label = T) +
DimPlot(PA14_v3, reduction = "umap.PA14.unintegrated", label = T) +
DimPlot(OP50.2_v4, reduction = "umap.OP50.2.unintegrated", label = T)

# Assign cell type identity to clusters and merge clusters with same cell type
OP50_v3$Q.cell_clusters <- OP50_v3$seurat_clusters
PA14_v3$Q.cell_clusters <- PA14_v3$seurat_clusters
OP50.2_v4$Q.cell_clusters <- OP50.2_v4$seurat_clusters

levels(OP50_v3$Q.cell_clusters) <- c("Qx.p", "Qx.pa", "Qx.p","Qx.ap", "Qx.ap", "Qx.ap", "Qx.a", "Qx.ap", "Qx.ap", "Qx", "Qx.pa", "Qx.ap", "Qx.pa")
levels(PA14_v3$Q.cell_clusters) <- c("Qx.p", "Qx.ap", "Qx.a", "Qx.pa", "Qx.p", "Qx.ap", "Qx.ap", "Qx.p", "Qx.pa", "Qx.ap", "Qx.ap", "Qx", "Qx.ap")
levels(OP50.2_v4$Q.cell_clusters) <- c("Qx.p", "Qx", "Qx", "Qx.pa", "Qx.ap", "Qx.pa", "Qx.a", "Qx.p", "Qx.ap", "Qx.ap", "Qx.a", "Qx.p", "Qx.a", "Qx.p", "Qx.ap", "Qx.ap", "Qx.ap", "Qx.pa")

DimPlot(OP50_v3, reduction = "umap.OP50.unintegrated", label = T, group.by = "Q.cell_clusters") +
DimPlot(PA14_v3, reduction = "umap.PA14.unintegrated", label = T, group.by = "Q.cell_clusters") +
DimPlot(OP50.2_v4, reduction = "umap.OP50.2.unintegrated", label = T, group.by = "Q.cell_clusters")

# Getting markers
markers.all_OP50_v3 <- FindAllMarkers(OP50_v3, group.by = "Q.cell_clusters", logfc.threshold = 2, return.thresh = 1.0e-20)

Calculating cluster Qx.p

For a (much!) faster implementation of the Wilcoxon Rank Sum Test,
(default method for FindMarkers) please install the presto package
--------------------------------------------
install.packages('devtools')
devtools::install_github('immunogenomics/presto')
--------------------------------------------
After installation of presto, Seurat will automatically use the more
efficient implementation (no further action necessary).
This message will be shown once per session

Calculating cluster Qx.pa

Calculating cluster Qx.ap

Calculating cluster Qx.a

Calculating cluster Qx

markers.all_PA14_v3 <- FindAllMarkers(PA14_v3, group.by = "Q.cell_clusters", logfc.threshold = 2, return.thresh = 1.0e-20)

Calculating cluster Qx.p

Calculating cluster Qx.ap

Calculating cluster Qx.a

Calculating cluster Qx.pa

Calculating cluster Qx

markers.all_OP50.2_v4 <- FindAllMarkers(OP50.2_v4, group.by = "Q.cell_clusters", logfc.threshold = 2, return.thresh = 1.0e-20)

Calculating cluster Qx.p
Calculating cluster Qx

Calculating cluster Qx.pa

Calculating cluster Qx.ap

Calculating cluster Qx.a

markers.all_Fed_rpca_v2 <- FindAllMarkers(Fed_rpca_v2, group.by = "Q.cell_v2_clusters", logfc.threshold = 2, return.thresh = 1.0e-20)

Calculating cluster Qx
Calculating cluster Qx.a

Calculating cluster Qx.p

Calculating cluster Qx.ap

Calculating cluster Qx.pa

Calculating cluster Qx.paa

Calculating cluster Qx.pap

markers.all_experiment.clusters <- FindAllMarkers(Fed_rpca_v2, group.by = "experiment_clusters", logfc.threshold = 2, return.thresh = 1.0e-20, only.pos = T)

Calculating cluster OP50.2

Calculating cluster OP50

Warning in FindMarkers.default(object = data.use, cells.1 = cells.1, cells.2 =
cells.2, : No features pass logfc.threshold threshold; returning empty
data.frame

Calculating cluster PA14

Warning in FindMarkers.default(object = data.use, cells.1 = cells.1, cells.2 =
cells.2, : No features pass logfc.threshold threshold; returning empty
data.frame

head(markers.all_OP50_v3)

p_val avg_log2FC pct.1 pct.2 p_val_adj cluster gene
F32H5.3 2.794463e-265 5.960577 0.783 0.048 2.702525e-261 Qx.p F32H5.3
hphd-1 4.143712e-242 6.169941 0.716 0.037 4.007384e-238 Qx.p hphd-1
shc-2 9.390317e-187 2.683182 0.902 0.302 9.081375e-183 Qx.p shc-2
cyd-1 1.000762e-185 4.277440 0.652 0.060 9.678373e-182 Qx.p cyd-1
ZK930.6 4.766569e-176 2.609151 0.919 0.362 4.609749e-172 Qx.p ZK930.6
syd-9 4.554000e-153 -6.534219 0.033 0.741 4.404173e-149 Qx.p syd-9

head(markers.all_PA14_v3)

p_val avg_log2FC pct.1 pct.2 p_val_adj cluster gene
F32H5.3 0.000000e+00 6.089107 0.761 0.043 0.000000e+00 Qx.p F32H5.3
hphd-1 1.174521e-281 5.739263 0.709 0.037 1.125543e-277 Qx.p hphd-1
cyd-1 1.139299e-243 4.658457 0.670 0.048 1.091790e-239 Qx.p cyd-1
syd-9 1.078537e-215 -6.167610 0.033 0.784 1.033562e-211 Qx.p syd-9
shc-2 2.437331e-195 2.401885 0.876 0.327 2.335694e-191 Qx.p shc-2
ZK930.6 1.079039e-192 2.356024 0.901 0.405 1.034043e-188 Qx.p ZK930.6

head(markers.all_OP50.2_v4)

p_val avg_log2FC pct.1 pct.2 p_val_adj cluster gene
F32H5.3 0.000000e+00 5.399118 0.796 0.069 0.000000e+00 Qx.p F32H5.3
hphd-1 7.374853e-228 4.043105 0.734 0.121 1.120609e-223 Qx.p hphd-1
cyd-1 1.419957e-211 3.107495 0.775 0.152 2.157624e-207 Qx.p cyd-1
shc-2 2.646615e-182 2.511307 0.846 0.299 4.021532e-178 Qx.p shc-2
syd-9 1.125765e-140 -5.900918 0.038 0.644 1.710600e-136 Qx.p syd-9
che-2 4.391121e-139 -5.867639 0.332 0.787 6.672309e-135 Qx.p che-2

head(markers.all_Fed_rpca_v2)

p_val avg_log2FC pct.1 pct.2 p_val_adj cluster gene
R05H11.2 0 4.842942 0.968 0.076 0 Qx R05H11.2
mig-21 0 4.937255 0.963 0.073 0 Qx mig-21
W07A12.4 0 3.141994 0.922 0.129 0 Qx W07A12.4
cpn-1 0 5.025039 0.802 0.038 0 Qx cpn-1
C30F12.5 0 2.556946 0.848 0.140 0 Qx C30F12.5
mcm-6 0 2.804253 0.792 0.098 0 Qx mcm-6

### – S1 File

write.csv(markers.all_OP50_v3, file = "output/csvs/markers.all_OP50_v3.csv", row.names = TRUE)
write.csv(markers.all_PA14_v3, file = "output/csvs/markers.all_PA14_v3.csv", row.names = TRUE)
write.csv(markers.all_OP50.2_v4, file = "output/csvs/markers.all_OP50.2_v4.csv", row.names = TRUE)
write.csv(markers.all_Fed_rpca_v2, file = "output/csvs/markers.all_Fed_rpca_v2.csv", row.names = TRUE)

#Filtering top markers for each cluster
get_top_markers_by_pval <- function(markers_df, top_n = 20) {
 markers_df %>%
 group_by(cluster) %>%
 slice_min(order_by = p_val_adj, n = top_n, with_ties = FALSE) %>%
 arrange(cluster, p_val_adj)
}

top_OP50_v3 <- get_top_markers_by_pval(markers.all_OP50_v3, top_n = 20)
top_PA14_v3 <- get_top_markers_by_pval(markers.all_PA14_v3, top_n = 20)
top_OP50.2_v4 <- get_top_markers_by_pval(markers.all_OP50.2_v4, top_n = 20)
top_Fed_rpca_v2 <- get_top_markers_by_pval(markers.all_Fed_rpca_v2, top_n = 20)
top_experiment.clusters <- get_top_markers_by_pval(markers.all_experiment.clusters, top_n = 20)

top_OP50_v3

# A tibble: 100 × 7
# Groups: cluster [5]
 p_val avg_log2FC pct.1 pct.2 p_val_adj cluster gene
 <dbl> <dbl> <dbl> <dbl> <dbl> <fct> <chr>
 1 2.79e-265 5.96 0.783 0.048 2.70e-261 Qx.p F32H5.3
 2 4.14e-242 6.17 0.716 0.037 4.01e-238 Qx.p hphd-1
 3 9.39e-187 2.68 0.902 0.302 9.08e-183 Qx.p shc-2
 4 1.00e-185 4.28 0.652 0.06 9.68e-182 Qx.p cyd-1
 5 4.77e-176 2.61 0.919 0.362 4.61e-172 Qx.p ZK930.6
 6 4.55e-153 -6.53 0.033 0.741 4.40e-149 Qx.p syd-9
 7 1.96e-142 -7.62 0.221 0.778 1.90e-138 Qx.p che-2
 8 5.76e-141 -8.31 0.057 0.714 5.57e-137 Qx.p C27A2.8
 9 1.09e-137 -6.59 0.041 0.699 1.05e-133 Qx.p gcy-36
10 8.17e-133 -7.16 0.026 0.676 7.90e-129 Qx.p tmem-231
# ℹ 90 more rows

top_PA14_v3

# A tibble: 100 × 7
# Groups: cluster [5]
 p_val avg_log2FC pct.1 pct.2 p_val_adj cluster gene
 <dbl> <dbl> <dbl> <dbl> <dbl> <fct> <chr>
 1 0 6.09 0.761 0.043 0 Qx.p F32H5.3
 2 1.17e-281 5.74 0.709 0.037 1.13e-277 Qx.p hphd-1
 3 1.14e-243 4.66 0.67 0.048 1.09e-239 Qx.p cyd-1
 4 1.08e-215 -6.17 0.033 0.784 1.03e-211 Qx.p syd-9
 5 2.44e-195 2.40 0.876 0.327 2.34e-191 Qx.p shc-2
 6 1.08e-192 2.36 0.901 0.405 1.03e-188 Qx.p ZK930.6
 7 9.68e-190 -9.69 0.026 0.717 9.28e-186 Qx.p C27A2.8
 8 8.06e-177 -7.85 0.198 0.761 7.72e-173 Qx.p che-2
 9 8.15e-176 -8.24 0.021 0.681 7.81e-172 Qx.p gcy-36
10 1.30e-172 -7.77 0.022 0.675 1.25e-168 Qx.p tmem-231
# ℹ 90 more rows

top_OP50.2_v4

# A tibble: 100 × 7
# Groups: cluster [5]
 p_val avg_log2FC pct.1 pct.2 p_val_adj cluster gene
 <dbl> <dbl> <dbl> <dbl> <dbl> <fct> <chr>
 1 0 5.40 0.796 0.069 0 Qx.p F32H5.3
 2 7.37e-228 4.04 0.734 0.121 1.12e-223 Qx.p hphd-1
 3 1.42e-211 3.11 0.775 0.152 2.16e-207 Qx.p cyd-1
 4 2.65e-182 2.51 0.846 0.299 4.02e-178 Qx.p shc-2
 5 1.13e-140 -5.90 0.038 0.644 1.71e-136 Qx.p syd-9
 6 4.39e-139 -5.87 0.332 0.787 6.67e-135 Qx.p che-2
 7 1.82e-126 -7.33 0.022 0.591 2.77e-122 Qx.p gcy-36
 8 8.04e-119 -3.42 0.113 0.667 1.22e-114 Qx.p Y7A9D.1
 9 2.31e-113 -6.01 0.019 0.552 3.51e-109 Qx.p egl-46
10 3.00e-112 2.88 0.56 0.153 4.55e-108 Qx.p pezo-1
# ℹ 90 more rows

top_Fed_rpca_v2

# A tibble: 140 × 7
# Groups: cluster [7]
 p_val avg_log2FC pct.1 pct.2 p_val_adj cluster gene
 <dbl> <dbl> <dbl> <dbl> <dbl> <fct> <chr>
 1 0 4.84 0.968 0.076 0 Qx R05H11.2
 2 0 4.94 0.963 0.073 0 Qx mig-21
 3 0 3.14 0.922 0.129 0 Qx W07A12.4
 4 0 5.03 0.802 0.038 0 Qx cpn-1
 5 0 2.56 0.848 0.14 0 Qx C30F12.5
 6 0 2.80 0.792 0.098 0 Qx mcm-6
 7 0 2.56 0.925 0.235 0 Qx rpl-31
 8 0 4.42 0.721 0.032 0 Qx suro-1
 9 0 2.61 0.859 0.177 0 Qx rpl-30
10 0 2.69 0.784 0.104 0 Qx mcm-2
# ℹ 130 more rows

top_experiment.clusters

# A tibble: 20 × 7
# Groups: cluster [1]
 p_val avg_log2FC pct.1 pct.2 p_val_adj cluster gene
 <dbl> <dbl> <dbl> <dbl> <dbl> <fct> <chr>
 1 0 12.2 0.975 0.001 0 OP50.2 lec-9
 2 0 12.1 0.968 0.001 0 OP50.2 C17F4.7
 3 0 5.95 0.812 0.035 0 OP50.2 ram-2
 4 0 11.1 0.758 0 0 OP50.2 Y119D3B.21
 5 0 11.6 0.722 0 0 OP50.2 lec-10
 6 0 5.26 0.653 0.048 0 OP50.2 col-107
 7 0 14.4 0.494 0 0 OP50.2 C16B8.3
 8 0 9.96 0.465 0 0 OP50.2 nspg-10
 9 0 11.6 0.464 0 0 OP50.2 lec-8
10 0 14.1 0.45 0 0 OP50.2 dod-19
11 0 14.1 0.433 0 0 OP50.2 nspg-14
12 0 4.07 0.451 0.025 0 OP50.2 nspg-9
13 0 14.1 0.418 0 0 OP50.2 fip-2
14 0 8.93 0.406 0.001 0 OP50.2 ftn-2
15 0 13.8 0.368 0 0 OP50.2 T07H8.11
16 0 13.7 0.353 0 0 OP50.2 C08E3.1
17 0 13.6 0.334 0 0 OP50.2 C14C6.5
18 0 13.5 0.318 0 0 OP50.2 F46G10.1
19 0 13.6 0.314 0 0 OP50.2 T03F1.11
20 0 13.4 0.301 0 0 OP50.2 F01D5.5

## Identifying major cell markers for each cluster

### – S5 File - All markers table

markers.all_Q.clusters <- FindAllMarkers(Fed_rpca_v2, group.by = "Q.cell_clusters", logfc.threshold = 2, return.thresh = 1.0e-20)

Calculating cluster Qx

Calculating cluster QL

Calculating cluster QR

Calculating cluster QL.a

Calculating cluster QR.a

Calculating cluster QL.p

Calculating cluster QR.p

Calculating cluster QL.ap

Calculating cluster QR.ap

Calculating cluster QL.pa

Calculating cluster QR.pa

Calculating cluster Qx.paa

Calculating cluster Qx.pap

markers.all_Q.clusters_noLR <- FindAllMarkers(Fed_rpca_v2, group.by = "Q.cell_v2_clusters", logfc.threshold = 2, return.thresh = 1.0e-20)

Calculating cluster Qx

Calculating cluster Qx.a

Calculating cluster Qx.p

Calculating cluster Qx.ap

Calculating cluster Qx.pa

Calculating cluster Qx.paa

Calculating cluster Qx.pap

markers.all_Q.clusters_noLR_onlyPos <- FindAllMarkers(Fed_rpca_v2, group.by = "Q.cell_v2_clusters", logfc.threshold = 2, return.thresh = 1.0e-20, only.pos = T)

Calculating cluster Qx

Calculating cluster Qx.a

Calculating cluster Qx.p

Calculating cluster Qx.ap

Calculating cluster Qx.pa

Calculating cluster Qx.paa

Calculating cluster Qx.pap

markers.all_Q.clusters_HighRes <- FindAllMarkers(Fed_rpca_v2, group.by = "Q.cell_v5_clusters", logfc.threshold = 2, return.thresh = 1.0e-20)

Calculating cluster Qx.1

Calculating cluster Qx.2

Calculating cluster Qx.3

Calculating cluster Qx.a.1

Calculating cluster Qx.a.2

Calculating cluster Qx.p.1

Calculating cluster Qx.p.2

Calculating cluster Qx.ap.1

Calculating cluster Qx.ap.2

Calculating cluster Qx.ap.3

Calculating cluster Qx.ap.4

Calculating cluster Qx.pa

Calculating cluster Qx.paa

Calculating cluster Qx.pap

write.csv(markers.all_Q.clusters_noLR, file = "output/csvs/markers.all_Q.clusters_noLR.csv", row.names = TRUE)
write.csv(markers.all_Q.clusters_noLR_onlyPos, file = "output/csvs/markers.all_Q.clusters_noLR_onlyPos.csv", row.names = TRUE)

### – S5 File - All markers filtered

top_Q.clusters <- get_top_markers_by_pval(markers.all_Q.clusters, top_n = 10)
top_Q.clusters <- top_Q.clusters %>%
 mutate(cluster = factor(cluster, levels = cluster_order)) %>%
 arrange(cluster)

top_Q.clusters_noLR <- get_top_markers_by_pval(markers.all_Q.clusters_noLR, top_n = 10)
top_Q.clusters_noLR <- top_Q.clusters_noLR %>%
 mutate(cluster = factor(cluster, levels = cluster_order_v2.2)) %>%
 arrange(cluster)

top_Q.clusters_noLR_onlyPos <- get_top_markers_by_pval(markers.all_Q.clusters_noLR_onlyPos, top_n = 10)
top_Q.clusters_noLR_onlyPos <- top_Q.clusters_noLR_onlyPos %>%
 mutate(cluster = factor(cluster, levels = cluster_order_v2.2)) %>%
 arrange(cluster)

write.csv(top_Q.clusters_noLR, file = "output/csvs/top_Q.clusters_noLR.csv", row.names = TRUE)
write.csv(top_Q.clusters_noLR_onlyPos, file = "output/csvs/top_Q.clusters_noLR_onlyPos.csv", row.names = TRUE)

DoHeatmap(Fed_rpca_v2, features = top_Q.clusters$gene)+ scale_fill_gradient2( low = rev(c('#d1e5f0','#67a9cf','#2166ac')), mid = "white", high = rev(c('#b2182b','#ef8a62','#fddbc7')), midpoint = 0, guide = "colourbar", aesthetics = "fill") + theme(text = element_text(size = 15))

Scale for fill is already present.
Adding another scale for fill, which will replace the existing scale.

### - Fig.5_Heatmap

Fig.5_Heatmap<-DoHeatmap(Fed_rpca_v2,
 features = top_Q.clusters_noLR$gene,
 group.by = "Q.cell_v2_clusters",
 group.colors = cluster_colors_v2) +
 scale_fill_gradient2(low = rev(c('#a4d2f2','#68b3e9','#2166ac')),
 mid = "white",
 high = rev(c('#b2182b','#eb8383','#f3b4b4')),
 midpoint = 0,
 guide = "colourbar",
 aesthetics = "fill") +
 theme(text = element_text(size = 18),
 axis.text.y = element_text(face = "italic"))

Scale for fill is already present.
Adding another scale for fill, which will replace the existing scale.

Fig.5_Heatmap

### – S9 Fig - Heatmap

Fig.S9_Heatmap<-DoHeatmap(Fed_rpca_v2,
 features = top_Q.clusters_noLR$gene,
 group.by = "Q.cell_clusters",
 group.colors = cluster_colors) +
 scale_fill_gradient2(low = rev(c('#a4d2f2','#68b3e9','#2166ac')),
 mid = "white",
 high = rev(c('#b2182b','#eb8383','#f3b4b4')),
 midpoint = 0,
 guide = "colourbar",
 aesthetics = "fill") +
 theme(text = element_text(size = 18),
 axis.text.y = element_text(face = "italic"))

Scale for fill is already present.
Adding another scale for fill, which will replace the existing scale.

Fig.S9_Heatmap

## Lineage comparisons

### – S3 File

markers.EarlyQ<-FindMarkers(Fed_rpca_v2, ident.1 = "Qx", ident.2 = c("QL", "QR"))
head(markers.EarlyQ)

p_val avg_log2FC pct.1 pct.2 p_val_adj
ctsa-1.1 3.302936e-71 -1.600480 0.934 1.000 5.062740e-67
Y54H5A.2 3.129898e-66 -3.382527 0.235 0.886 4.797508e-62
his-24 2.800027e-62 -2.706991 0.721 0.981 4.291882e-58
tbx-2 1.576825e-57 -2.484781 0.312 0.921 2.416957e-53
hmg-1.1 3.908075e-52 -1.307138 0.893 0.997 5.990297e-48
hil-2 5.337372e-52 -2.068912 0.757 0.981 8.181124e-48

write.csv(markers.EarlyQ, file = "output/csvs/markers_EarlyQ.csv", row.names = TRUE)

#### - Fig.3_QVolcano

keyvals2 <- ifelse(
 markers.EarlyQ$p_val_adj > 10e-6, 'grey' ,
 ifelse (markers.EarlyQ$avg_log2FC < -1, 'purple',
 ifelse(markers.EarlyQ$avg_log2FC > 1, 'darkgreen',
 'black')))
 #keyvals[is.na(keyvals)] <- 'black'
 names(keyvals2)[keyvals2 == 'purple'] <- 'Enriched in late Q cells'
 names(keyvals2)[keyvals2 == 'darkgreen'] <- 'Enriched in early Q cells'
 #names(keyvals)[keyvals == 'black']

markers.EarlyQ$gene_label <- paste0("italic('", rownames(markers.EarlyQ), "')")
Fig.3_QVolcano <- EnhancedVolcano(markers.EarlyQ,
 lab = markers.EarlyQ$gene_label,
 x = "avg_log2FC",
 y = "p_val_adj",
 labSize = 4,
 FCcutoff = 1,
 pointSize = 3.5,
 drawConnectors = T,
 widthConnectors = 0.5,
 typeConnectors = 'open',
 endsConnectors = 'last',
 colCustom = keyvals2,
 lengthConnectors = unit(0.005, "npc"),
 title = NULL,
 parseLabels = TRUE,
 max.overlaps = 5) +
 labs(subtitle = NULL)

Fig.3_QVolcano

Warning: ggrepel: 84 unlabeled data points (too many overlaps). Consider
increasing max.overlaps

### – S5 File - Q.a vs Q.p

markers.Qx.a_vs_Qx.p<-FindMarkers(Fed_rpca_v2, ident.1 = c("QL.a", "QR.a"), ident.2 = c("QL.p", "QR.p"))
head(markers.Qx.a_vs_Qx.p)

p_val avg_log2FC pct.1 pct.2 p_val_adj
lin-32 0 6.997895 0.969 0.067 0
ham-1 0 8.027733 0.889 0.009 0
unc-86 0 -5.894150 0.078 0.951 0
egl-46 0 7.520783 0.879 0.014 0
egl-13 0 7.791428 0.852 0.013 0
Y7A9D.1 0 5.048465 0.880 0.073 0

write.csv(markers.Qx.a_vs_Qx.p, file = "output/csvs/markers.Qx.a_vs_Qx.p.csv", row.names = TRUE)

#### - Fig.5_APVolcano

keyvals3 <- ifelse(
 markers.Qx.a_vs_Qx.p$p_val_adj > 10e-6, 'grey' ,
 ifelse (markers.Qx.a_vs_Qx.p$avg_log2FC < -1, '#5793d7',
 ifelse(markers.Qx.a_vs_Qx.p$avg_log2FC > 1, '#d55735',
 'black')))
 #keyvals[is.na(keyvals)] <- 'black'
 names(keyvals3)[keyvals3 == '#5793d7'] <- 'Enriched in Qx.p '
 names(keyvals3)[keyvals3 == '#d55735'] <- 'Enriched in Qx.a '
 #names(keyvals)[keyvals == 'black']


markers.Qx.a_vs_Qx.p$gene_label <- paste0("italic('", rownames(markers.Qx.a_vs_Qx.p), "')")

Fig.5_APVolcano <- EnhancedVolcano(markers.Qx.a_vs_Qx.p,
 lab = markers.Qx.a_vs_Qx.p$gene_label,
 x = "avg_log2FC",
 y = "p_val_adj",
 labSize = 4,
 FCcutoff = 1,
 pointSize = 3,
 drawConnectors = TRUE,
 widthConnectors = 0.5,
 typeConnectors = 'open',
 endsConnectors = 'last',
 colCustom = keyvals3,
 lengthConnectors = unit(0.005, "npc"),
 title = NULL,
 parseLabels = TRUE,
 max.overlaps = 7) +
 labs(subtitle = NULL)

Warning: One or more p-values is 0. Converting to 10^-1 * current lowest
non-zero p-value...

Fig.5_APVolcano

Warning: ggrepel: 485 unlabeled data points (too many overlaps). Consider
increasing max.overlaps

### Q.d vs Q

markers.Qx.d_vs_Qx<-FindMarkers(Fed_rpca_v2, ident.1 = c("QL.p", "QR.p", "QL.a", "QR.a"), ident.2 = c("Qx", "QL", "QR"))
head(markers.Qx.d_vs_Qx)

p_val avg_log2FC pct.1 pct.2 p_val_adj
R05H11.2 0.000000e+00 -3.882637 0.141 0.968 0.000000e+00
mig-21 0.000000e+00 -3.942659 0.138 0.963 0.000000e+00
cpn-1 0.000000e+00 -4.473987 0.054 0.802 0.000000e+00
unc-54 0.000000e+00 -4.625238 0.395 0.995 0.000000e+00
GFP 1.436401e-296 -5.417413 0.018 0.559 2.201715e-292
egl-17 5.259736e-295 -3.727324 0.044 0.668 8.062124e-291

EnhancedVolcano(markers.Qx.d_vs_Qx,
 rownames(markers.Qx.d_vs_Qx),
 x ="avg_log2FC",
 y ="p_val_adj", labSize = 4, FCcutoff = 1,
 pointSize = 3,
 title = 'Qx.d vs Qx')

Warning: One or more p-values is 0. Converting to 10^-1 * current lowest
non-zero p-value...

### A vs P

markers.A_P<-FindMarkers(Fed_rpca_v2, ident.1 = c("QL.a","QR.a"
 # ,"QL.ap", "QR.ap"
 ), ident.2 = c("QL.p","QL.pa","QR.p", "QR.pa"
# , "Qx.paa", "Qx.pap"
 ))
head(markers.A_P)

p_val avg_log2FC pct.1 pct.2 p_val_adj
lin-32 0 7.301624 0.969 0.053 0
ham-1 0 8.240703 0.889 0.009 0
unc-86 0 -5.781250 0.078 0.944 0
egl-13 0 7.887843 0.852 0.013 0
gcy-36 0 7.067759 0.791 0.021 0
Y7A9D.1 0 4.118255 0.880 0.118 0

keyvals <- ifelse(
 markers.A_P$p_val_adj > 10e-6, 'grey' ,
 ifelse (markers.A_P$avg_log2FC < -1, 'royalblue',
 ifelse(markers.A_P$avg_log2FC > 1, 'red',
 'black')))
 #keyvals[is.na(keyvals)] <- 'black'
 names(keyvals)[keyvals == 'red'] <- 'Enriched on the anterior lineage'
 #names(keyvals)[keyvals == 'black']
 names(keyvals)[keyvals == 'royalblue'] <- 'Enriched on the posterior lineage'


EnhancedVolcano(markers.A_P, rownames(markers.A_P), x ="avg_log2FC", y ="p_val_adj",
 #selectLab = rownames(Left.markers)[which(names(keyvals) %in% c('high', 'low'))],
 labSize = 5, FCcutoff = 1, pointSize = 4, #pCutoff = 10e-50,
 title = 'Anterior vs Posterior',
 drawConnectors = TRUE,
 widthConnectors = 0.5,
 typeConnectors = 'open',
 endsConnectors = 'last',
 colCustom = keyvals,
 lengthConnectors = unit(0.005, "npc"))

Warning: One or more p-values is 0. Converting to 10^-1 * current lowest
non-zero p-value...

Warning: ggrepel: 340 unlabeled data points (too many overlaps). Consider
increasing max.overlaps

## Volcano plots - Left vs right

### – S4 File - Qx

markers.QL<-FindMarkers(Fed_rpca_v2, ident.1 = "QL", ident.2 = "QR")

write.csv(markers.QL, file = "output/csvs/markers.QL.csv", row.names = TRUE)

#### - Fig.4_QxVolcano

keyvals <- ifelse(
 markers.QL$p_val_adj > 10e-6, 'grey' ,
 ifelse (markers.QL$avg_log2FC < -1, 'royalblue',
 ifelse(markers.QL$avg_log2FC > 1, 'red',
 'black')))
 names(keyvals)[keyvals == 'red'] <- 'Enriched in QL'
 names(keyvals)[keyvals == 'royalblue'] <- 'Enriched in QR'

markers.QL$gene_label <- paste0("italic('", rownames(markers.QL), "')")
Fig.4_QxVolcano <-EnhancedVolcano(markers.QL,
 lab= markers.QL$gene_label,
 x ="avg_log2FC",
 y ="p_val_adj",
 labSize = 6,
 FCcutoff = 1,
 pointSize = 3,
 drawConnectors = TRUE,
 widthConnectors = 0.5,
 typeConnectors = 'open',
 endsConnectors = 'last',
 colCustom = keyvals,
 lengthConnectors = unit(0.005, "npc"),
 title = NULL,
 parseLabels = TRUE,
 max.overlaps = 5) +
 labs(subtitle = NULL)

Fig.4_QxVolcano

Warning: ggrepel: 1 unlabeled data points (too many overlaps). Consider
increasing max.overlaps

### –S4 File - Qx.a

markers.QL.a<-FindMarkers(Fed_rpca_v2, ident.1 = "QL.a", ident.2 = "QR.a")

write.csv(markers.QL.a, file = "output/csvs/markers.QL.a.csv", row.names = TRUE)

### -Fig.4_Qx.aVolcano

keyvals <- ifelse(
 markers.QL.a$p_val_adj > 10e-6, 'grey' ,
 ifelse (markers.QL.a$avg_log2FC < -1, 'royalblue',
 ifelse(markers.QL.a$avg_log2FC > 1, 'red',
 'black')))
 names(keyvals)[keyvals == 'red'] <- 'Enriched in QL.a'
 names(keyvals)[keyvals == 'royalblue'] <- 'Enriched in QR.a'

markers.QL.a$gene_label <- paste0("italic('", rownames(markers.QL.a), "')")
Fig.4_Qx.aVolcano <- EnhancedVolcano(markers.QL.a,
 lab= markers.QL.a$gene_label,
 x ="avg_log2FC",
 y ="p_val_adj",
 labSize = 6,
 FCcutoff = 1,
 pointSize = 3,
 drawConnectors = TRUE,
 widthConnectors = 0.5,
 typeConnectors = 'open',
 endsConnectors = 'last',
 colCustom = keyvals,
 lengthConnectors = unit(0.005, "npc"),
 title = NULL,
 parseLabels = TRUE,
 max.overlaps = 4) +
 labs(subtitle = NULL)

Fig.4_Qx.aVolcano

Warning: ggrepel: 18 unlabeled data points (too many overlaps). Consider
increasing max.overlaps

### –S4 File - Qx.p

markers.QL.p<-FindMarkers(Fed_rpca_v2, ident.1 = "QL.p", ident.2 = "QR.p")

write.csv(markers.QL.p, file = "output/csvs/markers.QL.p.csv", row.names = TRUE)

### -Fig.4_Qx.pVolcano

keyvals <- ifelse(
 markers.QL.p$p_val_adj > 10e-6, 'grey' ,
 ifelse (markers.QL.p$avg_log2FC < -1, 'royalblue',
 ifelse(markers.QL.p$avg_log2FC > 1, 'red',
 'black')))
 names(keyvals)[keyvals == 'red'] <- 'Enriched in QL.p'
 names(keyvals)[keyvals == 'royalblue'] <- 'Enriched in QR.p'

markers.QL.p$gene_label <- paste0("italic('", rownames(markers.QL.p), "')")

Fig.4_Qx.pVolcano<-EnhancedVolcano(markers.QL.p,
 lab= markers.QL.p$gene_label,
 x ="avg_log2FC",
 y ="p_val_adj",
 labSize = 6,
 FCcutoff = 1,
 pointSize = 3,
 drawConnectors = TRUE,
 widthConnectors = 0.5,
 typeConnectors = 'open',
 endsConnectors = 'last',
 colCustom = keyvals,
 lengthConnectors = unit(0.005, "npc"),
 title = NULL,
 parseLabels = TRUE,
 max.overlaps = 4) +
 labs(subtitle = NULL)


Fig.4_Qx.pVolcano

Warning: ggrepel: 61 unlabeled data points (too many overlaps). Consider
increasing max.overlaps

### –S4 File - Qx.ap

markers.QL.ap<-FindMarkers(Fed_rpca_v2, ident.1 = "QL.ap", ident.2 = "QR.ap")

write.csv(markers.QL.ap, file = "output/csvs/markers.QL.ap.csv", row.names = TRUE)

### -Fig.4_Qx.apVolcano

keyvals <- ifelse(
 markers.QL.ap$p_val_adj > 10e-6, 'grey' ,
 ifelse (markers.QL.ap$avg_log2FC < -1, 'royalblue',
 ifelse(markers.QL.ap$avg_log2FC > 1, 'red',
 'black')))
 names(keyvals)[keyvals == 'red'] <- 'Enriched in QL.ap'
 names(keyvals)[keyvals == 'royalblue'] <- 'Enriched in QR.ap'

markers.QL.ap$gene_label <- paste0("italic('", rownames(markers.QL.ap), "')")
Fig.4_Qx.apVolcano<-EnhancedVolcano(markers.QL.ap,
 lab= markers.QL.ap$gene_label,
 x ="avg_log2FC",
 y ="p_val_adj",
 labSize = 6,
 FCcutoff = 1,
 pointSize = 3,
 drawConnectors = TRUE,
 widthConnectors = 0.5,
 typeConnectors = 'open',
 endsConnectors = 'last',
 colCustom = keyvals,
 lengthConnectors = unit(0.005, "npc"),
 title = NULL,
 parseLabels = TRUE,
 max.overlaps = 4) +
 labs(subtitle = NULL)


Fig.4_Qx.apVolcano

Warning: ggrepel: 182 unlabeled data points (too many overlaps). Consider
increasing max.overlaps

### –S4 File - Qx.pa

markers.QL.pa<-FindMarkers(Fed_rpca_v2, ident.1 = "QL.pa", ident.2 = "QR.pa")

write.csv(markers.QL.pa, file = "output/csvs/markers.QL.pa.csv", row.names = TRUE)

### -Fig.4_Qx.paVolcano

keyvals <- ifelse(
 markers.QL.pa$p_val_adj > 10e-6, 'grey' ,
 ifelse (markers.QL.pa$avg_log2FC < -1, 'royalblue',
 ifelse(markers.QL.pa$avg_log2FC > 1, 'red',
 'black')))
 names(keyvals)[keyvals == 'red'] <- 'Enriched in QL.pa'
 names(keyvals)[keyvals == 'royalblue'] <- 'Enriched in QR.pa'

markers.QL.pa$gene_label <- paste0("italic('", rownames(markers.QL.pa), "')")

Fig.4_Qx.paVolcano <- EnhancedVolcano(markers.QL.pa,
 lab= markers.QL.pa$gene_label,
 x ="avg_log2FC",
 y ="p_val_adj",
 labSize = 6,
 FCcutoff = 1,
 pointSize = 3,
 drawConnectors = TRUE,
 widthConnectors = 0.5,
 typeConnectors = 'open',
 endsConnectors = 'last',
 colCustom = keyvals,
 lengthConnectors = unit(0.005, "npc"),
 title = NULL,
 parseLabels = TRUE,
 max.overlaps = 4) +
 labs(subtitle = NULL)


Fig.4_Qx.paVolcano

Warning: ggrepel: 88 unlabeled data points (too many overlaps). Consider
increasing max.overlaps

# L vs R
markers.L_R<-FindMarkers(Fed_rpca_v2, ident.1 = c("QL", "QL.a","QL.ap","QL.p", "QL.pa"), ident.2 = c("QR", "QR.a","QR.ap","QR.p", "QR.pa"))
head(markers.L_R)

p_val avg_log2FC pct.1 pct.2 p_val_adj
mab-5 0.000000e+00 4.815000 0.468 0.016 0.000000e+00
F44E5.1 0.000000e+00 -3.272351 0.244 0.650 0.000000e+00
mig-13 0.000000e+00 -7.123884 0.005 0.410 0.000000e+00
lin-39 0.000000e+00 -5.458063 0.010 0.400 0.000000e+00
flp-5 1.062299e-261 -7.318045 0.044 0.394 1.628292e-257
ssq-1 6.260094e-207 -3.696826 0.050 0.359 9.595473e-203

keyvals <- ifelse(
 markers.L_R$p_val_adj > 10e-6, 'grey' ,
 ifelse (markers.L_R$avg_log2FC < -1, 'royalblue',
 ifelse(markers.L_R$avg_log2FC > 1, 'red',
 'black')))
 #keyvals[is.na(keyvals)] <- 'black'
 names(keyvals)[keyvals == 'red'] <- 'Enriched on the QL lineage'
 #names(keyvals)[keyvals == 'black']
 names(keyvals)[keyvals == 'royalblue'] <- 'Enriched on the QR lineage'


EnhancedVolcano(markers.L_R, rownames(markers.L_R), x ="avg_log2FC", y ="p_val_adj",
 #selectLab = rownames(Left.markers)[which(names(keyvals) %in% c('high', 'low'))],
 labSize = 5, FCcutoff = 1, pointSize = 4, #pCutoff = 10e-50,
 title = 'Left vs Right',
 drawConnectors = TRUE,
 widthConnectors = 0.5,
 typeConnectors = 'open',
 endsConnectors = 'last',
 colCustom = keyvals,
 lengthConnectors = unit(0.005, "npc"))

Warning: One or more p-values is 0. Converting to 10^-1 * current lowest
non-zero p-value...

Warning: ggrepel: 290 unlabeled data points (too many overlaps). Consider
increasing max.overlaps

# -

# Gene set analysis

## Known Q markers

### Annotation

FeaturePlot(Fed_rpca_v2, reduction = "umap.rpca",
 features = c("mab-5", "lin-39"),
 label = F, order = T, cols = c("#efefef", "#4011ba"), ncol = 2)

FeaturePlot(Fed_rpca_v2, reduction = "umap.rpca",
 features = c("mig-21"),
 label = F, order = T, cols = c("#efefef", "#4011ba"))

FeaturePlot(Fed_rpca_v2, reduction = "umap.rpca",
 features = c("egl-13", "gcy-32", "gcy-35", "gcy-36"),
 label = F, order = T, cols = c("#efefef", "#4011ba"), ncol = 2)

FeaturePlot(Fed_rpca_v2, reduction = "umap.rpca",
 features = c("mec-7", "mec-18", "mec-3", "lad-2", "ceh-31", "ZK265.7", "ceh-43"),
 label = F, order = T, cols = c("#efefef", "#4011ba"), ncol = 4)

FeaturePlot(Fed_rpca_v2, reduction = "umap.rpca",
 features = c("mab-5", "lin-39", "mig-21", "egl-13", "gcy-32", "mec-7", "mec-3", "lad-2"),
 label = F, order = T, cols = c("#efefef", "#4011ba"), ncol = 4)

## Initial Polarization

DotPlot(Fed_rpca_v2, features = c("mig-21", "cdh-3", "cdh-4", "unc-40", "mig-15", "ptp-3","dpy-19", "dpy-17", "sqt-3", "ced-10","rac-2", "mig-2", "unc-7", "pix-1"), dot.min = 0.05, dot.scale = 10) + scale_colour_gradientn(colors = c(low = "yellow", mid = "red", high = "black")) + theme(axis.text.x = element_text(angle = 60, hjust = 1), axis.text.y = ) + xlab('Gene') + ylab('Cell Cluster') + theme(panel.grid.minor = element_blank(),panel.grid.major = element_line(colour = "lightgray",linetype = "dashed", size=0.15), panel.border = element_rect(colour = "black", fill=NA, size=1)) #+ coord_flip()

Warning: The following requested variables were not found: rac-2

Scale for colour is already present.
Adding another scale for colour, which will replace the existing scale.

Warning: Removed 99 rows containing missing values or values outside the scale range
(`geom_point()`).

## Wnt analysis

Wnt_genes <- c("mom-1", "mig-14", "vps-26", "vps-29", "vps-35", "snx-3", "mom-2", "lin-44", "egl-20",
 "cwn-1", "cwn-2", "mom-5", "cfz-2", "lin-17", "mig-1", "cam-1", "lin-18", "sfrp-1",
 "kin-19", "mig-5", "dsh-1", "dsh-2", "gsk-3", "pry-1", "axl-1", "apr-1", "lin-23",
 "lit-1", "mom-4", "tap-1", "bar-1", "sys-1", "wrm-1", "hmp-2", "pop-1", "unc-37",
 "vang-1", "prkl-1", "fmi-1")

Wnt_genes_italic <- paste0("italic('", Wnt_genes, "')")

### - Fig.6_Wnt

Fig.6_Wnt <- DotPlot(Fed_rpca_v2,
 features = Wnt_genes,
 dot.min = 0.1) +
 scale_colour_gradientn(colors = c(low = "yellow", mid = "red", high = "black")) +
 theme(
 axis.text.x = element_text(angle = 60, hjust = 1),
 axis.text.y = ) +
 xlab('Gene') +
 ylab('Cell Identity') +
 theme(
 panel.grid.minor = element_blank(),
 panel.grid.major = element_line(colour = "lightgray",linetype = "dashed", size=0.15),
 panel.border = element_rect(colour = "black", fill=NA, size=1)) +
 scale_x_discrete(labels = parse(text = Wnt_genes_italic))

Scale for colour is already present.
Adding another scale for colour, which will replace the existing scale.

Fig.6_Wnt

Warning: Removed 327 rows containing missing values or values outside the scale range
(`geom_point()`).

### - Fig.6_Wnt_flip

Fig.6_Wnt_flip <- DotPlot(Fed_rpca_v2,
 features = rev(Wnt_genes),
 dot.min = 0.1,
 dot.scale = 10) +
 scale_colour_gradientn(colors = c(low = "yellow", mid = "red", high = "black")) +
 theme(
 axis.text.x = element_text(angle = 60, hjust = 1),
 axis.text.y = ) +
 xlab('Gene') +
 ylab('Cell Identity') +
 theme(
 panel.grid.minor = element_blank(),
 panel.grid.major = element_line(colour = "lightgray",linetype = "dashed", size=0.15),
 panel.border = element_rect(colour = "black", fill=NA, size=1)) +
 scale_x_discrete(labels = parse(text = rev(Wnt_genes_italic))) +
 coord_flip()

Scale for colour is already present.
Adding another scale for colour, which will replace the existing scale.

Fig.6_Wnt_flip

Warning: Removed 327 rows containing missing values or values outside the scale range
(`geom_point()`).

## Pseudotime plots

rowData(Fed_cds)$gene_name <- rownames(Fed_cds)
rowData(Fed_cds)$gene_short_name <- rowData(Fed_cds)$gene_name

### Q neuroblast polarization/initial migration

pseudo_Qx_pol_genes <- c("mig-21", "cdh-3", "cdh-4", "unc-40", "mig-15", "ptp-3","dpy-19"
 #, "dpy-17", "sqt-3"
 )


pseudo_Qx_pol_cds <- Fed_cds[rowData(Fed_cds)$gene_short_name %in% pseudo_Qx_pol_genes,
 colData(Fed_cds)$Q.cell_clusters %in% c("Qx", "QL", "QR", "QL.a","QR.a", "QL.ap", "QR.ap", "QL.p", "QR.p", "QL.pa", "QR.pa", "Qx.paa", "Qx.pap")]

pseudo_Qx_pol_cds <- pseudo_Qx_pol_cds[,Matrix::colSums(exprs(pseudo_Qx_pol_cds)) != 0]
pseudo_Qx_pol_cds <- estimate_size_factors(pseudo_Qx_pol_cds)


pseudo_Qx_pol_cds <- order_cells(pseudo_Qx_pol_cds, root_cells = mig21.max)

### - Fig.3_Qx_Pseudotime

Fig.3_Qx_Pseudotime<-plot_genes_in_pseudotime(pseudo_Qx_pol_cds, ncol = 1)
Fig.3_Qx_Pseudotime

Warning in scale_y_log10(): log-10 transformation introduced infinite values.
log-10 transformation introduced infinite values.

## Loading Paolillo’s data

Note: Lists of up- and downregulated genes in mab-5 mutants was extracted from Paolillo et al., 2024 (https://doi.org/10.1093/genetics/iyae045) to analyze expression pattern of possible targets of MAB-5

Up.lof <- read.csv("input/Upregulated_lof.csv")
Down.lof <- read.csv("input/Downregulated_lof.csv")
Up.gof <- read.csv("input/Upregulated_gof.csv")
Down.gof <- read.csv("input/Downregulated_gof.csv")

#Up.lof_list <- Up.lof$Symbol

#Up.lof_list_filtered <- intersect(Up.lof_list, rownames(Fed_rpca_v2))
#cat("Number of matching genes:", length(Up.lof_list_filtered),"of", length(Up.lof_list), "\n")

#dp_data_Up.lof <- DotPlot(Fed_rpca_v2, features = Up.lof_list_filtered)$data
dp_data_Up.lof <- DotPlot(Fed_rpca_v2, features = Up.lof)$data

Warning: The following requested variables were not found (10 out of 15 shown):
21ur-2841, C02G6.1, srbc-60, T01B10.13, nhr-39, B0563.18, ugt-38, srj-23,
H39E23.3, Y106G6D.4

Warning: The `facets` argument of `facet_grid()` is deprecated as of ggplot2 2.2.0.
ℹ Please use the `rows` argument instead.
ℹ The deprecated feature was likely used in the Seurat package.
 Please report the issue at <https://github.com/satijalab/seurat/issues>.

dp_data_Down.lof <- DotPlot(Fed_rpca_v2, features = Down.lof)$data

Warning: The following requested variables were not found: T02H6.7, his-56,
F55B12.11

dp_data_Up.gof <- DotPlot(Fed_rpca_v2, features = Up.gof)$data

Warning: The following requested variables were not found (10 out of 32 shown):
21ur-2841, 21ur-39, ZK218.23, sls-1.12, rrn-4.3, sls-1.11, sls-2.4, R160.11,
rrn-4.12, linc-144

dp_data_Down.gof <- DotPlot(Fed_rpca_v2, features = Down.gof)$data

Warning: The following requested variables were not found (10 out of 11 shown):
F58F9.10, cars-2, appg-2, C09F9.7, txt-2, H10D18.6, ZK353.3, Y39A1A.10,
str-139, rrn-3.1

### - Fig.S5_Down.lof - DotPlot 37 genes

Fig.S5_Down.lof <- DotPlot(Fed_rpca_v2,
 features = rev(sort(Down.lof$Symbol)),
 dot.min = 0.1) +
 scale_colour_gradientn(colors = c(low = "yellow", mid = "red", high = "black")) +
 theme(
 axis.text.x = element_text(angle = 45, hjust = 1, size = 16),
 axis.text.x.top = element_text(angle = 45, hjust = 0),
 axis.line = element_blank(),
 axis.text.y = element_text(size = 16),
 axis.title.x = element_text(size = 18),
 axis.title.y = element_text(size = 18),
 panel.grid.major = element_line(color = "grey90", size = 0.5),
 panel.background = element_blank(),
 panel.border = element_rect(colour = "black", fill = NA, size = 1)
 ) +
 coord_flip() +
 labs(x = "Downregulated genes in lof", y = "Cell identity")

Warning: The following requested variables were not found: T02H6.7, his-56,
F55B12.11

Scale for colour is already present.
Adding another scale for colour, which will replace the existing scale.

Fig.S5_Down.lof

Warning: Removed 345 rows containing missing values or values outside the scale range
(`geom_point()`).

### - Fig.S6_Up.lof - DotPlot 59 genes

Fig.S6_Up.lof <- DotPlot(Fed_rpca_v2,
 features = rev(sort(Up.lof$Symbol)),
 dot.min = 0.1) +
 scale_colour_gradientn(colors = c(low = "yellow", mid = "red", high = "black")) +
 theme(
 axis.text.x = element_text(angle = 45, hjust = 1, size = 16),
 axis.text.x.top = element_text(angle = 45, hjust = 0),
 axis.line = element_blank(),
 axis.text.y = element_text(size = 16),
 axis.title.x = element_text(size = 18),
 axis.title.y = element_text(size = 18),
 panel.grid.major = element_line(color = "grey90", size = 0.5),
 panel.background = element_blank(),
 panel.border = element_rect(colour = "black", fill = NA, size = 1)
 ) +
 coord_flip() +
 labs(x = "Upregulated genes in lof", y = "Cell identity")

Warning: The following requested variables were not found (10 out of 15 shown):
ZK218.23, Y80D3A.11, Y106G6D.4, ugt-38, T01B10.13, srj-23, srbc-60, rrn-4.3,
nhr-39, linc-84

Scale for colour is already present.
Adding another scale for colour, which will replace the existing scale.

Fig.S6_Up.lof

Warning: Removed 563 rows containing missing values or values outside the scale range
(`geom_point()`).

### - Fig.S7_Down.gof - DotPlot 91 genes

Fig.S7_Down.gof <- DotPlot(Fed_rpca_v2,
 features = rev(sort(Down.gof$Symbol)),
 dot.min = 0.1) +
 scale_colour_gradientn(colors = c(low = "yellow", mid = "red", high = "black")) +
 theme(
 axis.text.x = element_text(angle = 45, hjust = 1, size = 16),
 axis.text.x.top = element_text(angle = 45, hjust = 0),
 axis.line = element_blank(),
 axis.text.y = element_text(size = 16),
 axis.title.x = element_text(size = 18),
 axis.title.y = element_text(size = 18),
 panel.grid.major = element_line(color = "grey90", size = 0.5),
 panel.background = element_blank(),
 panel.border = element_rect(colour = "black", fill = NA, size = 1)
 ) +
 coord_flip() +
 labs(x = "Downregulated genes in gof", y = "Cell identity")

Warning: The following requested variables were not found (10 out of 11 shown):
ZK353.3, Y39A1A.10, txt-2, str-139, rrn-3.1, lipl-3, H10D18.6, F58F9.10,
cars-2, C09F9.7

Scale for colour is already present.
Adding another scale for colour, which will replace the existing scale.

Fig.S7_Down.gof

Warning: Removed 865 rows containing missing values or values outside the scale range
(`geom_point()`).

### - Fig.S8_Up.gof - DotPlot 178 genes

Fig.S8_Up.gof <- DotPlot(Fed_rpca_v2,
 features = rev(sort(Up.gof$Symbol)),
 dot.min = 0.1) +
 scale_colour_gradientn(colors = c(low = "yellow", mid = "red", high = "black")) +
 theme(
 axis.text.x = element_text(angle = 45, hjust = 1, size = 16),
 axis.text.x.top = element_text(angle = 45, hjust = 0),
 axis.line = element_blank(),
 axis.text.y = element_text(size = 16),
 axis.title.x = element_text(size = 18),
 axis.title.y = element_text(size = 18),
 panel.grid.major = element_line(color = "grey90", size = 0.5),
 panel.background = element_blank(),
 panel.border = element_rect(colour = "black", fill = NA, size = 1)
 ) +
 coord_flip() +
 labs(x = "Upregulated genes in gof", y = "Cell identity")

Warning: The following requested variables were not found (10 out of 32 shown):
ZK381.48, ZK218.23, W06H8.2, VF15C11L.2, T27E4.11, T20G5.15, sru-2, srt-23,
sls-2.4, sls-1.12

Scale for colour is already present.
Adding another scale for colour, which will replace the existing scale.

Fig.S8_Up.gof

Warning: Removed 1828 rows containing missing values or values outside the scale range
(`geom_point()`).

# -

# Manuscript figures

### - Fig.1 pt1

Fig.1_UMAP

ggsave("output/figures/Fig.1_UMAP.svg", height = 10, width = 10, units = "in", bg = "white", dpi = 300)

Fig.1_VlnUMI

ggsave("output/figures/Fig.1_VlnUMI.svg", height = 6, width = 9, units = "in", bg = "white", dpi = 300)

Fig.1_VlnFeature

ggsave("output/figures/Fig.1_VlnFeature.svg", height = 6, width = 9, units = "in", bg = "white", dpi = 300)

### - Fig.1 pt2 Feature plot pannel

Fig.1_mrk1 <- FeaturePlot(Fed_rpca_v2, reduction = "umap.rpca", features = c("mab-5"), label = F, order = T) +
 scale_color_gradientn(colors = c("#e1e1e1", "#edb304", "#edb304", "#e81a09", "#e81a09", "#330d96", "#330d96")) +
 ggtitle(expression(italic("mab-5"))) +
 labs(x = "UMAP 1", y = "UMAP 2")

Scale for colour is already present.
Adding another scale for colour, which will replace the existing scale.

Fig.1_mrk2 <- FeaturePlot(Fed_rpca_v2, reduction = "umap.rpca", features = c("lin-39"), label = F, order = T) +
 scale_color_gradientn(colors = c("#e1e1e1", "#edb304", "#edb304", "#e81a09", "#e81a09", "#330d96", "#330d96")) +
 ggtitle(expression(italic("lin-39"))) +
 labs(x = "UMAP 1", y = "UMAP 2")

Scale for colour is already present.
Adding another scale for colour, which will replace the existing scale.

Fig.1_mrk3 <- FeaturePlot(Fed_rpca_v2, reduction = "umap.rpca", features = c("mig-21"), label = F, order = T) +
 scale_color_gradientn(colors = c("#e1e1e1", "#edb304", "#edb304", "#e81a09", "#e81a09", "#330d96", "#330d96")) +
 ggtitle(expression(italic("mig-21"))) +
 labs(x = "UMAP 1", y = "UMAP 2")

Scale for colour is already present.
Adding another scale for colour, which will replace the existing scale.

Fig.1_mrk4 <- FeaturePlot(Fed_rpca_v2, reduction = "umap.rpca", features = c("egl-13"), label = F, order = T) +
 scale_color_gradientn(colors = c("#e1e1e1", "#edb304", "#edb304", "#e81a09", "#e81a09", "#330d96", "#330d96")) +
 ggtitle(expression(italic("egl-13"))) +
 labs(x = "UMAP 1", y = "UMAP 2")

Scale for colour is already present.
Adding another scale for colour, which will replace the existing scale.

Fig.1_mrk5 <- FeaturePlot(Fed_rpca_v2, reduction = "umap.rpca", features = c("gcy-32"), label = F, order = T) +
 scale_color_gradientn(colors = c("#e1e1e1", "#edb304", "#edb304", "#e81a09", "#e81a09", "#330d96", "#330d96")) +
 ggtitle(expression(italic("gcy-32"))) +
 labs(x = "UMAP 1", y = "UMAP 2")

Scale for colour is already present.
Adding another scale for colour, which will replace the existing scale.

Fig.1_mrk6 <- FeaturePlot(Fed_rpca_v2, reduction = "umap.rpca", features = c("mec-7"), label = F, order = T) +
 scale_color_gradientn(colors = c("#e1e1e1", "#edb304", "#edb304", "#e81a09", "#e81a09", "#330d96", "#330d96")) +
 ggtitle(expression(italic("mec-7"))) +
 labs(x = "UMAP 1", y = "UMAP 2")

Scale for colour is already present.
Adding another scale for colour, which will replace the existing scale.

Fig.1_mrk7 <- FeaturePlot(Fed_rpca_v2, reduction = "umap.rpca", features = c("mec-3"),label = F, order = T) +
 scale_color_gradientn(colors = c("#e1e1e1", "#edb304", "#edb304", "#e81a09", "#e81a09", "#330d96", "#330d96")) +
 ggtitle(expression(italic("mec-3"))) +
 labs(x = "UMAP 1", y = "UMAP 2")

Scale for colour is already present.
Adding another scale for colour, which will replace the existing scale.

Fig.1_mrk8 <- FeaturePlot(Fed_rpca_v2, reduction = "umap.rpca", features = c("lad-2"),label = F, order = T) +
 scale_color_gradientn(colors = c("#e1e1e1", "#edb304", "#edb304", "#e81a09", "#e81a09", "#330d96", "#330d96")) +
 ggtitle(expression(italic("lad-2"))) +
 labs(x = "UMAP 1", y = "UMAP 2")

Scale for colour is already present.
Adding another scale for colour, which will replace the existing scale.

(Fig.1_mrk1 | Fig.1_mrk2 | Fig.1_mrk3 | Fig.1_mrk4 ) /
(Fig.1_mrk5 | Fig.1_mrk6 | Fig.1_mrk7 | Fig.1_mrk8)

(Fig.1_mrk3 | Fig.1_mrk4 | Fig.1_mrk5 | Fig.1_mrk6 ) /
(Fig.1_mrk7 | Fig.1_mrk8 |Fig.1_mrk1 | Fig.1_mrk2)

ggsave("output/figures/Fig.1_FeatPlots.svg", height = 10, width = 20, units = "in", bg = "white", dpi = 300)

### - Fig.2

Fig.2_Pseudotime

ggsave("output/figures/Fig.2_Pseudotime.svg", height = 10, width = 11, units = "in", bg = "white", dpi = 300)

### - Fig.3

Fig.3_UMAP

ggsave("output/figures/Fig.3_UMAP.svg", height = 10, width = 11, units = "in", bg = "white", dpi = 300)

Fig.3_QVolcano

Warning: ggrepel: 84 unlabeled data points (too many overlaps). Consider
increasing max.overlaps

ggsave("output/figures/Fig.3_QVolcano.svg", height = 10, width = 10, units = "in", bg = "white", dpi = 300)

Warning: ggrepel: 84 unlabeled data points (too many overlaps). Consider
increasing max.overlaps

Fig.3_Qx_TFs

Warning: Removed 191 rows containing missing values or values outside the scale range
(`geom_point()`).

ggsave("output/figures/Fig.3_Qx_TFs.svg", height = 6, width = 7.5, units = "in", bg = "white", dpi = 300)

Warning: Removed 191 rows containing missing values or values outside the scale range
(`geom_point()`).

Fig.3_Qx_Pseudotime

Warning in scale_y_log10(): log-10 transformation introduced infinite values.

Warning in scale_y_log10(): log-10 transformation introduced infinite values.

ggsave("output/figures/Fig.3_Qx_Pseudotime.svg", height = 10, width = 5, units = "in", bg = "white", dpi = 300)

Warning in scale_y_log10(): log-10 transformation introduced infinite values.
log-10 transformation introduced infinite values.

### - Fig.4

Fig.4_UMAP

ggsave("output/figures/Fig.4_UMAP.svg", height = 10, width = 11, units = "in", bg = "white", dpi = 300)

Fig.4_QxVolcano

Warning: ggrepel: 1 unlabeled data points (too many overlaps). Consider
increasing max.overlaps

ggsave("output/figures/Fig.4_QxVolcano.svg", height = 10, width = 10, units = "in", bg = "white", dpi = 300)

Warning: ggrepel: 1 unlabeled data points (too many overlaps). Consider
increasing max.overlaps

Fig.4_Qx.aVolcano

Warning: ggrepel: 18 unlabeled data points (too many overlaps). Consider
increasing max.overlaps

ggsave("output/figures/Fig.4_Qx.aVolcano.svg", height = 10, width = 10, units = "in", bg = "white", dpi = 300)

Warning: ggrepel: 18 unlabeled data points (too many overlaps). Consider
increasing max.overlaps

Fig.4_Qx.pVolcano

Warning: ggrepel: 61 unlabeled data points (too many overlaps). Consider
increasing max.overlaps

ggsave("output/figures/Fig.4_Qx.pVolcano.svg", height = 10, width = 10, units = "in", bg = "white", dpi = 300)

Warning: ggrepel: 61 unlabeled data points (too many overlaps). Consider
increasing max.overlaps

Fig.4_Qx.apVolcano

Warning: ggrepel: 182 unlabeled data points (too many overlaps). Consider
increasing max.overlaps

ggsave("output/figures/Fig.4_Qx.apVolcano.svg", height = 10, width = 10, units = "in", bg = "white", dpi = 300)

Warning: ggrepel: 182 unlabeled data points (too many overlaps). Consider
increasing max.overlaps

Fig.4_Qx.paVolcano

Warning: ggrepel: 88 unlabeled data points (too many overlaps). Consider
increasing max.overlaps

ggsave("output/figures/Fig.4_Qx.paVolcano.svg", height = 10, width = 10, units = "in", bg = "white", dpi = 300)

Warning: ggrepel: 88 unlabeled data points (too many overlaps). Consider
increasing max.overlaps

### - Fig.5

Fig.5_UMAP

ggsave("output/figures/Fig.5_UMAP.svg", height = 10, width = 11, units = "in", bg = "white", dpi = 300)

Fig.5_APVolcano

Warning: ggrepel: 485 unlabeled data points (too many overlaps). Consider
increasing max.overlaps

ggsave("output/figures/Fig.5_APVolcano.svg", height = 10, width = 10, units = "in", bg = "white", dpi = 300)

Warning: ggrepel: 485 unlabeled data points (too many overlaps). Consider
increasing max.overlaps

ggsave("output/figures/Fig.5_APVolcano.png", height = 10, width = 10, units = "in", bg = "white", dpi = 300)

Warning: ggrepel: 485 unlabeled data points (too many overlaps). Consider
increasing max.overlaps

Fig.5_Heatmap

ggsave("output/figures/Fig.5_Heatmap.svg", height = 10, width = 10, units = "in", bg = "white", dpi = 300)
ggsave("output/figures/Fig.5_Heatmap.png", height = 20, width = 18, units = "in", bg = "white", dpi = 300)

### - Fig.6 pt 1 Dotplot

Fig.6_Wnt

Warning: Removed 327 rows containing missing values or values outside the scale range
(`geom_point()`).

ggsave("output/figures/Fig.6_Wnt.svg", height = 5, width = 15, units = "in", bg = "white", dpi = 300)

Warning: Removed 327 rows containing missing values or values outside the scale range
(`geom_point()`).

ggsave("output/figures/Fig.6_Wnt.png", height = 5, width = 15, units = "in", bg = "white", dpi = 300)

Warning: Removed 327 rows containing missing values or values outside the scale range
(`geom_point()`).

Fig.6_Wnt_flip

Warning: Removed 327 rows containing missing values or values outside the scale range
(`geom_point()`).

ggsave("output/figures/Fig.6_Wnt_flip.png", height = 16, width = 8, units = "in", bg = "white", dpi = 600)

Warning: Removed 327 rows containing missing values or values outside the scale range
(`geom_point()`).

ggsave("output/figures/Fig.6_Wnt_flip.svg", height = 16, width = 8, units = "in", bg = "white", dpi = 300)

Warning: Removed 327 rows containing missing values or values outside the scale range
(`geom_point()`).

### - Fig.6 pt 2 Feature plot pannel

Fig.6_cwn1 <- FeaturePlot(Fed_rpca_v2, reduction = "umap.rpca", features = c("cwn-1"), label = F, order = T) +
 scale_color_gradientn(colors = c("#e1e1e1", "#edb304", "#edb304", "#e81a09", "#e81a09", "#330d96", "#330d96")) +
 ggtitle(expression(italic("cwn-1"))) +
 labs(x = "UMAP 1", y = "UMAP 2")

Scale for colour is already present.
Adding another scale for colour, which will replace the existing scale.

Fig.6_cwn2 <- FeaturePlot(Fed_rpca_v2, reduction = "umap.rpca", features = c("cwn-2"), label = F, order = T) +
 scale_color_gradientn(colors = c("#e1e1e1", "#edb304", "#edb304", "#e81a09", "#e81a09", "#330d96", "#330d96")) +
 ggtitle(expression(italic("cwn-2"))) +
 labs(x = "UMAP 1", y = "UMAP 2")

Scale for colour is already present.
Adding another scale for colour, which will replace the existing scale.

Fig.6_bar1 <- FeaturePlot(Fed_rpca_v2, reduction = "umap.rpca", features = c("bar-1"), label = F, order = T) +
 scale_color_gradientn(colors = c("#e1e1e1", "#edb304", "#edb304", "#e81a09", "#e81a09", "#330d96", "#330d96")) +
 ggtitle(expression(italic("bar-1"))) +
 labs(x = "UMAP 1", y = "UMAP 2")

Scale for colour is already present.
Adding another scale for colour, which will replace the existing scale.

Fig.6_pop1 <- FeaturePlot(Fed_rpca_v2, reduction = "umap.rpca", features = c("pop-1"), label = F, order = T) +
 scale_color_gradientn(colors = c("#e1e1e1", "#edb304", "#edb304", "#e81a09", "#e81a09", "#330d96", "#330d96")) +
 ggtitle(expression(italic("pop-1"))) +
 labs(x = "UMAP 1", y = "UMAP 2")

Scale for colour is already present.
Adding another scale for colour, which will replace the existing scale.

Fig.6_sys1 <- FeaturePlot(Fed_rpca_v2, reduction = "umap.rpca", features = c("sys-1"), label = F, order = T) +
 scale_color_gradientn(colors = c("#e1e1e1", "#edb304", "#edb304", "#e81a09", "#e81a09", "#330d96", "#330d96")) +
 ggtitle(expression(italic("sys-1"))) +
 labs(x = "UMAP 1", y = "UMAP 2")

Scale for colour is already present.
Adding another scale for colour, which will replace the existing scale.

Fig.6_wrm1 <- FeaturePlot(Fed_rpca_v2, reduction = "umap.rpca", features = c("wrm-1"), label = F, order = T) +
 scale_color_gradientn(colors = c("#e1e1e1", "#edb304", "#edb304", "#e81a09", "#e81a09", "#330d96", "#330d96")) +
 ggtitle(expression(italic("wrm-1"))) +
 labs(x = "UMAP 1", y = "UMAP 2")

Scale for colour is already present.
Adding another scale for colour, which will replace the existing scale.

(Fig.6_cwn1 | Fig.6_cwn2 | Fig.6_bar1)/
 (Fig.6_pop1 | Fig.6_sys1 | Fig.6_wrm1)

ggsave("output/figures/Fig.6_FeatPlots.svg", height = 10, width = 15, units = "in", bg = "white", dpi = 300)

(Fig.6_cwn1 | Fig.6_cwn2 )/
 (Fig.6_bar1 | Fig.6_pop1) /
 (Fig.6_sys1 | Fig.6_wrm1)

ggsave("output/figures/Fig.6_FeatPlots_Flip.svg", height = 15, width = 10, units = "in", bg = "white", dpi = 300)

## - S1 Appendix info

### –Table 1 - UMI and gene count/cluster

print(median_umi)

median_UMI
1 1216.552

print(mean_umi)

mean_UMI
1 1268.816

print(median_umi_per_cluster)

# A tibble: 13 × 2
 Q.cell_clusters median_UMI
 <fct> <dbl>
 1 Qx 2104.
 2 QL 2313.
 3 QR 2422.
 4 QL.a 1078.
 5 QR.a 1301.
 6 QL.p 1106.
 7 QR.p 1134.
 8 QL.ap 1217.
 9 QR.ap 1290.
10 QL.pa 1031.
11 QR.pa 1099.
12 Qx.paa 714.
13 Qx.pap 452.

print(mean_umi_per_cluster)

# A tibble: 13 × 2
 Q.cell_clusters mean_UMI
 <fct> <dbl>
 1 Qx 2170.
 2 QL 2291.
 3 QR 2407.
 4 QL.a 1120.
 5 QR.a 1274.
 6 QL.p 1122.
 7 QR.p 1128.
 8 QL.ap 1213.
 9 QR.ap 1289.
10 QL.pa 1040.
11 QR.pa 1091.
12 Qx.paa 724.
13 Qx.pap 454.

print(median_genes)

median_genes
1 580

print(median_genes_per_cluster)

# A tibble: 13 × 2
 Q.cell_clusters median_genes
 <fct> <dbl>
 1 Qx 930.
 2 QL 1050
 3 QR 1114.
 4 QL.a 595
 5 QR.a 649
 6 QL.p 610.
 7 QR.p 636
 8 QL.ap 531
 9 QR.ap 519
10 QL.pa 560
11 QR.pa 587
12 Qx.paa 421
13 Qx.pap 240

### –Table 2 - Cell counts in each cluster

table(Idents(Fed_rpca_v2))

Qx QL QR QL.a QR.a QL.ap QR.ap QL.p QR.p QL.pa QR.pa
 272 183 132 231 511 1054 1451 1328 533 284 642
Qx.paa Qx.pap
 97 25

table(Fed_rpca_v2$Q.cell_v2_clusters)

Qx Qx.a Qx.p Qx.ap Qx.pa Qx.paa Qx.pap
 587 742 1861 2505 926 97 25

table(Fed_rpca_v2$experiment_clusters)

OP50 OP50.2 PA14
 1964 2418 2361

### –Figure S1 - Annotation markers (UMAPs)

Fig.S1_egl17 <- FeaturePlot(Fed_rpca_v2, reduction = "umap.rpca", features = c("egl-17"), label = F, order = T) +
 scale_color_gradientn(colors = c("#e1e1e1", "#edb304", "#edb304", "#e81a09", "#e81a09", "#330d96", "#330d96")) +
 ggtitle(expression(italic("egl-17")))+
 labs(x = "UMAP 1", y = "UMAP 2")

Scale for colour is already present.
Adding another scale for colour, which will replace the existing scale.

Fig.S1_gcy35 <- FeaturePlot(Fed_rpca_v2, reduction = "umap.rpca", features = c("gcy-35"), label = F, order = T) +
 scale_color_gradientn(colors = c("#e1e1e1", "#edb304", "#edb304", "#e81a09", "#e81a09", "#330d96", "#330d96")) +
 ggtitle(expression(italic("gcy-35")))+
 labs(x = "UMAP 1", y = "UMAP 2")

Scale for colour is already present.
Adding another scale for colour, which will replace the existing scale.

Fig.S1_gcy36 <- FeaturePlot(Fed_rpca_v2, reduction = "umap.rpca", features = c("gcy-36"), label = F, order = T) +
 scale_color_gradientn(colors = c("#e1e1e1", "#edb304", "#edb304", "#e81a09", "#e81a09", "#330d96", "#330d96")) +
 ggtitle(expression(italic("gcy-36")))+
 labs(x = "UMAP 1", y = "UMAP 2")

Scale for colour is already present.
Adding another scale for colour, which will replace the existing scale.

Fig.S1_mec18 <- FeaturePlot(Fed_rpca_v2, reduction = "umap.rpca", features = c("mec-18"), label = F, order = T) +
 scale_color_gradientn(colors = c("#e1e1e1", "#edb304", "#edb304", "#e81a09", "#e81a09", "#330d96", "#330d96")) +
 ggtitle(expression(italic("mec-18")))+
 labs(x = "UMAP 1", y = "UMAP 2")

Scale for colour is already present.
Adding another scale for colour, which will replace the existing scale.

Fig.S1_ceh31 <- FeaturePlot(Fed_rpca_v2, reduction = "umap.rpca", features = c("ceh-31"), label = F, order = T) +
 scale_color_gradientn(colors = c("#e1e1e1", "#edb304", "#edb304", "#e81a09", "#e81a09", "#330d96", "#330d96")) +
 ggtitle(expression(italic("ceh-31")))+
 labs(x = "UMAP 1", y = "UMAP 2")

Scale for colour is already present.
Adding another scale for colour, which will replace the existing scale.

Fig.S1_ZK265.7 <- FeaturePlot(Fed_rpca_v2, reduction = "umap.rpca", features = c("ZK265.7"), label = F, order = T) +
 scale_color_gradientn(colors = c("#e1e1e1", "#edb304", "#edb304", "#e81a09", "#e81a09", "#330d96", "#330d96")) +
 ggtitle(expression(italic("ZK265.7")))+
 labs(x = "UMAP 1", y = "UMAP 2")

Scale for colour is already present.
Adding another scale for colour, which will replace the existing scale.

Fig.S1_ceh43 <- FeaturePlot(Fed_rpca_v2, reduction = "umap.rpca", features = c("ceh-43"), label = F, order = T) +
 scale_color_gradientn(colors = c("#e1e1e1", "#edb304", "#edb304", "#e81a09", "#e81a09", "#330d96", "#330d96")) +
 ggtitle(expression(italic("ceh-43")))+
 labs(x = "UMAP 1", y = "UMAP 2")

Scale for colour is already present.
Adding another scale for colour, which will replace the existing scale.

Fig.S1_mec4 <- FeaturePlot(Fed_rpca_v2, reduction = "umap.rpca", features = c("mec-4"), label = F, order = T) +
 scale_color_gradientn(colors = c("#e1e1e1", "#edb304", "#edb304", "#e81a09", "#e81a09", "#330d96", "#330d96")) +
 ggtitle(expression(italic("mec-4")))+
 labs(x = "UMAP 1", y = "UMAP 2")

Scale for colour is already present.
Adding another scale for colour, which will replace the existing scale.

(Fig.S1_egl17 | Fig.S1_gcy35 | Fig.S1_gcy36 | Fig.S1_mec18) /
 (Fig.S1_ceh31 | Fig.S1_ZK265.7 | Fig.S1_ceh43 | Fig.S1_mec4)

ggsave("output/figures/S1.Fig.tiff", height = 10, width = 20, units = "in", bg = "white", dpi = 300, compression = "lzw")


Fig.S1_experiments <- DimPlot(Fed_rpca_v2, reduction = "umap.rpca", label = F, group.by = "experiment_clusters", order = F, cols = c("gold", "#e8000d", "#0051ba"), pt.size = 0.1) +
 ggtitle("Experiments")+
 labs(x = "UMAP 1", y = "UMAP 2")


(Fig.S1_egl17 | Fig.S1_gcy35 | Fig.S1_gcy36) /
 (Fig.S1_mec18 | Fig.S1_ceh31 | Fig.S1_ZK265.7) /
 (Fig.S1_ceh43 | Fig.S1_mec4 | Fig.S1_experiments)

ggsave("output/figures/S1.Fig.v2.svg", height = 15, width = 15, units = "in", bg = "white", dpi = 300)

### –Figure S2 - EMT markers (UMAPs)

Fig.S2_ajm1 <- FeaturePlot(Fed_rpca_v2, reduction = "umap.rpca", features = c("ajm-1"), label = F, order = T) +
 scale_color_gradientn(colors = c("#e1e1e1", "#edb304", "#edb304", "#e81a09", "#e81a09", "#330d96", "#330d96")) +
 ggtitle(expression(italic("ajm-1")))+
 labs(x = "UMAP 1", y = "UMAP 2")

Scale for colour is already present.
Adding another scale for colour, which will replace the existing scale.

Fig.S2_zag1 <- FeaturePlot(Fed_rpca_v2, reduction = "umap.rpca", features = c("zag-1"), label = F, order = T) +
 scale_color_gradientn(colors = c("#e1e1e1", "#edb304", "#edb304", "#e81a09", "#e81a09", "#330d96", "#330d96")) +
 ggtitle(expression(italic("zag-1")))+
 labs(x = "UMAP 1", y = "UMAP 2")

Scale for colour is already present.
Adding another scale for colour, which will replace the existing scale.

(Fig.S2_ajm1 | Fig.S2_zag1 )

ggsave("output/figures/S2.Fig.tiff", height = 5, width = 10, units = "in", bg = "white", dpi = 300, compression = "lzw")

### –Figure S3 - Early Q markers (UMAPs)

Fig.S3_cdh3 <- FeaturePlot(Fed_rpca_v2, reduction = "umap.rpca", features = c("cdh-3"), label = F, order = T) +
 scale_color_gradientn(colors = c("#e1e1e1", "#edb304", "#edb304", "#e81a09", "#e81a09", "#330d96", "#330d96")) +
 ggtitle(expression(italic("cdh-3")))+
 labs(x = "UMAP 1", y = "UMAP 2")

Scale for colour is already present.
Adding another scale for colour, which will replace the existing scale.

Fig.S3_cdh4 <- FeaturePlot(Fed_rpca_v2, reduction = "umap.rpca", features = c("cdh-4"), label = F, order = T) +
 scale_color_gradientn(colors = c("#e1e1e1", "#edb304", "#edb304", "#e81a09", "#e81a09", "#330d96", "#330d96")) +
 ggtitle(expression(italic("cdh-4")))+
 labs(x = "UMAP 1", y = "UMAP 2")

Scale for colour is already present.
Adding another scale for colour, which will replace the existing scale.

Fig.S3_unc40 <- FeaturePlot(Fed_rpca_v2, reduction = "umap.rpca", features = c("unc-40"), label = F, order = T) +
 scale_color_gradientn(colors = c("#e1e1e1", "#edb304", "#edb304", "#e81a09", "#e81a09", "#330d96", "#330d96")) +
 ggtitle(expression(italic("unc-40")))+
 labs(x = "UMAP 1", y = "UMAP 2")

Scale for colour is already present.
Adding another scale for colour, which will replace the existing scale.

Fig.S3_mig15 <- FeaturePlot(Fed_rpca_v2, reduction = "umap.rpca", features = c("mig-15"), label = F, order = T) +
 scale_color_gradientn(colors = c("#e1e1e1", "#edb304", "#edb304", "#e81a09", "#e81a09", "#330d96", "#330d96")) +
 ggtitle(expression(italic("mig-15")))+
 labs(x = "UMAP 1", y = "UMAP 2")

Scale for colour is already present.
Adding another scale for colour, which will replace the existing scale.

Fig.S3_ptp3 <- FeaturePlot(Fed_rpca_v2, reduction = "umap.rpca", features = c("ptp-3"), label = F, order = T) +
 scale_color_gradientn(colors = c("#e1e1e1", "#edb304", "#edb304", "#e81a09", "#e81a09", "#330d96", "#330d96")) +
 ggtitle(expression(italic("ptp-3")))+
 labs(x = "UMAP 1", y = "UMAP 2")

Scale for colour is already present.
Adding another scale for colour, which will replace the existing scale.

Fig.S3_dpy19 <- FeaturePlot(Fed_rpca_v2, reduction = "umap.rpca", features = c("dpy-19"), label = F, order = T) +
 scale_color_gradientn(colors = c("#e1e1e1", "#edb304", "#edb304", "#e81a09", "#e81a09", "#330d96", "#330d96")) +
 ggtitle(expression(italic("dpy-19")))+
 labs(x = "UMAP 1", y = "UMAP 2")

Scale for colour is already present.
Adding another scale for colour, which will replace the existing scale.

(Fig.S3_cdh3 | Fig.S3_cdh4 | Fig.S3_unc40 ) /
 (Fig.S3_mig15 | Fig.S3_ptp3 | Fig.S3_dpy19)

ggsave("output/figures/S3.Fig.tiff", height = 10, width = 15, units = "in", bg = "white", dpi = 300, compression = "lzw")

### –Figure S4 - L-R markers (UMAPs)

Fig.S4_lec4 <- FeaturePlot(Fed_rpca_v2, reduction = "umap.rpca", features = c("lec-4"), label = F, order = T) +
 scale_color_gradientn(colors = c("#e1e1e1", "#edb304", "#edb304", "#e81a09", "#e81a09", "#330d96", "#330d96")) +
 ggtitle(expression(italic("lec-4")))+
 labs(x = "UMAP 1", y = "UMAP 2")

Scale for colour is already present.
Adding another scale for colour, which will replace the existing scale.

Fig.S4_mex3 <- FeaturePlot(Fed_rpca_v2, reduction = "umap.rpca", features = c("mex-3"), label = F, order = T) +
 scale_color_gradientn(colors = c("#e1e1e1", "#edb304", "#edb304", "#e81a09", "#e81a09", "#330d96", "#330d96")) +
 ggtitle(expression(italic("mex-3")))+
 labs(x = "UMAP 1", y = "UMAP 2")

Scale for colour is already present.
Adding another scale for colour, which will replace the existing scale.

Fig.S4_cav1 <- FeaturePlot(Fed_rpca_v2, reduction = "umap.rpca", features = c("cav-1"), label = F, order = T) +
 scale_color_gradientn(colors = c("#e1e1e1", "#edb304", "#edb304", "#e81a09", "#e81a09", "#330d96", "#330d96")) +
 ggtitle(expression(italic("cav-1")))+
 labs(x = "UMAP 1", y = "UMAP 2")

Scale for colour is already present.
Adding another scale for colour, which will replace the existing scale.

Fig.S4_lgc52 <- FeaturePlot(Fed_rpca_v2, reduction = "umap.rpca", features = c("lgc-52"), label = F, order = T) +
 scale_color_gradientn(colors = c("#e1e1e1", "#edb304", "#edb304", "#e81a09", "#e81a09", "#330d96", "#330d96")) +
 ggtitle(expression(italic("lgc-52")))+
 labs(x = "UMAP 1", y = "UMAP 2")

Scale for colour is already present.
Adding another scale for colour, which will replace the existing scale.

Fig.S4_srd30 <- FeaturePlot(Fed_rpca_v2, reduction = "umap.rpca", features = c("srd-30"), label = F, order = T) +
 scale_color_gradientn(colors = c("#e1e1e1", "#edb304", "#edb304", "#e81a09", "#e81a09", "#330d96", "#330d96")) +
 ggtitle(expression(italic("srd-30")))+
 labs(x = "UMAP 1", y = "UMAP 2")

Scale for colour is already present.
Adding another scale for colour, which will replace the existing scale.

Fig.S4_srd29 <- FeaturePlot(Fed_rpca_v2, reduction = "umap.rpca", features = c("srd-29"), label = F, order = T) +
 scale_color_gradientn(colors = c("#e1e1e1", "#edb304", "#edb304", "#e81a09", "#e81a09", "#330d96", "#330d96")) +
 ggtitle(expression(italic("srd-29")))+
 labs(x = "UMAP 1", y = "UMAP 2")

Scale for colour is already present.
Adding another scale for colour, which will replace the existing scale.

Fig.S4_vab8 <- FeaturePlot(Fed_rpca_v2, reduction = "umap.rpca", features = c("vab-8"), label = F, order = T) +
 scale_color_gradientn(colors = c("#e1e1e1", "#edb304", "#edb304", "#e81a09", "#e81a09", "#330d96", "#330d96")) +
 ggtitle(expression(italic("vab-8")))+
 labs(x = "UMAP 1", y = "UMAP 2")

Scale for colour is already present.
Adding another scale for colour, which will replace the existing scale.

Fig.S4_ssq1 <- FeaturePlot(Fed_rpca_v2, reduction = "umap.rpca", features = c("ssq-1"), label = F, order = T) +
 scale_color_gradientn(colors = c("#e1e1e1", "#edb304", "#edb304", "#e81a09", "#e81a09", "#330d96", "#330d96")) +
 ggtitle(expression(italic("ssq-1")))+
 labs(x = "UMAP 1", y = "UMAP 2")

Scale for colour is already present.
Adding another scale for colour, which will replace the existing scale.

Fig.S4_T22B7.22 <- FeaturePlot(Fed_rpca_v2, reduction = "umap.rpca", features = c("T22B7.22"), label = F, order = T) +
 scale_color_gradientn(colors = c("#e1e1e1", "#edb304", "#edb304", "#e81a09", "#e81a09", "#330d96", "#330d96")) +
 ggtitle(expression(italic("T22B7.22")))+
 labs(x = "UMAP 1", y = "UMAP 2")

Scale for colour is already present.
Adding another scale for colour, which will replace the existing scale.

Fig.S4_H37A05.4 <- FeaturePlot(Fed_rpca_v2, reduction = "umap.rpca", features = c("H37A05.4"), label = F, order = T) +
 scale_color_gradientn(colors = c("#e1e1e1", "#edb304", "#edb304", "#e81a09", "#e81a09", "#330d96", "#330d96")) +
 ggtitle(expression(italic("H37A05.4")))+
 labs(x = "UMAP 1", y = "UMAP 2")

Scale for colour is already present.
Adding another scale for colour, which will replace the existing scale.

Fig.S4_unc5 <- FeaturePlot(Fed_rpca_v2, reduction = "umap.rpca", features = c("unc-5"), label = F, order = T) +
 scale_color_gradientn(colors = c("#e1e1e1", "#edb304", "#edb304", "#e81a09", "#e81a09", "#330d96", "#330d96")) +
 ggtitle(expression(italic("unc-5")))+
 labs(x = "UMAP 1", y = "UMAP 2")

Scale for colour is already present.
Adding another scale for colour, which will replace the existing scale.

Fig.S4_Y45G5AM.5 <- FeaturePlot(Fed_rpca_v2, reduction = "umap.rpca", features = c("Y45G5AM.5"), label = F, order = T) +
 scale_color_gradientn(colors = c("#e1e1e1", "#edb304", "#edb304", "#e81a09", "#e81a09", "#330d96", "#330d96")) +
 ggtitle(expression(italic("Y45G5AM.5")))+
 labs(x = "UMAP 1", y = "UMAP 2")

Scale for colour is already present.
Adding another scale for colour, which will replace the existing scale.

Fig.S4_grl10 <- FeaturePlot(Fed_rpca_v2, reduction = "umap.rpca", features = c("grl-10"), label = F, order = T) +
 scale_color_gradientn(colors = c("#e1e1e1", "#edb304", "#edb304", "#e81a09", "#e81a09", "#330d96", "#330d96")) +
 ggtitle(expression(italic("grl-10")))+
 labs(x = "UMAP 1", y = "UMAP 2")

Scale for colour is already present.
Adding another scale for colour, which will replace the existing scale.

Fig.S4_C01C4.3 <- FeaturePlot(Fed_rpca_v2, reduction = "umap.rpca", features = c("C01C4.3"), label = F, order = T) +
 scale_color_gradientn(colors = c("#e1e1e1", "#edb304", "#edb304", "#e81a09", "#e81a09", "#330d96", "#330d96")) +
 ggtitle(expression(italic("C01C4.3")))+
 labs(x = "UMAP 1", y = "UMAP 2")

Scale for colour is already present.
Adding another scale for colour, which will replace the existing scale.

Fig.S4_mig13 <- FeaturePlot(Fed_rpca_v2, reduction = "umap.rpca", features = c("mig-13"), label = F, order = T) +
 scale_color_gradientn(colors = c("#e1e1e1", "#edb304", "#edb304", "#e81a09", "#e81a09", "#330d96", "#330d96")) +
 ggtitle(expression(italic("mig-13")))+
 labs(x = "UMAP 1", y = "UMAP 2")

Scale for colour is already present.
Adding another scale for colour, which will replace the existing scale.

Fig.S4_cam1 <- FeaturePlot(Fed_rpca_v2, reduction = "umap.rpca", features = c("cam-1"), label = F, order = T) +
 scale_color_gradientn(colors = c("#e1e1e1", "#edb304", "#edb304", "#e81a09", "#e81a09", "#330d96", "#330d96")) +
 ggtitle(expression(italic("cam-1")))+
 labs(x = "UMAP 1", y = "UMAP 2")

Scale for colour is already present.
Adding another scale for colour, which will replace the existing scale.

Fig.S4_bath15 <- FeaturePlot(Fed_rpca_v2, reduction = "umap.rpca", features = c("bath-15"), label = F, order = T) +
 scale_color_gradientn(colors = c("#e1e1e1", "#edb304", "#edb304", "#e81a09", "#e81a09", "#330d96", "#330d96")) +
 ggtitle(expression(italic("bath-15")))+
 labs(x = "UMAP 1", y = "UMAP 2")

Scale for colour is already present.
Adding another scale for colour, which will replace the existing scale.

Fig.S4_M162.5 <- FeaturePlot(Fed_rpca_v2, reduction = "umap.rpca", features = c("M162.5"), label = F, order = T) +
 scale_color_gradientn(colors = c("#e1e1e1", "#edb304", "#edb304", "#e81a09", "#e81a09", "#330d96", "#330d96")) +
 ggtitle(expression(italic("M162.5")))+
 labs(x = "UMAP 1", y = "UMAP 2")

Scale for colour is already present.
Adding another scale for colour, which will replace the existing scale.

Fig.S4_F54F12.2 <- FeaturePlot(Fed_rpca_v2, reduction = "umap.rpca", features = c("F54F12.2"), label = F, order = T) +
 scale_color_gradientn(colors = c("#e1e1e1", "#edb304", "#edb304", "#e81a09", "#e81a09", "#330d96", "#330d96")) +
 ggtitle(expression(italic("F54F12.2")))+
 labs(x = "UMAP 1", y = "UMAP 2")

Scale for colour is already present.
Adding another scale for colour, which will replace the existing scale.

(Fig.S4_lec4 | Fig.S4_mex3 | Fig.S4_cav1 | Fig.S4_lgc52)/
 (Fig.S4_srd30 | Fig.S4_srd29 | Fig.S4_vab8 | Fig.S4_ssq1) /
 (Fig.S4_T22B7.22 | Fig.S4_H37A05.4 | Fig.S4_unc5 | Fig.S4_Y45G5AM.5) /
 (Fig.S4_grl10 | Fig.S4_C01C4.3 | Fig.S4_mig13 | Fig.S4_cam1) /
 (Fig.S4_bath15 | Fig.S4_M162.5 | Fig.S4_F54F12.2 |plot_spacer())

ggsave("output/figures/S4.Fig.tiff", height = 25, width = 20, units = "in", bg = "white", dpi = 300, compression = "lzw")

### –Figure S5-S8 - mab-5 mutants

Fig.S5_Down.lof

Warning: Removed 345 rows containing missing values or values outside the scale range
(`geom_point()`).

#ggsave("output/figures/S5_Fig.tiff", height = 8, width = 7.5, units = "in", bg = "white", dpi = 300, compression = "lzw")
ggsave("output/figures/S5_Fig.svg", height = 8, width = 7.5, units = "in", bg = "white", dpi = 300)

Warning: Removed 345 rows containing missing values or values outside the scale range
(`geom_point()`).

Fig.S6_Up.lof

Warning: Removed 563 rows containing missing values or values outside the scale range
(`geom_point()`).

#ggsave("output/figures/S6_Fig.tiff", height = 10, width = 7.5, units = "in", bg = "white", dpi = 300, compression = "lzw")
ggsave("output/figures/S6_Fig.svg", height = 10, width = 7.5, units = "in", bg = "white", dpi = 300)

Warning: Removed 563 rows containing missing values or values outside the scale range
(`geom_point()`).

Fig.S7_Down.gof

Warning: Removed 865 rows containing missing values or values outside the scale range
(`geom_point()`).

#ggsave("output/figures/S7_Fig.tiff", height = 18, width = 7.5, units = "in", bg = "white", dpi = 300, compression = "lzw")
ggsave("output/figures/S7_Fig.svg", height = 18, width = 7.5, units = "in", bg = "white", dpi = 300)

Warning: Removed 865 rows containing missing values or values outside the scale range
(`geom_point()`).

Fig.S8_Up.gof

Warning: Removed 1828 rows containing missing values or values outside the scale range
(`geom_point()`).

#ggsave("output/figures/S8_Fig.tiff", height = 35, width = 7.5, units = "in", bg = "white", dpi = 300, compression = "lzw")
ggsave("output/figures/S8_Fig.svg", height = 35, width = 7.5, units = "in", bg = "white", dpi = 300,)

Warning: Removed 1828 rows containing missing values or values outside the scale range
(`geom_point()`).

### –Figure S9 - A-P markers (UMAPs) | Heatmap added after PLOS ONE revision

Fig.S9_unc86 <- FeaturePlot(Fed_rpca_v2, reduction = "umap.rpca", features = c("unc-86"), label = F, order = T) +
 scale_color_gradientn(colors = c("#e1e1e1", "#edb304", "#edb304", "#e81a09", "#e81a09", "#330d96", "#330d96")) +
 ggtitle(expression(italic("unc-86")))+
 labs(x = "UMAP 1", y = "UMAP 2")

Scale for colour is already present.
Adding another scale for colour, which will replace the existing scale.

Fig.S9_lin32 <- FeaturePlot(Fed_rpca_v2, reduction = "umap.rpca", features = c("lin-32"), label = F, order = T) +
 scale_color_gradientn(colors = c("#e1e1e1", "#edb304", "#edb304", "#e81a09", "#e81a09", "#330d96", "#330d96")) +
 ggtitle(expression(italic("lin-32")))+
 labs(x = "UMAP 1", y = "UMAP 2")

Scale for colour is already present.
Adding another scale for colour, which will replace the existing scale.

Fig.S9_R05H11.2 <- FeaturePlot(Fed_rpca_v2, reduction = "umap.rpca", features = c("R05H11.2"), label = F, order = T) +
 scale_color_gradientn(colors = c("#e1e1e1", "#edb304", "#edb304", "#e81a09", "#e81a09", "#330d96", "#330d96")) +
 ggtitle(expression(italic("R05H11.2")))+
 labs(x = "UMAP 1", y = "UMAP 2")

Scale for colour is already present.
Adding another scale for colour, which will replace the existing scale.

Fig.S9_lipl7 <- FeaturePlot(Fed_rpca_v2, reduction = "umap.rpca", features = c("lipl-7"), label = F, order = T) +
 scale_color_gradientn(colors = c("#e1e1e1", "#edb304", "#edb304", "#e81a09", "#e81a09", "#330d96", "#330d96")) +
 ggtitle(expression(italic("lipl-7")))+
 labs(x = "UMAP 1", y = "UMAP 2")

Scale for colour is already present.
Adding another scale for colour, which will replace the existing scale.

Fig.S9_F32H5.3 <- FeaturePlot(Fed_rpca_v2, reduction = "umap.rpca", features = c("F32H5.3"), label = F, order = T) +
 scale_color_gradientn(colors = c("#e1e1e1", "#edb304", "#edb304", "#e81a09", "#e81a09", "#330d96", "#330d96")) +
 ggtitle(expression(italic("F32H5.3")))+
 labs(x = "UMAP 1", y = "UMAP 2")

Scale for colour is already present.
Adding another scale for colour, which will replace the existing scale.

Fig.S9_hlh14 <- FeaturePlot(Fed_rpca_v2, reduction = "umap.rpca", features = c("hlh-14"), label = F, order = T) +
 scale_color_gradientn(colors = c("#e1e1e1", "#edb304", "#edb304", "#e81a09", "#e81a09", "#330d96", "#330d96")) +
 ggtitle(expression(italic("hlh-14")))+
 labs(x = "UMAP 1", y = "UMAP 2")

Scale for colour is already present.
Adding another scale for colour, which will replace the existing scale.

(Fig.S9_unc86 | Fig.S9_lin32 | Fig.S9_R05H11.2)/
 (Fig.S9_lipl7 | Fig.S9_F32H5.3 | Fig.S9_hlh14)

ggsave("output/figures/S9_Fig.tiff", height = 10, width = 15, units = "in", bg = "white", dpi = 300, compression = "lzw")

Fig.S9_Heatmap

ggsave("output/figures/Fig.S9_Heatmap.svg", height = 10, width = 10, units = "in", bg = "white", dpi = 300)
ggsave("output/figures/Fig.S9_Heatmap.png", height = 20, width = 18, units = "in", bg = "white", dpi = 300)

### –Figure S10 - Wnt

Fig.S10_mig1 <- FeaturePlot(Fed_rpca_v2, reduction = "umap.rpca", features = c("mig-1"), label = F, order = T) +
 scale_color_gradientn(colors = c("#e1e1e1", "#edb304", "#edb304", "#e81a09", "#e81a09", "#330d96", "#330d96")) +
 ggtitle(expression(italic("mig-1")))+
 labs(x = "UMAP 1", y = "UMAP 2")

Scale for colour is already present.
Adding another scale for colour, which will replace the existing scale.

Fig.S10_lin17 <- FeaturePlot(Fed_rpca_v2, reduction = "umap.rpca", features = c("lin-17"), label = F, order = T) +
 scale_color_gradientn(colors = c("#e1e1e1", "#edb304", "#edb304", "#e81a09", "#e81a09", "#330d96", "#330d96")) +
 ggtitle(expression(italic("lin-17")))+
 labs(x = "UMAP 1", y = "UMAP 2")

Scale for colour is already present.
Adding another scale for colour, which will replace the existing scale.

Fig.S10_mom5 <- FeaturePlot(Fed_rpca_v2, reduction = "umap.rpca", features = c("mom-5"), label = F, order = T) +
 scale_color_gradientn(colors = c("#e1e1e1", "#edb304", "#edb304", "#e81a09", "#e81a09", "#330d96", "#330d96")) +
 ggtitle(expression(italic("mom-5")))+
 labs(x = "UMAP 1", y = "UMAP 2")

Scale for colour is already present.
Adding another scale for colour, which will replace the existing scale.

Fig.S10_cam1 <- FeaturePlot(Fed_rpca_v2, reduction = "umap.rpca", features = c("cam-1"), label = F, order = T) +
 scale_color_gradientn(colors = c("#e1e1e1", "#edb304", "#edb304", "#e81a09", "#e81a09", "#330d96", "#330d96")) +
 ggtitle(expression(italic("cam-1")))+
 labs(x = "UMAP 1", y = "UMAP 2")

Scale for colour is already present.
Adding another scale for colour, which will replace the existing scale.

(Fig.S10_mig1 | Fig.S10_lin17) /
 (Fig.S10_mom5 | Fig.S10_cam1)

ggsave("output/figures/S10_Fig.tiff", height = 10, width = 10, units = "in", bg = "white", dpi = 300, compression = "lzw")
