# Supplementary material for: Single-cell transcriptomic profiling of C. elegans Q neuroblast lineage during migration and differentiation
Source: PLoS One. 2026 Mar 3;21(3):e0343734. doi: 10.1371/journal.pone.0343734 (PMC12956130; doi:10.1371/journal.pone.0343734)
Supplement: Attachment — Submitted filename: Response to reviewers.pdf [file pone.0343734.s020.pdf]

Dear Editor and Reviewer,

We thank you for your evaluation of our manuscript and for the constructive comments provided. We have revised the manuscript accordingly. Below, we provide a point-by-point response detailing the changes made in the revised version. Line numbers refer to the clean version of the revised manuscript.

Response to Editorial Comments

1. PLOS ONE formatting requirements

Headings (levels 1 and 2) have been updated to match PLOS ONE's formatting guidelines.

2. Code sharing

Although our analyses rely primarily on existing R packages (e.g., Seurat) and do not involve novel code, we now provide a rendered R notebook containing all code and corresponding output as a new supplementary file (S6 File) to ensure reproducibility. The Methods section (lines 649-650) has been updated accordingly.

3. Grant information (Funding Information' vs 'Financial Disclosure')

We corrected the Funding Information in the submission form.

4. Funding information in the Acknowledgments section

Funding information has been removed from the Acknowledgments section, as requested.

5. Reviewer-suggested citations

Not applicable.

6. Reference list

Reference list was reviewed, and citations were updated.

- Reference 33: formatting corrected.
- Reference 69: updated from preprint to final published version.

Additional comments

Errors in gene labeling in the S5–S8 dot plots were identified and corrected in this revised version.

Response to Reviewer #1 comments

We appreciate the reviewer's thoughtful evaluation of our work and the constructive suggestions provided. All comments were carefully considered, and the resulting changes are detailed below.

Comment #1: "*If the authors were open to additional analyses, it could be useful to compare the early Q cells to seam cells (the authors report that seam cells are present in the data and were filtered out during QC). Some of the TFs mentioned as potential regulators of early aspects of Q biology like the "EMT-like" delamination (lines 264-269) such as nhr-25 are broadly expressed across epidermal cells. Finding TFs enriched in Q relative to seam cells might help predict those more likely to be instructive for the Q-specific cell behaviors.*"

Response: We have performed a comparison between early Q cells and seam cells, which revealed, for example, that *nhr-25* is upregulated in early Q cells relative to their seam cell sister V5. Because these findings relate more directly to the earlier developmental stage and to mechanistic aspects of Q-cell specification we are currently working on, we plan to present them in a follow-up study focused on these processes.

Comment #2. *"The annotations in Figure 1C are reasonable but it is maybe worth acknowledging in the text that the precise temporal boundaries (such as between Q and Q.a or Q.p) are inferred from expression differences so could be temporally offset from the true division time by a small amount."*

Response: We have expanded Figure 1C legend to clarify this issue (lines 56-58).

Comment #3: *"Fig 5B – this is a nice display, and it could be useful to include a version as supplement that separated lineages by LR as well (showing markers identified with the higher resolution lineage annotations) to more clearly show how LR and lineage(a/p) differences relate to each other"*

Response: We have added an LR-segregated version of Fig 5B to the S9 Fig and adjusted the text accordingly (line 381).

Comment #4: *"Line 426 – could rephrase to more clearly indicate EGL-20 is a Wnt ligand"*

Response: We have adjusted the text (lines 428-429) to explicitly indicate that EGL-20 functions as a Wnt ligand.

Comment #5: *"Lines 118-128 – I suggest reducing the discussion of high mitochondrial cells being Qx.aa an Qx.pp given this is uncertain and maybe not of major importance to the paper"*

Response: This paragraph (lines 121-126) has been substantially reduced to briefly report the observation without drawing speculative conclusions.

Comment #6: *"Fig. 2 – If possible I suggest giving more descriptive names to the modules (Group 3 could be "Q.a", "Group 4" could be "Q" etc) for clarity."*

Response: The module group names in Fig 2 have been updated to add more descriptive labels to figures (e.g., "Group 1" to "Group 1: Final descendants") and improve clarity. We retained the original numbering system since it is required for interpretation of the module analyses in S1 file.

Comment #7: *"Fig 2B (wormcat enrichments) should ideally describe in the legend what criteria were used for enrichment (P value cutoff, fold enrichment, etc)"*

Response: The Fig 2B legend (lines 181-183) now specifies that enrichment was performed using WormCat's standard settings (Fisher's exact test with Bonferroni correction; $P < 0.01$) and that categories shown represent the broadest functional level (Cat1) provided by WormCat.
