# Supplementary material for: Single-cell transcriptomic profiling of C. elegans Q neuroblast lineage during migration and differentiation
Source: PLoS One. 2026 Mar 3;21(3):e0343734. doi: 10.1371/journal.pone.0343734 (PMC12956130; doi:10.1371/journal.pone.0343734)
Supplement: Attachment — Submitted filename: Response to reviewers 2nd round - v2.pdf [file pone.0343734.s021.pdf]

Dear Editor and Reviewer,

We appreciate your thoughtful evaluation of our manuscript and the constructive feedback you've provided. In response, we have made the necessary revisions to the manuscript, with the specific changes outlined below.

Response to Reviewer #2 comments

We appreciate the reviewer's incredibly thorough review and the suggestions provided. All comments were carefully considered, and the resulting changes are detailed below.

Comment #1: "*This dataset was comprised of three separate experiments. From the entry at the SRA archive of the raw files and the R notebook, it became clear that one of the three samples was from worms fed PA14, while the other two were fed OP50. PA14 is toxic to C. elegans. The use of different bacterial food sources for one of the samples needs to be explicitly addressed in the methods. Ideally, differential expression analysis should be performed between food sources within each cell type to identify possible gene expression differences resulting from food source that could confound the results, especially if some cell types preferentially arise from this sample. If no differences in gene expression are seen, this can be noted. At the very least, the possibility of effects should be noted and the rationale for different food sources justified.*"

Response: We thank the reviewer for pointing this out. We agree that the difference in bacterial food source should be explicitly documented and have now revised the Methods (Lines 612-615; 695) and Results (Lines 94-95; 131-134) sections to clearly describe the different food sources, the rationale for their use, and the analyses performed.

Importantly, analyses assessing potential effects of food source were conducted as part of the original data analysis but were previously documented only in the rendered analysis notebook and not explicitly described in the manuscript text. We have now made these analyses more accessible and explicit in the manuscript and supplement (S1 Fig; new S1 File).

We did not observe obvious clustering or cell-type composition differences attributable to food source. Differential expression analyses comparing PA14- and OP50-fed animals did not reveal significant shifts in global transcriptional profiles across cell types or systematic changes in established marker genes associated with food source. Consistent with this, cells from experiments 1 and 2 (performed with the identical parameters except for the bacterial food sources) presented a nearly identical clustering pattern upon merging of the two datasets, even prior to formal dataset integration. These results suggest that differences in bacterial food source did not measurably confound cell-type identity or marker gene expression in this dataset.

Comment #2. *"The fact that three samples were used and integrated should be mentioned much earlier in the methods."*

Response: We now mention the three samples earlier in the Methods, both during sample preparation (Lines 612-615) and at the beginning of the data processing steps (Lines 660), and in the Results (Lines 94-95). The initial mention of dataset integration was expanded on line 668 to improve clarity.

Comment #3: *"I recommend the authors pursue subsetting the data to see if they can more clearly resolve L/R cells in the Q cells and Q.p lineages. This is a common approach in scRNA-seq analysis. In particular, rerunning dimensionality reduction (PCA and UMAP) on the cells without the Q.a lineages may allow for more distinct separation of L/R populations in the remaining lineages."*

Response: Subsetting analyses of this type were part of our initial analysis workflow. We performed targeted subsetting of Q lineage cells, including, for example, analyses in which all other cell types were excluded or in which only anterior or posterior lineage cells were retained, and reran dimensionality reduction to assess whether L/R asymmetry could be more clearly resolved, but these analyses did not result in improved separation of left/right populations relative to the full dataset, as assessed by cell clustering and expression of known L/R markers. Because these results did not alter our interpretation or conclusions, they were not included in the original manuscript to maintain focus and avoid unnecessary complexity.

Comment #4: *"No methods are provided or context given to the identification of the "co-expressed gene modules" described beginning on line 187. Therefore, it is difficult to interpret the meaning and conclusions of this analysis. Please provide more detail about how these modules were identified."*

Response: We used Monocle3's graph_test function to find genes that vary between clusters followed by find_gene_modules to predict co-regulated genes and group them into modules. Details were added to the Methods (Lines 703-704) and Results (Lines 195-196) sections.

Comment #5: *"The authors mention a "post-sort check" on lines 122-123 that "indicated high viability of sorted cells," but there is no direct description of this "check" in the methods. It could be part of the cell counting on the hemocytometer prior to 10X processing, but this should be clarified. Please describe what criteria were used to assess viability and health."*

Response: More information about the post-sort check was added to the Methods section (Lines 646-649).

Comment #6: *"Lines 380-383. The issue of markers associated with Qx.p being depleted can easily be remedied by changing the parameters of the FindAllMarkers function, setting the parameter "only.pos" to TRUE. If left as false, it will detect both enrichment (positive log2fold change) and depletion (negative log2fold change). This can be used to identify positive markers. Qx.p will likely have fewer than 10 top markers."*

Response: We thank the reviewer for the helpful suggestion. Following the recommendation, we performed the "only.pos" analysis by adjusting the FindAllMarkers function and have included the results in the S5 file. This analysis highlighted new top 10 markers for Qx.p, but as expected, these markers are not exclusive to Qx.p and seem to have a relatively high expression in the parent Q cells as well. We have retained the original figure, as we believe it is important to also highlight the depleted genes, particularly when they appear as top markers. This provides additional context for understanding the marker dynamics in Qx.p, as depletion may still carry biological significance. The text has been revised to mention the positive markers and to increase simplicity, as suggested in comment #7 (Lines 391-394; 402; 407).

Comment #7: *"The analysis described in 372-383 uses a one group vs all others approach. It may not be surprising, then, that the differentiated neuron group Qx.ap (consisting of AQR/PQR) shows a higher number of differentially expressed genes when compared to all the other groups, which are almost entirely transient neuroblast populations. I think this section can be simplified by highlight only the positive markers for each lineal group vs others (see comment above) and moving directly into the analysis between Qx.a and Qx.p featured in the next paragraph."*

Response: The paragraph was shortened for simplicity, as described above.

Comment #8: *"The volcano plots indicate thresholds for both log2 fold change and p-values were used in determining differential gene expression. The exact thresholds used need to be reported explicitly in the Methods."*

Response: The thresholds were added to methods (Lines 697-698).

Comment #9: *"The set of genes used as the background for the WormCat enrichment comparisons should be reported. The proper set of genes for this comparison should be limited to genes that are detected in the dataset, rather than the entire set of genes with WormCat annotations."*

Response: We thank the reviewer for pointing this out. We agree that enrichment analyses based on differential expression typically benefit from restricting the background to genes detectable in the dataset. In this study, however, WormCat was used to broadly describe biological programs in Monocle3 gene modules, not for hypothesis-driven enrichment testing. Because WormCat 2.0 uses a fixed genome-wide background, enrichment outputs were considered descriptive aids for functional interpretation, not hypothesis-driven statistical tests. We have clarified both the fixed background used by WormCat and the interpretive scope of this analysis in the Results (Lines 190; 207-210) and Methods (Lines 704-707) sections.

Comment #10: *"Lines 401-402. The anterior lineage does differentiate earlier than the posterior lineage, as known from the initial lineage tracing from Sulston and colleagues."*

Response: We thank the reviewer for pointing this out, as our original wording was ambiguous and did not convey our intended meaning. We have reworded the sentence (Lines 410-412) to clarify that our observation reflects differences in cell state between the two Q daughter cells, with Qx.p retaining a neuroblast-like transition state while Qx.a shows stronger transcriptional signatures associated with neuronal differentiation, even before Qx.ap is born.

Comment #11: *"Figures S5, S6, S7, and S8 might be more easily interpretable with respect to L/R differences if the cell types on the x-axis were grouped by L/R and then sorted by lineal generation."*

Response: We thank the reviewer for the suggestion. Figures S5, S6, S7, and S8 were updated accordingly.

Comment #12: *"The authors appear to use zag-1 expression as an indicator of epithelial identity, but its highest expression is in Q.p progenitors, which arise after the purported EMT in this lineage. There is no explanation of this with respect to the argument of EMT."*

Response: We have reworded the text to clarify the interpretation of *zag-1* expression in the Q lineage. While members of the *zag-1* transcription factor family are known to regulate EMT in vertebrates, *zag-1* in *C. elegans* has also been implicated in neuronal differentiation. We now explicitly acknowledge this dual role and clarify that *zag-1* expression in Q.p progenitors is consistent with a function in later neuronal differentiation, rather than being used solely as an indicator of epithelial identity or EMT (Lines 248-254).

Comment #13: *"The authors mention the inconsistency of egl-17 expression as a reason for low counts of some postmitotic neurons and reference their own single-cell data. It would be more convincing as a reason for the low sampling to see images of the reporter strain in the various Q lineage progenitors, as it is the levels of GFP from the reporter that directly affect sorting efficiency."*

Response: Based on recent CeNGEN data analysis, we now believe that the developmental time window post-hatching is the primary factor contributing to the low counts of Qx.pax and have updated the manuscript accordingly (Lines 114-117; 121-124). We continue to mention the transient nature of *egl-17* expression, as the expression dynamics of a major lineage marker remain relevant, and note that contribution to cell count discrepancy, particularly between Qx.paa and Qx.pap, cannot be excluded.

We have imaged worms at this stage and confirmed that GFP from the *Pegl-17::gfp* reporter is detectable in early Qx.paa and Qx.pap, although at lower intensity than in progenitor cells. However, upregulation of *Pegl-17::gfp* in P cells around this time complicates tracking GFP dynamics in Qx.pax beyond the earliest stages.

Comment #14: *"In abstract: lines 23-25: The use of the word "mechanisms" suggests experiments were done to test a role for novel Wnt-related genes in this manuscript. This might be more accurate if changed from "mechanisms" to "expression"."*

Response: The text has been revised as suggested.

Comment #15: *"There are multiple instances where the word "on" should be changed to "in." See Lines 42, 91, 243, and possibly others."*

Response: All mentioned instances have been corrected, and the manuscript was reviewed for additional occurrences.

Comment #16: *"Lines 112-113: "The final descendants of the posterior Q cell lineage, Qx.paa and Qx.pap, were the smaller population in our dataset" – change to "smallest populations"."*

Response: The text has been revised as suggested.

Comment #17: *"Line 657 – "Clustering was performed with UMAP" UMAP is a dimensionality reduction algorithm, not a clustering algorithm. The UMAP algorithm does not assign cells into discrete clusters. The clustering algorithm used (or function in Seurat) should be explicitly described."*

Response: We thank the reviewer for pointing that out. We have revised the text to accurately distinguish clustering from dimensionality reduction (Lines 675-676; 684-685).

Comment #18: *"Line 137 – "transcription factor (TFs)" should be "transcription factor (TF)""*

Response: The text has been revised as suggested.

Comment #19: *"Lines 202-203 "goblin" should be "globin.""*

Response: The text has been revised as suggested.

Comment #20: *"Lines 203-203 "confirming the differentiation" would be better as "consistent with the differentiation""*

Response: The text has been revised as suggested.

Comment #21: *"Line 302 – should read "similar to mammalian caveolin-1 and caveolin-3""*

Response: The text has been revised as suggested.

Comment #22: *"Lines 325-326 change "expressive" to "enriched""*

Response: The text has been revised as suggested.

Comment #23: *"Line 353 – This sentence made it sound like the authors were looking at genes that were commonly downregulated by both lof and gof mab-5 mutations, but this is not the case. This first sentence should be reworded to clarify."*

Response: This sentence has been reworded to clarify the intended interpretation and avoid potential misinterpretation.

Comment #24: *"Line 381 – "top 10 genes expressed" should be "top 10 genes enriched" or "top 10 genes differentially expressed""*

Response: This text has been removed following revisions made in response to Comments #6 and #7.

Comment #25: *"Line 395 – "seemed previously" – maybe this should be "seen previously""*

Response: This text has been removed following revisions made in response to Comments #6 and #7.

Comment #26: *"Sentence beginning at line 469 is a run-on and should split into separate sentences."*

Response: The sentence has been revised and split into separate sentences for clarity.

Comment #27: *"Multiple issues with plural vs singular agreement (lines 476, 483, possibly elsewhere). Please check for and correct these instances."*

Response: The singular/plural agreement has been corrected in line 476 (now line 486).

On line 483 (now line 492), the phrase "as it migrates and differentiates into neurons" refers to "the entire Q neuroblast lineage", with the portion about the descendants being parenthetical. These sentences have been rearranged to improve readability and grammatical clarity.

Comment #28: *"Line 491 "cells segregation into clusters" reads awkwardly."*

Response: The wording has been revised to "cluster assignment" to improve clarity.

Additional comments to the editor

- We added more details to the WormEnrichr methods (Lines 708-709).
- The reference list was reviewed, and Reference 34 has been updated from citing a website entry to citing a published preprint.
- Files S1-S6 have been renumbered as S2-S7 to accommodate the new S1 file created in response to Reviewer Comment #1.
- S1 Fig legend was updated to accommodate new panel.
